# Time trends in ethnic inequalities in child health and nutrition: analysis of 59 low and middle-income countries

**DOI:** 10.1186/s12939-023-01888-5

**Published:** 2023-04-28

**Authors:** Luis Paulo Vidaletti, Bianca O. Cata-Preta, David E. Phillips, Sonya Shekhar, Aluísio J.D. Barros, Cesar G. Victora

**Affiliations:** 1 International Center for Equity in Health, Federal University of Pelotas, Rua Marechal Deodoro 1160, Pelotas, Pelotas, RS 96020-220 Brazil; 2 Postgraduate Program in Epidemiology, Federal University of Pelotas, Rua Marechal Deodoro 1160, Pelotas, Pelotas, RS 96020-220 Brazil; 3 Gates Ventures, 4110 Carillon Pt, Kirkland, Seattle, WA 98033-7463 USA

**Keywords:** Ethnicity, Child health, Inequality, Stunting, Child mortality

## Abstract

**Background:**

Although ethnicity is a key social determinant of health, there are no global analyses aimed at identifying countries that succeeded in reducing ethnic gaps in child health and nutrition.

**Methods:**

We identified 59 low and middle-income countries with at least two surveys since 2010 providing information on ethnicity or language and on three outcomes: under-five mortality, child stunting prevalence and a composite index (CCI) based on coverage with eight maternal and child health interventions. Firstly, we calculated population-weighted and unweighted measures of inequality among ethnic or language groups within each country. These included the mean difference from the overall national mean (absolute inequality), mean ratio relative to the overall mean (relative inequality), and the difference and ratio between the best- and worst-performing ethnic groups. Second, we examined annual changes in these measures in terms of annual absolute and relative changes. Thirdly, we compared trends for each of the three outcome indicators and identified exemplar countries with marked progress in reducing inequalities.

**Results:**

For each outcome indicator, annual changes in summary measures tended to show moderate (Pearson correlation coefficients of 0.4 to 0.69) or strong correlations (0.7 or higher) among themselves, and we thus focused on four of the 12 measures: absolute and relative annual changes in mean differences and ratios from the overall national mean. On average, absolute ethnic or language group inequalities tended to decline slightly for the three outcomes, and relative inequality declined for stunting and CCI, but increased for mortality. Correlations for annual trends across the three outcomes were inconsistent, with several countries showing progress in terms of one outcome but not in others. Togo and Uganda showed with the most consistent progress in reducing inequality, whereas the worst performers were Nigeria, Moldova, Kyrgyzstan, Sao Tome and Principe, and Burkina Faso.

**Conclusions:**

Although measures of annual changes in ethnic or language group inequalities in child health were consistently correlated within each outcome, analyses of such inequalities should rely upon multiple measures. Countries showing progress in one child health outcome did not necessarily show improvements in the remaining outcomes. In-depth analyses at country level are needed to understand the drivers of success in reducing ethnic gaps.

**Supplementary Information:**

The online version contains supplementary material available at 10.1186/s12939-023-01888-5.

## Background

Ethnic group affiliation (also referred to as race, tribe or caste) is widely recognized as a major social determinant of health. Given its influence on culture, diet, language, and ancestry, ethnicity is associated with variations in health beliefs and behaviors. In most if not all countries, ethnicity is also associated with unequal access to socio-economic opportunities, to health information and to health services. [1–3] Nevertheless, whereas much attention has been given to the study of child health inequalities according to wealth, maternal education, sex and place of residence in low and middle-income countries (LMICS), [4–6] limited information is available on ethnic gaps in child health and nutrition. [6–16].

Our review of the LMIC literature did not identify any multicountry analyses of time trends in child health and nutrition outcomes according to ethnic groups. It is important to learn which countries, if any, managed to reduce ethnic related health inequalities over time. Identification of such exemplar countries will help understand how existing disparities may be tackled by other LMICs. To help fill this gap, we analyzed how ethnic gaps evolved over time in 59 countries, with a focus on three outcomes. The first two, child stunting and under-five mortality, are Sustainable Development Goals (SDG) indicators 2.2.1 and 3.2.1, respectively. [17] The third indicator is the composite coverage index (CCI), [18] a summary indicator for coverage with reproductive, maternal, newborn and child health services, which is directly linked to SDG indicator 3.8.1. Our analyses are also relevant to SGD goals 10 on reducing inequality within and between countries, and to SDG 17.18 on building capacity for disaggregated analyses of progress towards the achievement of all SDGs.

Because there are several methodological approaches for assessing levels and trends in ethnic inequalities in health, [19, 20] the first part of our article entails a comparison of different summary measures of inequality. This is followed by presentation of results on ethnic trends in health for the 59 LMICs, aimed at identifying well-performing countries.

## Methods

The national surveys database at the International Center for Equity in Health (ICEH), which serves as the equity database for the Countdown to 2030 initiative (www.countdown2030.org) includes over 450 surveys carried out since the mid-1990s in 122 countries, of which 117 were LMICs. The database includes Demographic and Health Surveys (DHS), Multiple Indicator Cluster Survey (MICS) and a few non-standard national surveys. Further information on the methodology employed by these surveys is available elsewhere: DHS (https://dhsprogram.com/what-we-do/survey-Types/dHs.cfm) and MICS (http://mics.UNICEF.org/). Both types of surveys are highly comparable in terms of sampling and questionnaires. [21, 22] Within each sampled household, women aged 15–49 years provided information on their households and on children aged under five years.

When information on self-reported ethnicity was not available but there was information on language spoken at home, we used the latter as a proxy for ethnic group affiliation. [23, 24] A total of 59 countries in our database have more than one survey over time with information on ethnicity or language spoken at home regarding the women (in DHS) or the head of the household (in MICS), as well as information on at least one of the study outcomes described below. In 16 of the 59 countries, the information was on language spoken at home, whereas for the remaining countries the information referred to ethnicity or tribe. In Latin America and the Caribbean, for consistency with earlier analyses, we grouped the ethnic categories into three groups as follows: reference (mostly individuals with European, or mixed European and indigenous ancestry), indigenous and Afrodescendants. [25] We recoded groups with fewer than 50 children (or fewer than 250 births) into country-specific “other” categories; if the “other group” still included fewer than 50 children, these were excluded from all analyses. A detailed description of the ethnic or language groups in each country is available in Additional file 1.

Three outcomes were studied: stunting prevalence, the composite coverage index and under-five mortality rate.

Stunting prevalence was assessed for children aged under five years who had slept in the household in the night preceding the interview. Recumbent length was measured for children aged under 24 months and standing height for those aged 24–59 months. Length- or height-for-age *Z* scores were calculated using the WHO Growth Standards and children with Z scores below − 2 were classified as stunted. [26].

Coverage with essential interventions was assessed using the composite coverage index (CCI), a weighted average of eight indicators along four stages of the continuum of care: reproductive health (demand for family planning satisfied with modern methods), maternal health (at least four antenatal care visits and skilled birth attendance), child immunization (diphtheria-tetanus-pertussis, measles and Bacillus Calmette-Guérin vaccines); and management of child illness (oral rehydration for diarrhea and care-seeking for suspected pneumonia. All four stages have equal weights in this composite indicator. [18].

Under-five mortality rates (U5MR) were estimated for children born alive in the 10 years preceding the surveys using the Stata (Statacorp, USA) syncmrates procedure. [27] Mortality is expressed in deaths per 1,000 live births.

The first step in the analyses consisted of calculating the values of the above three outcomes for each ethnic group in the first and last available surveys. Using these values, six ethnic inequality summary measures suitable for unordered population groups (as is the case for ethnicity) were calculated for each survey, of which three measured absolute inequality and three relative inequality: [19].

- WMDOM: weighted mean difference from overall mean (absolute).
- WMROM: weighted mean ratio to overall mean (relative).
- UMDOM: unweighted mean difference from overall mean (absolute).
- UMROM: unweighted mean ratio to overall mean (relative).
- HLD: high-low difference among the best- and worst-performing groups (absolute).
- HLR: high-low ratio among the best- and worst-performing groups (relative).

Mean differences were obtained by subtracting the value of the outcome in each ethnic group from the overall national sample mean value, and then dividing the sum of absolute differences by the number of groups. A similar approach was used for calculating mean ratios using division rather than subtraction. Weighted summary measures consider the sample size of each ethnic group in their calculation, while unweighted measures do not. Further details are provided elsewhere. [19].

To assess changes in inequality along time, we calculated average annual absolute and relative changes between the first and last surveys per country. [28] Absolute changes were expressed as percent points (pp) per year, by dividing difference between levels of the summary measures in the two surveys by the number of years elapsed. Relative (compound) annual changes were estimated by

$$\left(\sqrt[n]{\raisebox{1ex}{$end$}\!\left/ \!\raisebox{-1ex}{$start$}\right.}-1\right)\times 100$$

where n is the number of years elapsed between the first and last surveys, end is the endline (last survey) estimate and start is the baseline (first survey) estimate. Negative values for absolute or relative change indicate reductions in ethnic inequalities.

After calculating annual absolute and relative changes, the results from the 59 countries for each outcome were standardized with a mean of zero and a standard deviation of one. Correlation matrices for the 12 continuous estimates of change over time for each outcome were calculated using Pearson’s correlation coefficients.

All analyses accounted for sampling weights and clustering. Ethical clearance was the responsibility of the institutions that administered the surveys and all analyses relied on anonymized databases.

## Results

Information on 316 ethnic groups was available in the 59 countries, with a median of five groups per country (range 2–16). The median interval between surveys was of 11 years (range 3–20) with median dates in 2005 (range 2000–2014) for the first and 2017 (2010–2020) for the last survey. Additional file 2 shows details from the surveys and sample sizes, and Additional file 3 shows the values of the indices for the first and last surveys as well as for annual changes per country. According to the UNICEF classification of world regions, there were 21 countries from West & Central Africa, 11 from Eastern Europe & Central Asia, 10 from Latin America & Caribbean, 8 from Eastern & Southern Africa, 6 from East Asia & Pacific, and 3 from South Asia.

Table 1 shows three correlation matrices (for stunting, CCI and U5MR) including the six summary measures, each expressed in terms of annual absolute or relative changes. Within each outcome, annual changes in summary measures tended to be highly correlated among themselves, whether weighted or unweighted, whether inequality was measured in absolute or relative scales, and also whether change was expressed in absolute or relative terms. Changes in extreme group differences and ratios were moderately or strongly associated with changes in the summary measures. Results were consistent for stunting, CCI and U5MR (Table 1), although some of the correlations for CCI were weaker, particularly for the extreme group ratio.

**Table 1:** Pearson correlation matrix for average annual changes in ethnic inequality measures for stunting prevalence, the composite coverage index and under-five mortality rate

			Absolute change						Relative change					
			WMDOM (absolute inequality)	WRDOM (relative inequality)	UMDOM (absolute inequality)	URDOM (relative inequality)	HLD (absolute)	HLR (relative)	WMDOM (absolute inequality)	WRDOM (relative inequality)	UMDOM (absolute inequality)	URDOM (relative inequality)	HLD (absolute)	HLR (relative)
**Stunting prevalence**	**absolute change**	**WMDOM**	1											
		**WMROM**	0.852	1										
		**UMDOM**	0.648	0.660	1									
		**UMROM**	0.558	0.757	0.867	1								
		**HLD**	0.591	0.503	0.912	0.743	1							
		**HLR**	0.458	0.531	0.799	0.829	0.839	1						
	**relative change**	**WMDOM**	0.886	0.873	0.622	0.601	0.533	0.45	1					
		**WMROM**	0.843	0.965	0.598	0.696	0.455	0.461	0.901	1				
		**UMDOM**	0.615	0.639	0.945	0.856	0.882	0.779	0.669	0.595	1			
		**UMROM**	0.553	0.711	0.891	0.958	0.787	0.786	0.583	0.664	0.907	1		
		**HLD**	0.550	0.521	0.892	0.781	0.945	0.799	0.596	0.492	0.948	0.844	1	
		**HLR**	0.471	0.541	0.825	0.841	0.869	0.99	0.461	0.484	0.804	0.821	0.831	1
**Composite coverage index**	**absolute change**	**WMDOM**	1											
		**WMROM**	0.833	1										
		**UMDOM**	0.773	0.660	1									
		**UMROM**	0.468	0.651	0.755	1								
		**HLD**	0.633	0.585	0.879	0.782	1							
		**HLR**	0.316	0.575	0.588	0.897	0.764	1						
	**relative change**	**WMDOM**	0.883	0.670	0.673	0.337	0.465	0.118	1					
		**WMROM**	0.844	0.793	0.650	0.469	0.468	0.284	0.934	1				
		**UMDOM**	0.693	0.539	0.93	0.678	0.799	0.471	0.716	0.663	1			
		**UMROM**	0.557	0.56	0.85	0.849	0.788	0.668	0.561	0.624	0.911	1		
		**HLD**	0.572	0.468	0.846	0.709	0.878	0.605	0.561	0.526	0.927	0.899	1	
		**HLR**	0.333	0.582	0.613	0.900	0.795	0.994	0.137	0.300	0.505	0.696	0.642	1
**Under-five mortality rate**	**absolute change**	**WMDOM**	1											
		**WMROM**	0.804	1										
		**UMDOM**	0.852	0.736	1									
		**UMROM**	0.640	0.736	0.818	1								
		**HLD**	0.666	0.635	0.900	0.782	1							
		**HLR**	0.482	0.587	0.727	0.840	0.806	1						
	**relative change**	**WMDOM**	0.933	0.797	0.88	0.69	0.738	0.575	1					
		**WMROM**	0.829	0.907	0.770	0.718	0.666	0.516	0.832	1				
		**UMDOM**	0.802	0.722	0.949	0.874	0.862	0.762	0.892	0.759	1			
		**UMROM**	0.688	0.764	0.843	0.959	0.808	0.789	0.757	0.813	0.908	1		
		**HLD**	0.654	0.639	0.884	0.831	0.946	0.833	0.774	0.679	0.926	0.873	1	
		**HLR**	0.508	0.620	0.767	0.892	0.840	0.978	0.609	0.581	0.813	0.859	0.872	1

Legend: Countries are the units of analysis. Green cells stand for correlations greater than 0.85. WMDOM: weighted mean difference from overall mean (absolute); WMROM: weighted mean ratio to overall mean (relative); UMDOM: unweighted mean difference from overall mean (absolute); UMROM: unweighted mean ratio to overall mean (relative); HLD: high-low difference among the best- and worst-performing groups (absolute); HLR: high-low ratio among the best- and worst-performing groups (relative).

Based upon the moderate to high correlations among the summary measures, we simplified the presentation of results by focusing on annual absolute changes in WMDOM and WMROM for the three outcomes. Table 2 shows the mean, median and standard deviation for absolute changes over time in all countries with data. Overall, absolute ethnic inequalities tended to decline slightly for the three outcomes, and relative inequality declined for stunting and CCI, but increased for U5MR. The large standard deviations suggest that annual changes were modest and highly variable among countries.

**Table 2:** Summary of average annual absolute changes in absolute and relative inequality measures, with countries as the units of analysis

	Annual absolute change					
	WMDOM (absolute inequality)			WMROM (relative inequality)		
Outcome	Mean	Median	SD	Mean	Median	SD
**Stunting**	-0.075	-0.076	0.192	-0.179	-0.016	0.772
**CCI**	-0.067	-0.043	0.200	-0.253	-0.159	0.476
**U5MR**	-0.059	-0.197	0.884	0.156	0.173	1.028

Legend: WMDOM:weighted mean difference from overall mean (absolute). WMROM:weighted mean ratio to overall mean (relative). CCI: composite coverage index. U5MR: under-five mortality rate. SD:– standard deviation.

Still using countries as the units of analyses, we explored variables associated with annual absolute changes in WMDOM and WMROM (data not shown). There were no consistent associations with baseline national outcome levels, number of ethnic groups, median number of children per ethnic group, nor the time elapsed between the two surveys. However, countries showing wider inequalities in the baseline survey tended to show faster reductions over time.

**Fig. 1:**
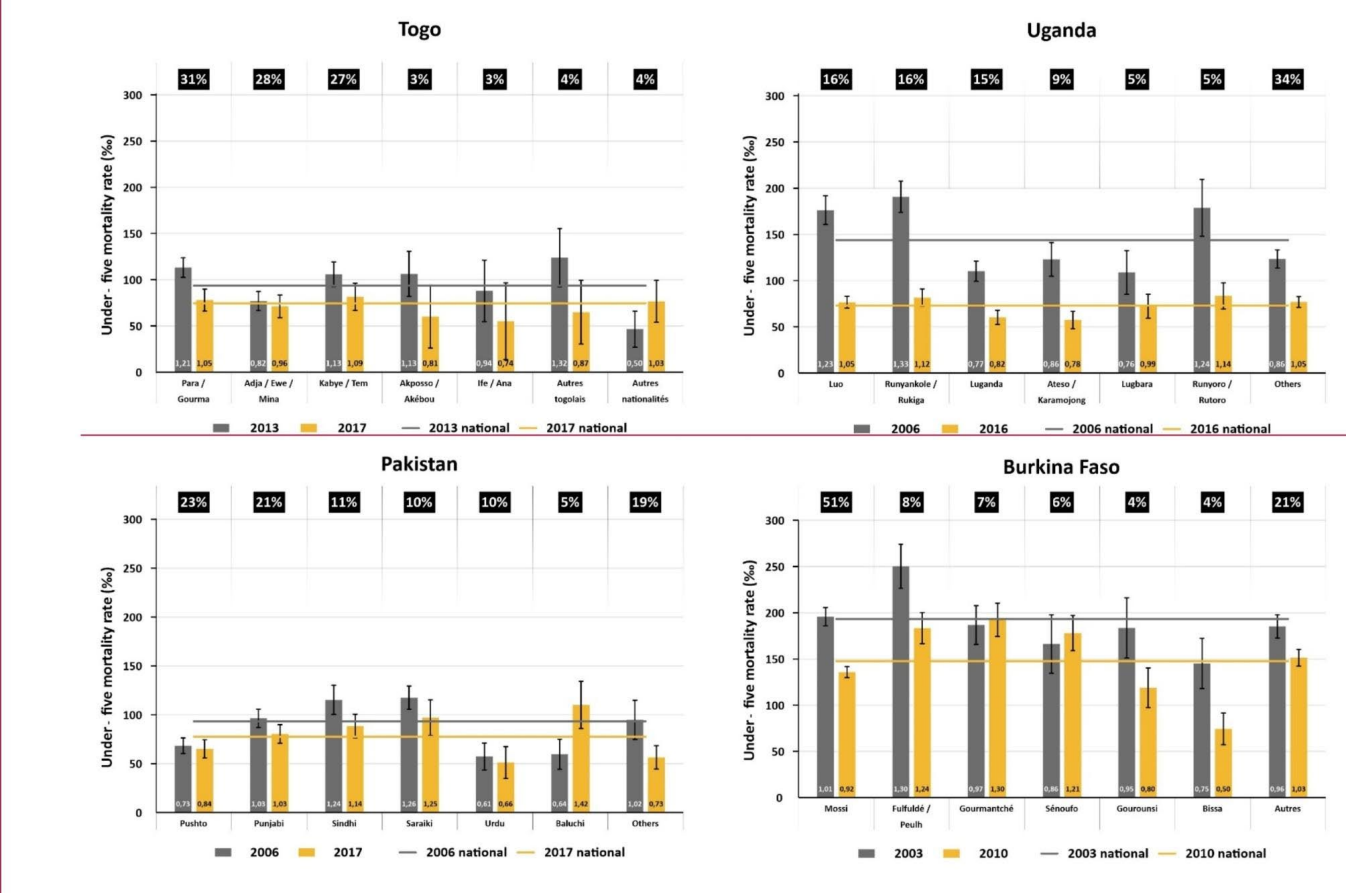
Under-five mortality rates prevalence by ethnic groups in the first and last surveys. Results for selected countries Legend: The numbers in the black rectangles show the average proportion of the samples for each ethnic groups in the two surveys. The numbers at the bottom of the bars show the ratio between the rate in a particular ethnic group and the national rate for that point in time.

To help interpret time trends in ethnic inequality, Fig. 1 shows four examples of changes over time in U5MR by ethnic group. Full results for the 59 countries are available in Additional file 3.

The bars show mortality rates in each group in the first and last surveys, and the horizontal lines show national prevalence for baseline and endline surveys. The numbers in black boxes show the average proportions of the sample in each ethnic group in the two surveys. The numbers in the bottom of the bars show the ratios between the rates in each group and national U5MR.

The two graphs on the top row show results for two countries where inequalities were reduced: Togo (absolute annual change of -3.1 in WMDOM and − 3.0 in WMROM; see Additional file 3) and Uganda (-2.3 and − 1.2, respectively). The height difference between bars in each pair show reductions in absolute inequality. In both countries, ethnic groups with higher baseline U5MR showed more marked reductions than those where baseline levels were lower. The ratios inside the bars show that, over time, ratios for the two largest ethnic groups relative to the national rate dropped substantially, showing a reduction in the relative inequality. Such changes were even more marked in Uganda, where ratios fell markedly for the ethnic groups with the highest baseline rates.

Still in Fig. 1, Pakistan provides an example of a country where changes over time were minimal: -0.3 for WMDOM and 0.0 for WMROM (Additional file 3). U5MR only fell very slightly over time at national level and for most ethnic groups, the exception being the Baluchi where mortality increased. The ratios inside the bars hardly changed (except again for the Baluchi), signaling that relative inequality remained constant.

Lastly in Fig. 1, Burkina Faso is an example of a country where inequalities were exacerbated: annual increases of 1.1 for WMDOM and 1.0 for WMROM (Additional file 3). Despite an important reduction in national U5MR, some ethnic groups experienced increases whereas most declined, leading to larger absolute and relative gaps.

**Fig. 2:**
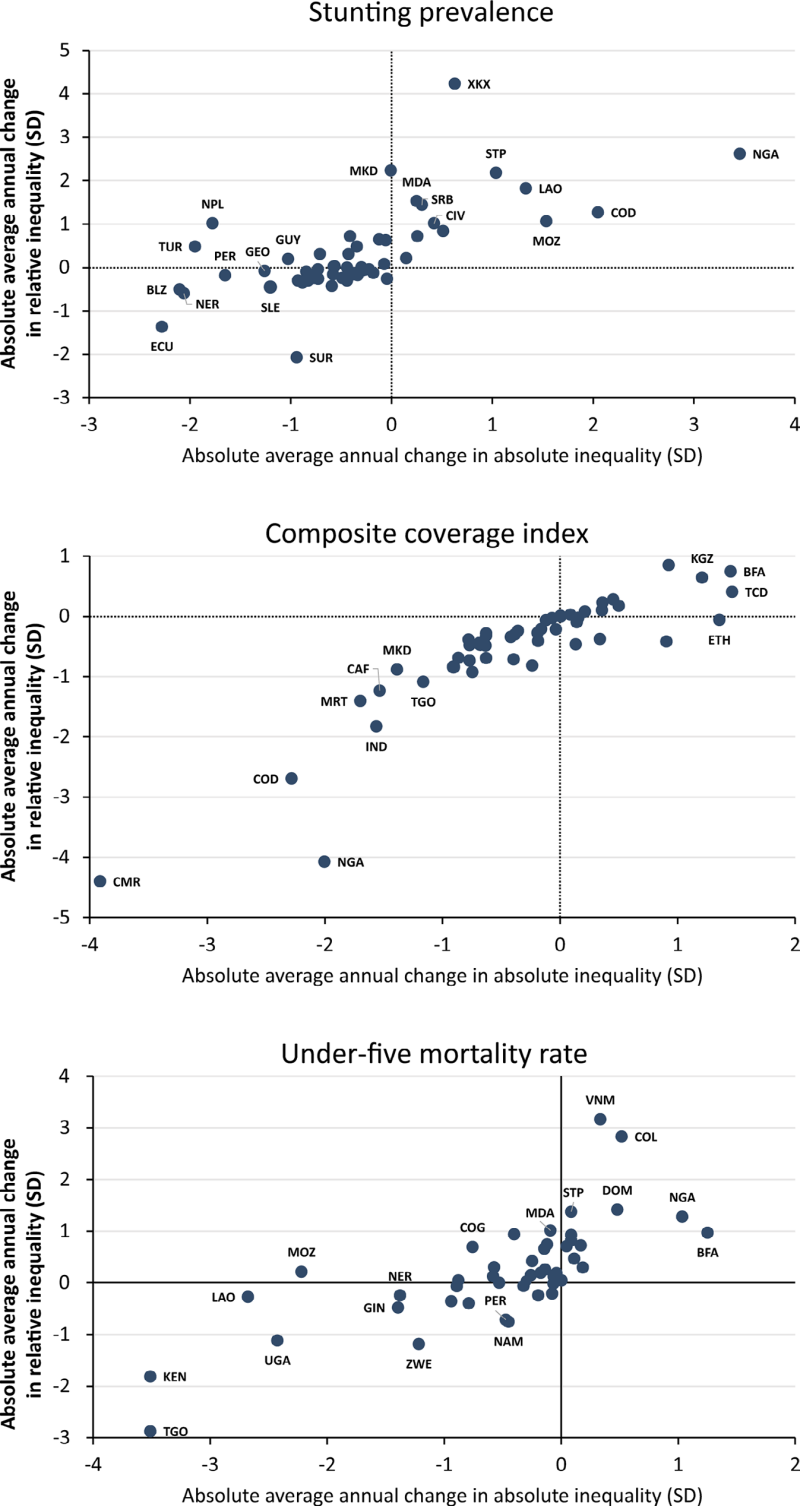
Average annual absolute changes in relative and absolute inequality in stunting prevalence, the composite coverage index and under-five mortality rate Legend: Each dot is one country.

We further explored between-country variability by plotting annual changes in absolute inequality against changes in relative inequality (Fig. 2). The patterns for the three outcomes confirm the moderate to strong correlations among changes in absolute and relative inequality. For stunting, there were reductions in absolute inequality in most countries, in contrast to increases in relative inequality in about half of all countries. For the CCI, there were reductions in absolute and relative inequality in most countries. For U5MR, there were reductions in absolute inequality in most countries accompanied by increases in relative inequality, also in most countries.

Up to this point, we have shown that different summary metrics tend to be moderately to highly correlated within each outcome. The next step was to correlate the annual absolute changes in WMDOM and WMROM across the three outcomes, again using countries as the units of analysis. Table 3 shows that correlations between changes in WMDOM and in WMROM for the same outcome were moderate to strong: 0.658 for stunting, 0.879 for CCI and 0.705 for U5MR. However, a different picture emerged when comparing progress across the three outcomes. Changes over time for stunting tended to be inversely correlated with changes in CCI, and changes in U5MR were weakly correlated with changes in stunting and uncorrelated to changes in the CCI. An extreme example of dissociation in trends was Nigeria, which was one of the worst-performing countries for ethnic inequalities in stunting and U5MR, and one of the best performers for the CCI (Fig. 2).

**Table 3:** Pearson correlation coefficients between absolute annual changes summary statistics for stunting, CCI and U5MR.

		WMDOM (absolute inequality)			WMROM (relative inequality)		
		Stunting	CCI	U5MR	Stunting	CCI	U5MR
**WMDOM**	**Stunting**	1.000					
	**CCI**	**-0.459**	1.000				
	**U5MR**	0.046	0.156	1.000			
**WMROM**	**Stunting**	**0.658**	**-0.398**	0.084	1.000		
	**CCI**	**-0.579**	**0.879**	0.086	**-0.471**	1.000	
	**U5MR**	0.237	0.085	**0.705**	0.281	0.003	1.000

Legend: Countries are the units of analysis. Values in bold indicate p value < 0.05. WMDOM - weighted mean difference from overall mean (absolute). WMROM - weighted mean ratio to overall mean (relative). CCI – composite coverage index. U5MR – under-five mortality rate.

**Fig. 3:**
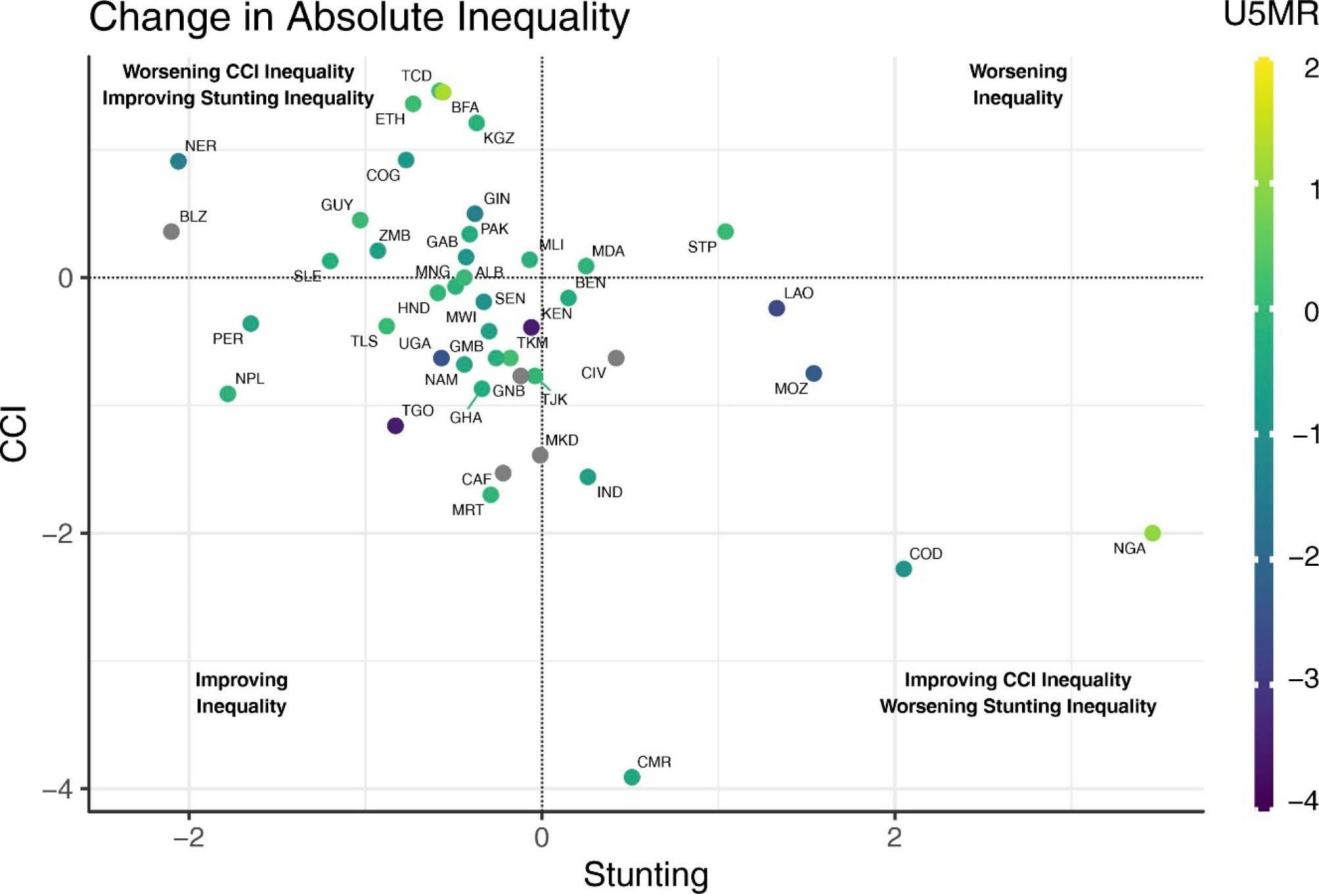
Absolute annual changes in ethnic inequalities stunting and CCI. Legend: CCI – composite coverage index. Dots are colored according to changes in ethnic under-five mortality rate (U5M) inequalities.

The inverse correlation of -0.459 between absolute annual changes in absolute inequality in stunting prevalence and in CCI is shown in Fig. 3. Each country is displayed as a colored dot, with yellow indicating annual increases in ethnic U5MR inequalities, green dots signaling stability and purple dots showing reduced inequalities. For example, Togo and Uganda are in the bottom left quadrant (improved inequality for stunting and CCI) and are colored in purple indicating reduction in U5MR inequality.

Lastly, we ranked the 39 countries with information on all three outcomes (Table 4). An arbitrary score of + 1 was given to annual reductions of 0.5 SD or higher, a score of zero for changes between − 0.5 and + 0.5 SD and of -1 for increases of 0.5 SD or higher. Uganda and Togo showed reductions in absolute inequality for the three outcomes, and in relative inequality for two outcomes (CCI and U5MR, respectively), and as a result were well ahead of the remaining 37 countries. São Tomé and Principe as well as Burkina Faso were the worst performers, with scores of -3, indicating that ethnic inequality tended to increase over time.

**Table 4:** Scoring system for assessing changes over time in ethnic inequalities in the three outcome indicators

	Change in absolute inequality			Change in relative inequality			
Country	Stunting	CCI	U5MR	Stunting	CCI	U5MR	Score
Uganda	+ 1	+ 1	+ 1	0	+ 1	+ 1	**5**
Togo	+ 1	+ 1	+ 1	0	+ 1	+ 1	**5**
Zambia	+ 1	0	+ 1	0	0	0	**2**
Peru	+ 1	0	0	0	0	+ 1	**2**
Niger	+ 1	-1	+ 1	+ 1	0	0	**2**
Namibia	0	+ 1	0	0	0	+ 1	**2**
Mauritania	0	+ 1	0	0	+ 1	0	**2**
Kenya	0	0	+ 1	-1	+ 1	+ 1	**2**
India	0	+ 1	+ 1	-1	+ 1	0	**2**
Ghana	0	+ 1	0	0	+ 1	0	**2**
Turkmenistan	0	+ 1	0	0	0	0	**1**
Tajikistan	0	+ 1	0	0	0	0	**1**
Sierra Leone	+ 1	0	0	0	0	0	**1**
Senegal	0	0	+ 1	0	0	0	**1**
Nepal	+ 1	+ 1	0	-1	+ 1	-1	**1**
Mozambique	-1	+ 1	+ 1	-1	+ 1	0	**1**
Malawi	0	0	+ 1	0	0	0	**1**
Honduras	+ 1	0	0	0	0	0	**1**
Guinea	0	0	+ 1	0	0	0	**1**
Gambia	0	+ 1	0	0	0	0	**1**
Gabon	0	0	+ 1	0	0	0	**1**
Congo DR	-1	+ 1	+ 1	-1	+ 1	0	**1**
Timor-Leste	+ 1	0	0	0	0	-1	**0**
Mali	0	0	0	0	0	0	**0**
Lao PDR	-1	0	+ 1	-1	+ 1	0	**0**
Guyana	+ 1	0	0	0	0	-1	**0**
Ethiopia	+ 1	-1	0	0	0	0	**0**
Benin	0	0	0	0	0	0	**0**
Pakistan	0	0	0	-1	0	0	**-1**
Mongolia	0	0	0	0	0	-1	**-1**
Congo Republic	+ 1	-1	+ 1	0	-1	-1	**-1**
Chad	+ 1	-1	0	0	0	-1	**-1**
Cameroon	-1	+ 1	0	-1	+ 1	-1	**-1**
Albania	0	0	0	0	0	-1	**-1**
Nigeria	-1	+ 1	-1	-1	+ 1	-1	**-2**
Moldova	0	0	0	-1	0	-1	**-2**
Kyrgyzstan	0	-1	0	0	-1	0	**-2**
Sao Tome and Principe	-1	0	0	-1	0	-1	**-3**
Burkina Faso	+ 1	-1	-1	0	-1	-1	**-3**

Legend: CCI – composite coverage index. U5MR – under-five mortality rate.

## Discussion

Assessing time trends in health inequalities is a complex matter, as summarized by Harper and Lynch:

*“The choice of a summary measure of disparity may affect the interpretation of changes in health disparities. Important issues to consider are the reference point from which differences are measured, whether to measure disparity on the absolute or relative scale, and whether to weight disparity measures by population size. A suite of indicators is needed to provide a clear picture of health disparity change.”*^[[20]]^.

Additional challenges arise when studying unordered categorical variables such as ethnicity or subnational regions [19] as – unlike stratification variables such as wealth quintiles or urban-rural residence – the number of categories being compared vary from country to country.

Our first objective was to assess how results might have been affected by the type of inequality measure (absolute or relative), by whether ethnic groups should be weighted by population size, and also whether the summary measure should compare the outcomes in all ethnic groups or only between the worst- and best-performing groups. Given such methodological difficulties, it was reassuring that the 12 combinations of six summary measures with two approaches (absolute and relative) for expressing time trends led to highly comparable results within each outcome variable, allowing us to simplify the actual comparison of countries and the identification of exemplars.

In our analyses we addressed combinations of several measurement alternatives: absolute versus relative inequality; whole-distribution summary measures versus extreme group comparisons; population weighted versus unweighted measures; and examination of absolute versus relative time trends. We will discuss what we learned from these analyses and from the literature.

There are many examples of on how trends in absolute and relative inequality may appear to be inconsistent. [20, 29] It is not uncommon in inequality trend analysis to find that relative inequality decreased while absolute inequality increased, or vice-versa. This is the result of the mathematics of absolute and relative indicators. With an intervention coverage indicator such as the CCI increasing rapidly over time, on average, the better-off group may move close to universal coverage with limited room for further improvement, and both absolute and relative inequality measures will decrease. For detrimental outcomes such as stunting and U5MR, the opposite will tend to happen when the better-group reaches level below which further progress in unlikely, and absolute inequality will fall, followed by relative inequality. [29] Taking a hypothetical example in which stunting prevalence fell from 6 to 3% over time in the best-performing ethnicity (a drop of 50% but only of 3% points), compared to from 30 to 20% in the worst-off group (a drop of 33% and of 10% points), the summary measures will show and increase in relative inequality as the best-performing group had a greater relative decline (50% versus 33%) accompanied by a decrease in absolute inequality for the worst-performing groups (10 versus 3% points). Therefore, we feel it is important to examine both absolute and relative inequality measures.

Secondly, we compared weighted and unweighted measures. The latter present the potential disadvantage of being influenced by large changes small ethnic groups. Results of our analyses were very similar for weighted and unweighted measures, and given the conceptual advantages of the former, we recommend their use.

Thirdly, extreme group comparisons were less strongly associated with changes in summary measures that include the whole sample in their calculation. This is likely due to the fact that one or both these groups may be small, leading to imprecision. There is also the limiting issue that the best- and worst-performing groups may change over time. Although comparisons of extreme groups may be easy to understand and useful for advocacy, we do not recommend them as the basis of time trend analyses.

Lastly, we compared relative changes over time with absolute changes. As shown in Table 1, absolute and relative annual changes in the same summary measure were highly correlated among themselves, with only one of the 18 correlation coefficients tested being below 0.8: the correlation between absolute and relative annual changes in WMROM for the CCI.

Based on the above analyses, we feel that the study of absolute annual changes in weighted summary indices for both relative and absolute inequalities is appropriate for assessing reductions in ethnic group inequalities at national level, which was the primary purpose of our study. Analyses of overall trends, however, do not replace examination of progress for each separate ethnic group, whether large or small. In particular, changes in the less populous groups may not be picked up by overall summary measures. Additional files 4 to 6 show country-by-country changes over time for the three outcome measures. In addition, groups with small numbers of children in the survey sample had to be merged in a category of “other ethnic groups”, which will often include diverse ethnicities with different rates of progress.

Our analyses also had other limitations. Language was used as proxy for ethnic group affiliation in 16 of the 59 countries, where the surveys did not collect information on affiliation; nevertheless, ethnicity and language are closely linked in LMICs. [23, 24] Only 34 of the 59 countries studied had full results for the three outcomes. The time elapsed between the baseline and endline surveys ranged from 3 to 20 years with a median of 11 years; longer periods should allow changes to be measured more accurately, but we did not find an association between the time interval and annual changes. Nor did we find noteworthy associations of the magnitude of changes with national baseline coverage, the number of ethnic groups or the number of children per group.

Whereas our results were robust in showing that different metrics tend to provide similar results for time trends in inequalities within each outcome, comparisons in progress across the three outcomes showed consistent results. One possible explanation is that such comparisons may be affected by measurement periods. Stunting is calculated for children aged 0–59 months, CCI for children aged 12–59 months, and U5MR for all births in the 10 years preceding the survey accrue enough deaths and improve precision. Although stunting is measured at the time of the survey, it is a cumulative deficit starting during gestation. The CCI mostly reflects recent interventions (family planning, vaccines, case-management of illnesses) but also includes antenatal and delivery care which are measured retrospectively for up to three years before the survey. Although child mortality and nutritional status share common determinants, [30] mortality is more likely to respond to existing biomedical interventions than stunting. [31, 32] In addition, CCI may be improved rapidly through focused interventions which is not the case for stunting nor for U5MR, particularly because – as mentioned above – the latter refers to children born in the 10 years preceding the survey. Therefore, it is not surprising that countries that performed well in reducing inequalities in a given outcome did not necessarily do so for the other outcomes.

## Conclusions

Our results show that different metrics of changes over time in national-level ethnic inequalities tend to show similar results for a given outcome indicator. However, changes over time in one outcome were not consistently related to time trends for other outcomes. In spite of these caveats, among the 39 countries with full information, Togo and Uganda were well ahead of the rest and may be regarded as exemplar countries. At the other extreme, the worst-performing countries include Nigeria, Moldova, Kyrgyzstan, Sao Tome and Principe, and Burkina Faso. Reasons for success – or lack thereof – should be investigated by in-depth studies at national level.

## Electronic supplementary material

Below is the link to the electronic supplementary material.

Additional file 1. Title: List of countries included and description of the ethnic or language groups in each country. Description: Table describing ethnic or language groups present in each country.

Additional file 2. Title: Available information on ethnicity or language, by country and outcome. Description: Table with surveys included, number of ethnic groups per country, and the mean sample sizes for each outcome.

Additional file 3. Title: Values of the indices in the first and last surveys, per country and indicator: stunting, U5MR and CCI. Description: Table with values of the summary indices for the first and last surveys as well as for annual changes per country.

Additional file 4. Title: Stunting prevalence by ethnic groups in the first and last surveys. Results for selected countries. The numbers in the black rectangles show the average proportion of the samples for each ethnic group in the two surveys. The numbers at the bottom of the bars show the ratio between the rate in a particular ethnic group and the national rate for that point in time. Description: One graph for each country showing Stunting prevalence by ethnic groups in the first and last surveys.

Additional file 5. Title: Composite coverage index by ethnic groups in the first and last surveys. Results for selected countries. The numbers in the black rectangles show the average proportion of the samples for each ethnic group in the two surveys. The numbers at the bottom of the bars show the ratio between the rate in a particular ethnic group and the national rate for that point in time. Description: One graph for each country showing the composite coverage index (CCI) by ethnic groups in the first and last surveys.

Additional file 6. Title: Under-five mortality rate by ethnic groups in the first and last surveys. Results for selected countries. The numbers in the black rectangles show the average proportion of the samples for each ethnic group in the two surveys. The numbers at the bottom of the bars show the ratio between the rate in a particular ethnic group and the national rate for that point in time. Description: One graph for each country showing under-five mortality rate (U5MR) by ethnic groups in the first and last surveys.

## Data Availability

The datasets used and/or analyzed during the current study are available from the corresponding author on reasonable request.
