# Supplementary material for: Time trends in ethnic inequalities in child health and nutrition: analysis of 59 low and middle-income countries
Source: Int J Equity Health. 2023 Apr 28;22:76. doi: 10.1186/s12939-023-01888-5 (PMC10148503; doi:10.1186/s12939-023-01888-5)
Supplement: Supplementary file 1 — Additional file 1. Title: List of countries included and description of the ethnic or language groups in each country. Description: Table describing ethnic or language groups present in each country. [file 12939_2023_1888_MOESM1_ESM.docx]

| Country | Ethnicity or language | Obs. |
| --- | --- | --- |
| Albania | Albanian |  |
|  | Other |  |
| Belize | Creole |  |
|  | Garifuna | only stunting analysis, insuff. sample for CCI |
|  | Maya |  |
|  | Mestizo / Spanish / Latino |  |
|  | Other |  |
| Benin | Adja et apparentés |  |
|  | Bariba et apparentés |  |
|  | Betamaribe et apparentés |  |
|  | Dendi et apparentés |  |
|  | Fon et apparentés |  |
|  | Peulh et apparentés |  |
|  | Yoruba et apparentés |  |
|  | Autre |  |
| Burkina Faso | Bissa |  |
|  | Fulfuldé / Peul |  |
|  | Gourmantché |  |
|  | Gourounsi |  |
|  | Mossi |  |
|  | Sénoufo |  |
|  | Autres |  |
| Cameroon | Adamaoua-Oubangui |  |
|  | Arabe _ Choa / Peulh / Haoussa / Kanouri |  |
|  | Bamiléké / Bamoun |  |
|  | Beti / Bassa / Mbam |  |
|  | Biu-Mandara |  |
|  | Grassfields / Nord-Ouest |  |
|  | Kako / Maka / Pygmée |  |
|  | Autre groupe ethnique |  |
| Central African Republic | Banda |  |
|  | Gbaya |  |
|  | Haoussa |  |
|  | Mandja |  |
|  | Mboum |  |
|  | Ngbaka / Bantou |  |
|  | Sara |  |
|  | Yakoma / Sango |  |
|  | Autre ethnie |  |
| Chad | Arabe |  |
|  | Baguirmi / Barma |  |
|  | Gorane |  |
|  | Kanembou / Bornou |  |
|  | Ouaddai / Mimi |  |
|  | Peul / Foulbé |  |
|  | Sara |  |
|  | Autres éthnies |  |
| Colombia | Indígena |  |
|  | Negro(a) / Mulato(a) / Afrocolombiano / Afrodescendiente |  |
|  | Raizal del Archipélago |  |
|  | Otra |  |
| Congo DR | Bakongo Nord & Sud |  |
|  | Bas-Kasaï et Kwilu-Kwango |  |
|  | Basele-K, Man. et Kivu |  |
|  | Cuvette central |  |
|  | Kasai, Katanga, Tanganyika |  |
|  | Ubangi et Itimbiri |  |
|  | Uele Lac Albert |  |
|  | Autres |  |
| Congo Republic | Kongo |  |
|  | Mbétis |  |
|  | Mbosi |  |
|  | Tékés |  |
|  | Autre groupe ethnique |  |
|  | Etrangers (non congolais) |  |
| Costa Rica | Indígena |  |
|  | Negro o afrodescendiente |  |
|  | Otra |  |
| Côte d'Ivoire | Akan |  |
|  | Gur |  |
|  | Mandé du Nord |  |
|  | Mandé du Sud |  |
|  | Autre |  |
| Dominican Republic | Español |  |
|  | Otro idioma |  |
| Ecuador | Indígena |  |
|  | Mestizo |  |
|  | Otro |  |
| Ethiopia | Amarigna |  |
|  | Oromigna |  |
|  | Tigrigna |  |
|  | Other |  |
| Gabon | Fang |  |
|  | Kota-Kele |  |
|  | Mbede-Teke |  |
|  | Myene |  |
|  | Nzabi-Duma |  |
|  | Okande-Tsogho |  |
|  | Shira-Punu / Vili |  |
|  | Autre |  |
| Gambia | Fula / Tukulur / Lorobo |  |
|  | Jola / Karoninka |  |
|  | Mandinka / Jahanka |  |
|  | Wollof |  |
|  | Other |  |
| Georgia | Armenian |  |
|  | Azerbaijani |  |
|  | Georgian |  |
|  | Other |  |
| Ghana | Akan |  |
|  | Ewe |  |
|  | Ga / Damgme |  |
|  | Grusi |  |
|  | Guan |  |
|  | Mole Dagbani |  |
|  | Others |  |
| Guatemala | Ladina / Mestiza |  |
|  | Indígena |  |
| Guinea | Guerzé |  |
|  | Malinké |  |
|  | Peulh |  |
|  | Soussou |  |
|  | Autre / étranger |  |
| Guinea-Bissau | Balanta |  |
|  | Fula |  |
|  | Mancanha |  |
|  | Mandinga |  |
|  | Manjaco |  |
|  | Papel |  |
|  | Outra etnia |  |
| Guyana | African / Black |  |
|  | Amerindian |  |
|  | East Indian |  |
|  | Mixed Race |  |
| Honduras | Garífuna |  |
|  | Lenca |  |
|  | Maya Chortí |  |
|  | Misquito |  |
|  | Otros Pueblos |  |
| India | Assamese |  |
|  | Bengali |  |
|  | English | omitted in the graphs (< 1% of the total sample) |
|  | Gujarati |  |
|  | Hindi |  |
|  | Kannada |  |
|  | Kashmiri | only U5MR analysis |
|  | Malayalam |  |
|  | Manipuri | omitted in the graphs (< 1% of the total sample) |
|  | Marathi |  |
|  | Nepali | omitted in the graphs (< 1% of the total sample) |
|  | Oriya |  |
|  | Punjabi |  |
|  | Tamil |  |
|  | Telugu |  |
|  | Urdu | insuff. sample for U5MR analysis |
|  | Other |  |
| Kazakhstan | Kazakh |  |
|  | Russian |  |
|  | Other ethnic groups |  |
| Kenya | Embu | only CCI analysis, insuff. sample for stunting & U5MR |
|  | Kalenjin |  |
|  | Kamba |  |
|  | Kikuyu |  |
|  | Kisii |  |
|  | Luhya |  |
|  | Luo |  |
|  | Meru |  |
|  | Mijikenda | joint with Swahili for stunting analysis and insuff. sample for U5MR |
|  | Somali |  |
|  | Swahili | joint with Mijikenda for stunting analysis |
|  | Taita / Taveta | only U5MR analysis |
|  | Other |  |
| Kosovo | Albanian |  |
|  | Other ethnic groups |  |
| Kyrgyzstan | Kyrgyz |  |
|  | Russian |  |
|  | Other language |  |
| Lao PDR | Hmong-Mien |  |
|  | Lao-Tai |  |
|  | Other |  |
| Malawi | Chewa |  |
|  | Lomwe |  |
|  | Ngoni |  |
|  | Nkhonde |  |
|  | Sena |  |
|  | Tonga |  |
|  | Tumbuka |  |
|  | Yao |  |
|  | Other ethnicity |  |
| Mali | Bambara |  |
|  | Bobo |  |
|  | Dogon |  |
|  | Peulh |  |
|  | Sarakolé / Soninké / Marka |  |
|  | Sénoufo / Minianka |  |
|  | Autres |  |
| Mauritania | Arabe |  |
|  | Poular |  |
|  | Soninké |  |
|  | Wolof |  |
|  | Autre langue | insuff. sample for U5MR analysis |
| Moldova | Moldovan / Romanian |  |
|  | Russian |  |
|  | Ukrainian |  |
|  | Other ethnic group |  |
| Mongolia | Kazakh |  |
|  | Khalkh |  |
|  | Other |  |
| Mozambique | Cindau |  |
|  | Cinyungwe |  |
|  | Cisena |  |
|  | Echuwabo | insuff. sample for U5MR analysis |
|  | Elomwe |  |
|  | Emakhuwa |  |
|  | Português |  |
|  | Xichangana |  |
|  | Xitswa |  |
|  | Outras |  |
| Namibia | Afrikaans |  |
|  | Damara / Nama |  |
|  | Oshiwambo |  |
|  | Otjiherero |  |
|  | Rukwangali |  |
|  | Other |  |
| Nepal | Hill Brahmin |  |
|  | Hill Chhetri |  |
|  | Hill Dalit |  |
|  | Hill Janajati |  |
|  | Muslim |  |
|  | Newar |  |
|  | Terai Dalit |  |
|  | Terai Janajati |  |
|  | Other Terai caste |  |
|  | Other |  |
| Niger | Fulfuldé |  |
|  | Haoussa |  |
|  | Tamasheq |  |
|  | Zarma |  |
|  | Autres |  |
| Nigeria | Fulani |  |
|  | Hausa |  |
|  | Ibibio |  |
|  | Igbo |  |
|  | Yoruba |  |
|  | Other |  |
| North Macedonia | Albanian |  |
|  | Macedonian |  |
|  | Other |  |
| Pakistan | Baluchi |  |
|  | Punjabi |  |
|  | Pushto |  |
|  | Saraiki |  |
|  | Sindhi |  |
|  | Urdu |  |
|  | Other |  |
| Peru | Castellano |  |
|  | Quechua |  |
|  | Otra lengua nativa u originaria |  |
| Philippines | Bikolano |  |
|  | Cebuano |  |
|  | Ilocano |  |
|  | Ilonggo |  |
|  | Kapampangan |  |
|  | Maranao |  |
|  | Tagalog |  |
|  | Tausug |  |
|  | Waray |  |
|  | Other |  |
| Sao Tome and Principe | Português |  |
|  | Crioulo Forro |  |
| Senegal | Diola |  |
|  | Mandingue / Socé |  |
|  | Poular |  |
|  | Serer |  |
|  | Wolof |  |
|  | Autre / non Sénégalais |  |
| Serbia | Bosnian |  |
|  | Roma |  |
|  | Serbian |  |
|  | Other |  |
| Sierra Leone | Kono |  |
|  | Limba |  |
|  | Loko |  |
|  | Mandingo |  |
|  | Mende |  |
|  | Temne |  |
|  | Other |  |
| Suriname | Creole |  |
|  | Hindustani |  |
|  | Indigenous / Amerindian |  |
|  | Javanese |  |
|  | Maroon |  |
|  | Other |  |
| Tajikistan | Russian | insuff. sample for U5MR analysis |
|  | Tajik |  |
|  | Other |  |
| Thailand | Thai |  |
|  | Non-Thai |  |
| Timor-Leste | Tetum |  |
|  | Other |  |
| Togo | Adja / Ewe |  |
|  | Akposso / Akébou |  |
|  | Ife / Ana | only stunting analysis, insuff. sample for CCI |
|  | Kabye / Tem |  |
|  | Para / Gourma |  |
|  | Autres togolais |  |
|  | Autres nationalités |  |
| Türkiye | Turkish |  |
|  | Other |  |
| Turkmenistan | Russian | only stunting analysis, insuff. sample for CCI |
|  | Turkmen |  |
|  | Uzbek |  |
|  | Other language | insuff. sample for U5MR analysis |
| Uganda | Ateso |  |
|  | Luganda |  |
|  | Lugbara |  |
|  | Luo |  |
|  | Runyankole / Rukiga |  |
|  | Runyoro / Rutoro |  |
|  | Other |  |
| Vietnam | Kinh / Hoa |  |
|  | Ethnic Minorities |  |
| Zambia | Bemba |  |
|  | Kaonde |  |
|  | Lozi |  |
|  | Lunda |  |
|  | Luvale |  |
|  | Nyanja |  |
|  | Tonga |  |
|  | Other |  |
| Zimbabwe | Ndebele |  |
|  | Shona |  |
|  | Other language |  |
