# Supplementary material for: Time trends in ethnic inequalities in child health and nutrition: analysis of 59 low and middle-income countries
Source: Int J Equity Health. 2023 Apr 28;22:76. doi: 10.1186/s12939-023-01888-5 (PMC10148503; doi:10.1186/s12939-023-01888-5)
Supplement: Supplementary file 2 — Additional file 2. Title: Available information on ethnicity or language, by country and outcome. Description: Table with surveys included, number of ethnic groups per country, and the mean sample sizes for each outcome. [file 12939_2023_1888_MOESM2_ESM.docx]

|  |  |  |  | **Weighted summary** | | **Unweighted summary** | |  |  |  |  |
| --- | --- | --- | --- | --- | --- | --- | --- | --- | --- | --- | --- |
|  |  |  | **National stunting prevalence** | **Absolute** | **Relative** | **Absolute** | **Relative** | **Extreme group comparisons** | | **Prevalence (%) in worst and best groups** | |
| **Country** | **Year** | **Nº of groups** | **%** | **WMDOM** | **WMROM** | **UMDOM** | **UMROM** | **Hi/lo diff** | **Hi/lo ratio** | **Worst** | **Best** |
| Albania | 2008 | 2 | 23.3 | 1.0 | 4.4 | 8.7 | 37.3 | 17.4 | 1.8 | 40.1 | 22.7 |
|  | 2017 | 2 | 11.3 | 0.3 | 2.4 | 3.0 | 26.4 | 5.9 | 1.5 | 17.3 | 11.3 |
|  | Absolute annual change (p.p.) | | -1.33 | -0.08 | -0.23 | -0.63 | -1.21 | -1.27 | -0.03 | -2.53 | -1.26 |
|  | Relative annual change (%) | | -7.7 | -13.9 | -6.7 | -11.2 | -3.8 | -11.2 | -1.6 | -8.9 | -7.4 |
| Belize | 2006 | 5 | 22.5 | 8.6 | 38.3 | 11.3 | 50.2 | 46.2 | 32.2 | 47.6 | 1.5 |
|  | 2015 | 5 | 14.9 | 5.2 | 34.6 | 7.6 | 51.1 | 26.5 | 4.2 | 34.7 | 8.2 |
|  | Absolute annual change (p.p.) | | -0.844 | -0.384 | -0.410 | -0.408 | 0.101 | -2.184 | -3.112 | -1.433 | 0.751 |
|  | Relative annual change (%) | | -4.46 | -5.54 | -1.12 | -4.27 | 0.20 | -5.98 | -20.22 | -3.45 | 21.03 |
| Benin | 2001 | 8 | 39.0 | 3.9 | 10.1 | 4.9 | 12.6 | 14.4 | 1.4 | 48.1 | 33.7 |
|  | 2017 | 8 | 33.4 | 4.4 | 13.2 | 5.0 | 15.0 | 20.7 | 1.8 | 47.6 | 26.9 |
|  | Absolute annual change (p.p.) | | -0.35 | 0.03 | 0.19 | 0.01 | 0.15 | 0.40 | 0.02 | -0.03 | -0.43 |
|  | Relative annual change (%) | | -1.0 | 0.7 | 1.6 | 0.1 | 1.1 | 2.3 | 1.4 | -0.1 | -1.4 |
| Burkina Faso | 2003 | 7 | 43.1 | 4.6 | 10.7 | 7.2 | 16.6 | 28.7 | 1.8 | 62.5 | 33.8 |
|  | 2010 | 7 | 34.9 | 3.0 | 8.6 | 4.9 | 14.1 | 17.6 | 1.7 | 44.1 | 26.6 |
|  | Absolute annual change (p.p.) | | -1.182 | -0.229 | -0.294 | -0.326 | -0.370 | -1.589 | -0.027 | -2.622 | -1.032 |
|  | Relative annual change (%) | | -3.00 | -5.92 | -3.01 | -5.31 | -2.39 | -6.76 | -1.52 | -4.85 | -3.38 |
| Cameroon | 2006 | 8 | 36.4 | 6.1 | 16.7 | 5.2 | 14.4 | 20.6 | 1.8 | 47.5 | 26.9 |
|  | 2014 | 8 | 31.8 | 6.7 | 21.2 | 5.6 | 17.8 | 20.4 | 2.0 | 40.9 | 20.5 |
|  | Absolute annual change (p.p.) | | -0.58 | 0.08 | 0.56 | 0.05 | 0.42 | -0.02 | 0.03 | -0.82 | -0.80 |
|  | Relative annual change (%) | | -1.7 | 1.3 | 3.0 | 0.9 | 2.7 | -0.1 | 1.5 | -1.8 | -3.3 |
| Central African Republic | 2006 | 9 | 44.2 | 3.4 | 7.8 | 4.4 | 9.9 | 17.8 | 1.5 | 52.6 | 34.8 |
|  | 2018 | 9 | 39.8 | 3.2 | 8.1 | 3.2 | 8.1 | 12.5 | 1.4 | 46.5 | 34.1 |
|  | Absolute annual change (p.p.) | | -0.368 | -0.018 | 0.026 | -0.096 | -0.151 | -0.446 | -0.012 | -0.507 | -0.061 |
|  | Relative annual change (%) | | -0.87 | -0.55 | 0.33 | -2.53 | -1.67 | -2.93 | -0.84 | -1.02 | -0.18 |
| Chad | 2004 | 8 | 44.8 | 5.4 | 12.0 | 7.7 | 17.2 | 21.8 | 1.6 | 57.1 | 35.3 |
|  | 2019 | 8 | 37.8 | 3.6 | 9.6 | 4.4 | 11.7 | 19.2 | 1.7 | 48.6 | 29.4 |
|  | Absolute annual change (p.p.) | | -0.47 | -0.12 | -0.16 | -0.22 | -0.37 | -0.17 | 0.00 | -0.56 | -0.39 |
|  | Relative annual change (%) | | -1.1 | -2.6 | -1.5 | -3.6 | -2.5 | -0.8 | 0.1 | -1.1 | -1.2 |
| Congo DR | 2007 | 8 | 45.8 | 2.2 | 4.9 | 4.6 | 10.0 | 18.1 | 1.6 | 48.5 | 30.4 |
|  | 2013 | 8 | 42.7 | 4.6 | 10.8 | 4.4 | 10.4 | 16.3 | 1.5 | 49.8 | 33.5 |
|  | Absolute annual change (p.p.) | | -0.52 | 0.39 | 0.98 | -0.03 | 0.06 | -0.30 | -0.02 | 0.21 | 0.51 |
|  | Relative annual change (%) | | -1.2 | 12.8 | 14.1 | -0.6 | 0.6 | -1.7 | -1.2 | 0.4 | 1.6 |
| Congo Republic | 2011 | 6 | 24.5 | 2.5 | 10.2 | 3.6 | 14.6 | 12.5 | 1.8 | 28.8 | 16.2 |
|  | 2014 | 6 | 21.3 | 2.1 | 9.7 | 2.6 | 12.1 | 6.7 | 1.3 | 26.3 | 19.6 |
|  | Absolute annual change (p.p.) | | -1.06 | -0.15 | -0.19 | -0.33 | -0.84 | -1.95 | -0.14 | -0.83 | 1.12 |
|  | Relative annual change (%) | | -4.5 | -6.3 | -1.8 | -10.4 | -6.1 | -18.9 | -8.9 | -3.0 | 6.5 |
| Côte d'Ivoire | 2006 | 5 | 40.1 | 2.0 | 4.9 | 2.4 | 6.0 | 8.1 | 1.2 | 44.3 | 36.2 |
|  | 2016 | 5 | 21.8 | 2.8 | 12.7 | 3.8 | 17.5 | 12.3 | 1.7 | 29.7 | 17.5 |
|  | Absolute annual change (p.p.) | | -1.83 | 0.08 | 0.78 | 0.14 | 1.15 | 0.41 | 0.05 | -1.46 | -1.87 |
|  | Relative annual change (%) | | -5.9 | 3.5 | 10.0 | 4.7 | 11.3 | 4.2 | 3.3 | -3.9 | -7.0 |
| Ecuador | 2004 | 3 | 32.6 | 6.0 | 18.4 | 13.0 | 39.9 | 36.9 | 2.7 | 58.3 | 21.4 |
|  | 2012 | 3 | 25.1 | 2.5 | 10.0 | 6.8 | 27.1 | 19.7 | 2.0 | 39.5 | 19.9 |
|  | Absolute annual change (p.p.) | | -0.93 | -0.44 | -1.05 | -0.77 | -1.60 | -2.16 | -0.09 | -2.35 | -0.19 |
|  | Relative annual change (%) | | -3.2 | -10.3 | -7.3 | -7.8 | -4.7 | -7.6 | -3.9 | -4.7 | -0.9 |
| Ethiopia | 2000 | 4 | 57.5 | 2.5 | 4.3 | 2.6 | 4.6 | 7.5 | 1.1 | 61.5 | 54.0 |
|  | 2019 | 4 | 36.9 | 1.5 | 4.1 | 3.5 | 9.4 | 12.9 | 1.4 | 48.0 | 35.1 |
|  | Absolute annual change (p.p.) | | -1.09 | -0.05 | -0.01 | 0.04 | 0.25 | 0.28 | 0.01 | -0.71 | -0.99 |
|  | Relative annual change (%) | | -2.3 | -2.5 | -0.2 | 1.5 | 3.9 | 2.9 | 1.0 | -1.3 | -2.2 |
| Gabon | 2000 | 8 | 26.3 | 4.2 | 16.0 | 4.8 | 18.4 | 18.1 | 1.9 | 38.3 | 20.2 |
|  | 2012 | 8 | 17.0 | 3.2 | 18.9 | 4.9 | 28.8 | 16.9 | 2.6 | 27.5 | 10.6 |
|  | Absolute annual change (p.p.) | | -0.77 | -0.08 | 0.24 | 0.01 | 0.87 | -0.10 | 0.06 | -0.90 | -0.80 |
|  | Relative annual change (%) | | -3.5 | -2.2 | 1.4 | 0.1 | 3.8 | -0.6 | 2.6 | -2.7 | -5.2 |
| Gambia | 2005 | 5 | 27.7 | 1.6 | 5.7 | 1.7 | 6.1 | 4.2 | 1.2 | 29.1 | 24.9 |
|  | 2019 | 5 | 17.5 | 1.4 | 8.0 | 1.8 | 10.3 | 6.7 | 1.4 | 22.0 | 15.3 |
|  | Absolute annual change (p.p.) | | -0.73 | -0.01 | 0.16 | 0.01 | 0.30 | 0.18 | 0.02 | -0.51 | -0.68 |
|  | Relative annual change (%) | | -3.2 | -0.9 | 2.4 | 0.5 | 3.9 | 3.3 | 1.5 | -2.0 | -3.4 |
| Georgia | 2005 | 4 | 14.6 | 4.0 | 27.4 | 8.9 | 60.7 | 27.1 | 5.3 | 33.3 | 6.2 |
|  | 2018 | 4 | 5.8 | 0.9 | 14.9 | 2.9 | 49.6 | 10.0 | 4.6 | 12.8 | 2.8 |
|  | Absolute annual change (p.p.) | | -0.68 | -0.24 | -0.96 | -0.46 | -0.86 | -1.31 | -0.06 | -1.58 | -0.27 |
|  | Relative annual change (%) | | -6.9 | -11.2 | -4.6 | -8.3 | -1.5 | -7.4 | -1.1 | -7.1 | -6.0 |
| Ghana | 2003 | 7 | 35.6 | 3.9 | 10.8 | 4.1 | 11.6 | 15.3 | 1.5 | 43.6 | 28.3 |
|  | 2017 | 7 | 17.6 | 2.1 | 12.2 | 3.5 | 20.2 | 14.4 | 2.0 | 28.3 | 13.9 |
|  | Absolute annual change (p.p.) | | -1.29 | -0.12 | 0.09 | -0.04 | 0.61 | -0.06 | 0.04 | -1.09 | -1.03 |
|  | Relative annual change (%) | | -4.9 | -4.1 | 0.8 | -1.1 | 4.0 | -0.4 | 2.0 | -3.0 | -5.0 |
| Guatemala | 2002 | 2 | 54.7 | 16.4 | 29.9 | 17.0 | 31.0 | 34.0 | 1.8 | 75.0 | 41.0 |
|  | 2014 | 2 | 46.7 | 13.6 | 29.1 | 13.7 | 29.3 | 27.4 | 1.8 | 61.4 | 34.0 |
|  | Absolute annual change (p.p.) | | -0.67 | -0.23 | -0.06 | -0.27 | -0.15 | -0.55 | 0.00 | -1.13 | -0.58 |
|  | Relative annual change (%) | | -1.3 | -1.5 | -0.2 | -1.8 | -0.5 | -1.8 | -0.1 | -1.7 | -1.5 |
| Guinea | 2005 | 5 | 39.0 | 3.0 | 7.6 | 3.8 | 9.8 | 8.9 | 1.2 | 45.0 | 36.1 |
|  | 2018 | 5 | 30.2 | 2.1 | 7.0 | 3.0 | 9.9 | 9.0 | 1.3 | 37.1 | 28.1 |
|  | Absolute annual change (p.p.) | | -0.68 | -0.07 | -0.04 | -0.06 | 0.01 | 0.01 | 0.01 | -0.61 | -0.61 |
|  | Relative annual change (%) | | -2.0 | -2.5 | -0.6 | -1.8 | 0.1 | 0.1 | 0.4 | -1.5 | -1.9 |
| Guinea-Bissau | 2006 | 7 | 47.7 | 4.6 | 9.6 | 5.4 | 11.4 | 15.7 | 1.4 | 53.8 | 38.2 |
|  | 2018 | 7 | 27.7 | 4.3 | 15.6 | 6.5 | 23.3 | 16.4 | 2.0 | 33.3 | 16.9 |
|  | Absolute annual change (p.p.) | | -1.67 | -0.02 | 0.50 | 0.08 | 0.99 | 0.06 | 0.05 | -1.71 | -1.77 |
|  | Relative annual change (%) | | -4.4 | -0.5 | 4.1 | 1.4 | 6.1 | 0.4 | 2.8 | -3.9 | -6.6 |
| Guyana | 2006 | 4 | 17.9 | 5.0 | 27.9 | 6.1 | 33.8 | 20.4 | 2.8 | 31.8 | 11.4 |
|  | 2019 | 4 | 9.2 | 2.6 | 28.7 | 3.0 | 33.2 | 9.0 | 2.5 | 14.9 | 5.9 |
|  | Absolute annual change (p.p.) | | -0.67 | -0.18 | 0.06 | -0.23 | -0.05 | -0.88 | -0.02 | -1.30 | -0.42 |
|  | Relative annual change (%) | | -5.0 | -4.8 | 0.2 | -5.2 | -0.1 | -6.1 | -0.8 | -5.7 | -4.9 |
| Honduras | 2011 | 5 | 22.7 | 2.4 | 10.6 | 6.0 | 26.6 | 23.1 | 2.4 | 39.5 | 16.4 |
|  | 2019 | 5 | 18.7 | 1.5 | 7.9 | 6.0 | 32.1 | 24.5 | 5.5 | 29.9 | 5.4 |
|  | Absolute annual change (p.p.) | | -0.49 | -0.11 | -0.33 | 0.00 | 0.69 | 0.17 | 0.39 | -1.20 | -1.37 |
|  | Relative annual change (%) | | -2.3 | -5.8 | -3.5 | 0.0 | 2.4 | 0.7 | 10.9 | -3.4 | -12.9 |
| India | 2005 | 17 | 47.7 | 4.8 | 10.0 | 8.2 | 17.1 | 26.8 | 2.1 | 52.1 | 25.3 |
|  | 2015 | 17 | 38.0 | 5.3 | 13.9 | 7.1 | 18.7 | 23.0 | 2.2 | 42.7 | 19.7 |
|  | Absolute annual change (p.p.) | | -0.98 | 0.05 | 0.39 | -0.11 | 0.16 | -0.38 | 0.01 | -0.94 | -0.56 |
|  | Relative annual change (%) | | -2.3 | 1.0 | 3.3 | -1.4 | 0.9 | -1.5 | 0.5 | -2.0 | -2.5 |
| Kazakhstan | 2006 | 3 | 17.5 | 2.6 | 14.9 | 3.2 | 18.4 | 6.4 | 1.5 | 19.4 | 13.0 |
|  | 2015 | 3 | 8.0 | 0.7 | 8.5 | 1.1 | 13.8 | 3.0 | 1.4 | 9.9 | 6.9 |
|  | Absolute annual change (p.p.) | | -1.05 | -0.21 | -0.70 | -0.23 | -0.51 | -0.37 | -0.01 | -1.06 | -0.68 |
|  | Relative annual change (%) | | -8.3 | -13.7 | -6.0 | -11.2 | -3.2 | -8.0 | -0.4 | -7.2 | -6.8 |
| Kenya | 2003 | 12 | 35.8 | 4.0 | 11.3 | 5.6 | 15.7 | 32.7 | 3.0 | 49.0 | 16.3 |
|  | 2014 | 12 | 26.2 | 3.8 | 14.5 | 4.0 | 15.3 | 15.4 | 1.8 | 35.5 | 20.1 |
|  | Absolute annual change (p.p.) | | -0.87 | -0.02 | 0.30 | -0.15 | -0.04 | -1.57 | -0.11 | -1.23 | 0.34 |
|  | Relative annual change (%) | | -2.8 | -0.5 | 2.3 | -3.0 | -0.3 | -6.6 | -4.7 | -2.9 | 1.9 |
| Kosovo | 2013 | 2 | 4.3 | 1.0 | 23.7 | 3.5 | 82.5 | 7.0 | 2.9 | 10.7 | 3.7 |
|  | 2019 | 2 | 4.0 | 1.7 | 43.3 | 3.7 | 92.7 | 7.4 | 3.5 | 10.4 | 3.0 |
|  | Absolute annual change (p.p.) | | -0.04 | 0.12 | 3.27 | 0.03 | 1.70 | 0.06 | 0.10 | -0.05 | -0.12 |
|  | Relative annual change (%) | | -1.0 | 9.4 | 10.6 | 0.9 | 2.0 | 0.9 | 3.1 | -0.5 | -3.5 |
| Kyrgyzstan | 2012 | 3 | 17.9 | 2.2 | 12.2 | 2.3 | 13.0 | 6.9 | 1.4 | 23.4 | 16.5 |
|  | 2018 | 3 | 11.8 | 1.5 | 12.9 | 2.9 | 25.1 | 8.0 | 2.1 | 15.6 | 7.6 |
|  | Absolute annual change (p.p.) | | -1.02 | -0.11 | 0.12 | 0.10 | 2.00 | 0.18 | 0.11 | -1.30 | -1.49 |
|  | Relative annual change (%) | | -6.7 | -5.9 | 0.9 | 4.0 | 11.5 | 2.5 | 6.4 | -6.6 | -12.2 |
| Lao PDR | 2006 | 3 | 47.8 | 8.7 | 18.1 | 8.1 | 17.0 | 18.4 | 1.5 | 57.2 | 38.9 |
|  | 2017 | 3 | 33.1 | 11.1 | 33.7 | 12.5 | 37.9 | 27.1 | 2.2 | 50.3 | 23.2 |
|  | Absolute annual change (p.p.) | | -1.34 | 0.22 | 1.41 | 0.40 | 1.91 | 0.79 | 0.06 | -0.63 | -1.42 |
|  | Relative annual change (%) | | -3.3 | 2.3 | 5.8 | 4.0 | 7.6 | 3.6 | 3.6 | -1.2 | -4.6 |
| Moldova | 2005 | 4 | 10.6 | 0.8 | 7.4 | 1.2 | 11.7 | 3.9 | 1.6 | 11.0 | 7.0 |
|  | 2012 | 4 | 6.4 | 0.8 | 13.0 | 1.7 | 26.7 | 4.4 | 2.4 | 7.5 | 3.1 |
|  | Absolute annual change (p.p.) | | -0.60 | 0.01 | 0.80 | 0.07 | 2.14 | 0.06 | 0.12 | -0.50 | -0.56 |
|  | Relative annual change (%) | | -7.0 | 0.8 | 8.4 | 4.7 | 12.5 | 1.5 | 6.3 | -5.3 | -11.0 |
| Mozambique | 2003 | 10 | 47.1 | 6.4 | 13.5 | 6.2 | 13.3 | 20.5 | 1.6 | 56.2 | 35.7 |
|  | 2011 | 10 | 43.0 | 8.3 | 19.4 | 7.9 | 18.3 | 32.4 | 2.4 | 55.2 | 22.8 |
|  | Absolute annual change (p.p.) | | -0.51 | 0.25 | 0.73 | 0.20 | 0.63 | 1.49 | 0.11 | -0.13 | -1.62 |
|  | Relative annual change (%) | | -1.1 | 3.4 | 4.6 | 2.9 | 4.1 | 5.9 | 5.5 | -0.2 | -5.5 |
| North Macedonia | 2005 | 3 | 11.5 | 2.2 | 18.8 | 3.4 | 29.6 | 9.3 | 2.0 | 19.0 | 9.8 |
|  | 2018 | 3 | 4.3 | 1.8 | 41.9 | 2.4 | 55.7 | 6.4 | 3.5 | 9.0 | 2.6 |
|  | Absolute annual change (p.p.) | | -0.55 | -0.03 | 1.78 | -0.08 | 2.01 | -0.22 | 0.12 | -0.77 | -0.55 |
|  | Relative annual change (%) | | -7.2 | -1.3 | 6.4 | -2.6 | 5.0 | -2.8 | 4.5 | -5.6 | -9.7 |
| Malawi | 2000 | 9 | 54.6 | 4.3 | 7.8 | 7.0 | 12.7 | 21.9 | 1.6 | 59.7 | 37.8 |
|  | 2019 | 9 | 35.5 | 2.8 | 8.0 | 5.4 | 15.3 | 20.0 | 2.0 | 40.0 | 20.0 |
|  | Absolute annual change (p.p.) | | -1.01 | -0.08 | 0.01 | -0.08 | 0.14 | -0.10 | 0.02 | -1.04 | -0.94 |
|  | Relative annual change (%) | | -2.2 | -2.1 | 0.1 | -1.3 | 1.0 | -0.5 | 1.3 | -2.1 | -3.3 |
| Mali | 2001 | 7 | 42.8 | 2.3 | 5.4 | 2.7 | 6.3 | 8.9 | 1.2 | 46.2 | 37.3 |
|  | 2018 | 7 | 27.3 | 1.8 | 6.7 | 3.5 | 13.0 | 16.7 | 2.1 | 31.9 | 15.2 |
|  | Absolute annual change (p.p.) | | -0.91 | -0.03 | 0.08 | 0.05 | 0.40 | 0.46 | 0.05 | -0.84 | -1.30 |
|  | Relative annual change (%) | | -2.6 | -1.3 | 1.3 | 1.6 | 4.4 | 3.8 | 3.2 | -2.2 | -5.2 |
| Mauritania | 2007 | 5 | 31.4 | 3.5 | 11.2 | 8.5 | 27.1 | 18.3 | 2.2 | 33.6 | 15.4 |
|  | 2015 | 5 | 28.2 | 3.1 | 10.8 | 7.4 | 26.2 | 20.4 | 3.1 | 30.2 | 9.8 |
|  | Absolute annual change (p.p.) | | -0.40 | -0.06 | -0.05 | -0.14 | -0.11 | 0.26 | 0.11 | -0.43 | -0.69 |
|  | Relative annual change (%) | | -1.3 | -1.8 | -0.4 | -1.7 | -0.4 | 1.4 | 4.3 | -1.3 | -5.5 |
| Mongolia | 2005 | 3 | 27.5 | 2.1 | 7.6 | 5.9 | 21.3 | 16.3 | 1.6 | 42.4 | 26.2 |
|  | 2018 | 3 | 9.4 | 1.1 | 11.4 | 3.3 | 34.6 | 8.8 | 2.0 | 17.6 | 8.7 |
|  | Absolute annual change (p.p.) | | -1.39 | -0.08 | 0.29 | -0.20 | 1.02 | -0.57 | 0.03 | -1.91 | -1.34 |
|  | Relative annual change (%) | | -7.9 | -5.0 | 3.1 | -4.4 | 3.8 | -4.6 | 1.7 | -6.6 | -8.1 |
| Namibia | 2000 | 6 | 29.6 | 4.3 | 14.5 | 6.7 | 22.7 | 25.7 | 2.8 | 40.3 | 14.6 |
|  | 2013 | 6 | 22.7 | 3.3 | 14.6 | 5.2 | 22.8 | 15.7 | 2.2 | 29.1 | 13.4 |
|  | Absolute annual change (p.p.) | | -0.53 | -0.08 | 0.01 | -0.12 | 0.01 | -0.77 | -0.05 | -0.86 | -0.09 |
|  | Relative annual change (%) | | -2.0 | -2.0 | 0.0 | -2.0 | 0.0 | -3.7 | -1.8 | -2.5 | -0.7 |
| Nepal | 2011 | 10 | 40.5 | 5.3 | 13.1 | 7.7 | 18.9 | 31.8 | 2.7 | 50.7 | 18.9 |
|  | 2016 | 10 | 35.7 | 3.6 | 10.1 | 5.6 | 15.7 | 22.8 | 2.1 | 43.8 | 21.0 |
|  | Absolute annual change (p.p.) | | -0.97 | -0.34 | -0.60 | -0.41 | -0.64 | -1.79 | -0.12 | -1.37 | 0.42 |
|  | Relative annual change (%) | | -2.5 | -7.4 | -5.1 | -6.1 | -3.6 | -6.4 | -4.9 | -2.9 | 2.1 |
| Niger | 2006 | 5 | 54.7 | 5.7 | 10.5 | 9.7 | 17.8 | 25.1 | 1.6 | 68.6 | 43.5 |
|  | 2012 | 5 | 43.6 | 3.4 | 7.7 | 3.2 | 7.4 | 11.7 | 1.3 | 46.2 | 34.5 |
|  | Absolute annual change (p.p.) | | -1.85 | -0.40 | -0.46 | -1.08 | -1.73 | -2.24 | -0.04 | -3.74 | -1.50 |
|  | Relative annual change (%) | | -3.7 | -8.5 | -5.0 | -16.7 | -13.5 | -12.0 | -2.7 | -6.4 | -3.8 |
| Nigeria | 2008 | 6 | 40.7 | 7.1 | 17.5 | 8.9 | 21.8 | 27.7 | 2.2 | 50.2 | 22.5 |
|  | 2018 | 6 | 36.8 | 13.7 | 37.2 | 14.7 | 40.0 | 38.7 | 3.3 | 55.6 | 16.9 |
|  | Absolute annual change (p.p.) | | -0.39 | 0.66 | 1.97 | 0.58 | 1.82 | 1.10 | 0.11 | 0.54 | -0.56 |
|  | Relative annual change (%) | | -1.0 | 6.8 | 7.8 | 5.2 | 6.2 | 3.4 | 4.0 | 1.0 | -2.8 |
| Pakistan | 2012 | 7 | 45.1 | 8.6 | 19.0 | 9.9 | 21.9 | 41.0 | 2.6 | 66.2 | 25.1 |
|  | 2017 | 7 | 37.5 | 8.2 | 21.8 | 8.1 | 21.5 | 27.7 | 2.0 | 54.6 | 27.0 |
|  | Absolute annual change (p.p.) | | -1.51 | -0.08 | 0.55 | -0.36 | -0.08 | -2.67 | -0.12 | -2.30 | 0.37 |
|  | Relative annual change (%) | | -3.6 | -0.9 | 2.8 | -4.0 | -0.4 | -7.6 | -5.1 | -3.8 | 1.4 |
| Peru | 2000 | 3 | 31.4 | 7.7 | 24.5 | 15.2 | 48.5 | 29.4 | 2.1 | 56.1 | 26.7 |
|  | 2020 | 3 | 12.0 | 3.5 | 29.5 | 16.4 | 136.1 | 30.0 | 4.1 | 39.7 | 9.6 |
|  | Absolute annual change (p.p.) | | -0.97 | -0.21 | 0.25 | 0.06 | 4.38 | 0.03 | 0.10 | -0.82 | -0.86 |
|  | Relative annual change (%) | | -4.7 | -3.8 | 0.9 | 0.4 | 5.3 | 0.1 | 3.4 | -1.7 | -5.0 |
| Sao Tome and Principe | 2014 | 2 | 17.2 | 0.0 | 0.1 | 0.0 | 0.1 | 0.0 | 1.0 | 17.2 | 17.2 |
|  | 2019 | 2 | 11.8 | 1.0 | 8.5 | 2.9 | 24.5 | 5.8 | 1.5 | 17.1 | 11.3 |
|  | Absolute annual change (p.p.) | | -1.07 | 0.20 | 1.68 | 0.58 | 4.88 | 1.15 | 0.10 | -0.02 | -1.18 |
|  | Relative annual change (%) | | -7.2 | 156.7 | 176.6 | 181.2 | 203.0 | 181.2 | 8.6 | -0.1 | -8.1 |
| Senegal | 2005 | 6 | 19.7 | 5.3 | 26.9 | 4.7 | 23.8 | 13.3 | 1.9 | 28.6 | 15.2 |
|  | 2019 | 6 | 17.9 | 2.1 | 11.5 | 3.0 | 16.8 | 11.2 | 2.2 | 20.6 | 9.4 |
|  | Absolute annual change (p.p.) | | -0.13 | -0.23 | -1.10 | -0.12 | -0.50 | -0.15 | 0.02 | -0.57 | -0.42 |
|  | Relative annual change (%) | | -0.7 | -6.5 | -5.9 | -3.2 | -2.5 | -1.2 | 1.1 | -2.3 | -3.4 |
| Serbia | 2005 | 4 | 8.2 | 1.5 | 19.0 | 4.6 | 56.1 | 17.8 | 3.5 | 25.0 | 7.2 |
|  | 2019 | 4 | 5.4 | 1.2 | 22.0 | 3.6 | 66.2 | 13.3 | 6.5 | 15.8 | 2.4 |
|  | Absolute annual change (p.p.) | | -0.19 | -0.03 | 0.21 | -0.07 | 0.72 | -0.32 | 0.22 | -0.66 | -0.34 |
|  | Relative annual change (%) | | -2.9 | -1.8 | 1.1 | -1.7 | 1.2 | -2.0 | 4.6 | -3.2 | -7.5 |
| Sierra Leone | 2008 | 7 | 37.1 | 2.3 | 6.1 | 7.0 | 18.7 | 20.9 | 2.1 | 39.3 | 18.4 |
|  | 2019 | 7 | 29.5 | 1.4 | 4.6 | 4.2 | 14.2 | 16.7 | 1.8 | 37.0 | 20.3 |
|  | Absolute annual change (p.p.) | | -0.69 | -0.08 | -0.14 | -0.25 | -0.41 | -0.38 | -0.03 | -0.22 | 0.17 |
|  | Relative annual change (%) | | -2.1 | -4.6 | -2.6 | -4.5 | -2.5 | -2.0 | -1.4 | -0.6 | 0.9 |
| Suriname | 2006 | 6 | 10.7 | 2.4 | 22.9 | 3.4 | 31.4 | 13.8 | 3.5 | 19.3 | 5.5 |
|  | 2018 | 6 | 8.4 | 0.9 | 11.2 | 1.4 | 16.5 | 4.4 | 1.7 | 11.0 | 6.6 |
|  | Absolute annual change (p.p.) | | -0.19 | -0.13 | -0.98 | -0.16 | -1.24 | -0.78 | -0.15 | -0.69 | 0.09 |
|  | Relative annual change (%) | | -2.0 | -7.7 | -5.8 | -7.1 | -5.2 | -9.1 | -6.0 | -4.6 | 1.6 |
| Tajikistan | 2005 | 3 | 33.1 | 0.8 | 2.4 | 8.1 | 24.6 | 23.5 | 3.3 | 33.7 | 10.1 |
|  | 2017 | 3 | 17.5 | 0.8 | 4.7 | 1.3 | 7.5 | 2.9 | 1.2 | 18.1 | 15.2 |
|  | Absolute annual change (p.p.) | | -1.30 | 0.00 | 0.19 | -0.57 | -1.43 | -1.72 | -0.18 | -1.30 | 0.42 |
|  | Relative annual change (%) | | -5.2 | 0.1 | 5.6 | -14.1 | -9.4 | -16.0 | -8.2 | -5.1 | 3.4 |
| Thailand | 2005 | 2 | 15.7 | 3.3 | 20.8 | 8.5 | 54.1 | 17.0 | 2.2 | 30.9 | 13.9 |
|  | 2019 | 2 | 13.3 | 1.2 | 9.1 | 4.9 | 37.2 | 9.9 | 1.8 | 22.5 | 12.6 |
|  | Absolute annual change (p.p.) | | -0.18 | -0.15 | -0.83 | -0.26 | -1.21 | -0.51 | -0.03 | -0.60 | -0.09 |
|  | Relative annual change (%) | | -1.2 | -6.9 | -5.7 | -3.8 | -2.6 | -3.8 | -1.6 | -2.3 | -0.7 |
| Timor-Leste | 2009 | 2 | 57.6 | 1.8 | 3.1 | 3.5 | 6.1 | 7.0 | 1.1 | 63.4 | 56.4 |
|  | 2016 | 2 | 45.7 | 0.6 | 1.3 | 3.9 | 8.4 | 7.7 | 1.2 | 53.2 | 45.5 |
|  | Absolute annual change (p.p.) | | -1.70 | -0.17 | -0.26 | 0.05 | 0.33 | 0.10 | 0.01 | -1.46 | -1.56 |
|  | Relative annual change (%) | | -3.3 | -14.8 | -11.9 | 1.3 | 4.7 | 1.3 | 0.6 | -2.5 | -3.0 |
| Togo | 2006 | 7 | 31.1 | 4.9 | 15.8 | 4.5 | 14.4 | 18.5 | 1.8 | 41.9 | 23.5 |
|  | 2017 | 7 | 23.8 | 3.7 | 15.5 | 4.9 | 20.5 | 18.2 | 2.3 | 32.0 | 13.8 |
|  | Absolute annual change (p.p.) | | -0.66 | -0.11 | -0.03 | 0.04 | 0.55 | -0.02 | 0.05 | -0.91 | -0.88 |
|  | Relative annual change (%) | | -2.4 | -2.6 | -0.2 | 0.8 | 3.2 | -0.1 | 2.4 | -2.4 | -4.7 |
| Türkiye | 2003 | 2 | 15.3 | 4.8 | 31.5 | 14.0 | 91.7 | 28.1 | 3.2 | 40.7 | 12.6 |
|  | 2013 | 2 | 10.1 | 1.1 | 10.8 | 5.6 | 55.9 | 11.3 | 2.2 | 20.7 | 9.4 |
|  | Absolute annual change (p.p.) | | -0.52 | -0.37 | -2.07 | -0.84 | -3.58 | -1.68 | -0.10 | -2.00 | -0.32 |
|  | Relative annual change (%) | | -4.1 | -13.8 | -10.2 | -8.7 | -4.8 | -8.7 | -3.8 | -6.5 | -2.9 |
| Turkmenistan | 2006 | 4 | 18.9 | 0.9 | 4.6 | 5.6 | 29.5 | 20.9 | 3.6 | 28.8 | 8.0 |
|  | 2019 | 4 | 7.1 | 0.5 | 6.8 | 1.5 | 21.7 | 5.1 | 2.0 | 10.2 | 5.1 |
|  | Absolute annual change (p.p.) | | -0.90 | -0.03 | 0.17 | -0.31 | -0.60 | -1.22 | -0.13 | -1.43 | -0.22 |
|  | Relative annual change (%) | | -7.2 | -4.3 | 3.1 | -9.4 | -2.3 | -10.3 | -4.5 | -7.7 | -3.3 |
| Uganda | 2006 | 7 | 38.3 | 4.1 | 10.6 | 4.9 | 12.8 | 20.4 | 1.8 | 44.9 | 24.5 |
|  | 2016 | 7 | 28.9 | 3.1 | 10.8 | 3.9 | 13.3 | 16.2 | 1.9 | 34.8 | 18.7 |
|  | Absolute annual change (p.p.) | | -0.95 | -0.09 | 0.02 | -0.11 | 0.05 | -0.42 | 0.00 | -1.01 | -0.59 |
|  | Relative annual change (%) | | -2.8 | -2.6 | 0.2 | -2.4 | 0.4 | -2.3 | 0.2 | -2.5 | -2.7 |
| Zambia | 2001 | 8 | 52.6 | 4.1 | 7.8 | 5.3 | 10.1 | 19.7 | 1.5 | 60.6 | 40.9 |
|  | 2013 | 8 | 40.1 | 2.1 | 5.2 | 3.3 | 8.2 | 10.3 | 1.3 | 44.0 | 33.6 |
|  | Absolute annual change (p.p.) | | -1.04 | -0.17 | -0.21 | -0.17 | -0.16 | -0.78 | -0.01 | -1.39 | -0.61 |
|  | Relative annual change (%) | | -2.2 | -5.4 | -3.3 | -3.9 | -1.7 | -5.2 | -1.0 | -2.6 | -1.6 |
| Zimbabwe | 2009 | 3 | 35.1 | 1.5 | 4.3 | 2.5 | 7.1 | 6.6 | 1.2 | 36.1 | 29.5 |
|  | 2019 | 3 | 23.5 | 0.6 | 2.7 | 1.2 | 5.3 | 2.7 | 1.1 | 23.9 | 21.2 |
|  | Absolute annual change (p.p.) | | -1.16 | -0.09 | -0.15 | -0.12 | -0.18 | -0.39 | -0.01 | -1.22 | -0.84 |
|  | Relative annual change (%) | | -3.9 | -8.2 | -4.4 | -6.7 | -2.9 | -8.5 | -0.8 | -4.1 | -3.3 |

|  |  |  |  | **Weighted summary** | | **Unweighted summary** | |  |  |  |  |
| --- | --- | --- | --- | --- | --- | --- | --- | --- | --- | --- | --- |
|  |  |  | **National CCI prevalence** | **Absolute** | **Relative** | **Absolute** | **Relative** | **Extreme group comparisons** | | **Prevalence (%) in worst and best groups** | |
| **Country** | **Year** | **Nº of groups** | **%** | **WMDOM** | **WMROM** | **UMDOM** | **UMROM** | **Hi/lo diff** | **Hi/lo ratio** | **Worst** | **Best** |
| Albania | 2008 | 2 | 64.0 | 0.2 | 0.3 | 1.9 | 3.0 | 3.8 | 1.1 | 67.8 | 63.9 |
|  | 2017 | 2 | 61.5 | 0.2 | 0.4 | 0.5 | 0.8 | 1.0 | 1.0 | 61.5 | 60.5 |
|  | Absolute annual change (p.p.) | | -0.28 | 0.00 | 0.00 | -0.16 | -0.24 | -0.32 | 0.00 | -0.69 | -0.38 |
|  | Relative annual change (%) | | -0.4 | 0.3 | 0.8 | -14.0 | -13.6 | -14.0 | -0.5 | -1.1 | -0.6 |
| Belize | 2006 | 4 | 68.7 | 2.3 | 3.4 | 3.0 | 4.4 | 7.7 | 1.1 | 70.3 | 62.6 |
|  | 2015 | 4 | 76.5 | 2.8 | 3.6 | 3.5 | 4.5 | 11.2 | 1.2 | 78.9 | 67.7 |
|  | Absolute annual change (p.p.) | | 0.86 | 0.05 | 0.02 | 0.05 | 0.01 | 0.38 | 0.00 | 0.96 | 0.57 |
|  | Relative annual change (%) | | 1.2 | 1.9 | 0.7 | 1.5 | 0.3 | 4.2 | 0.4 | 1.3 | 0.9 |
| Benin | 2001 | 8 | 46.9 | 5.3 | 11.2 | 5.6 | 11.9 | 28.3 | 2.2 | 52.6 | 24.3 |
|  | 2017 | 8 | 49.2 | 6.1 | 12.4 | 5.6 | 11.4 | 30.5 | 2.1 | 57.0 | 26.6 |
|  | Absolute annual change (p.p.) | | 0.14 | 0.05 | 0.07 | 0.00 | -0.03 | 0.14 | 0.00 | 0.27 | 0.14 |
|  | Relative annual change (%) | | 0.3 | 0.9 | 0.6 | 0.0 | -0.3 | 0.5 | 0.0 | 0.5 | 0.5 |
| Burkina Faso | 2003 | 7 | 34.4 | 2.1 | 6.2 | 4.7 | 13.6 | 21.9 | 1.9 | 46.4 | 24.5 |
|  | 2010 | 7 | 54.6 | 4.1 | 7.5 | 5.1 | 9.4 | 17.6 | 1.4 | 62.9 | 45.3 |
|  | Absolute annual change (p.p.) | | 2.88 | 0.28 | 0.18 | 0.06 | -0.60 | -0.61 | -0.07 | 2.36 | 2.97 |
|  | Relative annual change (%) | | 6.8 | 9.7 | 2.7 | 1.3 | -5.1 | -3.1 | -4.3 | 4.4 | 9.2 |
| Cameroon | 2011 | 8 | 47.6 | 12.8 | 26.9 | 11.8 | 24.7 | 36.4 | 2.3 | 65.0 | 28.6 |
|  | 2014 | 8 | 50.8 | 10.5 | 20.7 | 9.5 | 18.6 | 29.9 | 1.8 | 65.6 | 35.7 |
|  | Absolute annual change (p.p.) | | 1.05 | -0.78 | -2.10 | -0.77 | -2.02 | -2.17 | -0.15 | 0.19 | 2.37 |
|  | Relative annual change (%) | | 2.2 | -6.5 | -8.5 | -7.0 | -8.9 | -6.4 | -6.8 | 0.3 | 7.7 |
| Central African Republic | 2006 | 9 | 50.1 | 6.5 | 13.0 | 6.8 | 13.7 | 18.0 | 1.5 | 53.1 | 35.1 |
|  | 2018 | 9 | 33.6 | 2.2 | 6.6 | 2.7 | 8.1 | 9.4 | 1.3 | 40.4 | 31.0 |
|  | Absolute annual change (p.p.) | | -1.37 | -0.36 | -0.54 | -0.34 | -0.46 | -0.72 | -0.02 | -1.06 | -0.34 |
|  | Relative annual change (%) | | -3.3 | -8.6 | -5.5 | -7.4 | -4.2 | -5.3 | -1.2 | -2.3 | -1.0 |
| Chad | 2004 | 8 | 15.9 | 3.2 | 19.8 | 4.4 | 27.9 | 16.5 | 2.9 | 25.0 | 8.5 |
|  | 2014 | 8 | 28.0 | 6.1 | 21.8 | 6.7 | 24.0 | 21.3 | 2.5 | 35.4 | 14.1 |
|  | Absolute annual change (p.p.) | | 1.20 | 0.29 | 0.19 | 0.23 | -0.39 | 0.48 | -0.04 | 1.04 | 0.57 |
|  | Relative annual change (%) | | 5.8 | 6.8 | 0.9 | 4.2 | -1.5 | 2.6 | -1.6 | 3.5 | 5.2 |
| Congo DR | 2007 | 8 | 41.7 | 6.9 | 16.5 | 8.2 | 19.6 | 26.0 | 1.8 | 57.3 | 31.4 |
|  | 2013 | 8 | 47.1 | 4.1 | 8.8 | 5.1 | 10.9 | 18.1 | 1.4 | 58.6 | 40.5 |
|  | Absolute annual change (p.p.) | | 0.90 | -0.46 | -1.28 | -0.51 | -1.45 | -1.31 | -0.06 | 0.21 | 1.52 |
|  | Relative annual change (%) | | 2.0 | -8.1 | -9.9 | -7.5 | -9.3 | -5.8 | -3.8 | 0.4 | 4.3 |
| Congo Republic | 2011 | 6 | 57.9 | 2.5 | 4.4 | 2.8 | 4.8 | 6.5 | 1.1 | 60.4 | 53.9 |
|  | 2014 | 6 | 55.2 | 3.1 | 5.6 | 4.2 | 7.7 | 13.1 | 1.3 | 57.7 | 44.6 |
|  | Absolute annual change (p.p.) | | -0.89 | 0.18 | 0.41 | 0.50 | 0.98 | 2.21 | 0.06 | -0.87 | -3.09 |
|  | Relative annual change (%) | | -1.6 | 6.8 | 8.5 | 15.6 | 17.4 | 26.5 | 4.9 | -1.5 | -6.1 |
| Costa Rica | 2011 | 3 | 84.3 | 1.5 | 1.8 | 5.8 | 6.9 | 9.8 | 1.1 | 85.2 | 75.3 |
|  | 2018 | 3 | 83.6 | 0.4 | 0.5 | 2.7 | 3.3 | 8.1 | 1.1 | 88.1 | 80.0 |
|  | Absolute annual change (p.p.) | | -0.11 | -0.16 | -0.18 | -0.44 | -0.52 | -0.25 | 0.00 | 0.42 | 0.67 |
|  | Relative annual change (%) | | -0.1 | -17.4 | -17.3 | -10.2 | -10.1 | -2.8 | -0.4 | 0.5 | 0.9 |
| Côte d'Ivoire | 2006 | 5 | 56.1 | 4.0 | 7.1 | 4.6 | 8.2 | 15.4 | 1.4 | 59.2 | 43.8 |
|  | 2016 | 5 | 47.5 | 2.7 | 5.7 | 2.6 | 5.4 | 7.6 | 1.2 | 52.7 | 45.1 |
|  | Absolute annual change (p.p.) | | -0.86 | -0.13 | -0.14 | -0.20 | -0.28 | -0.79 | -0.02 | -0.65 | 0.13 |
|  | Relative annual change (%) | | -1.7 | -3.7 | -2.1 | -5.7 | -4.1 | -6.9 | -1.5 | -1.2 | 0.3 |
| Dominican Republic | 2014 | 2 | 77.6 | 1.4 | 1.8 | 4.7 | 6.0 | 9.3 | 1.1 | 78.3 | 69.0 |
|  | 2019 | 2 | 78.6 | 1.4 | 1.8 | 6.3 | 8.0 | 12.5 | 1.2 | 79.4 | 66.8 |
|  | Absolute annual change (p.p.) | | 0.21 | 0.00 | 0.00 | 0.32 | 0.40 | 0.65 | 0.01 | 0.21 | -0.44 |
|  | Relative annual change (%) | | 0.3 | 0.1 | -0.2 | 6.1 | 5.9 | 6.1 | 0.9 | 0.3 | -0.6 |
| Ethiopia | 2000 | 4 | 16.3 | 4.0 | 24.4 | 5.8 | 35.7 | 16.8 | 2.3 | 29.6 | 12.8 |
|  | 2016 | 4 | 45.1 | 8.6 | 19.1 | 9.4 | 20.7 | 27.1 | 1.8 | 61.7 | 34.5 |
|  | Absolute annual change (p.p.) | | 1.80 | 0.29 | -0.33 | 0.22 | -0.93 | 0.65 | -0.03 | 2.00 | 1.35 |
|  | Relative annual change (%) | | 6.6 | 4.9 | -1.5 | 3.0 | -3.3 | 3.1 | -1.6 | 4.7 | 6.4 |
| Gabon | 2000 | 8 | 46.3 | 1.8 | 3.9 | 2.8 | 6.1 | 12.2 | 1.3 | 51.7 | 39.5 |
|  | 2012 | 8 | 58.1 | 2.2 | 3.7 | 2.5 | 4.3 | 10.8 | 1.2 | 62.0 | 51.2 |
|  | Absolute annual change (p.p.) | | 0.99 | 0.03 | -0.01 | -0.03 | -0.15 | -0.12 | -0.01 | 0.86 | 0.98 |
|  | Relative annual change (%) | | 1.9 | 1.6 | -0.3 | -1.0 | -2.8 | -1.0 | -0.6 | 1.5 | 2.2 |
| Gambia | 2010 | 5 | 59.7 | 2.0 | 3.4 | 2.3 | 3.9 | 8.4 | 1.2 | 64.0 | 55.6 |
|  | 2019 | 5 | 67.4 | 1.2 | 1.8 | 1.2 | 1.8 | 4.0 | 1.1 | 69.0 | 64.9 |
|  | Absolute annual change (p.p.) | | 0.86 | -0.09 | -0.17 | -0.13 | -0.24 | -0.48 | -0.01 | 0.55 | 1.03 |
|  | Relative annual change (%) | | 1.4 | -5.2 | -6.5 | -7.1 | -8.4 | -7.8 | -0.9 | 0.8 | 1.7 |
| Ghana | 2003 | 7 | 53.4 | 3.8 | 7.1 | 3.4 | 6.3 | 10.3 | 1.2 | 57.0 | 46.7 |
|  | 2017 | 7 | 65.3 | 1.1 | 1.7 | 1.7 | 2.5 | 5.0 | 1.1 | 68.0 | 63.0 |
|  | Absolute annual change (p.p.) | | 0.85 | -0.19 | -0.39 | -0.12 | -0.27 | -0.38 | -0.01 | 0.79 | 1.16 |
|  | Relative annual change (%) | | 1.5 | -8.6 | -9.9 | -4.9 | -6.3 | -5.0 | -0.9 | 1.3 | 2.2 |
| Guinea | 2005 | 5 | 38.0 | 5.6 | 14.7 | 6.6 | 17.3 | 15.4 | 1.5 | 47.1 | 31.7 |
|  | 2018 | 5 | 44.3 | 7.1 | 16.1 | 7.1 | 16.1 | 24.2 | 1.7 | 57.3 | 33.1 |
|  | Absolute annual change (p.p.) | | 0.48 | 0.12 | 0.11 | 0.04 | -0.09 | 0.68 | 0.02 | 0.78 | 0.11 |
|  | Relative annual change (%) | | 1.2 | 1.9 | 0.7 | 0.6 | -0.6 | 3.5 | 1.2 | 1.5 | 0.3 |
| Guinea-Bissau | 2006 | 7 | 51.6 | 5.5 | 10.8 | 5.8 | 11.2 | 25.6 | 1.6 | 66.7 | 41.1 |
|  | 2018 | 7 | 56.6 | 3.7 | 6.5 | 4.8 | 8.5 | 14.6 | 1.3 | 66.1 | 51.5 |
|  | Absolute annual change (p.p.) | | 0.42 | -0.15 | -0.35 | -0.08 | -0.23 | -0.92 | -0.03 | -0.05 | 0.87 |
|  | Relative annual change (%) | | 0.8 | -3.3 | -4.1 | -1.5 | -2.3 | -4.6 | -1.9 | -0.1 | 1.9 |
| Guyana | 2006 | 4 | 67.1 | 3.5 | 5.1 | 4.6 | 6.9 | 13.8 | 1.2 | 71.1 | 57.3 |
|  | 2019 | 4 | 69.3 | 3.2 | 4.6 | 3.9 | 5.6 | 7.9 | 1.1 | 71.6 | 63.7 |
|  | Absolute annual change (p.p.) | | 0.17 | -0.02 | -0.04 | -0.06 | -0.10 | -0.46 | -0.01 | 0.04 | 0.49 |
|  | Relative annual change (%) | | 0.3 | -0.6 | -0.7 | -1.3 | -1.6 | -4.2 | -0.8 | 0.1 | 0.8 |
| Honduras | 2011 | 5 | 79.7 | 0.6 | 0.8 | 3.4 | 4.2 | 13.1 | 1.2 | 86.9 | 73.7 |
|  | 2019 | 5 | 81.3 | 0.4 | 0.5 | 3.0 | 3.7 | 12.2 | 1.2 | 82.2 | 70.0 |
|  | Absolute annual change (p.p.) | | 0.20 | -0.02 | -0.03 | -0.05 | -0.07 | -0.12 | 0.00 | -0.59 | -0.47 |
|  | Relative annual change (%) | | 0.2 | -4.5 | -4.7 | -1.5 | -1.7 | -0.9 | 0.0 | -0.7 | -0.7 |
| India | 2005 | 17 | 55.4 | 10.7 | 19.3 | 11.7 | 21.1 | 37.1 | 1.8 | 81.3 | 44.2 |
|  | 2015 | 17 | 71.4 | 7.5 | 10.6 | 7.9 | 11.0 | 22.7 | 1.4 | 84.4 | 61.6 |
|  | Absolute annual change (p.p.) | | 1.61 | -0.31 | -0.87 | -0.38 | -1.01 | -1.43 | -0.05 | 0.31 | 1.74 |
|  | Relative annual change (%) | | 2.6 | -3.4 | -5.8 | -3.9 | -6.3 | -4.8 | -2.9 | 0.4 | 3.4 |
| Kenya | 2003 | 12 | 52.4 | 7.3 | 13.9 | 9.7 | 18.5 | 46.4 | 2.5 | 78.1 | 31.7 |
|  | 2014 | 12 | 70.4 | 5.9 | 8.4 | 7.8 | 11.1 | 39.8 | 1.9 | 82.6 | 42.8 |
|  | Absolute annual change (p.p.) | | 1.63 | -0.13 | -0.50 | -0.17 | -0.67 | -0.60 | -0.05 | 0.41 | 1.01 |
|  | Relative annual change (%) | | 2.7 | -1.9 | -4.5 | -1.9 | -4.5 | -1.4 | -2.2 | 0.5 | 2.8 |
| Kyrgyzstan | 2012 | 3 | 70.0 | 0.9 | 1.2 | 3.0 | 4.3 | 8.9 | 1.1 | 76.9 | 68.0 |
|  | 2018 | 3 | 75.3 | 2.3 | 3.1 | 5.6 | 7.5 | 11.4 | 1.2 | 85.6 | 74.2 |
|  | Absolute annual change (p.p.) | | 0.88 | 0.24 | 0.31 | 0.43 | 0.52 | 0.42 | 0.00 | 1.45 | 1.03 |
|  | Relative annual change (%) | | 1.2 | 17.7 | 16.3 | 10.9 | 9.5 | 4.3 | 0.3 | 1.8 | 1.5 |
| Lao PDR | 2011 | 3 | 54.8 | 8.3 | 15.1 | 12.8 | 23.3 | 34.9 | 2.2 | 63.2 | 28.3 |
|  | 2017 | 3 | 62.6 | 8.0 | 12.8 | 10.6 | 16.9 | 26.0 | 1.6 | 69.6 | 43.6 |
|  | Absolute annual change (p.p.) | | 1.30 | -0.05 | -0.39 | -0.37 | -1.08 | -1.48 | -0.11 | 1.06 | 2.55 |
|  | Relative annual change (%) | | 2.2 | -0.6 | -2.8 | -3.1 | -5.3 | -4.8 | -5.4 | 1.6 | 7.5 |
| Malawi | 2000 | 9 | 55.9 | 2.8 | 5.1 | 3.3 | 5.9 | 11.3 | 1.2 | 63.9 | 52.6 |
|  | 2019 | 9 | 76.2 | 1.2 | 1.6 | 1.5 | 1.9 | 5.0 | 1.1 | 78.4 | 73.3 |
|  | Absolute annual change (p.p.) | | 1.06 | -0.09 | -0.18 | -0.10 | -0.21 | -0.33 | -0.01 | 0.76 | 1.09 |
|  | Relative annual change (%) | | 1.6 | -4.5 | -6.0 | -4.2 | -5.7 | -4.2 | -0.7 | 1.1 | 1.8 |
| Mali | 2001 | 7 | 29.3 | 2.6 | 8.8 | 3.7 | 12.7 | 15.2 | 1.8 | 33.5 | 18.4 |
|  | 2018 | 7 | 49.3 | 2.8 | 5.7 | 3.0 | 6.1 | 10.0 | 1.2 | 54.1 | 44.1 |
|  | Absolute annual change (p.p.) | | 1.17 | 0.01 | -0.18 | -0.04 | -0.39 | -0.30 | -0.04 | 1.21 | 1.51 |
|  | Relative annual change (%) | | 3.1 | 0.5 | -2.5 | -1.2 | -4.2 | -2.4 | -2.3 | 2.8 | 5.3 |
| Mauritania | 2007 | 5 | 49.8 | 3.4 | 6.9 | 4.7 | 9.5 | 13.9 | 1.3 | 60.1 | 46.2 |
|  | 2015 | 5 | 49.0 | 0.8 | 1.7 | 6.3 | 12.8 | 27.6 | 1.6 | 70.1 | 42.5 |
|  | Absolute annual change (p.p.) | | -0.11 | -0.33 | -0.65 | 0.19 | 0.41 | 1.71 | 0.04 | 1.25 | -0.46 |
|  | Relative annual change (%) | | -0.2 | -16.2 | -16.0 | 3.5 | 3.7 | 8.9 | 3.0 | 1.9 | -1.0 |
| Moldova | 2005 | 4 | 73.0 | 0.7 | 0.9 | 2.1 | 2.9 | 8.3 | 1.1 | 78.0 | 69.8 |
|  | 2012 | 4 | 79.4 | 0.8 | 1.0 | 2.4 | 3.1 | 8.1 | 1.1 | 84.0 | 76.0 |
|  | Absolute annual change (p.p.) | | 0.93 | 0.02 | 0.01 | 0.05 | 0.02 | -0.03 | 0.00 | 0.86 | 0.88 |
|  | Relative annual change (%) | | 1.2 | 2.5 | 1.2 | 2.1 | 0.8 | -0.3 | -0.2 | 1.1 | 1.2 |
| Mongolia | 2005 | 3 | 81.9 | 1.2 | 1.5 | 5.3 | 6.4 | 12.1 | 1.2 | 81.9 | 69.8 |
|  | 2018 | 3 | 79.6 | 0.6 | 0.8 | 3.2 | 4.0 | 9.5 | 1.1 | 81.1 | 71.6 |
|  | Absolute annual change (p.p.) | | -0.18 | -0.05 | -0.06 | -0.16 | -0.19 | -0.20 | 0.00 | -0.07 | 0.14 |
|  | Relative annual change (%) | | -0.2 | -5.4 | -5.2 | -3.9 | -3.6 | -1.9 | -0.3 | -0.1 | 0.2 |
| Mozambique | 2003 | 10 | 51.6 | 7.9 | 15.3 | 8.3 | 16.1 | 42.5 | 2.4 | 73.9 | 31.4 |
|  | 2015 | 10 | 61.2 | 6.1 | 10.0 | 6.4 | 10.4 | 28.2 | 1.6 | 77.8 | 49.6 |
|  | Absolute annual change (p.p.) | | 0.80 | -0.15 | -0.45 | -0.16 | -0.48 | -1.19 | -0.07 | 0.33 | 1.52 |
|  | Relative annual change (%) | | 1.4 | -2.1 | -3.5 | -2.2 | -3.6 | -3.4 | -3.3 | 0.4 | 3.9 |
| Namibia | 2000 | 6 | 69.0 | 2.6 | 3.8 | 3.6 | 5.2 | 15.5 | 1.2 | 79.0 | 63.5 |
|  | 2013 | 6 | 77.0 | 1.4 | 1.8 | 1.9 | 2.5 | 6.6 | 1.1 | 77.7 | 71.1 |
|  | Absolute annual change (p.p.) | | 0.61 | -0.09 | -0.15 | -0.13 | -0.21 | -0.69 | -0.01 | -0.10 | 0.59 |
|  | Relative annual change (%) | | 0.8 | -4.7 | -5.5 | -4.7 | -5.5 | -6.4 | -1.0 | -0.1 | 0.9 |
| Nepal | 2011 | 10 | 60.0 | 5.0 | 8.4 | 6.1 | 10.1 | 22.1 | 1.5 | 71.2 | 49.1 |
|  | 2016 | 10 | 64.7 | 4.1 | 6.4 | 4.2 | 6.5 | 14.4 | 1.2 | 72.6 | 58.2 |
|  | Absolute annual change (p.p.) | | 0.94 | -0.18 | -0.40 | -0.37 | -0.72 | -1.54 | -0.04 | 0.28 | 1.82 |
|  | Relative annual change (%) | | 1.5 | -3.9 | -5.3 | -7.0 | -8.4 | -8.2 | -3.0 | 0.4 | 3.4 |
| Niger | 2006 | 5 | 28.9 | 2.8 | 9.8 | 4.5 | 15.6 | 17.3 | 2.0 | 34.6 | 17.4 |
|  | 2012 | 5 | 45.4 | 3.9 | 8.6 | 6.6 | 14.6 | 23.3 | 1.8 | 53.8 | 30.5 |
|  | Absolute annual change (p.p.) | | 2.76 | 0.18 | -0.20 | 0.35 | -0.17 | 1.01 | -0.04 | 3.20 | 2.19 |
|  | Relative annual change (%) | | 7.9 | 5.5 | -2.1 | 6.7 | -1.1 | 5.1 | -2.0 | 7.6 | 9.8 |
| Nigeria | 2008 | 6 | 35.3 | 13.0 | 36.8 | 16.2 | 45.8 | 47.1 | 4.4 | 60.9 | 13.8 |
|  | 2018 | 6 | 47.5 | 9.6 | 20.3 | 11.0 | 23.1 | 36.1 | 2.3 | 63.9 | 27.8 |
|  | Absolute annual change (p.p.) | | 1.22 | -0.34 | -1.66 | -0.52 | -2.27 | -1.10 | -0.21 | 0.30 | 1.40 |
|  | Relative annual change (%) | | 3.0 | -3.0 | -5.8 | -3.8 | -6.6 | -2.6 | -6.3 | 0.5 | 7.3 |
| North Macedonia | 2005 | 3 | 70.1 | 6.0 | 8.5 | 5.8 | 8.2 | 12.2 | 1.2 | 74.9 | 62.6 |
|  | 2011 | 3 | 71.9 | 4.3 | 6.0 | 5.9 | 8.2 | 14.0 | 1.2 | 75.6 | 61.5 |
|  | Absolute annual change (p.p.) | | 0.31 | -0.28 | -0.42 | 0.02 | -0.01 | 0.30 | 0.01 | 0.12 | -0.19 |
|  | Relative annual change (%) | | 0.4 | -5.3 | -5.7 | 0.4 | -0.1 | 2.3 | 0.5 | 0.2 | -0.3 |
| Pakistan | 2006 | 7 | 48.7 | 5.8 | 12.0 | 7.0 | 14.3 | 27.5 | 1.7 | 65.8 | 38.3 |
|  | 2017 | 7 | 61.7 | 7.1 | 11.4 | 7.2 | 11.7 | 19.7 | 1.4 | 74.2 | 54.4 |
|  | Absolute annual change (p.p.) | | 1.18 | 0.11 | -0.05 | 0.02 | -0.24 | -0.71 | -0.03 | 0.76 | 1.47 |
|  | Relative annual change (%) | | 2.2 | 1.7 | -0.4 | 0.3 | -1.8 | -3.0 | -2.1 | 1.1 | 3.3 |
| Peru | 2000 | 3 | 63.2 | 4.1 | 6.6 | 11.2 | 17.6 | 19.1 | 1.4 | 65.6 | 46.5 |
|  | 2020 | 3 | 71.4 | 3.6 | 5.0 | 11.3 | 15.9 | 27.1 | 1.6 | 74.5 | 47.4 |
|  | Absolute annual change (p.p.) | | 0.41 | -0.03 | -0.08 | 0.01 | -0.09 | 0.40 | 0.01 | 0.44 | 0.04 |
|  | Relative annual change (%) | | 0.6 | -0.8 | -1.4 | 0.1 | -0.5 | 1.8 | 0.5 | 0.6 | 0.1 |
| Philippines | 2003 | 10 | 60.8 | 4.3 | 7.1 | 7.7 | 12.6 | 37.0 | 1.9 | 77.4 | 40.4 |
|  | 2017 | 10 | 70.2 | 3.9 | 5.5 | 7.7 | 10.9 | 34.6 | 1.8 | 75.7 | 41.1 |
|  | Absolute annual change (p.p.) | | 0.67 | -0.03 | -0.11 | 0.00 | -0.12 | -0.17 | -0.01 | -0.12 | 0.05 |
|  | Relative annual change (%) | | 1.0 | -0.8 | -1.8 | 0.0 | -1.0 | -0.5 | -0.3 | -0.2 | 0.1 |
| Sao Tome and Principe | 2014 | 2 | 73.0 | 0.1 | 0.1 | 0.1 | 0.1 | 0.1 | 1.0 | 73.1 | 72.9 |
|  | 2019 | 2 | 67.5 | 0.4 | 0.6 | 1.2 | 1.7 | 2.3 | 1.0 | 67.8 | 65.5 |
|  | Absolute annual change (p.p.) | | -1.09 | 0.07 | 0.11 | 0.22 | 0.33 | 0.44 | 0.01 | -1.05 | -1.49 |
|  | Relative annual change (%) | | -1.5 | 46.3 | 48.6 | 76.1 | 78.9 | 76.1 | 0.7 | -1.5 | -2.1 |
| Senegal | 2005 | 6 | 45.3 | 2.9 | 6.5 | 4.0 | 8.7 | 11.0 | 1.3 | 52.2 | 41.2 |
|  | 2019 | 6 | 61.4 | 2.3 | 3.8 | 3.6 | 5.9 | 10.1 | 1.2 | 68.3 | 58.2 |
|  | Absolute annual change (p.p.) | | 1.15 | -0.05 | -0.20 | -0.02 | -0.20 | -0.06 | -0.01 | 1.15 | 1.22 |
|  | Relative annual change (%) | | 2.2 | -1.7 | -3.8 | -0.6 | -2.7 | -0.6 | -0.5 | 1.9 | 2.5 |
| Sierra Leone | 2008 | 7 | 47.2 | 3.3 | 6.9 | 4.0 | 8.4 | 13.7 | 1.3 | 55.7 | 42.0 |
|  | 2019 | 7 | 72.9 | 3.3 | 4.6 | 3.9 | 5.4 | 11.9 | 1.2 | 80.6 | 68.8 |
|  | Absolute annual change (p.p.) | | 2.34 | 0.01 | -0.21 | 0.00 | -0.28 | -0.16 | -0.01 | 2.27 | 2.43 |
|  | Relative annual change (%) | | 4.0 | 0.2 | -3.7 | -0.1 | -4.0 | -1.3 | -1.1 | 3.4 | 4.6 |
| Tajikistan | 2005 | 3 | 68.3 | 2.5 | 3.7 | 7.4 | 10.9 | 20.9 | 1.3 | 86.4 | 65.5 |
|  | 2017 | 3 | 70.9 | 0.7 | 0.9 | 2.1 | 3.0 | 6.1 | 1.1 | 74.2 | 68.1 |
|  | Absolute annual change (p.p.) | | 0.22 | -0.15 | -0.23 | -0.44 | -0.66 | -1.23 | -0.02 | -1.02 | 0.22 |
|  | Relative annual change (%) | | 0.3 | -10.6 | -10.8 | -10.0 | -10.3 | -9.7 | -1.6 | -1.3 | 0.3 |
| Timor-Leste | 2009 | 2 | 55.6 | 0.8 | 1.4 | 1.7 | 3.1 | 3.4 | 1.1 | 56.1 | 52.6 |
|  | 2016 | 2 | 63.0 | 0.2 | 0.4 | 0.9 | 1.4 | 1.7 | 1.0 | 64.5 | 62.8 |
|  | Absolute annual change (p.p.) | | 1.05 | -0.08 | -0.14 | -0.12 | -0.25 | -0.25 | -0.01 | 1.21 | 1.45 |
|  | Relative annual change (%) | | 1.8 | -15.7 | -17.2 | -9.5 | -11.1 | -9.5 | -0.5 | 2.0 | 2.6 |
| Togo | 2006 | 6 | 50.4 | 6.4 | 12.6 | 8.1 | 16.1 | 24.8 | 1.7 | 61.3 | 36.5 |
|  | 2017 | 6 | 51.1 | 1.7 | 3.4 | 1.5 | 2.9 | 6.0 | 1.1 | 55.0 | 49.0 |
|  | Absolute annual change (p.p.) | | 0.06 | -0.42 | -0.84 | -0.60 | -1.20 | -1.72 | -0.05 | -0.58 | 1.14 |
|  | Relative annual change (%) | | 0.1 | -11.2 | -11.3 | -14.3 | -14.4 | -12.2 | -3.6 | -1.0 | 2.7 |
| Turkmenistan | 2006 | 3 | 80.5 | 2.4 | 3.0 | 2.0 | 2.5 | 4.4 | 1.1 | 82.4 | 78.0 |
|  | 2015 | 3 | 79.7 | 1.3 | 1.6 | 4.5 | 5.6 | 13.2 | 1.2 | 81.6 | 68.4 |
|  | Absolute annual change (p.p.) | | -0.09 | -0.13 | -0.15 | 0.28 | 0.35 | 0.98 | 0.02 | -0.09 | -1.07 |
|  | Relative annual change (%) | | -0.1 | -6.8 | -6.7 | 9.5 | 9.7 | 13.0 | 1.4 | -0.1 | -1.5 |
| Uganda | 2006 | 7 | 50.5 | 3.1 | 6.1 | 3.2 | 6.3 | 12.4 | 1.3 | 58.3 | 45.9 |
|  | 2016 | 7 | 65.1 | 2.0 | 3.0 | 2.1 | 3.3 | 7.3 | 1.1 | 69.2 | 61.8 |
|  | Absolute annual change (p.p.) | | 1.46 | -0.11 | -0.31 | -0.10 | -0.30 | -0.50 | -0.02 | 1.08 | 1.59 |
|  | Relative annual change (%) | | 2.6 | -4.4 | -6.8 | -3.8 | -6.2 | -5.1 | -1.3 | 1.7 | 3.0 |
| Vietnam | 2002 | 2 | 65.3 | 4.9 | 7.5 | 8.7 | 13.3 | 17.3 | 1.3 | 68.7 | 51.4 |
|  | 2013 | 2 | 78.1 | 5.4 | 6.9 | 9.0 | 11.5 | 18.0 | 1.3 | 81.8 | 63.8 |
|  | Absolute annual change (p.p.) | | 1.16 | 0.05 | -0.05 | 0.03 | -0.16 | 0.06 | -0.01 | 1.19 | 1.13 |
|  | Relative annual change (%) | | 1.6 | 0.9 | -0.7 | 0.3 | -1.3 | 0.3 | -0.4 | 1.6 | 2.0 |
| Zambia | 2001 | 8 | 59.8 | 1.3 | 2.2 | 1.6 | 2.6 | 5.4 | 1.1 | 63.6 | 58.2 |
|  | 2013 | 8 | 69.5 | 1.7 | 2.4 | 2.0 | 2.9 | 8.7 | 1.1 | 75.1 | 66.4 |
|  | Absolute annual change (p.p.) | | 0.81 | 0.03 | 0.02 | 0.04 | 0.02 | 0.28 | 0.00 | 0.96 | 0.68 |
|  | Relative annual change (%) | | 1.3 | 1.9 | 0.7 | 2.1 | 0.8 | 4.1 | 0.3 | 1.4 | 1.1 |

|  |  |  |  | **Weighted summary** | | **Unweighted summary** | |  |  |  |  |
| --- | --- | --- | --- | --- | --- | --- | --- | --- | --- | --- | --- |
|  |  |  | **National U5MR prevalence** | **Absolute** | **Relative** | **Absolute** | **Relative** | **Extreme group comparisons** | | **Prevalence (%) in worst and best groups** | |
| **Country** | **Year** | **Nº of groups** | **%** | **WMDOM** | **WMROM** | **UMDOM** | **UMROM** | **Hi/lo diff** | **Hi/lo ratio** | **Worst** | **Best** |
| Albania | 2008 | 2 | 22 | 0.0 | 0.0 | 0.1 | 0.3 | 0 | 1.01 | 22 | 22 |
|  | 2017 | 2 | 6 | 0.4 | 6.6 | 2.6 | 45.3 | 5 | 7.83 | 6 | 1 |
|  | Absolute annual change (p.p.t.) | | -1.8 | 0.0 | 0.7 | 0.3 | 5.0 | 0.6 | 0.8 | -1.8 | -2.4 |
|  | Relative annual change (%) | | -13.8 | 55.6 | 80.5 | 51.5 | 75.7 | 51.5 | 25.6 | -13.5 | -31.1 |
| Benin | 2001 | 8 | 163 | 9.3 | 5.7 | 14.2 | 8.7 | 44 | 1.31 | 188 | 143 |
|  | 2017 | 8 | 102 | 9.0 | 8.8 | 12.2 | 12.0 | 51 | 1.58 | 139 | 88 |
|  | Absolute annual change (p.p.t.) | | -3.8 | 0.0 | 0.2 | -0.1 | 0.2 | 0.4 | 0.0 | -3.0 | -3.5 |
|  | Relative annual change (%) | | -2.9 | -0.2 | 2.8 | -0.9 | 2.0 | 0.9 | 1.2 | -1.9 | -3.0 |
| Burkina Faso | 2003 | 7 | 193 | 10.8 | 5.6 | 22.7 | 11.7 | 105 | 1.72 | 250 | 145 |
|  | 2010 | 7 | 148 | 18.6 | 12.6 | 32.7 | 22.1 | 118 | 2.59 | 192 | 74 |
|  | Absolute annual change (p.p.t.) | | -6.5 | 1.1 | 1.0 | 1.4 | 1.5 | 1.8 | 0.1 | -8.3 | -10.1 |
|  | Relative annual change (%) | | -3.8 | 8.0 | 12.3 | 5.4 | 9.5 | 1.7 | 6.0 | -3.7 | -9.1 |
| Cameroon | 2011 | 8 | 128 | 34.7 | 27.0 | 32.4 | 25.3 | 93 | 2.18 | 172 | 79 |
|  | 2014 | 8 | 112 | 33.6 | 29.9 | 32.1 | 28.6 | 96 | 2.64 | 154 | 58 |
|  | Absolute annual change (p.p.t.) | | -5.3 | -0.4 | 1.0 | -0.1 | 1.1 | 0.9 | 0.2 | -5.9 | -6.8 |
|  | Relative annual change (%) | | -4.3 | -1.0 | 3.5 | -0.3 | 4.2 | 0.9 | 6.6 | -3.6 | -9.5 |
| Chad | 2004 | 8 | 203 | 27.3 | 13.5 | 38.6 | 19.0 | 131 | 1.92 | 272 | 141 |
|  | 2019 | 8 | 116 | 29.6 | 25.6 | 29.9 | 25.9 | 96 | 2.33 | 168 | 72 |
|  | Absolute annual change (p.p.t.) | | -5.8 | 0.2 | 0.8 | -0.6 | 0.5 | -2.3 | 0.0 | -6.9 | -4.6 |
|  | Relative annual change (%) | | -3.7 | 0.5 | 4.4 | -1.7 | 2.1 | -2.0 | 1.3 | -3.1 | -4.4 |
| Colombia | 2010 | 4 | 22 | 3.2 | 14.6 | 5.0 | 23.2 | 11 | 1.57 | 30 | 19 |
|  | 2015 | 4 | 19 | 5.5 | 29.2 | 7.5 | 40.2 | 24 | 2.50 | 40 | 16 |
|  | Absolute annual change (p.p.t.) | | -0.6 | 0.5 | 2.9 | 0.5 | 3.4 | 2.6 | 0.2 | 2.0 | -0.6 |
|  | Relative annual change (%) | | -2.9 | 11.4 | 14.8 | 8.4 | 11.6 | 16.9 | 9.7 | 5.8 | -3.6 |
| Congo DR | 2007 | 8 | 155 | 15.1 | 9.7 | 21.5 | 13.8 | 91 | 1.78 | 207 | 117 |
|  | 2013 | 8 | 112 | 10.3 | 9.3 | 12.1 | 10.9 | 47 | 1.57 | 130 | 83 |
|  | Absolute annual change (p.p.t.) | | -7.3 | -0.8 | -0.1 | -1.6 | -0.5 | -7.2 | 0.0 | -12.9 | -5.7 |
|  | Relative annual change (%) | | -5.4 | -6.1 | -0.7 | -9.1 | -4.0 | -10.3 | -2.0 | -7.5 | -5.6 |
| Congo Republic | 2011 | 6 | 82 | 8.9 | 10.9 | 11.8 | 14.4 | 33 | 1.44 | 107 | 74 |
|  | 2014 | 6 | 53 | 6.9 | 13.0 | 11.0 | 20.9 | 54 | 2.40 | 92 | 38 |
|  | Absolute annual change (p.p.t.) | | -9.6 | -0.7 | 0.7 | -0.2 | 2.2 | 6.9 | 0.3 | -5.0 | -12.0 |
|  | Relative annual change (%) | | -13.5 | -8.2 | 6.2 | -2.1 | 13.2 | 17.8 | 18.5 | -4.9 | -19.7 |
| Dominican Republic | 2014 | 2 | 35 | 1.8 | 5.2 | 6.1 | 17.2 | 12 | 1.35 | 46 | 34 |
|  | 2019 | 2 | 32 | 3.9 | 12.4 | 12.0 | 37.8 | 24 | 1.81 | 53 | 29 |
|  | Absolute annual change (p.p.t.) | | -0.7 | 0.4 | 1.5 | 1.2 | 4.1 | 2.4 | 0.1 | 1.4 | -1.0 |
|  | Relative annual change (%) | | -2.1 | 16.7 | 19.3 | 14.6 | 17.1 | 14.6 | 6.0 | 2.8 | -3.0 |
| Ethiopia | 2000 | 4 | 188 | 6.5 | 3.5 | 9.0 | 4.8 | 32 | 1.19 | 196 | 165 |
|  | 2019 | 4 | 68 | 9.2 | 13.6 | 12.4 | 18.4 | 34 | 1.81 | 76 | 42 |
|  | Absolute annual change (p.p.t.) | | -6.3 | 0.1 | 0.5 | 0.2 | 0.7 | 0.1 | 0.0 | -6.3 | -6.5 |
|  | Relative annual change (%) | | -5.2 | 1.9 | 7.5 | 1.7 | 7.3 | 0.4 | 2.2 | -4.9 | -6.9 |
| Gabon | 2000 | 8 | 91 | 17.2 | 18.9 | 16.5 | 18.1 | 59 | 2.06 | 114 | 55 |
|  | 2012 | 8 | 63 | 8.8 | 14.0 | 12.3 | 19.5 | 59 | 4.22 | 78 | 18 |
|  | Absolute annual change (p.p.t.) | | -2.3 | -0.7 | -0.4 | -0.4 | 0.1 | 0.0 | 0.2 | -3.1 | -3.1 |
|  | Relative annual change (%) | | -3.0 | -5.4 | -2.5 | -2.4 | 0.6 | 0.0 | 6.1 | -3.2 | -8.8 |
| Gambia | 2013 | 5 | 62 | 4.4 | 7.2 | 5.4 | 8.7 | 17 | 1.34 | 66 | 49 |
|  | 2019 | 5 | 60 | 3.2 | 5.3 | 4.6 | 7.6 | 17 | 1.34 | 66 | 50 |
|  | Absolute annual change (p.p.t.) | | -0.2 | -0.2 | -0.3 | -0.1 | -0.2 | 0.0 | 0.0 | 0.1 | 0.1 |
|  | Relative annual change (%) | | -0.4 | -5.3 | -5.0 | -2.6 | -2.3 | 0.0 | 0.0 | 0.1 | 0.1 |
| Ghana | 2003 | 7 | 110 | 17.8 | 16.3 | 17.1 | 15.6 | 66 | 1.82 | 146 | 80 |
|  | 2017 | 7 | 59 | 18.2 | 30.8 | 17.7 | 30.0 | 69 | 3.17 | 101 | 32 |
|  | Absolute annual change (p.p.t.) | | -3.6 | 0.0 | 1.0 | 0.0 | 1.0 | 0.3 | 0.1 | -3.2 | -3.5 |
|  | Relative annual change (%) | | -4.3 | 0.1 | 4.7 | 0.3 | 4.8 | 0.4 | 4.1 | -2.6 | -6.4 |
| Guinea | 2005 | 5 | 188 | 18.3 | 9.7 | 23.6 | 12.6 | 62 | 1.36 | 233 | 171 |
|  | 2018 | 5 | 108 | 4.3 | 3.9 | 5.2 | 4.8 | 18 | 1.18 | 115 | 97 |
|  | Absolute annual change (p.p.t.) | | -6.1 | -1.1 | -0.4 | -1.4 | -0.6 | -3.4 | 0.0 | -9.1 | -5.6 |
|  | Relative annual change (%) | | -4.1 | -10.6 | -6.7 | -11.0 | -7.2 | -9.2 | -1.1 | -5.3 | -4.2 |
| Guyana | 2009 | 4 | 39 | 5.9 | 14.9 | 5.5 | 14.0 | 17 | 1.64 | 43 | 26 |
|  | 2019 | 4 | 22 | 6.6 | 30.2 | 6.7 | 30.4 | 22 | 2.72 | 35 | 13 |
|  | Absolute annual change (p.p.t.) | | -1.7 | 0.1 | 1.5 | 0.1 | 1.6 | 0.5 | 0.1 | -0.8 | -1.3 |
|  | Relative annual change (%) | | -5.7 | 1.2 | 7.3 | 1.9 | 8.1 | 2.7 | 5.2 | -2.1 | -6.9 |
| Honduras | 2011 | 5 | 30 | 1.9 | 6.4 | 7.8 | 26.3 | 29 | 2.19 | 53 | 24 |
|  | 2019 | 5 | 23 | 1.4 | 6.3 | 6.7 | 29.3 | 18 | 2.66 | 28 | 11 |
|  | Absolute annual change (p.p.t.) | | -0.8 | -0.1 | 0.0 | -0.1 | 0.4 | -1.4 | 0.1 | -3.1 | -1.7 |
|  | Relative annual change (%) | | -3.2 | -3.4 | -0.2 | -1.9 | 1.4 | -5.9 | 2.4 | -7.5 | -9.7 |
| India | 2005 | 16 | 85 | 17.3 | 20.2 | 22.6 | 26.4 | 77 | 4.46 | 99 | 22 |
|  | 2015 | 16 | 52 | 12.2 | 23.4 | 15.2 | 29.1 | 56 | 8.34 | 64 | 8 |
|  | Absolute annual change (p.p.t.) | | -3.3 | -0.5 | 0.3 | -0.7 | 0.3 | -2.0 | 0.4 | -3.5 | -1.5 |
|  | Relative annual change (%) | | -4.8 | -3.4 | 1.5 | -3.9 | 1.0 | -3.1 | 6.5 | -4.3 | -10.1 |
| Kenya | 2003 | 11 | 113 | 45.6 | 40.4 | 40.7 | 36.1 | 201 | 5.28 | 249 | 47 |
|  | 2014 | 11 | 56 | 12.4 | 22.2 | 12.0 | 21.4 | 59 | 2.57 | 96 | 37 |
|  | Absolute annual change (p.p.t.) | | -5.2 | -3.0 | -1.7 | -2.6 | -1.3 | -13.0 | -0.2 | -13.9 | -0.9 |
|  | Relative annual change (%) | | -6.2 | -11.1 | -5.3 | -10.5 | -4.6 | -10.6 | -6.3 | -8.3 | -2.1 |
| Kosovo | 2013 | 2 | 20 | 0.1 | 0.5 | 0.3 | 1.7 | 1 | 1.04 | 20 | 19 |
|  | 2019 | 2 | 15 | 0.1 | 0.8 | 0.4 | 2.4 | 1 | 1.05 | 15 | 14 |
|  | Absolute annual change (p.p.t.) | | -0.8 | 0.0 | 0.0 | 0.0 | 0.1 | 0.0 | 0.0 | -0.8 | -0.8 |
|  | Relative annual change (%) | | -4.4 | 2.6 | 7.3 | 1.2 | 5.8 | 1.2 | 0.2 | -4.4 | -4.6 |
| Kyrgyzstan | 2012 | 3 | 33 | 2.8 | 8.5 | 3.4 | 10.4 | 9 | 1.36 | 35 | 26 |
|  | 2018 | 3 | 22 | 2.5 | 11.4 | 7.4 | 33.0 | 21 | 3.62 | 29 | 8 |
|  | Absolute annual change (p.p.t.) | | -1.8 | 0.0 | 0.5 | 0.7 | 3.8 | 2.0 | 0.4 | -1.0 | -2.9 |
|  | Relative annual change (%) | | -6.3 | -1.6 | 5.1 | 13.6 | 21.3 | 14.6 | 17.7 | -3.0 | -17.6 |
| Lao PDR | 2011 | 3 | 105 | 25.3 | 24.2 | 23.7 | 22.7 | 51 | 1.63 | 133 | 82 |
|  | 2017 | 3 | 49 | 11.1 | 22.5 | 10.2 | 20.7 | 26 | 1.64 | 66 | 40 |
|  | Absolute annual change (p.p.t.) | | -9.2 | -2.4 | -0.3 | -2.3 | -0.3 | -4.2 | 0.0 | -11.1 | -6.9 |
|  | Relative annual change (%) | | -11.8 | -12.8 | -1.2 | -13.1 | -1.5 | -10.7 | 0.2 | -10.9 | -11.1 |
| Malawi | 2000 | 9 | 203 | 18.6 | 9.2 | 25.2 | 12.4 | 94 | 1.69 | 232 | 138 |
|  | 2019 | 9 | 58 | 5.3 | 9.1 | 7.8 | 13.3 | 30 | 1.78 | 68 | 38 |
|  | Absolute annual change (p.p.t.) | | -7.6 | -0.7 | 0.0 | -0.9 | 0.0 | -3.4 | 0.0 | -8.6 | -5.2 |
|  | Relative annual change (%) | | -6.3 | -6.4 | 0.0 | -6.0 | 0.4 | -5.9 | 0.3 | -6.3 | -6.5 |
| Mali | 2001 | 7 | 238 | 9.9 | 4.1 | 16.5 | 6.9 | 68 | 1.34 | 267 | 199 |
|  | 2018 | 7 | 113 | 7.2 | 6.4 | 13.9 | 12.3 | 55 | 1.73 | 131 | 76 |
|  | Absolute annual change (p.p.t.) | | -7.4 | -0.2 | 0.1 | -0.2 | 0.3 | -0.8 | 0.0 | -8.0 | -7.3 |
|  | Relative annual change (%) | | -4.3 | -1.9 | 2.5 | -1.0 | 3.4 | -1.2 | 1.5 | -4.1 | -5.5 |
| Mauritania | 2011 | 4 | 73 | 1.6 | 2.1 | 12.0 | 16.4 | 48 | 2.14 | 90 | 42 |
|  | 2015 | 4 | 53 | 1.3 | 2.4 | 7.8 | 14.7 | 30 | 1.95 | 61 | 31 |
|  | Absolute annual change (p.p.t.) | | -5.1 | -0.1 | 0.1 | -1.1 | -0.4 | -4.5 | 0.0 | -7.1 | -2.6 |
|  | Relative annual change (%) | | -7.9 | -4.9 | 3.2 | -10.4 | -2.7 | -11.2 | -2.3 | -9.1 | -6.9 |
| Moldova | 2005 | 4 | 26 | 4.3 | 16.3 | 9.5 | 36.3 | 29 | 2.47 | 48 | 19 |
|  | 2012 | 4 | 16 | 3.7 | 23.6 | 10.8 | 68.7 | 32 | 9.07 | 35 | 4 |
|  | Absolute annual change (p.p.t.) | | -1.5 | -0.1 | 1.0 | 0.2 | 4.6 | 0.4 | 0.9 | -1.8 | -2.2 |
|  | Relative annual change (%) | | -7.1 | -2.0 | 5.4 | 1.8 | 9.6 | 1.4 | 20.4 | -4.2 | -20.4 |
| Mongolia | 2013 | 3 | 28 | 3.5 | 12.5 | 13.3 | 46.9 | 39 | 2.52 | 65 | 26 |
|  | 2018 | 3 | 18 | 2.9 | 15.9 | 6.2 | 33.9 | 18 | 2.03 | 35 | 17 |
|  | Absolute annual change (p.p.t.) | | -2.0 | -0.1 | 0.7 | -1.4 | -2.6 | -4.4 | -0.1 | -6.2 | -1.8 |
|  | Relative annual change (%) | | -8.5 | -3.9 | 4.9 | -14.2 | -6.3 | -14.9 | -4.2 | -12.0 | -8.1 |
| Mozambique | 2003 | 9 | 178 | 31.9 | 17.9 | 33.9 | 19.0 | 135 | 2.45 | 227 | 93 |
|  | 2015 | 9 | 41 | 8.1 | 19.8 | 9.2 | 22.5 | 24 | 1.88 | 52 | 27 |
|  | Absolute annual change (p.p.t.) | | -11.5 | -2.0 | 0.2 | -2.1 | 0.3 | -9.2 | 0.0 | -14.6 | -5.4 |
|  | Relative annual change (%) | | -11.6 | -10.8 | 0.8 | -10.3 | 1.4 | -13.3 | -2.2 | -11.6 | -9.6 |
| Namibia | 2000 | 6 | 60 | 14.5 | 24.0 | 17.4 | 28.9 | 46 | 2.34 | 81 | 34 |
|  | 2013 | 6 | 59 | 12.1 | 20.6 | 18.1 | 30.7 | 74 | 4.47 | 96 | 21 |
|  | Absolute annual change (p.p.t.) | | -0.1 | -0.2 | -0.3 | 0.1 | 0.1 | 2.2 | 0.2 | 1.2 | -1.0 |
|  | Relative annual change (%) | | -0.2 | -1.4 | -1.2 | 0.3 | 0.5 | 3.7 | 5.1 | 1.3 | -3.6 |
| Nepal | 2011 | 10 | 62 | 8.8 | 14.1 | 10.7 | 17.2 | 47 | 2.28 | 83 | 36 |
|  | 2016 | 10 | 46 | 8.3 | 17.9 | 10.4 | 22.5 | 56 | 2.91 | 85 | 29 |
|  | Absolute annual change (p.p.t.) | | -3.3 | -0.1 | 0.8 | -0.1 | 1.1 | 1.8 | 0.1 | 0.4 | -1.5 |
|  | Relative annual change (%) | | -5.9 | -1.2 | 4.9 | -0.7 | 5.5 | 3.6 | 5.0 | 0.4 | -4.4 |
| Niger | 2006 | 5 | 218 | 16.8 | 7.7 | 40.9 | 18.8 | 119 | 2.10 | 228 | 108 |
|  | 2012 | 5 | 153 | 9.5 | 6.2 | 32.3 | 21.1 | 98 | 2.44 | 167 | 69 |
|  | Absolute annual change (p.p.t.) | | -10.8 | -1.2 | -0.3 | -1.4 | 0.4 | -3.5 | 0.1 | -10.1 | -6.6 |
|  | Relative annual change (%) | | -5.7 | -9.1 | -3.6 | -3.9 | 2.0 | -3.1 | 2.5 | -5.0 | -7.3 |
| Nigeria | 2008 | 6 | 171 | 35.3 | 20.7 | 40.2 | 23.5 | 139 | 2.58 | 227 | 88 |
|  | 2018 | 6 | 129 | 43.3 | 33.6 | 43.8 | 34.0 | 122 | 2.96 | 184 | 62 |
|  | Absolute annual change (p.p.t.) | | -4.2 | 0.8 | 1.3 | 0.4 | 1.0 | -1.7 | 0.0 | -4.2 | -2.6 |
|  | Relative annual change (%) | | -2.8 | 2.0 | 5.0 | 0.9 | 3.8 | -1.3 | 1.4 | -2.0 | -3.4 |
| Pakistan | 2006 | 7 | 93 | 18.0 | 19.3 | 20.8 | 22.2 | 60 | 2.05 | 117 | 57 |
|  | 2017 | 7 | 78 | 15.1 | 19.5 | 18.0 | 23.1 | 59 | 2.15 | 110 | 51 |
|  | Absolute annual change (p.p.t.) | | -1.4 | -0.3 | 0.0 | -0.3 | 0.1 | -0.1 | 0.0 | -0.7 | -0.6 |
|  | Relative annual change (%) | | -1.7 | -1.6 | 0.1 | -1.3 | 0.4 | -0.2 | 0.4 | -0.6 | -1.0 |
| Peru | 2000 | 3 | 60 | 17.5 | 29.0 | 23.6 | 39.1 | 63 | 2.24 | 114 | 51 |
|  | 2020 | 3 | 15 | 2.9 | 19.1 | 11.9 | 77.8 | 36 | 3.65 | 49 | 13 |
|  | Absolute annual change (p.p.t.) | | -2.3 | -0.7 | -0.5 | -0.6 | 1.9 | -1.4 | 0.1 | -3.3 | -1.9 |
|  | Relative annual change (%) | | -6.6 | -8.5 | -2.1 | -3.3 | 3.5 | -2.8 | 2.5 | -4.2 | -6.5 |
| Philippines | 2003 | 10 | 42 | 10.5 | 25.3 | 13.2 | 31.7 | 48 | 2.60 | 79 | 30 |
|  | 2017 | 10 | 28 | 7.9 | 28.4 | 8.4 | 30.0 | 43 | 3.47 | 61 | 18 |
|  | Absolute annual change (p.p.t.) | | -1.0 | -0.2 | 0.2 | -0.3 | -0.1 | -0.4 | 0.1 | -1.3 | -0.9 |
|  | Relative annual change (%) | | -2.8 | -2.0 | 0.8 | -3.2 | -0.4 | -0.8 | 2.1 | -1.8 | -3.8 |
| Sao Tome and Principe | 2014 | 2 | 43 | 1.4 | 3.4 | 2.3 | 5.4 | 5 | 1.12 | 43 | 39 |
|  | 2019 | 2 | 17 | 1.8 | 10.4 | 4.8 | 27.4 | 10 | 1.58 | 26 | 16 |
|  | Absolute annual change (p.p.t.) | | -5.1 | 0.1 | 1.4 | 0.5 | 4.4 | 1.0 | 0.1 | -3.5 | -4.5 |
|  | Relative annual change (%) | | -16.5 | 4.8 | 25.4 | 15.4 | 38.2 | 15.4 | 7.1 | -9.8 | -15.8 |
| Senegal | 2005 | 6 | 135 | 13.8 | 10.2 | 22.9 | 17.0 | 95 | 1.90 | 200 | 105 |
|  | 2019 | 6 | 48 | 4.9 | 10.0 | 8.5 | 17.6 | 37 | 1.97 | 75 | 38 |
|  | Absolute annual change (p.p.t.) | | -6.2 | -0.6 | 0.0 | -1.0 | 0.0 | -4.1 | 0.0 | -9.0 | -4.8 |
|  | Relative annual change (%) | | -7.1 | -7.2 | -0.2 | -6.8 | 0.3 | -6.5 | 0.3 | -6.8 | -7.1 |
| Sierra Leone | 2008 | 7 | 168 | 9.4 | 5.6 | 20.7 | 12.3 | 70 | 1.65 | 177 | 108 |
|  | 2019 | 7 | 124 | 10.3 | 8.3 | 16.8 | 13.5 | 57 | 1.76 | 132 | 75 |
|  | Absolute annual change (p.p.t.) | | -4.0 | 0.1 | 0.2 | -0.3 | 0.1 | -1.1 | 0.0 | -4.1 | -3.0 |
|  | Relative annual change (%) | | -2.7 | 0.8 | 3.6 | -1.9 | 0.9 | -1.8 | 0.6 | -2.7 | -3.3 |
| Tajikistan | 2012 | 2 | 48 | 1.8 | 3.8 | 2.9 | 5.9 | 6 | 1.13 | 49 | 43 |
|  | 2017 | 2 | 33 | 1.6 | 4.9 | 1.9 | 5.6 | 4 | 1.12 | 34 | 30 |
|  | Absolute annual change (p.p.t.) | | -3.0 | 0.0 | 0.2 | -0.2 | -0.1 | -0.4 | 0.0 | -3.0 | -2.6 |
|  | Relative annual change (%) | | -7.1 | -2.2 | 5.3 | -8.1 | -1.0 | -8.1 | -0.1 | -7.1 | -7.0 |
| Timor-Leste | 2009 | 2 | 80 | 3.8 | 4.7 | 9.3 | 11.6 | 19 | 1.24 | 97 | 78 |
|  | 2016 | 2 | 41 | 4.3 | 10.6 | 19.9 | 48.9 | 40 | 2.04 | 78 | 38 |
|  | Absolute annual change (p.p.t.) | | -5.7 | 0.1 | 0.8 | 1.5 | 5.3 | 3.0 | 0.1 | -2.7 | -5.8 |
|  | Relative annual change (%) | | -9.3 | 1.9 | 12.3 | 11.4 | 22.9 | 11.4 | 7.4 | -3.1 | -9.8 |
| Togo | 2013 | 7 | 94 | 17.6 | 18.8 | 13.9 | 14.8 | 47 | 1.61 | 124 | 77 |
|  | 2017 | 7 | 75 | 5.2 | 6.9 | 8.2 | 10.9 | 26 | 1.48 | 82 | 55 |
|  | Absolute annual change (p.p.t.) | | -4.8 | -3.1 | -3.0 | -1.4 | -1.0 | -5.2 | 0.0 | -10.6 | -5.5 |
|  | Relative annual change (%) | | -5.6 | -26.4 | -22.0 | -12.5 | -7.3 | -13.4 | -2.1 | -10.0 | -8.0 |
| Turkey | 2003 | 2 | 47 | 13.8 | 29.5 | 31.8 | 68.1 | 64 | 2.59 | 104 | 40 |
|  | 2013 | 2 | 20 | 6.0 | 29.9 | 8.7 | 43.6 | 17 | 2.05 | 34 | 17 |
|  | Absolute annual change (p.p.t.) | | -2.7 | -0.8 | 0.0 | -2.3 | -2.5 | -4.6 | -0.1 | -7.0 | -2.3 |
|  | Relative annual change (%) | | -8.1 | -8.0 | 0.2 | -12.1 | -4.4 | -12.1 | -2.3 | -10.5 | -8.4 |
| Turkmenistan | 2015 | 3 | 33 | 2.3 | 6.9 | 5.9 | 17.8 | 11 | 1.48 | 34 | 23 |
|  | 2019 | 3 | 36 | 2.8 | 7.8 | 9.6 | 26.7 | 23 | 2.55 | 37 | 15 |
|  | Absolute annual change (p.p.t.) | | 0.7 | 0.1 | 0.2 | 0.9 | 2.2 | 2.9 | 0.3 | 0.8 | -2.1 |
|  | Relative annual change (%) | | 2.1 | 5.3 | 3.1 | 13.0 | 10.7 | 19.5 | 14.5 | 2.2 | -10.8 |
| Uganda | 2006 | 7 | 144 | 30.4 | 21.1 | 32.0 | 22.3 | 82 | 1.75 | 191 | 109 |
|  | 2016 | 7 | 73 | 7.0 | 9.6 | 8.0 | 10.9 | 26 | 1.45 | 84 | 57 |
|  | Absolute annual change (p.p.t.) | | -7.1 | -2.3 | -1.2 | -2.4 | -1.1 | -5.6 | 0.0 | -10.7 | -5.2 |
|  | Relative annual change (%) | | -6.5 | -13.7 | -7.6 | -13.0 | -6.9 | -10.8 | -1.8 | -7.9 | -6.2 |
| Vietnam | 2002 | 2 | 33 | 8.6 | 26.1 | 13.8 | 42.1 | 28 | 2.03 | 55 | 27 |
|  | 2013 | 2 | 19 | 11.8 | 61.9 | 18.1 | 94.8 | 36 | 3.88 | 49 | 13 |
|  | Absolute annual change (p.p.t.) | | -1.2 | 0.3 | 3.3 | 0.4 | 4.8 | 0.8 | 0.2 | -0.5 | -1.3 |
|  | Relative annual change (%) | | -4.8 | 3.0 | 8.2 | 2.5 | 7.7 | 2.5 | 6.1 | -1.0 | -6.6 |
| Zambia | 2001 | 8 | 168 | 14.9 | 8.9 | 20.3 | 12.1 | 74 | 1.66 | 185 | 111 |
|  | 2013 | 8 | 81 | 8.6 | 10.6 | 13.3 | 16.6 | 46 | 1.96 | 94 | 48 |
|  | Absolute annual change (p.p.t.) | | -7.3 | -0.5 | 0.1 | -0.6 | 0.4 | -2.3 | 0.0 | -7.6 | -5.3 |
|  | Relative annual change (%) | | -5.9 | -4.5 | 1.5 | -3.4 | 2.6 | -3.9 | 1.4 | -5.5 | -6.8 |
| Zimbabwe | 2009 | 3 | 86 | 9.7 | 11.3 | 15.6 | 18.1 | 31 | 1.52 | 91 | 60 |
|  | 2015 | 3 | 82 | 3.2 | 3.9 | 6.2 | 7.6 | 13 | 1.18 | 84 | 71 |
|  | Absolute annual change (p.p.t.) | | -0.6 | -1.1 | -1.2 | -1.6 | -1.8 | -3.1 | -0.1 | -1.2 | 1.8 |
|  | Relative annual change (%) | | -0.7 | -16.7 | -16.1 | -14.1 | -13.5 | -13.9 | -4.1 | -1.4 | 2.8 |
