# Supplementary material for: Time trends in ethnic inequalities in child health and nutrition: analysis of 59 low and middle-income countries
Source: Int J Equity Health. 2023 Apr 28;22:76. doi: 10.1186/s12939-023-01888-5 (PMC10148503; doi:10.1186/s12939-023-01888-5)
Supplement: Supplementary file 3 — Additional file 3. Title: Values of the indices in the first and last surveys, per country and indicator: stunting, U5MR and CCI. Description: Table with values of the summary indices for the first and last surveys as well as for annual changes per country. [file 12939_2023_1888_MOESM3_ESM.docx]

| **ISO code** | **Country** | **Ethnicity data** | | **Survey dates** | | | **Mean sample size (2 surveys)** | | |
| --- | --- | --- | --- | --- | --- | --- | --- | --- | --- |
|  |  | **Variable** | **Groups** | **First survey** | **Last survey** | **Interval (years)** | **Stunting** | **U5MR** | **CCI** |
| ALB | Albania | Ethnicity | 2 | 2002 | 2017 | 15 | 1,958 | 14,447 | 7,994 |
| BLZ | Belize | Ethnicity | 5 | 2006 | 2015 | 9 | 1,644 | - | 6,215 |
| BEN | Benin | Ethnicity | 8 | 2001 | 2017 | 16 | 10,009 | 42,603 | 26,459 |
| BFA | Burkina Faso | Ethnicity | 7 | 2003 | 2010 | 7 | 6,430 | 48,805 | 22,345 |
| CMR | Cameroon | Ethnicity | 8 | 2006 | 2014 | 8 | 5,923 | 34,257 | 21,037 |
| CAF | Central African Republic | Ethnicity | 9 | 2006 | 2018 | 12 | 9,259 | - | 19,804 |
| TCD | Chad | Ethnicity | 8 | 2004 | 2019 | 15 | 10,919 | 54,319 | 22,195 |
| COL | Colombia | Ethnicity | 4 | 2010 | 2015 | 5 | - | 76,996 | - |
| COD | Congo DR | Ethnicity | 8 | 2007 | 2013 | 6 | 5,764 | 44,365 | 30,485 |
| COG | Congo Republic | Ethnicity | 6 | 2011 | 2014 | 3 | 6,533 | 31,794 | 18,458 |
| CRI | Costa Rica | Ethnicity | 3 | 2011 | 2018 | 7 | - | - | 7,105 |
| CIV | Côte d'Ivoire | Ethnicity | 5 | 2006 | 2016 | 10 | 7,868 | - | 15,625 |
| DOM | Dominican Republic | Language | 2 | 2014 | 2019 | 5 | - | 50,079 | 30,854 |
| ECU | Ecuador | Ethnicity | 3 | 2004 | 2012 | 8 | 5,775 | - | - |
| ETH | Ethiopia | Language | 4 | 2000 | 2019 | 19 | 7,231 | 38,781 | 26,776 |
| GAB | Gabon | Ethnicity | 8 | 2000 | 2012 | 12 | 3,271 | 18,794 | 11,354 |
| GMB | Gambia | Ethnicity | 5 | 2005 | 2019 | 14 | 6,905 | 31,758 | 19,787 |
| GEO | Georgia | Ethnicity | 4 | 2005 | 2018 | 13 | 1,945 | - | - |
| GHA | Ghana | Ethnicity | 7 | 2003 | 2017 | 14 | 4,636 | 23,166 | 12,808 |
| GTM | Guatemala | Ethnicity | 2 | 2002 | 2014 | 12 | 9,453 | - | - |
| GIN | Guinea | Ethnicity | 5 | 2005 | 2018 | 13 | 4,093 | 27,628 | 15,371 |
| GNB | Guinea-Bissau | Ethnicity | 7 | 2006 | 2018 | 12 | 6,124 | - | 12,355 |
| GUY | Guyana | Ethnicity | 4 | 2006 | 2019 | 13 | 2,347 | 11,156 | 5,843 |
| HND | Honduras | Ethnicity | 5 | 2011 | 2019 | 8 | 9,083 | 44,055 | 26,989 |
| IND | India | Ethnicity | 16 | 2005 | 2015 | 10 | 137,074 | 786,189 | 477,464 |
| KAZ | Kazakhstan | Ethnicity | 3 | 2006 | 2015 | 9 | 4,847 | - | - |
| KEN | Kenya | Ethnicity | 11 | 2003 | 2014 | 11 | 9,557 | 42,726 | 24,839 |
| XKX | Kosovo | Ethnicity | 2 | 2013 | 2019 | 6 | 1,367 | 8,767 | - |
| KGZ | Kyrgyzstan | Language | 3 | 2012 | 2018 | 6 | 3,973 | 14,483 | 9,053 |
| LAO | Lao PDR | Ethnicity | 3 | 2006 | 2017 | 11 | 8,650 | 55,483 | 27,685 |
| MWI | Malawi | Ethnicity | 9 | 2000 | 2019 | 19 | 11,708 | 61,732 | 40,236 |
| MLI | Mali | Ethnicity | 7 | 2001 | 2018 | 17 | 11,950 | 44,551 | 30,562 |
| MRT | Mauritania | Language | 4 | 2007 | 2015 | 8 | 8,902 | 35,577 | 18,884 |
| MDA | Moldova | Ethnicity | 4 | 2005 | 2012 | 7 | 1,504 | 8,891 | 6,139 |
| MNG | Mongolia | Ethnicity | 3 | 2005 | 2018 | 13 | 4,675 | 23,692 | 12,109 |
| MOZ | Mozambique | Ethnicity | 9 | 2003 | 2011 | 8 | 8,771 | 27,248 | 20,487 |
| NAM | Namibia | Language | 6 | 2000 | 2013 | 13 | 2,843 | 17,507 | 11,409 |
| NPL | Nepal | Ethnicity | 10 | 2011 | 2016 | 5 | 2,352 | 26,322 | 17,135 |
| NER | Niger | Language | 5 | 2006 | 2012 | 6 | 4,333 | 39,281 | 24,516 |
| NGA | Nigeria | Ethnicity | 6 | 2008 | 2018 | 10 | 19,039 | 117,014 | 64,768 |
| MKD | North Macedonia | Ethnicity | 3 | 2005 | 2018 | 13 | 2,297 | - | 4,588 |
| PAK | Pakistan | Language | 7 | 2006 | 2017 | 11 | 3,684 | 46,594 | 26,570 |
| PER | Peru | Ethnicity | 3 | 2000 | 2020 | 20 | 12,072 | 45,242 | 25,625 |
| PHL | Philippines | Ethnicity | 10 | 2003 | 2017 | 14 | - | 34,471 | 20,359 |
| STP | Sao Tome and Principe | Language | 2 | 2014 | 2019 | 5 | 1,880 | 7,485 | 4,220 |
| SEN | Senegal | Ethnicity | 6 | 2005 | 2019 | 14 | 5,901 | 29,080 | 18,609 |
| SRB | Serbia | Ethnicity | 4 | 2005 | 2019 | 14 | 2,431 | - | - |
| SLE | Sierra Leone | Ethnicity | 7 | 2008 | 2019 | 11 | 5,494 | 37,786 | 21,060 |
| SUR | Suriname | Ethnicity | 6 | 2006 | 2018 | 12 | 2,675 | - | - |
| TJK | Tajikistan | Language | 2 | 2005 | 2017 | 12 | 3,650 | 20,962 | 10,108 |
| THA | Thailand | Language | 2 | 2005 | 2019 | 14 | 10,966 | - | - |
| TLS | Timor-Leste | Language | 2 | 2009 | 2016 | 7 | 6,854 | 32,340 | 19,646 |
| TGO | Togo | Ethnicity | 7 | 2006 | 2017 | 11 | 4,178 | 22,332 | 15,288 |
| TUR | Türkiye | Language | 2 | 2003 | 2013 | 10 | 3,418 | 20,129 | - |
| TKM | Turkmenistan | Language | 3 | 2006 | 2019 | 13 | 2,346 | 13,175 | 7,010 |
| UGA | Uganda | Language | 7 | 2006 | 2016 | 10 | 2,957 | 38,868 | 25,879 |
| VNM | Vietnam | Ethnicity | 2 | 2002 | 2013 | 11 | - | 14,927 | 10,588 |
| ZMB | Zambia | Language | 8 | 2001 | 2013 | 12 | 7,411 | 31,428 | 21,409 |
| ZWE | Zimbabwe | Language | 3 | 2009 | 2019 | 10 | 5,700 | 22,254 | - |
