# Supplementary material for: Time trends in ethnic inequalities in child health and nutrition: analysis of 59 low and middle-income countries
Source: Int J Equity Health. 2023 Apr 28;22:76. doi: 10.1186/s12939-023-01888-5 (PMC10148503; doi:10.1186/s12939-023-01888-5)

Supplementary figures 1. Stunting prevalence by ethnic groups in the first and last surveys. Results for selected countries. The numbers in the black rectangles show the average proportion of the samples for each ethnic groups in the two surveys. The numbers at the bottom of the bars show the ratio between the rate in a particular ethnic group and the national rate for that point in time (* statistically significant in relation to the respective national average).

Albania

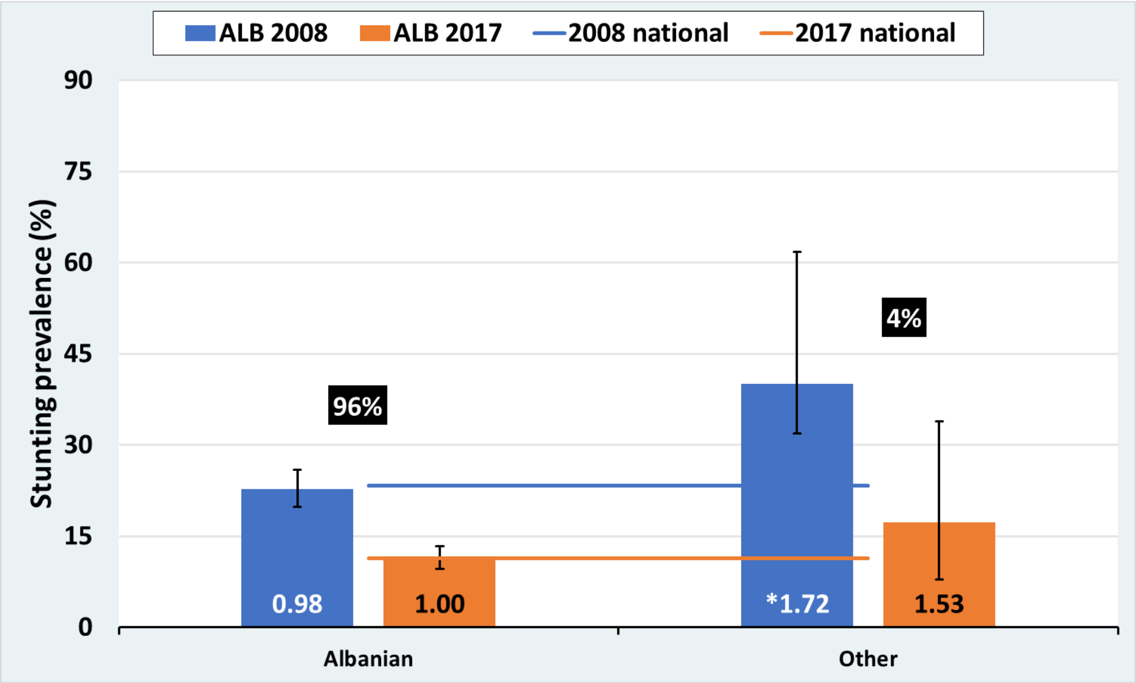

Belize

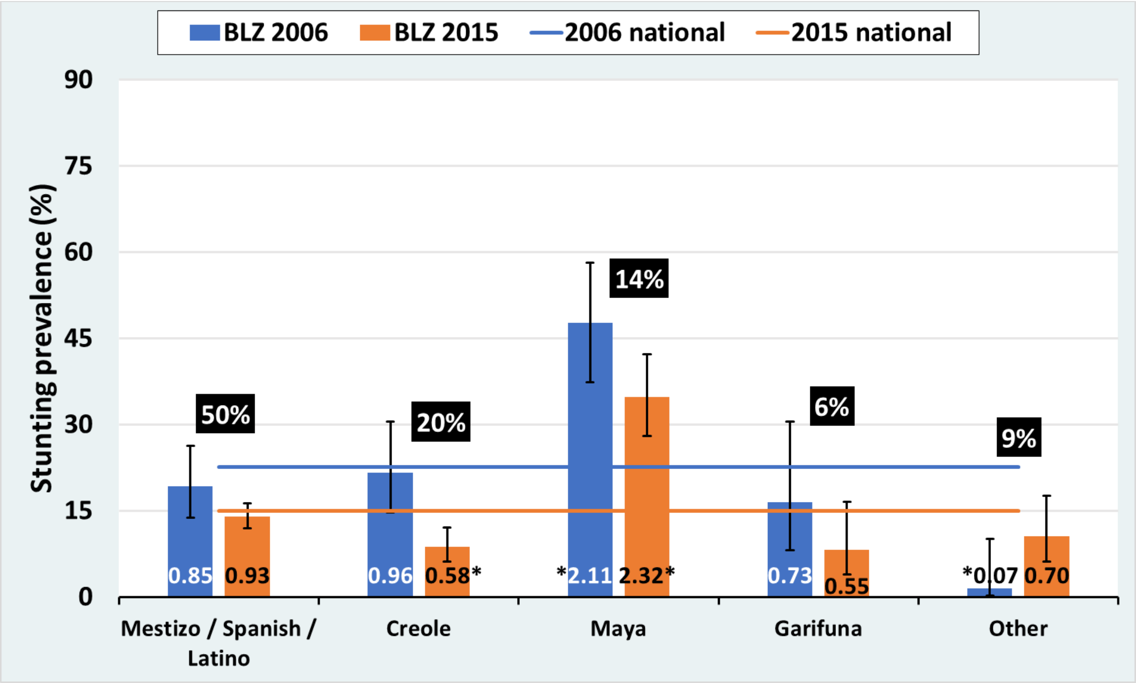

Benin

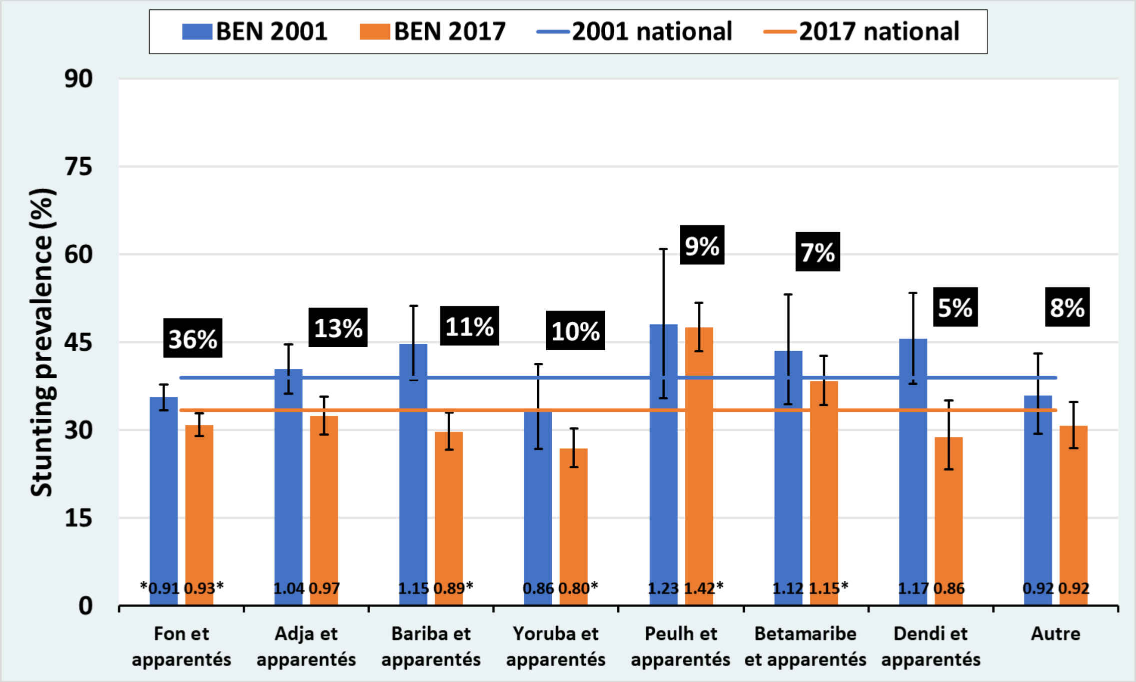

Burkina Faso

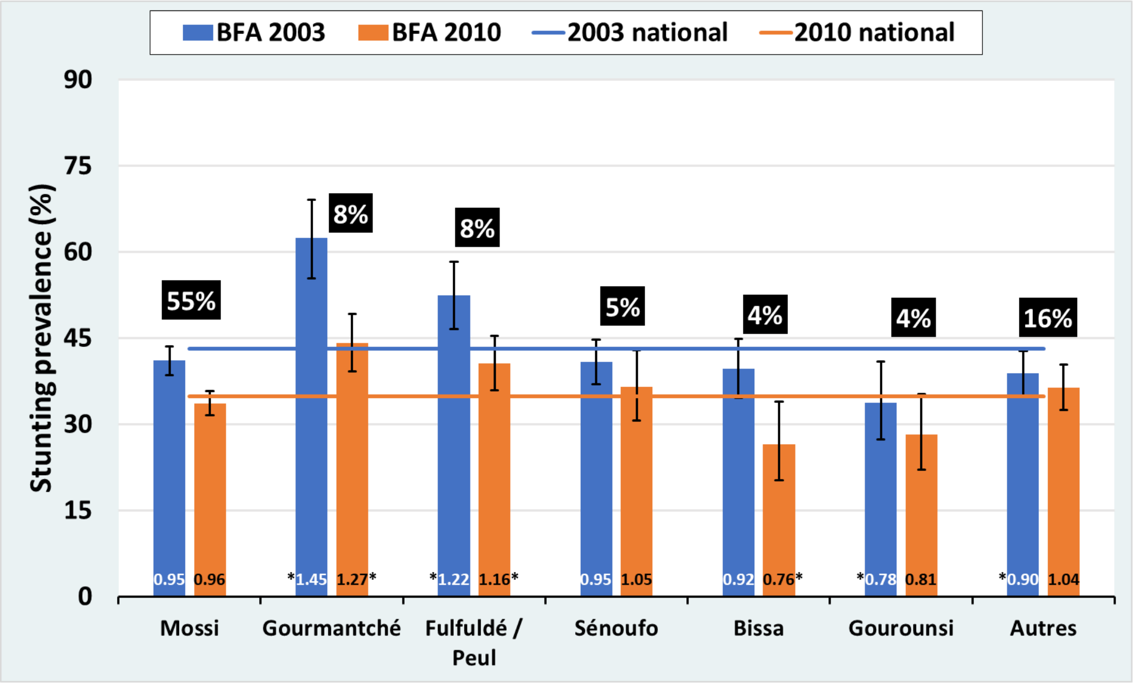

Cameroon

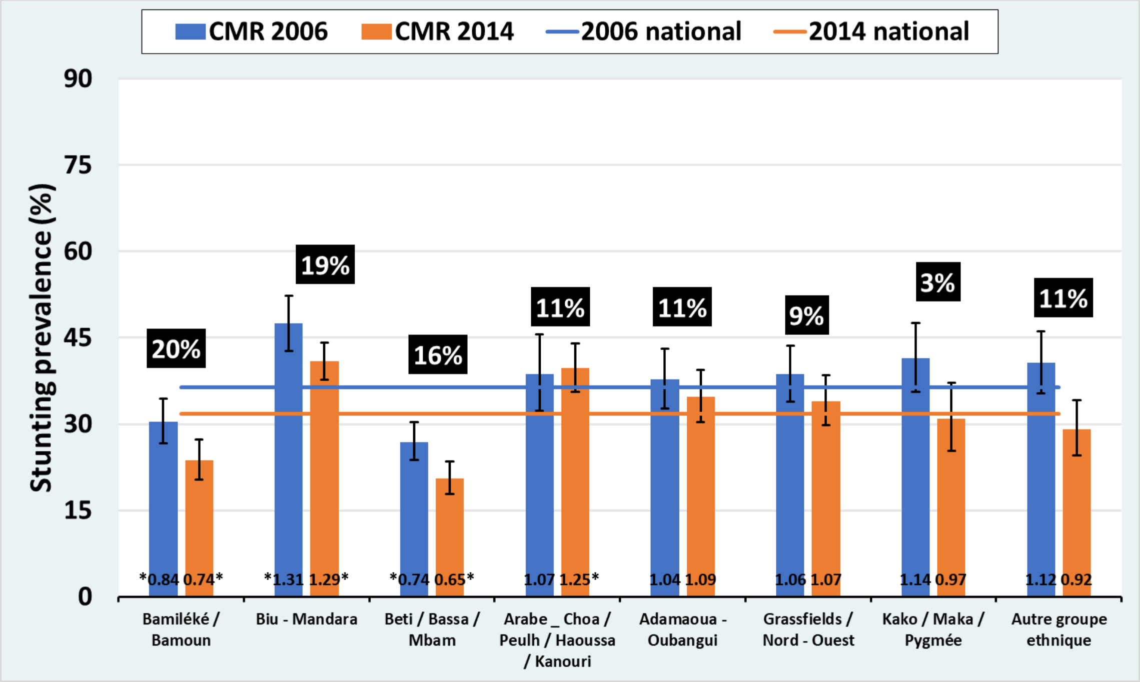

Central African Republic

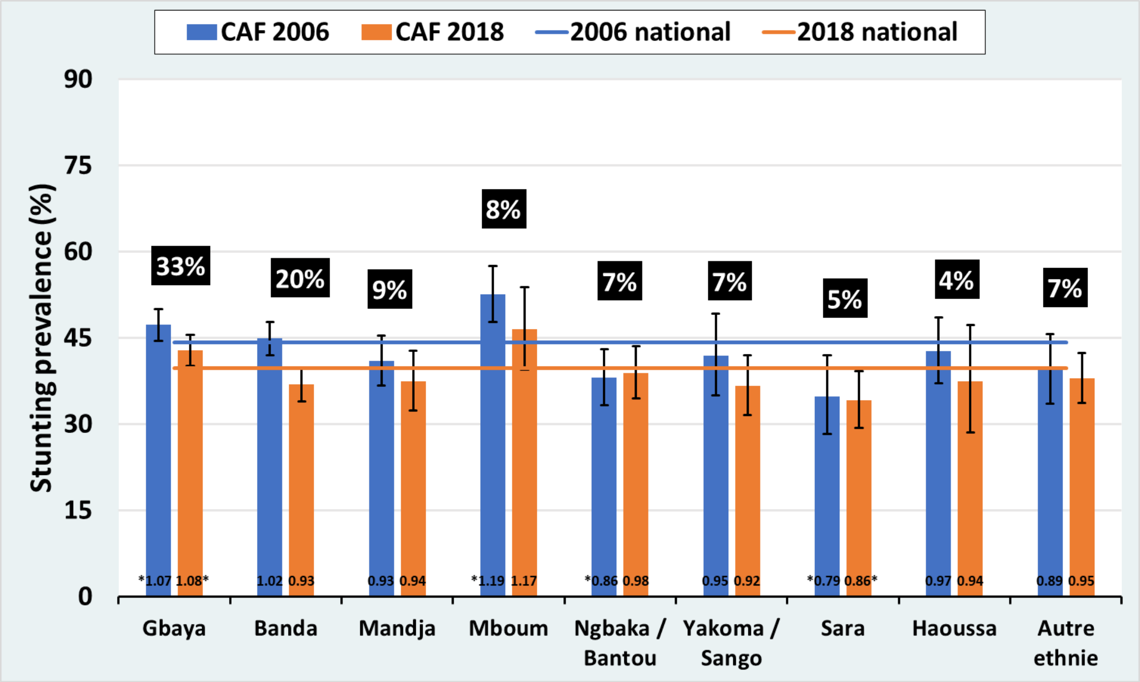

Chad

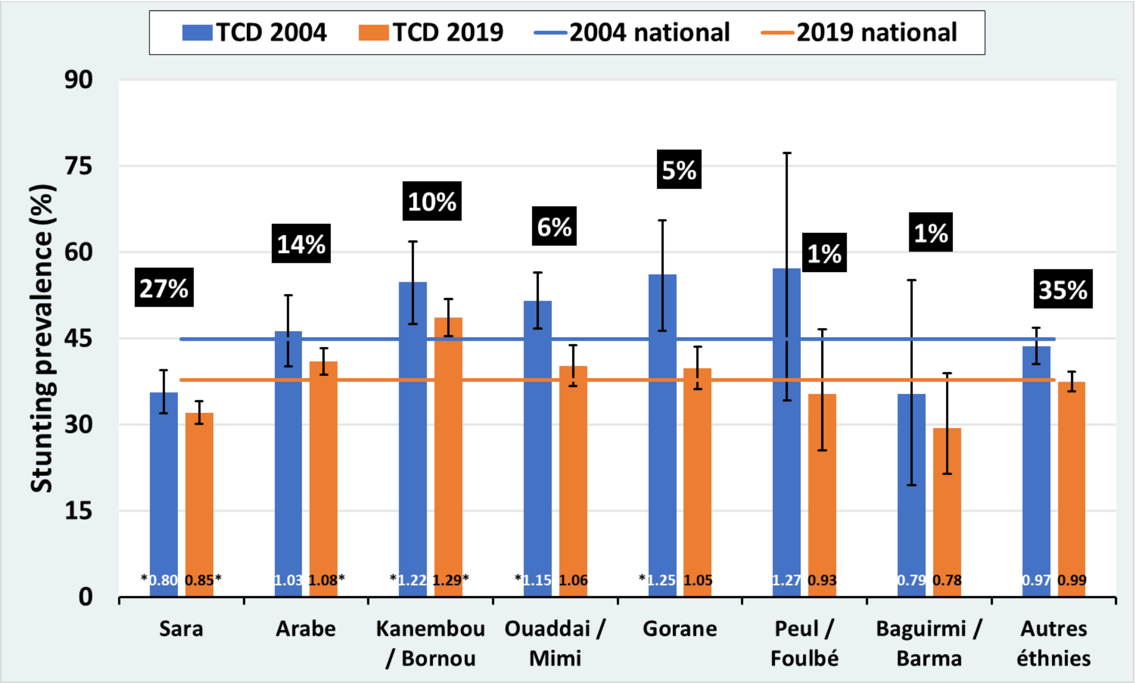

Congo DR

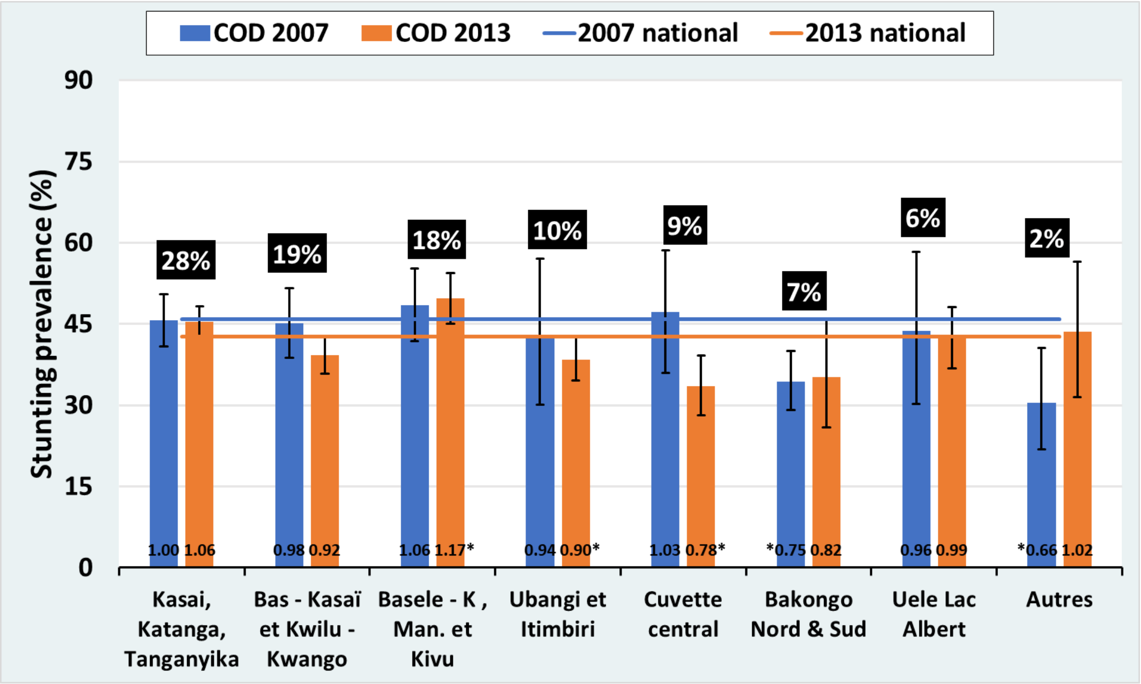

Congo Republic

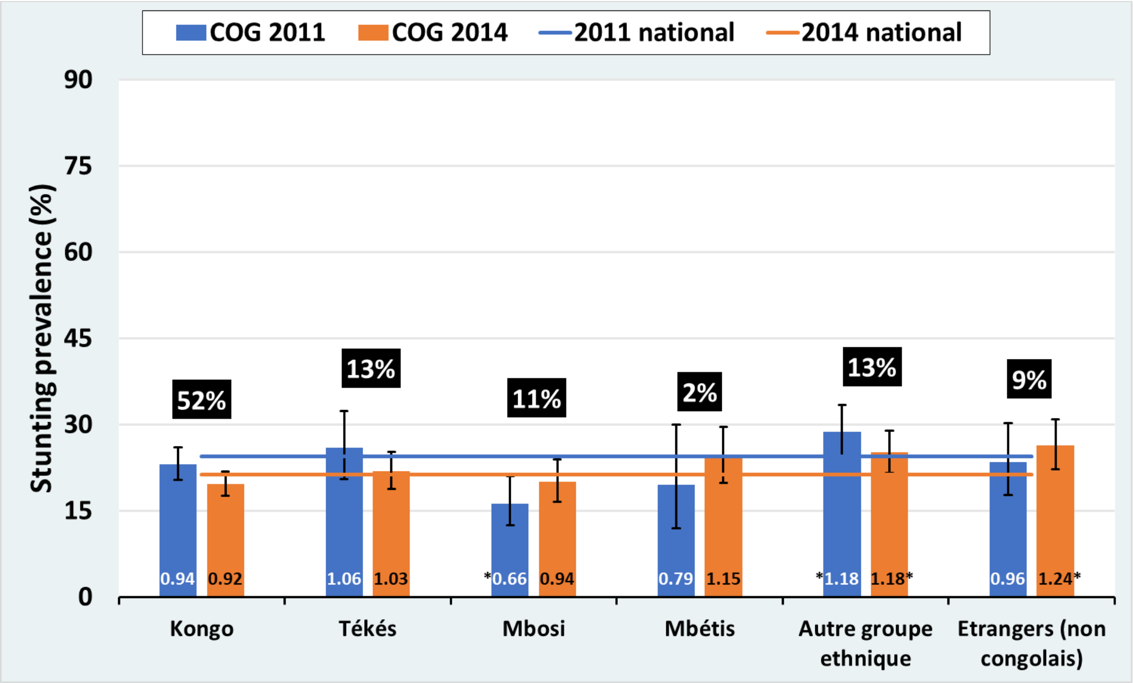

Côte d'Ivoire

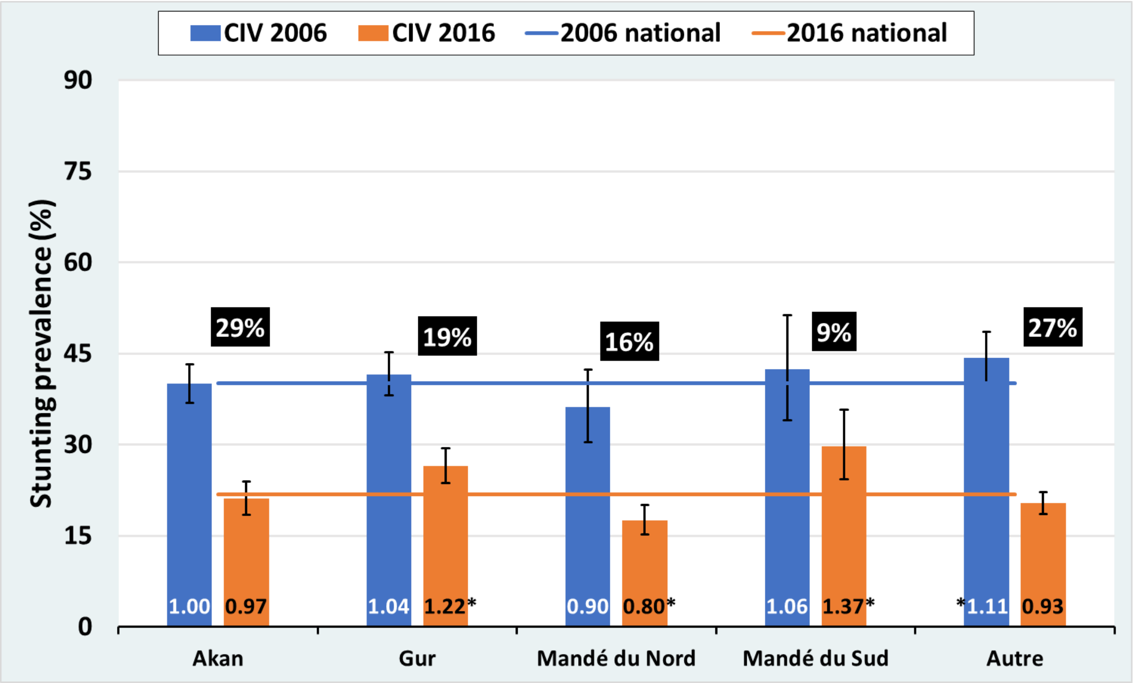

Ecuador

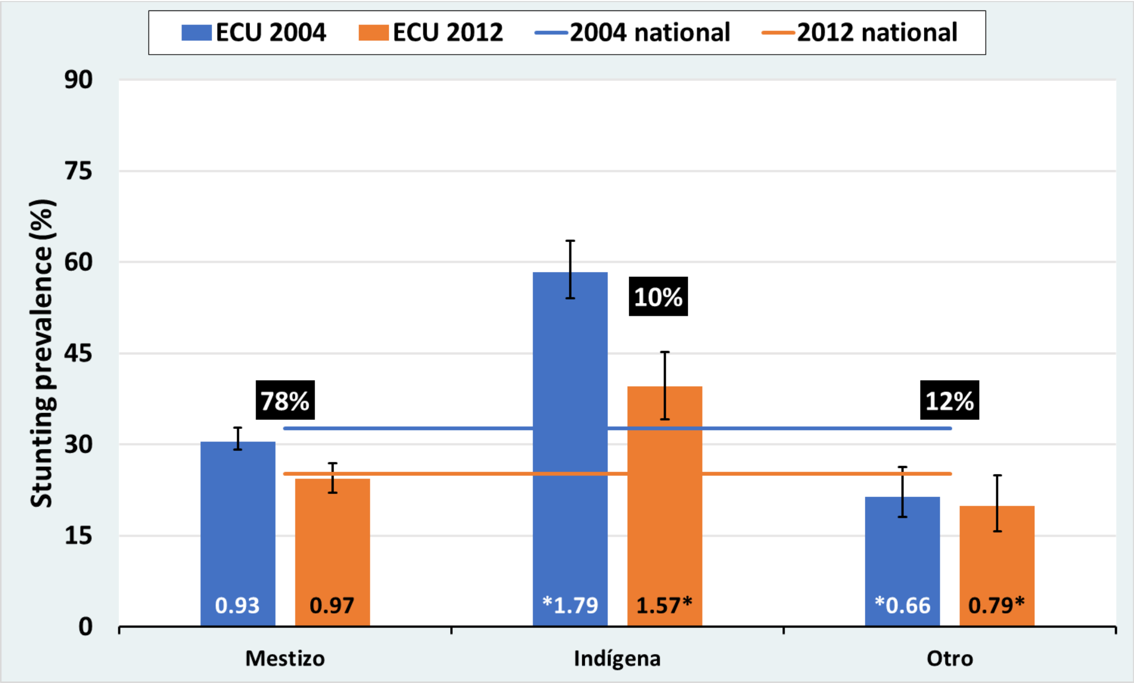

Ethiopia

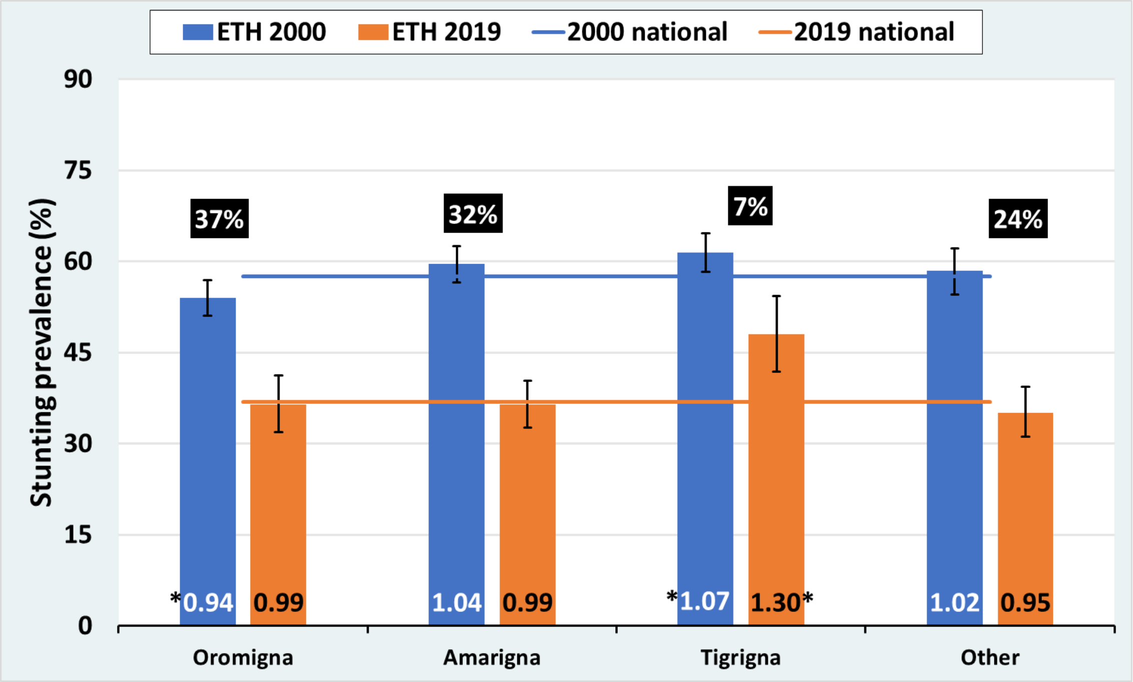

Gabon

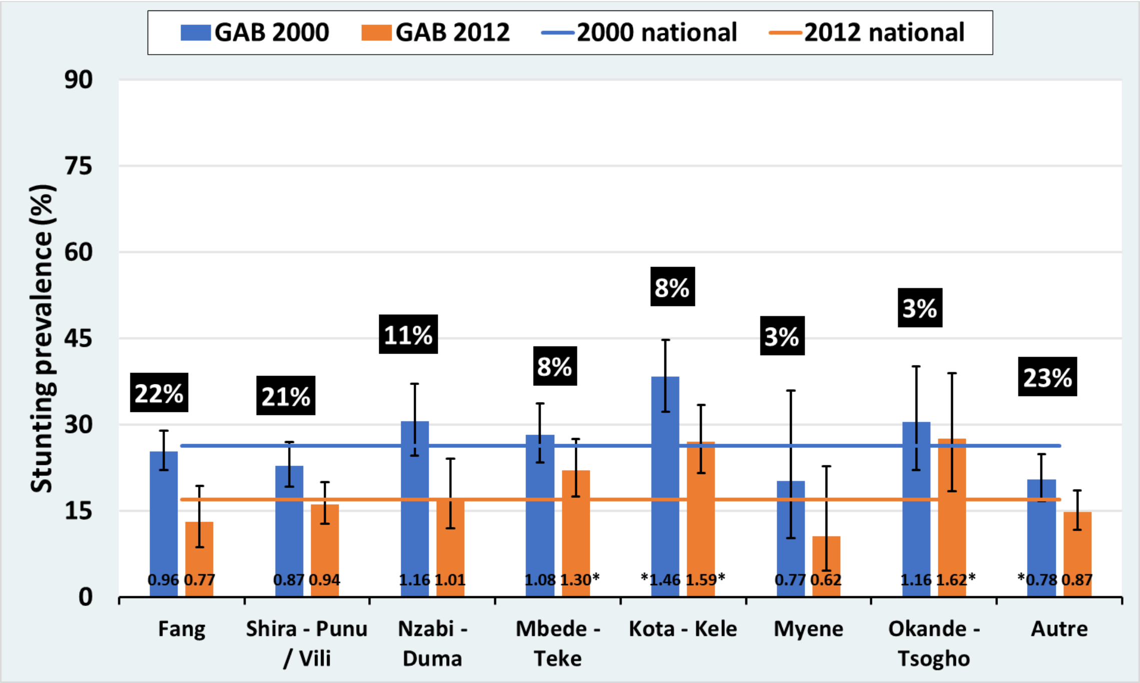

Gambia

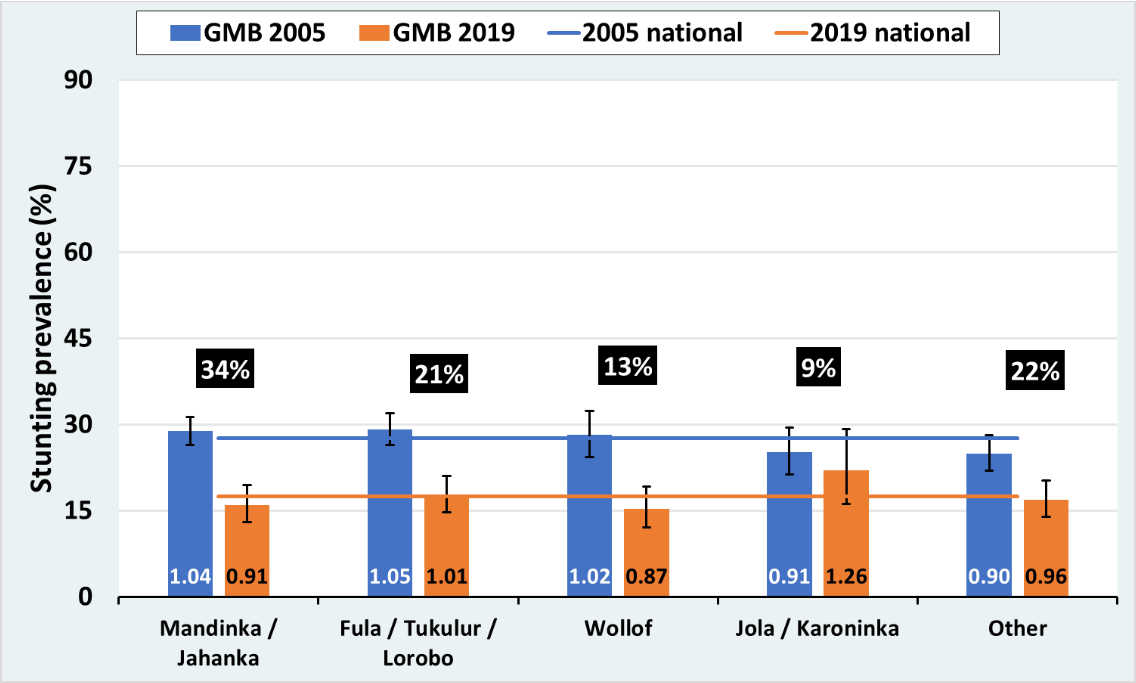

Georgia

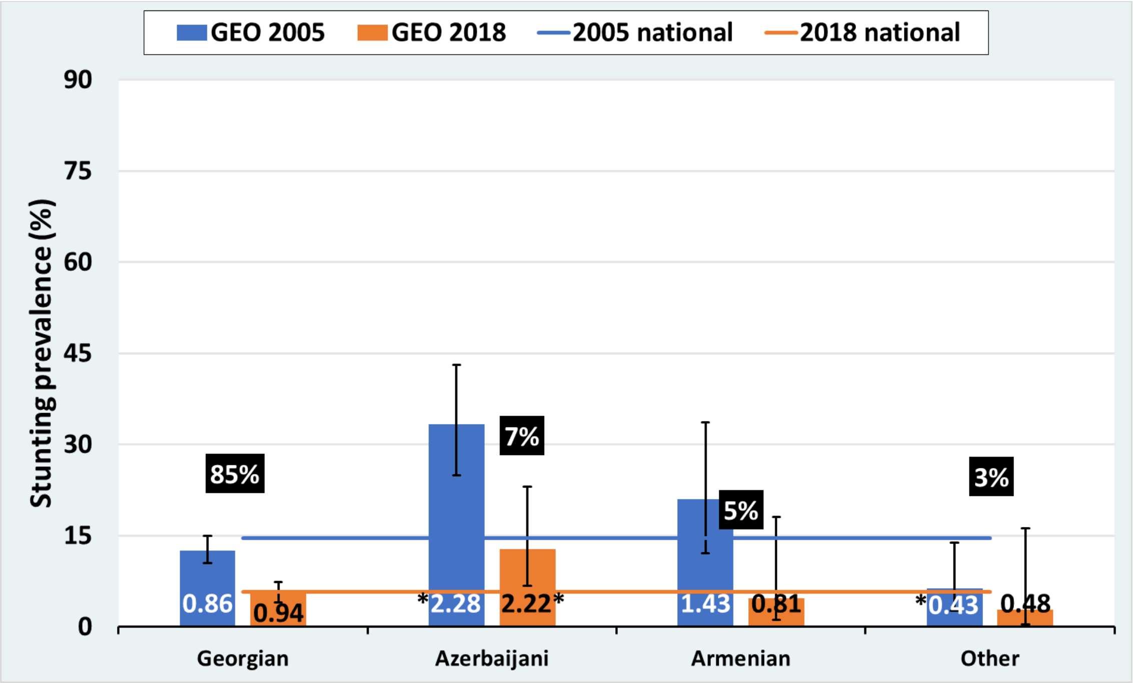

Ghana

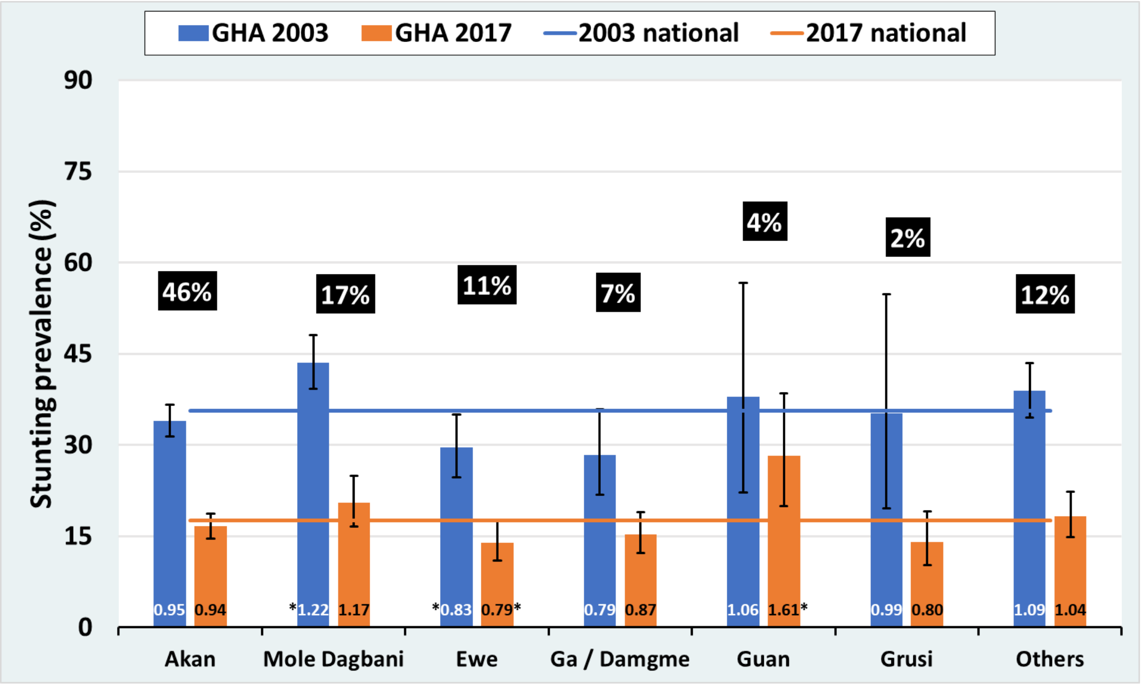

Guatemala

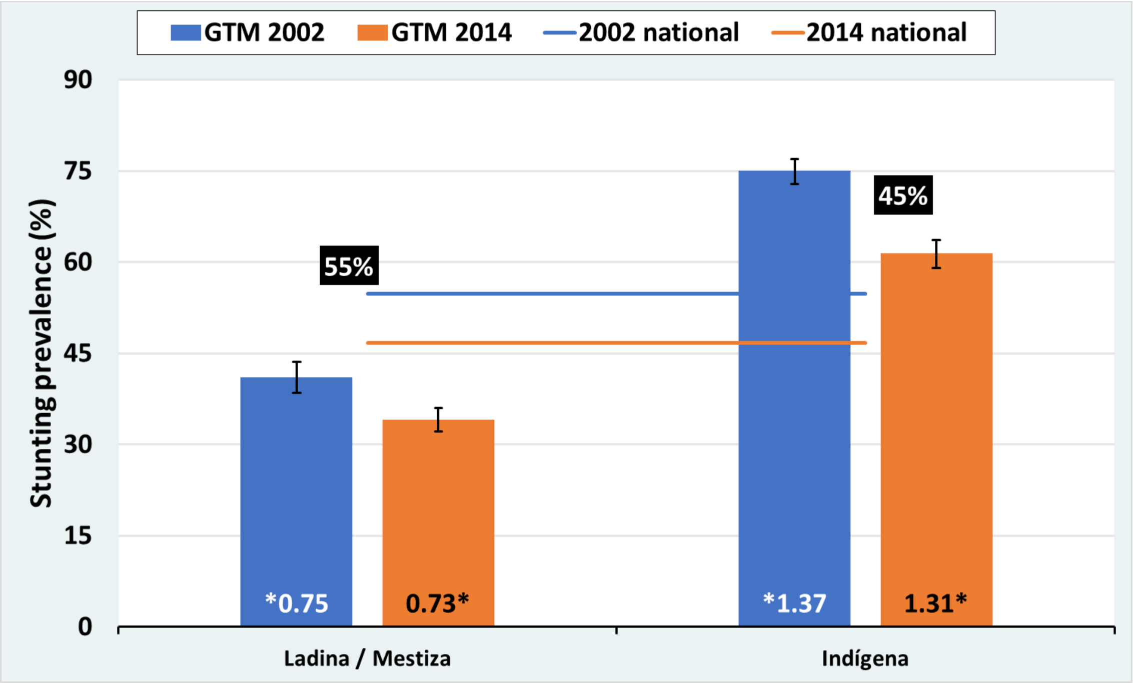

Guinea

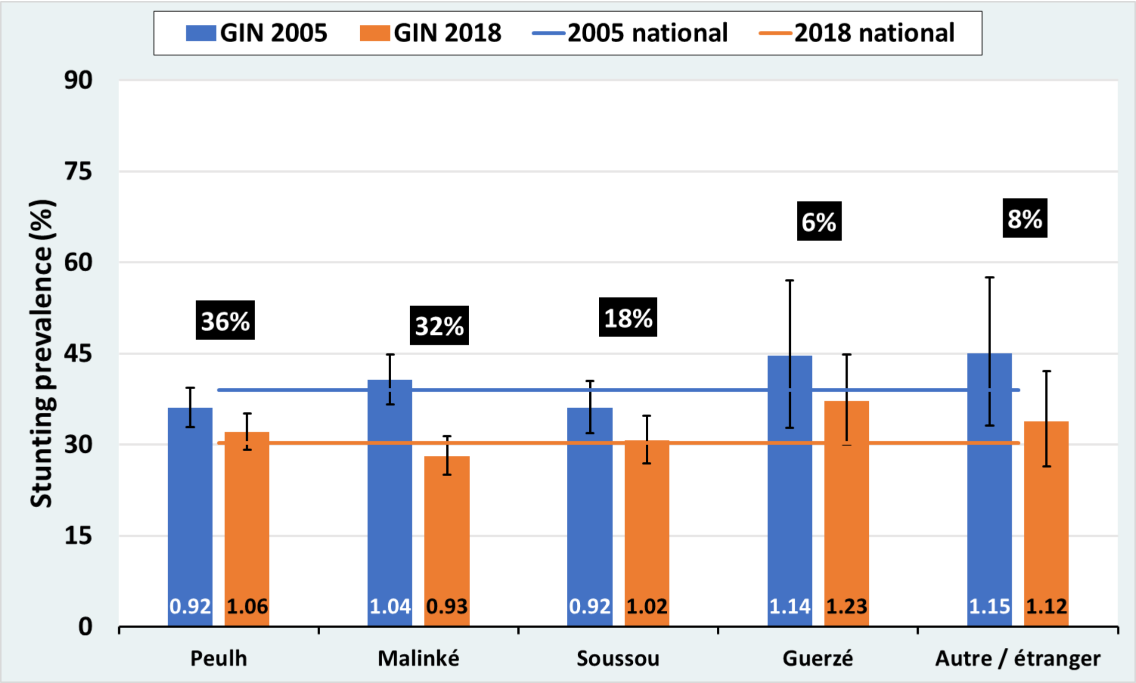

Guinea-Bissau

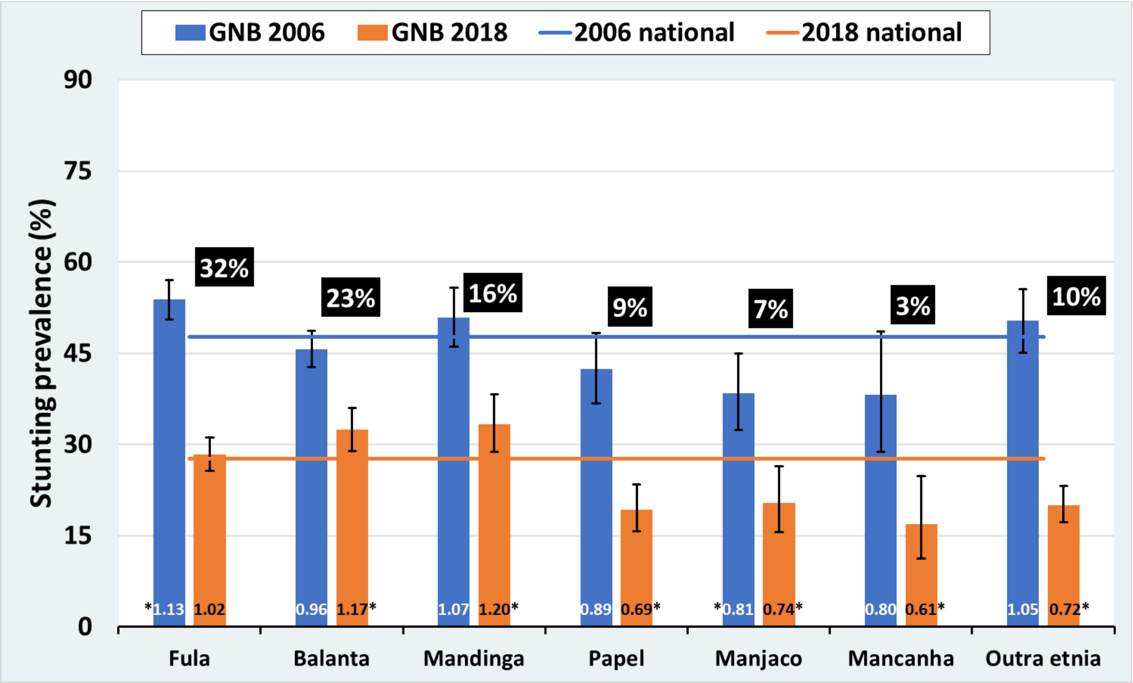

Guyana

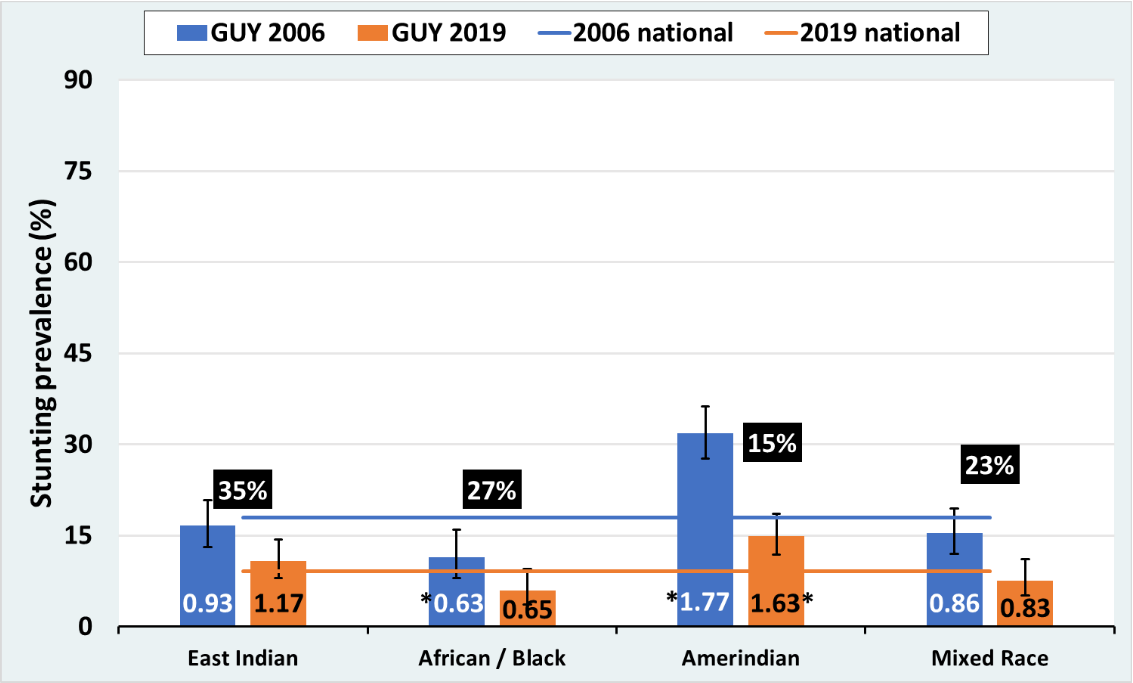

Honduras

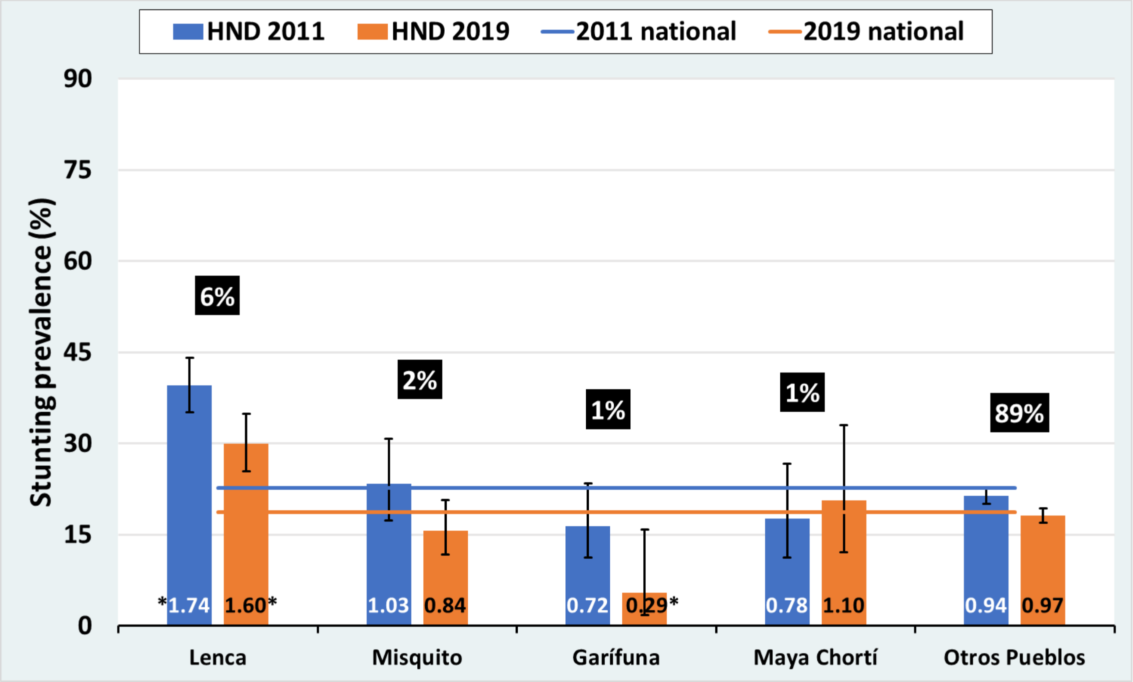

India

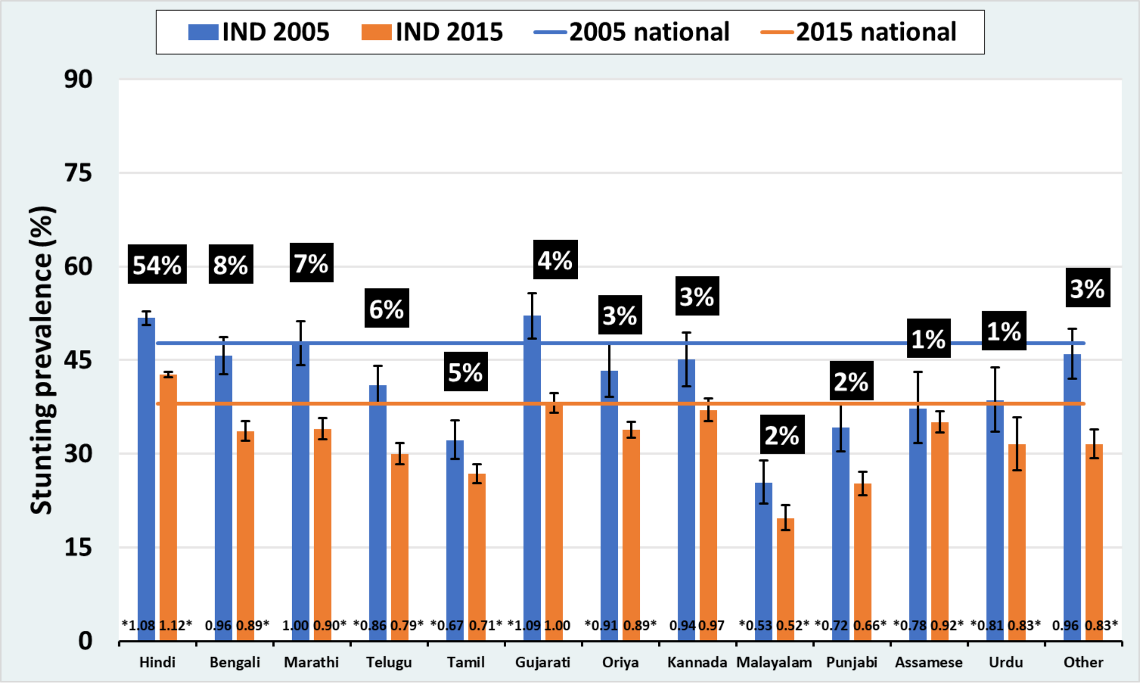

Kazakhstan

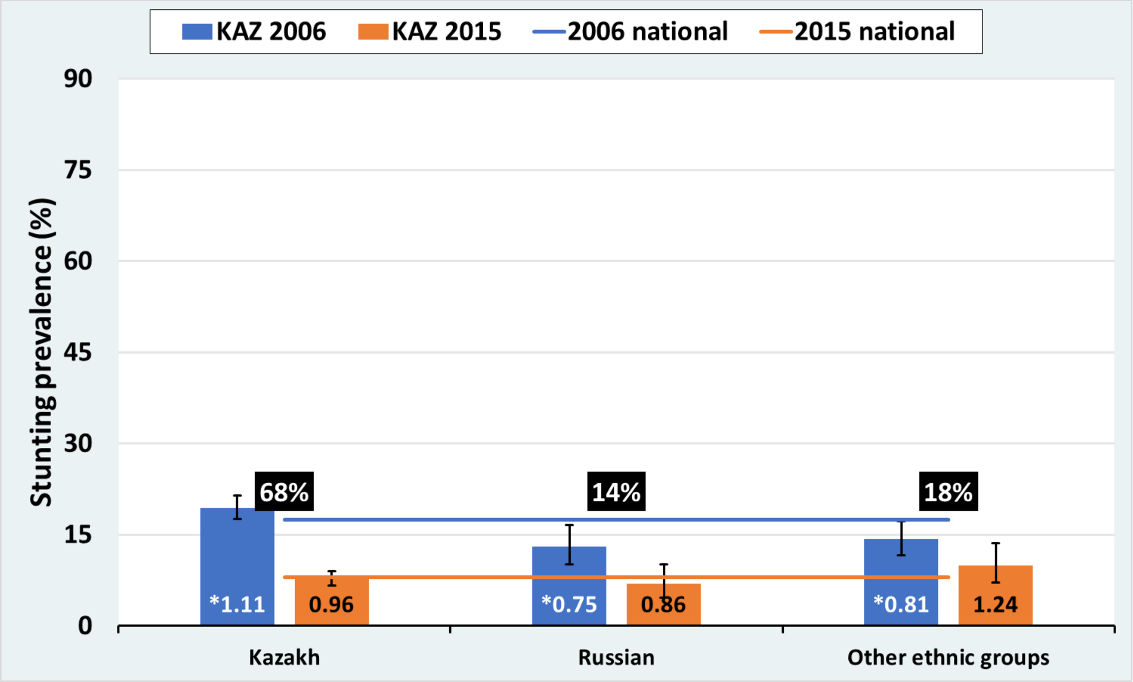

Kenya

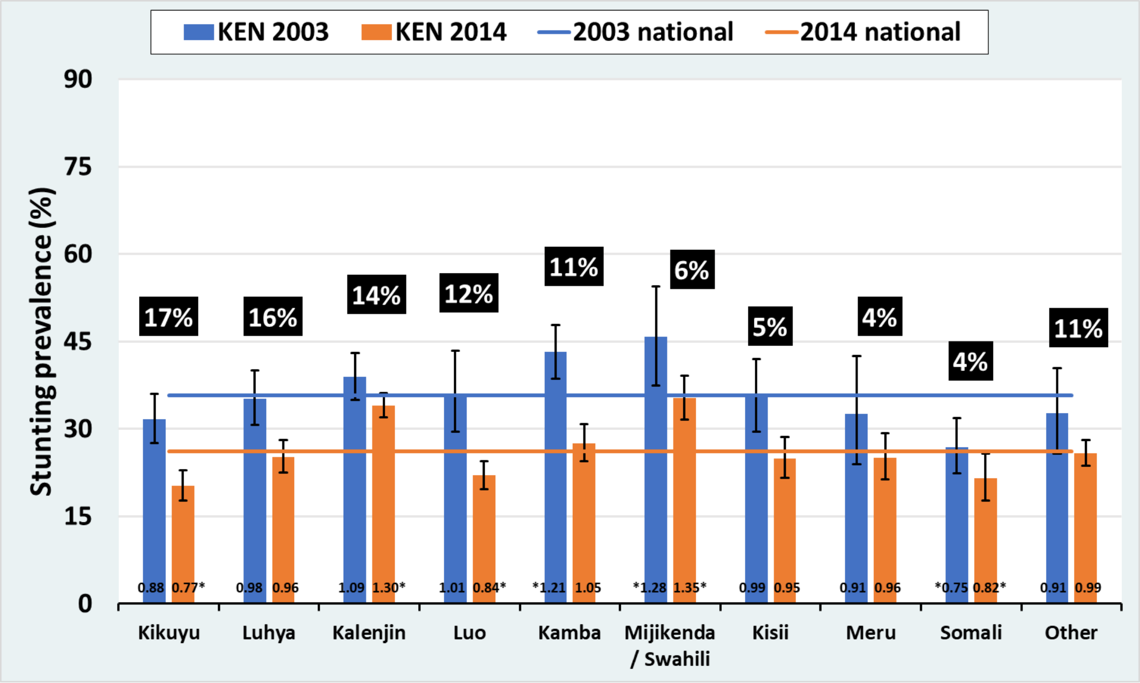

Kosovo

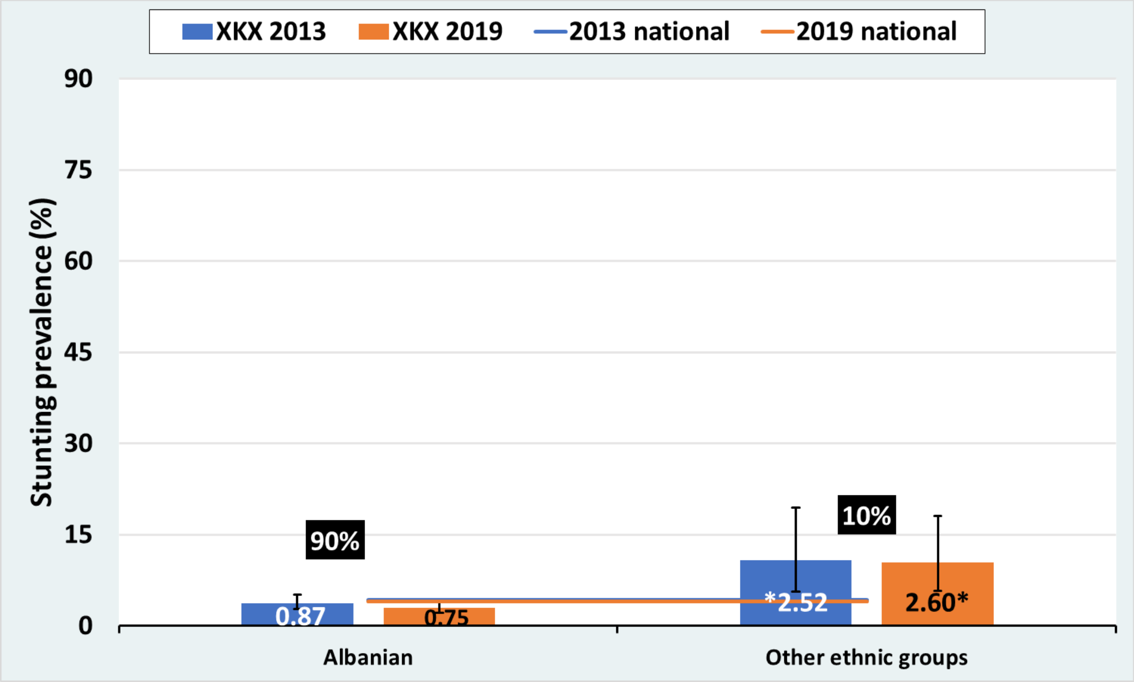

Kyrgyzstan

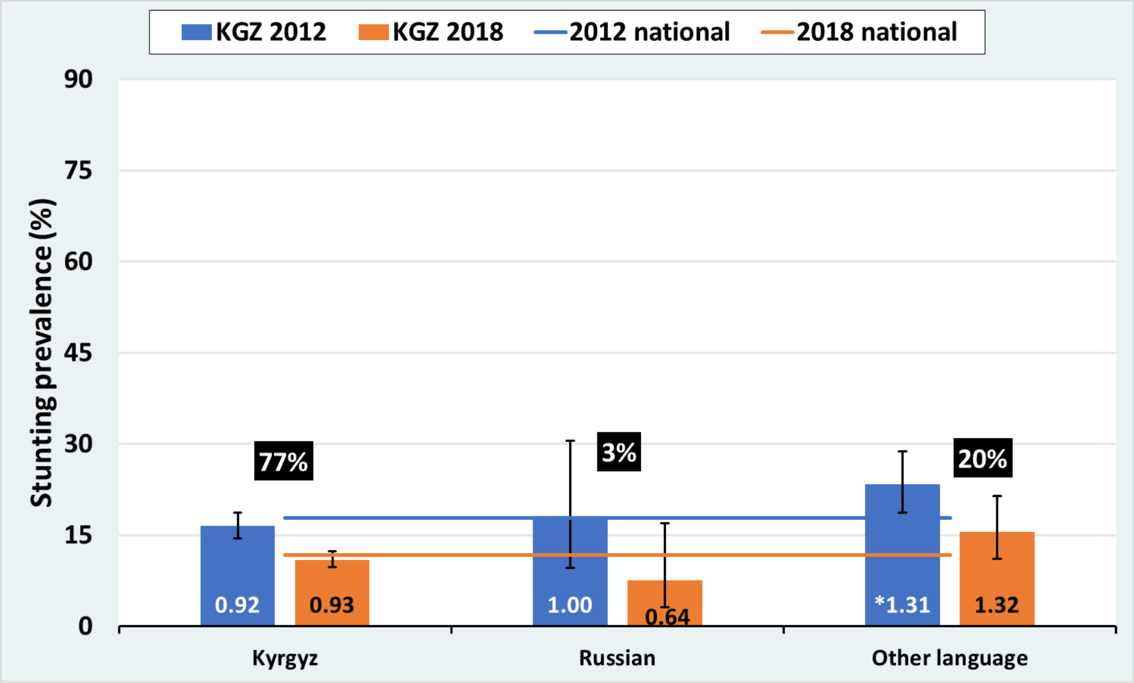

Lao PDR

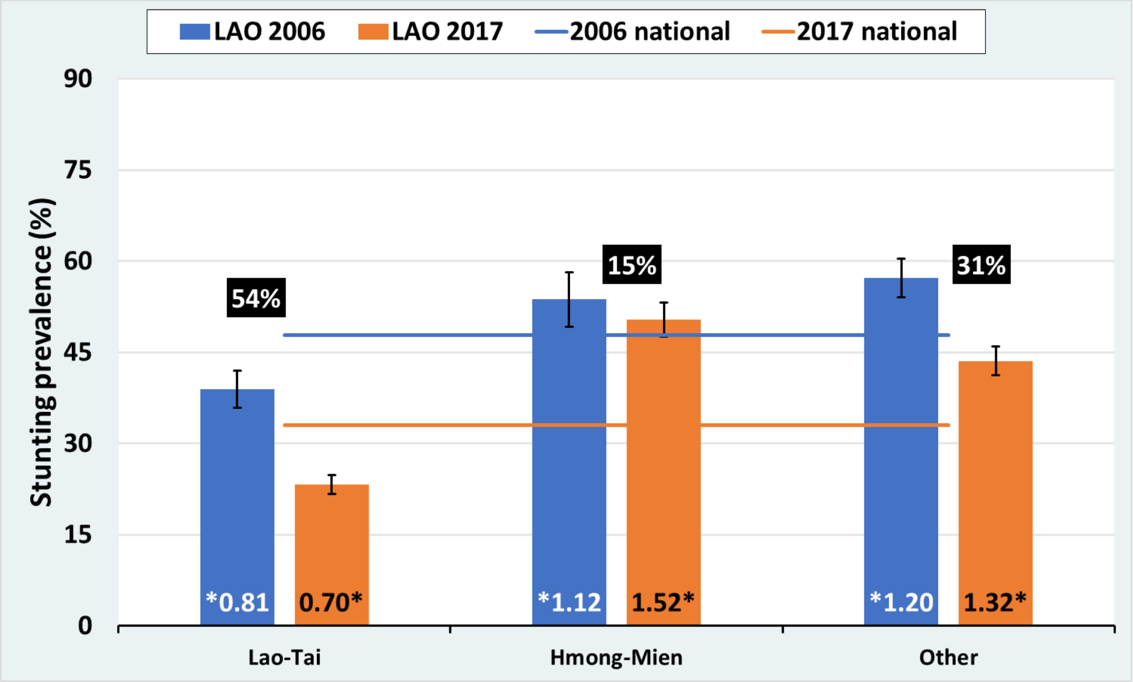

Malawi

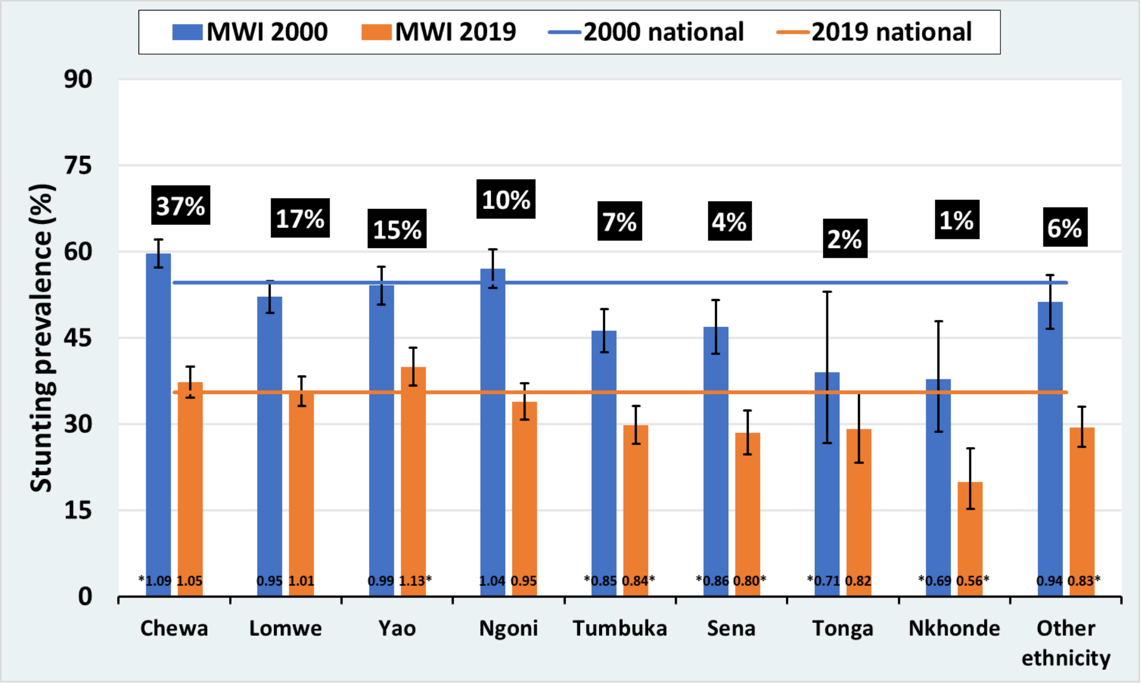

Mali

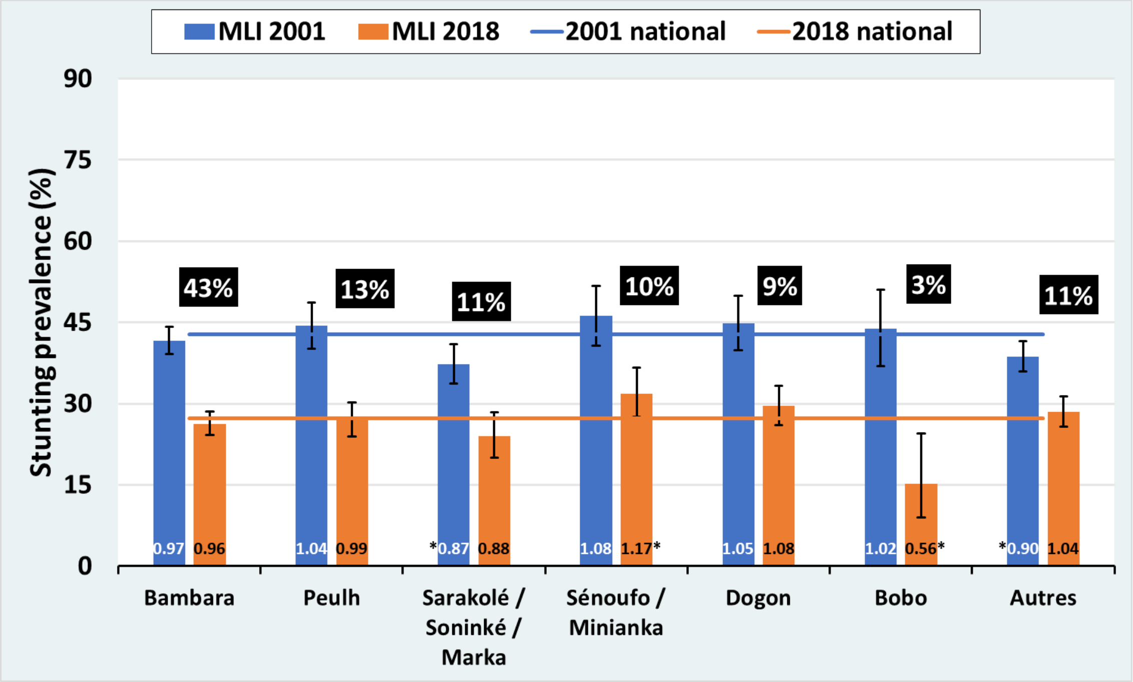

Mauritania

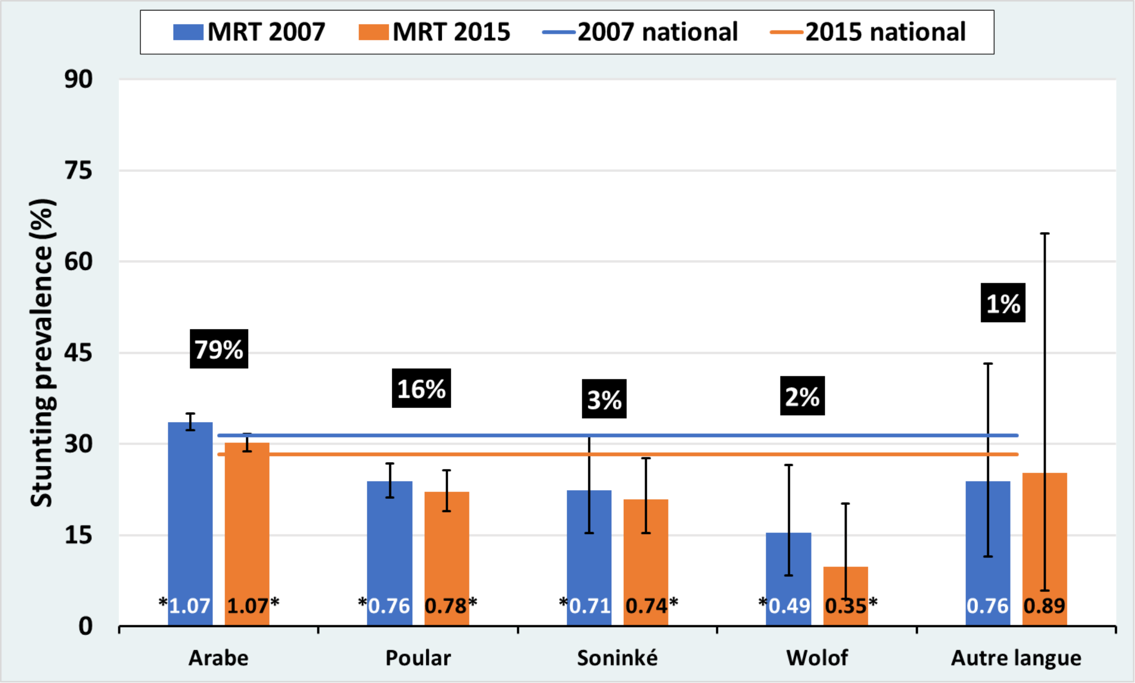

Moldova

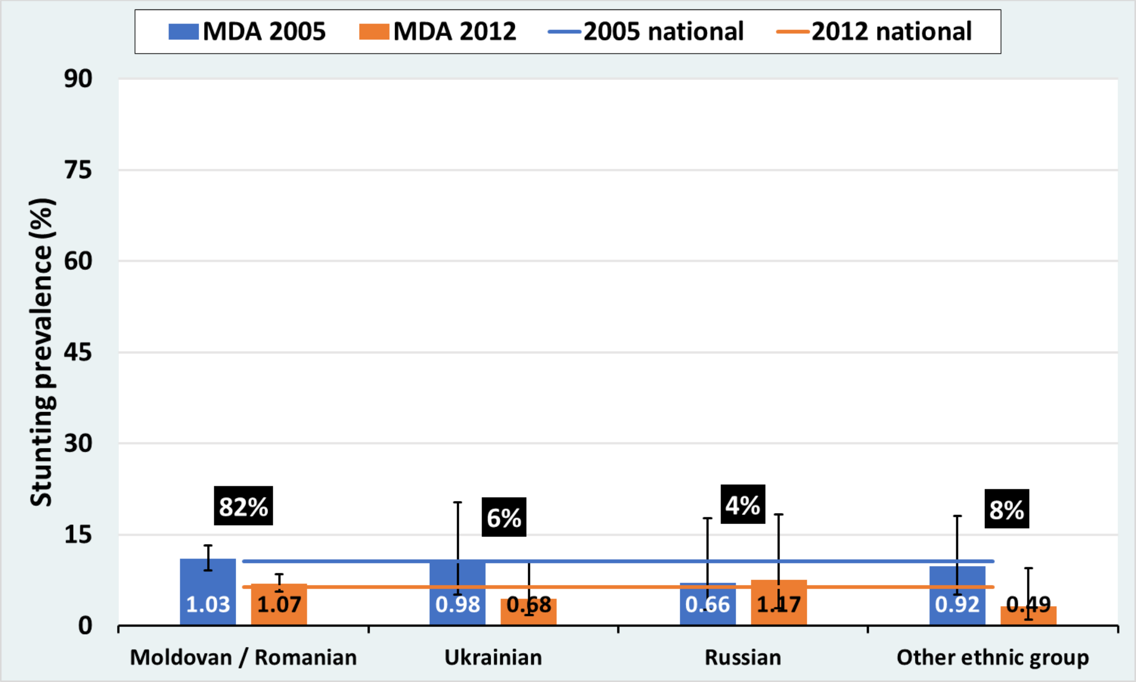

Mongolia

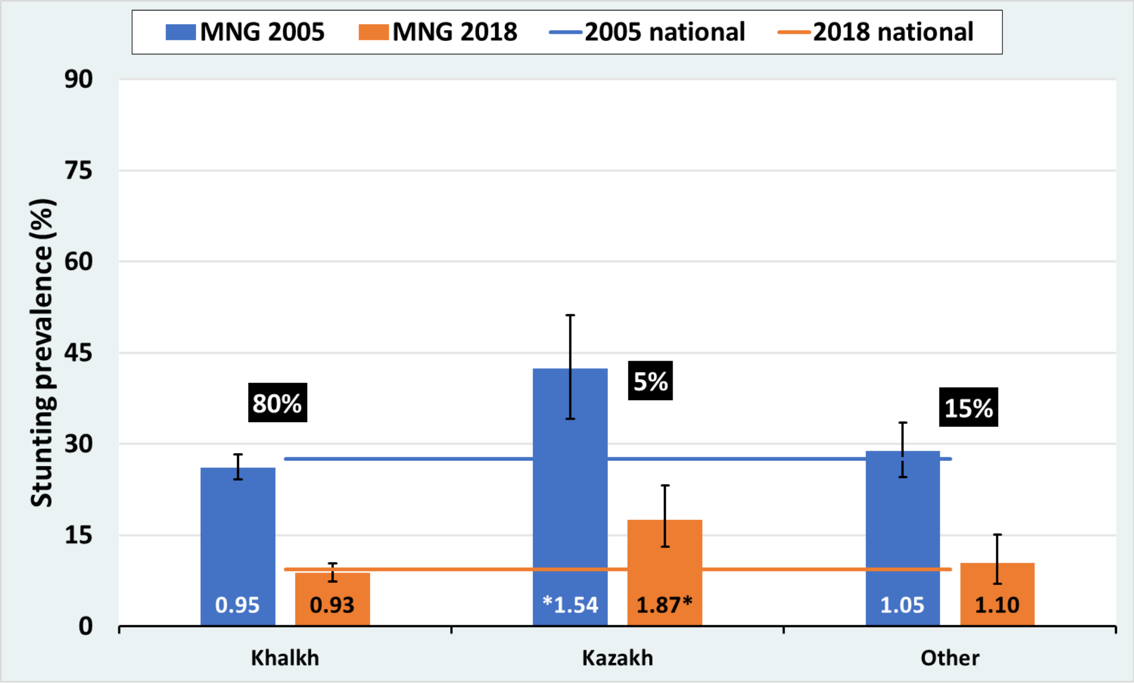

Mozambique

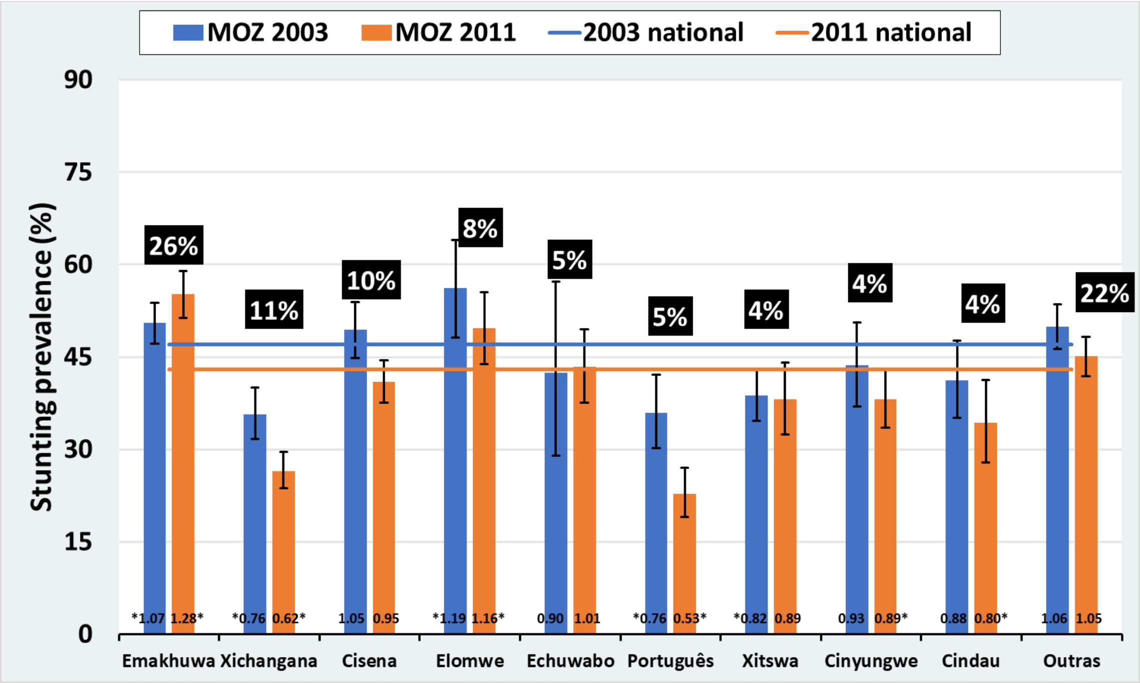

Namibia

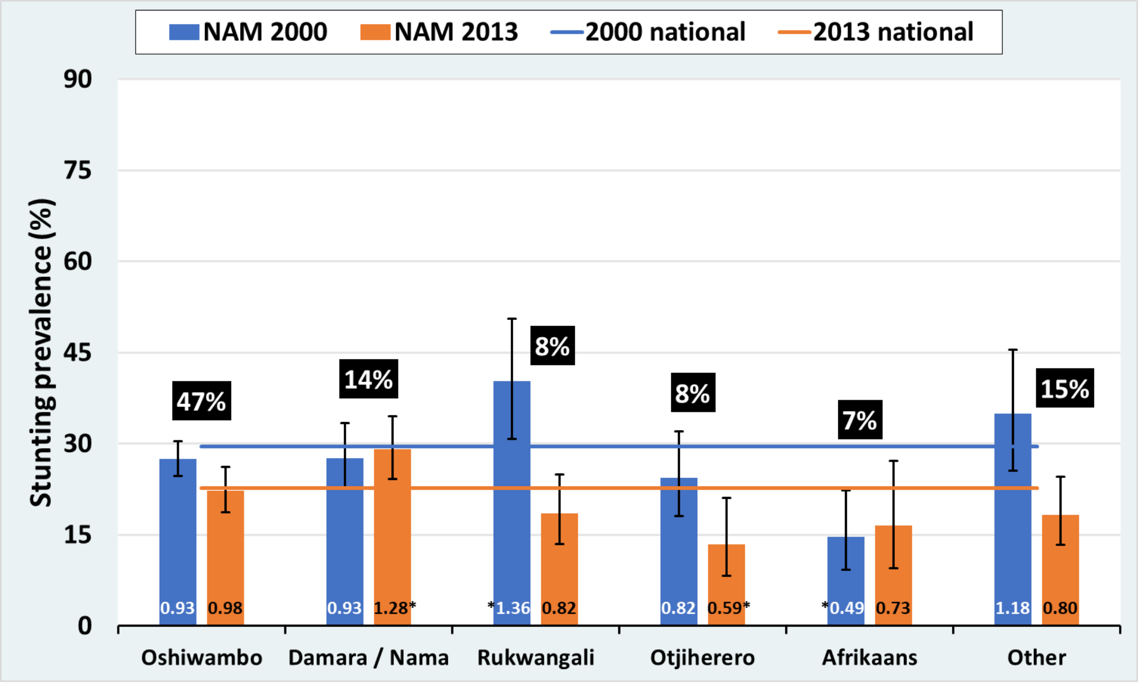

Nepal

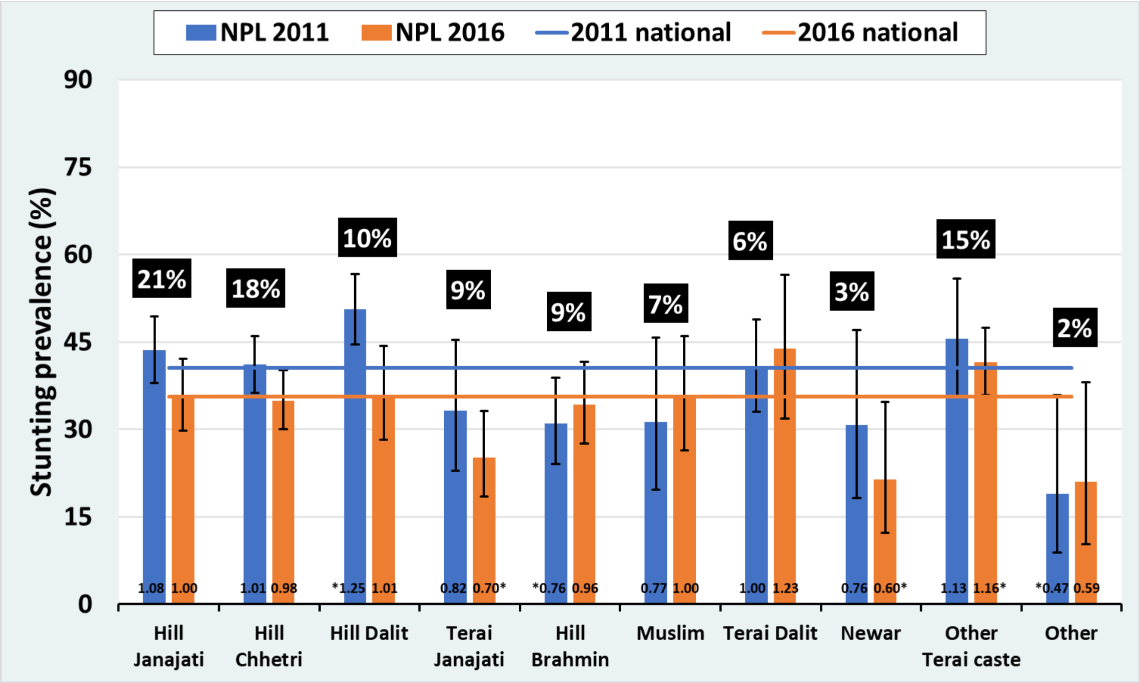
NPL 2011
NPL 2016
2011 national
2016 national
Stunting prevalence (%)
90
75
60
45
30
15
0
21%
18%
10%
9%
9%
7%
6%
3%
15%
2%
1.08 1.00
1.01 0.98
*1.25 1.01
0.82 0.70*
*0.76 0.96
0.77 1.00
1.00 1.23
0.76 0.60*
1.13 1.16*
*0.47 0.59
Hill Janajati
Hill Chhetri
Hill Dalit
Terai Janajati
Hill Brahmin
Muslim
Terai Dalit
Newar
Other Terai caste
Other

Niger

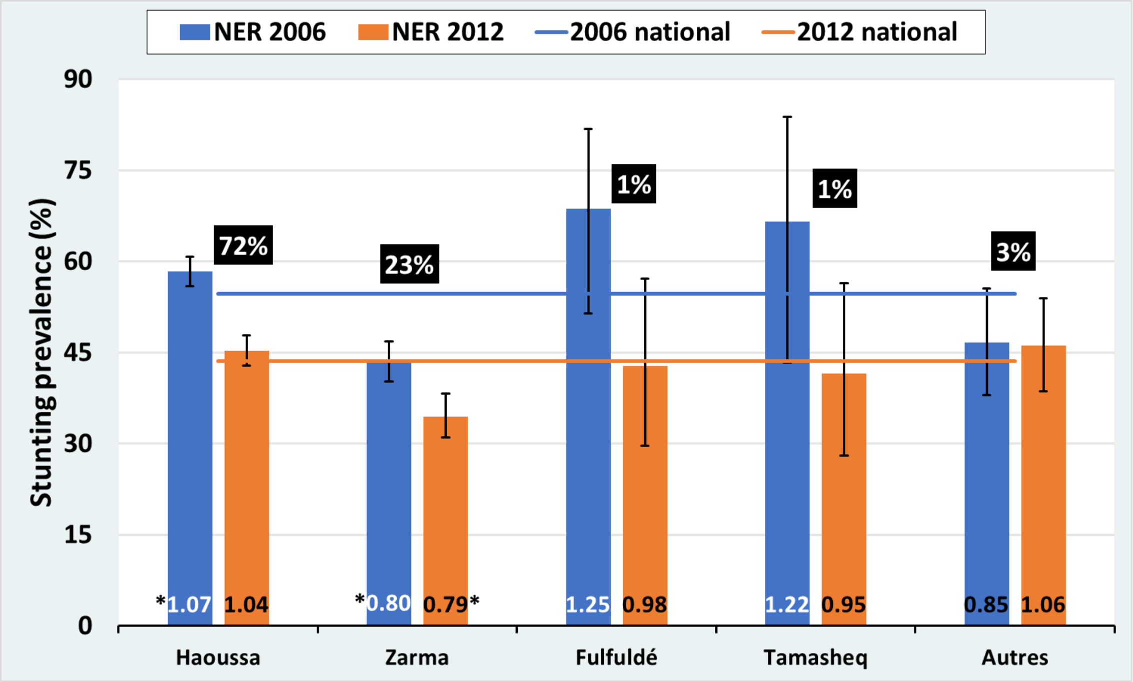
NER 2006
NER 2012
2006 national
2012 national
Stunting prevalence (%)
90
75
60
45
30
15
0
72%
23%
1%
1%
3%
*1.07 1.04
0.80 0.79
1.25 0.98
1.22 0.95
0.85 1.06
Haoussa
Zarma
Fulfuldé
Tamasheq
Autres

Nigeria

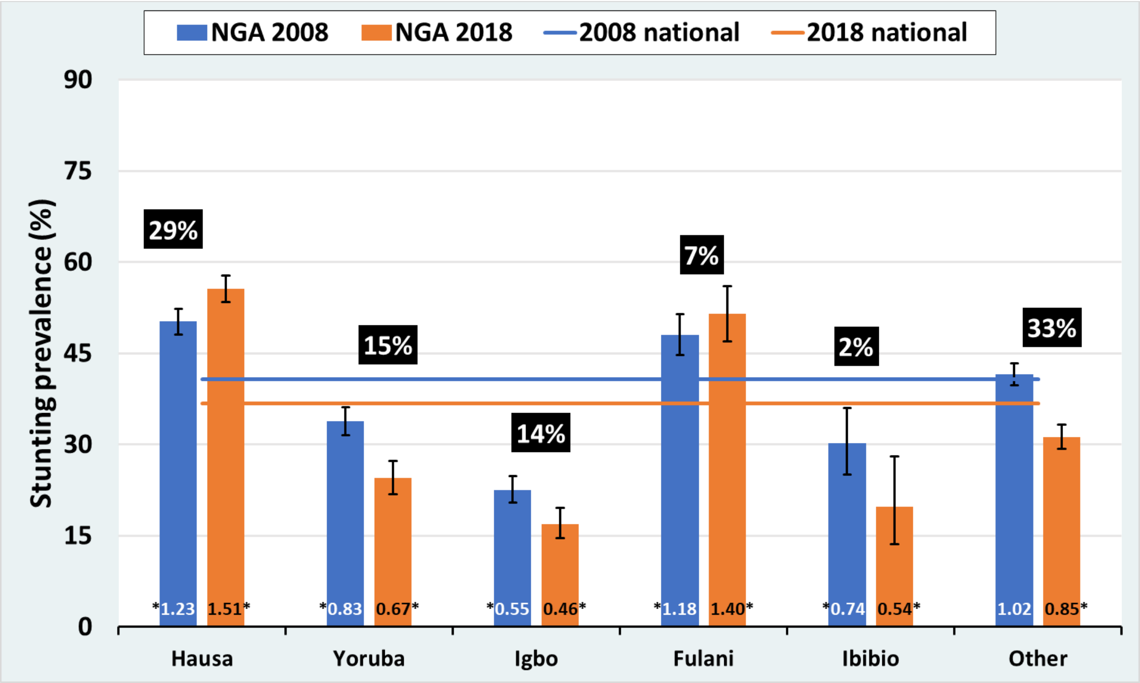

North Macedonia

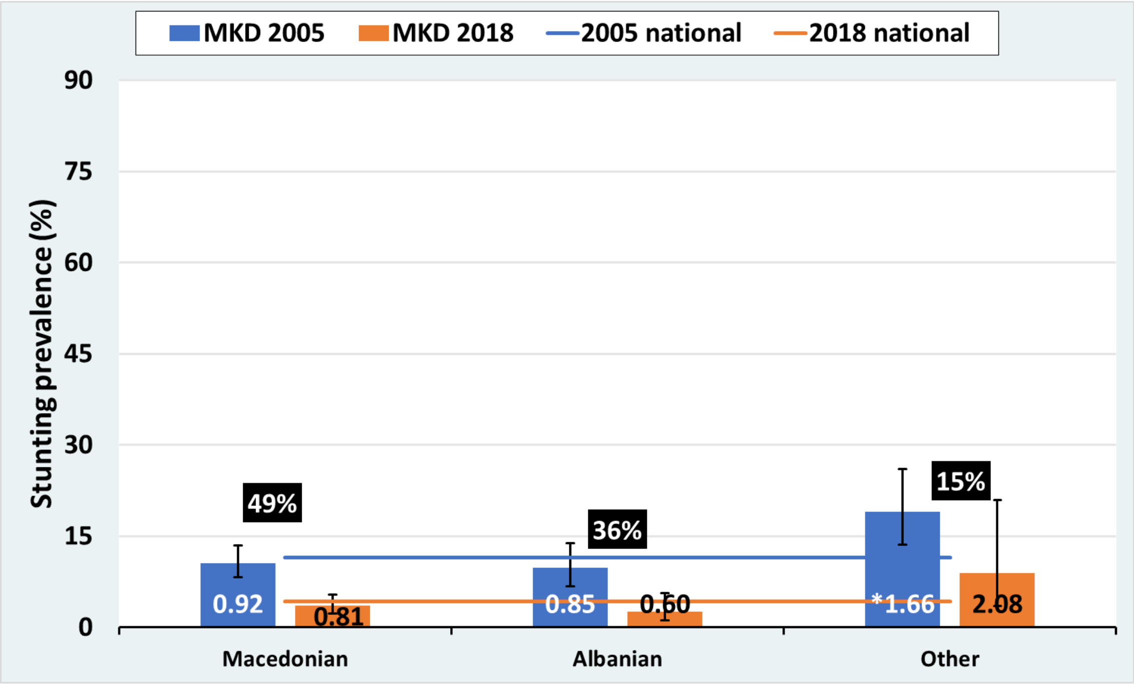

Pakistan

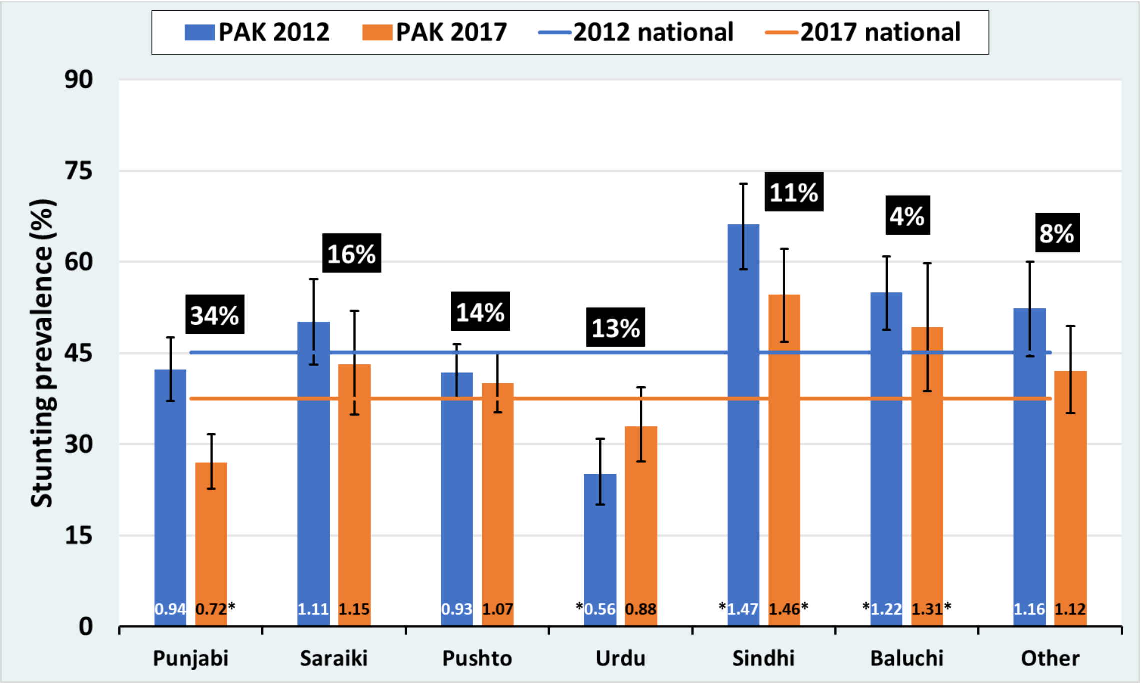

Peru

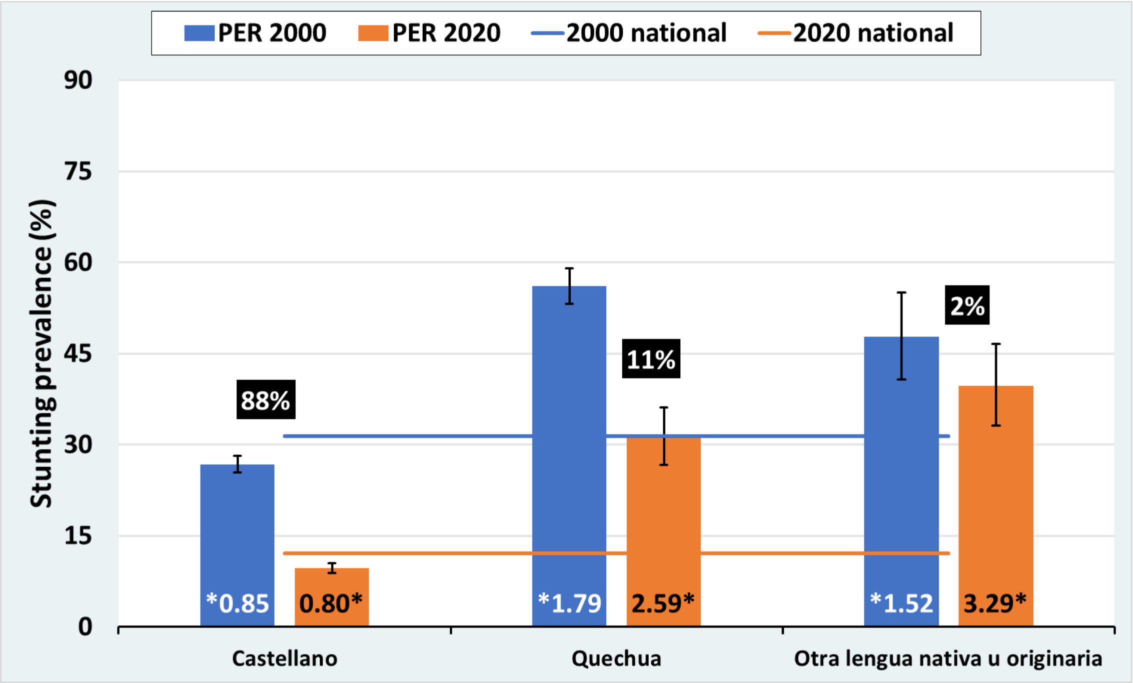

Sao Tome and Principe

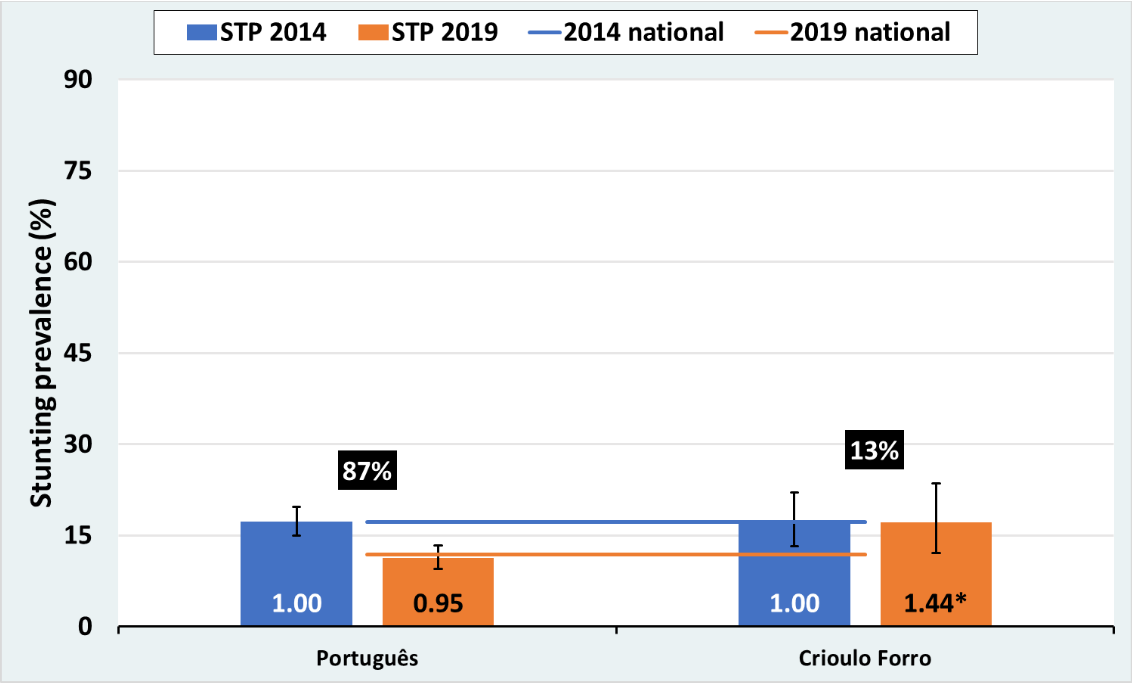

Senegal

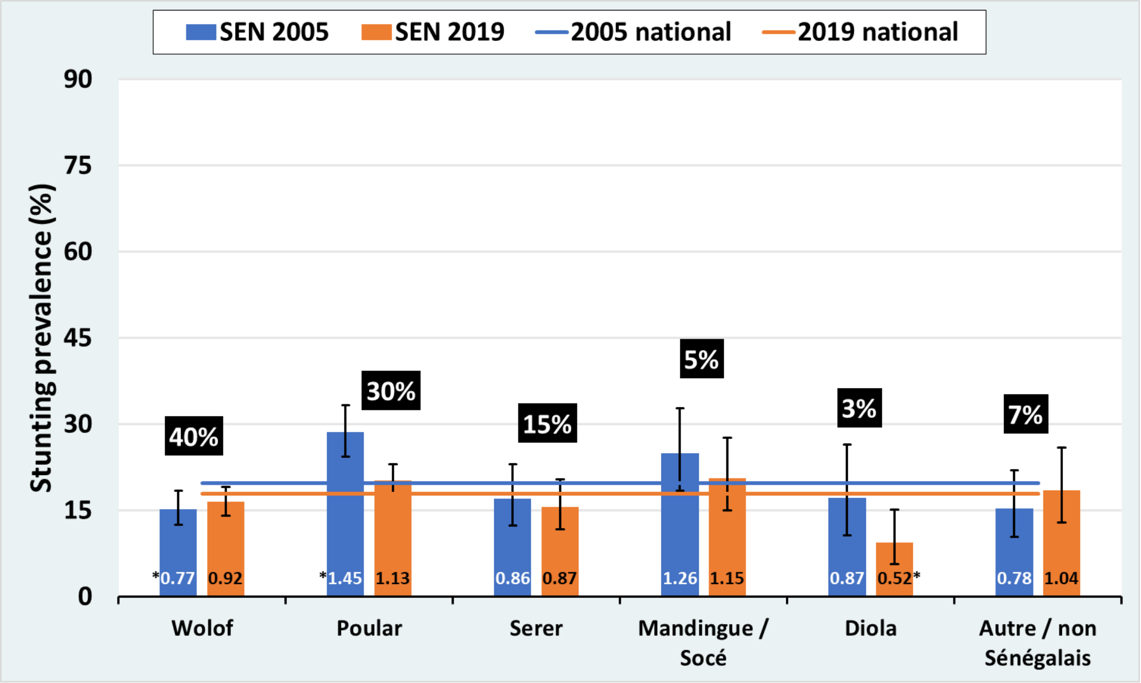

Serbia

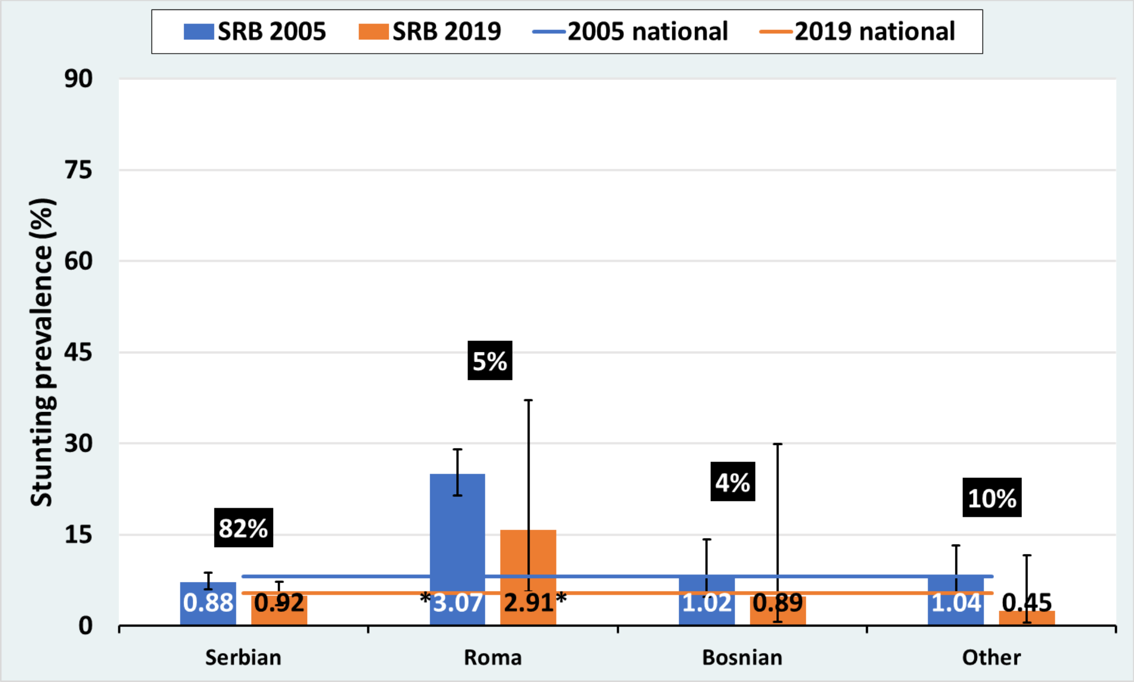

Sierra Leone

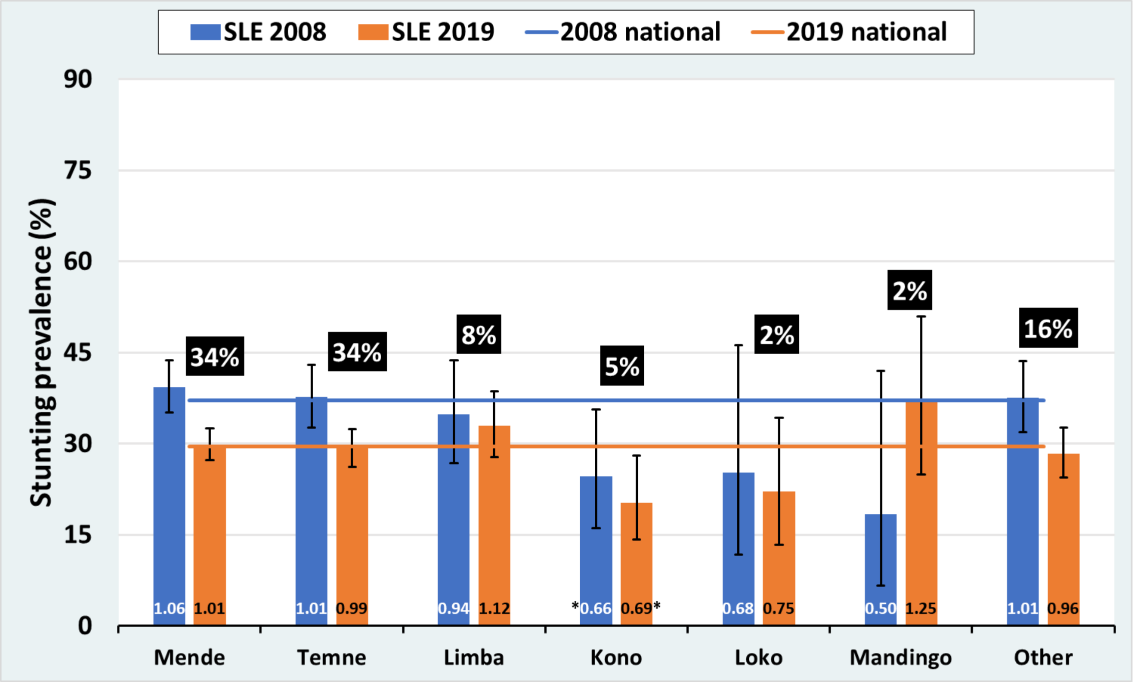

Suriname

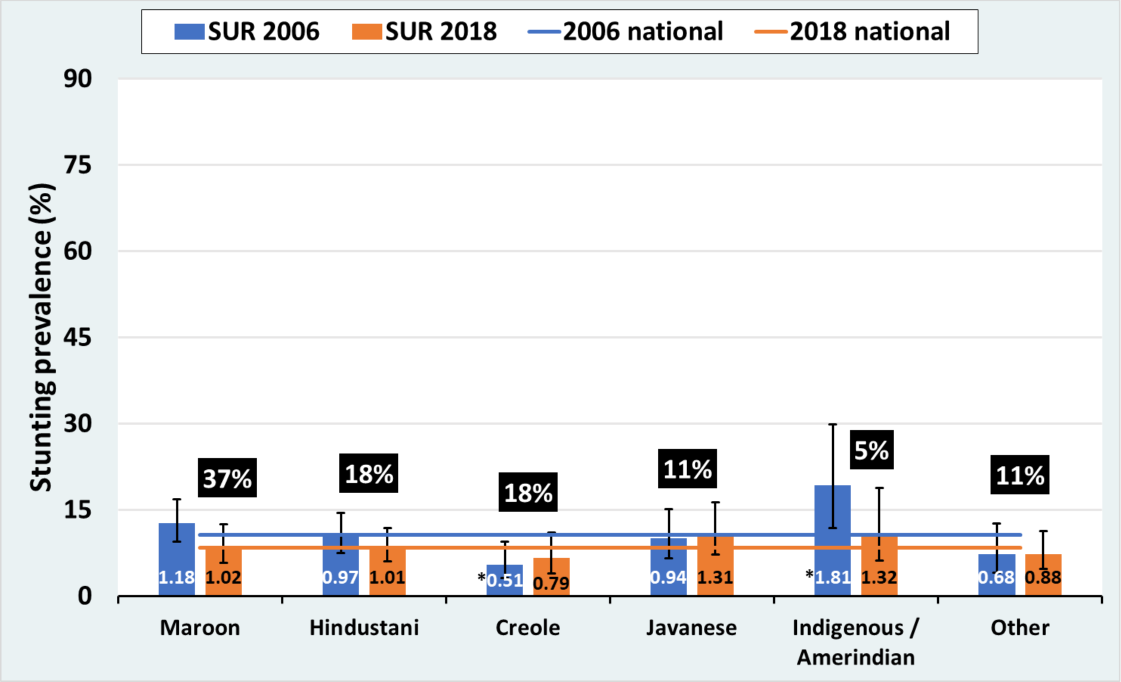

Tajikistan

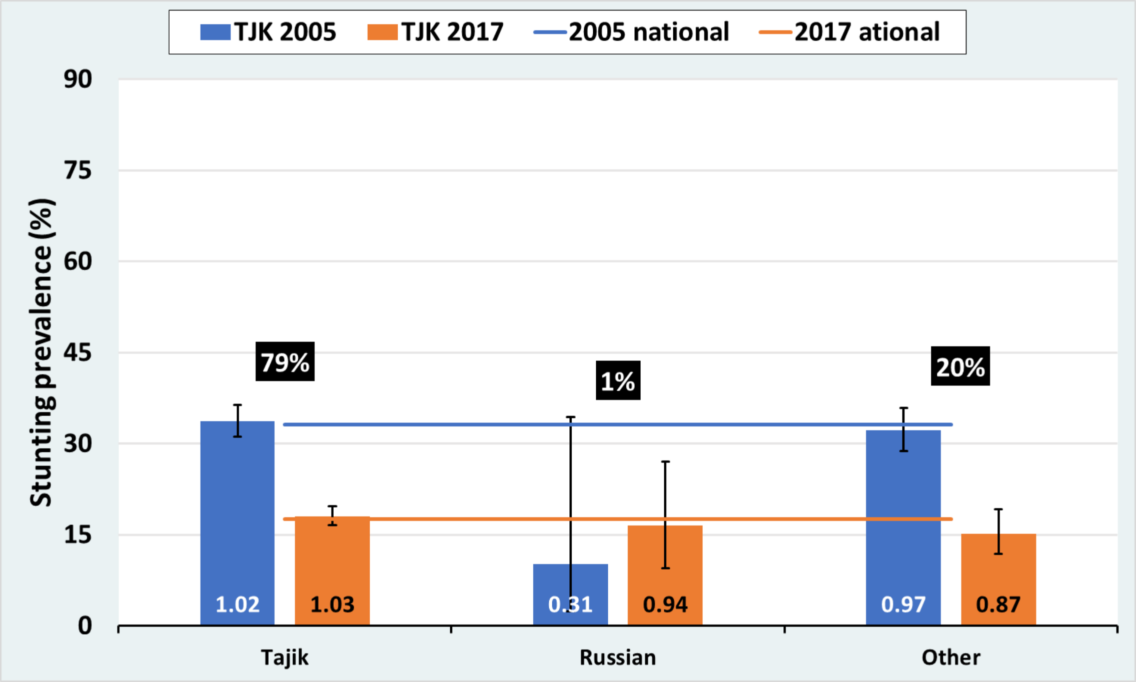

Thailand

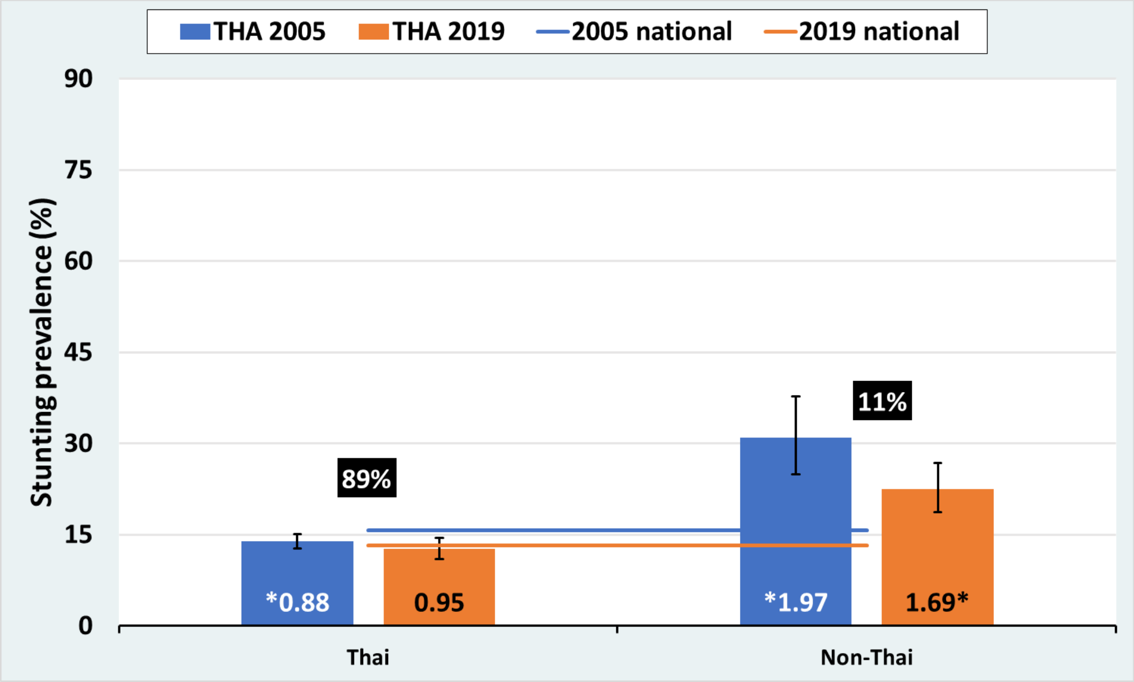

Timor-Leste

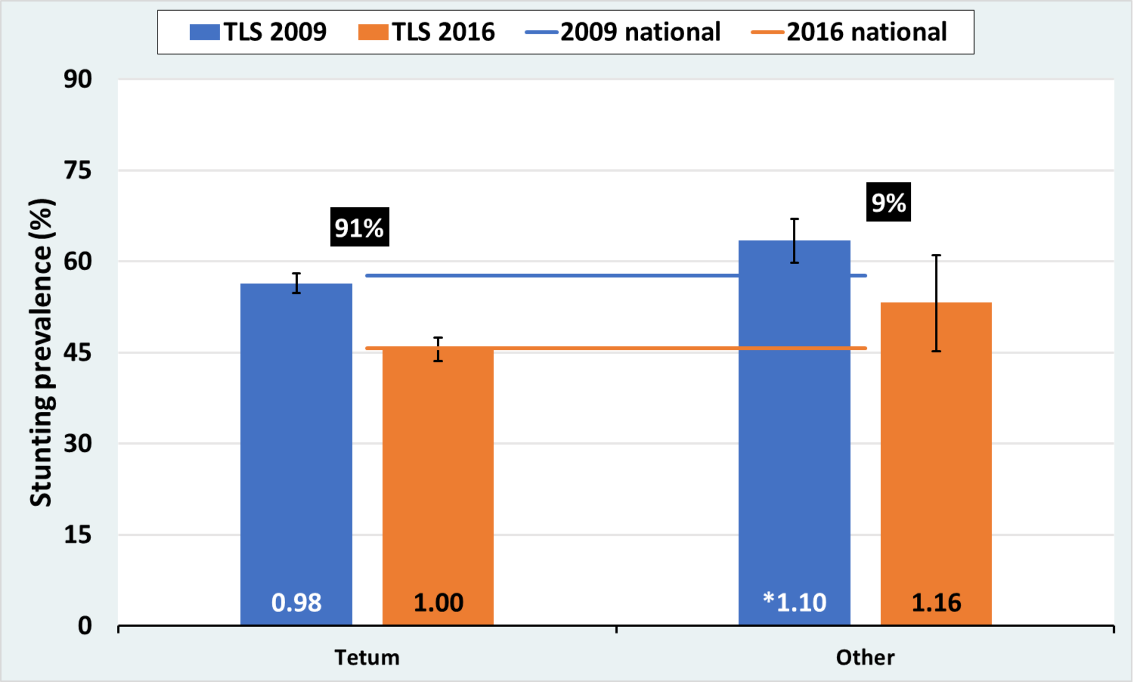

Togo

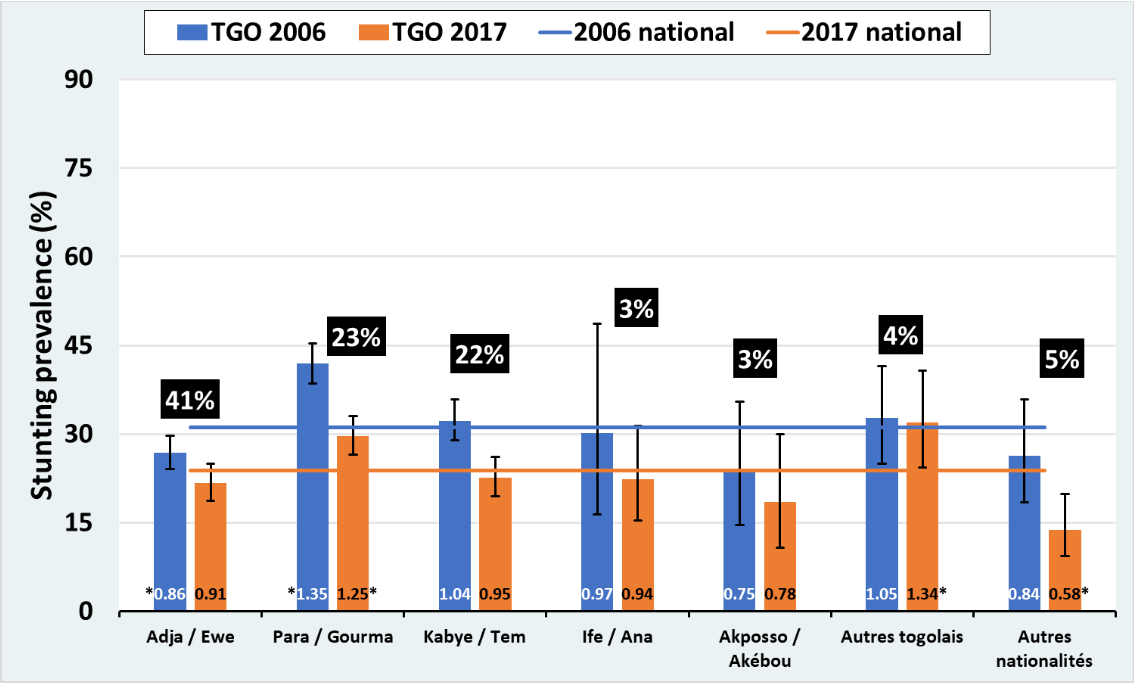

Türkiye

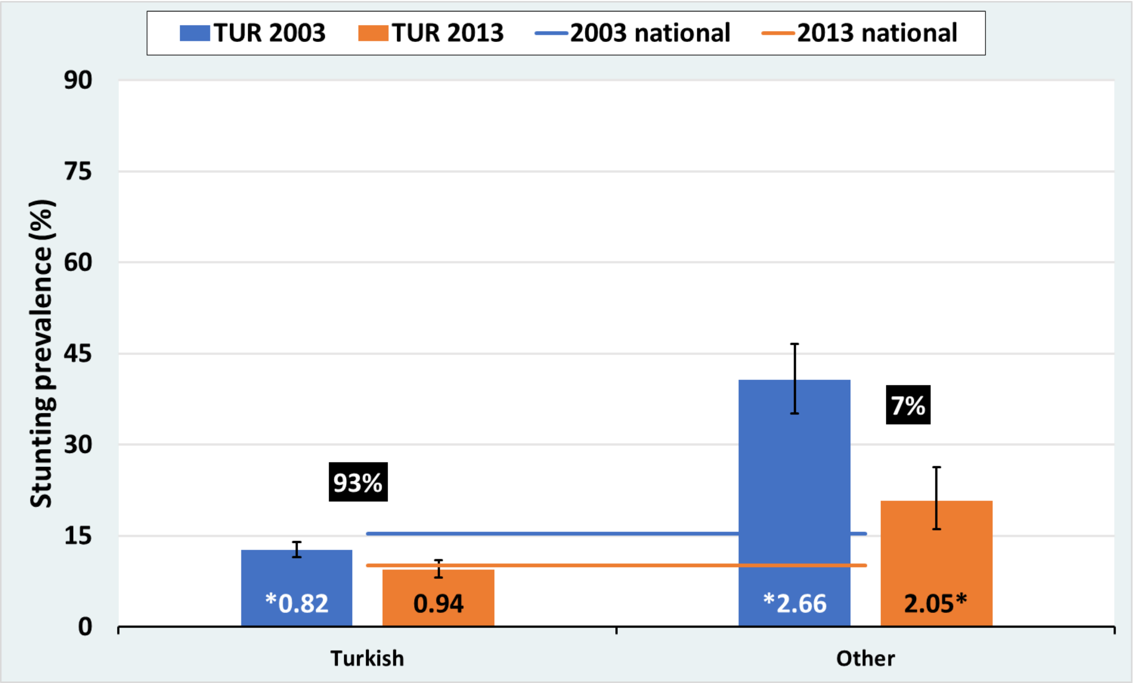

Turkmenistan

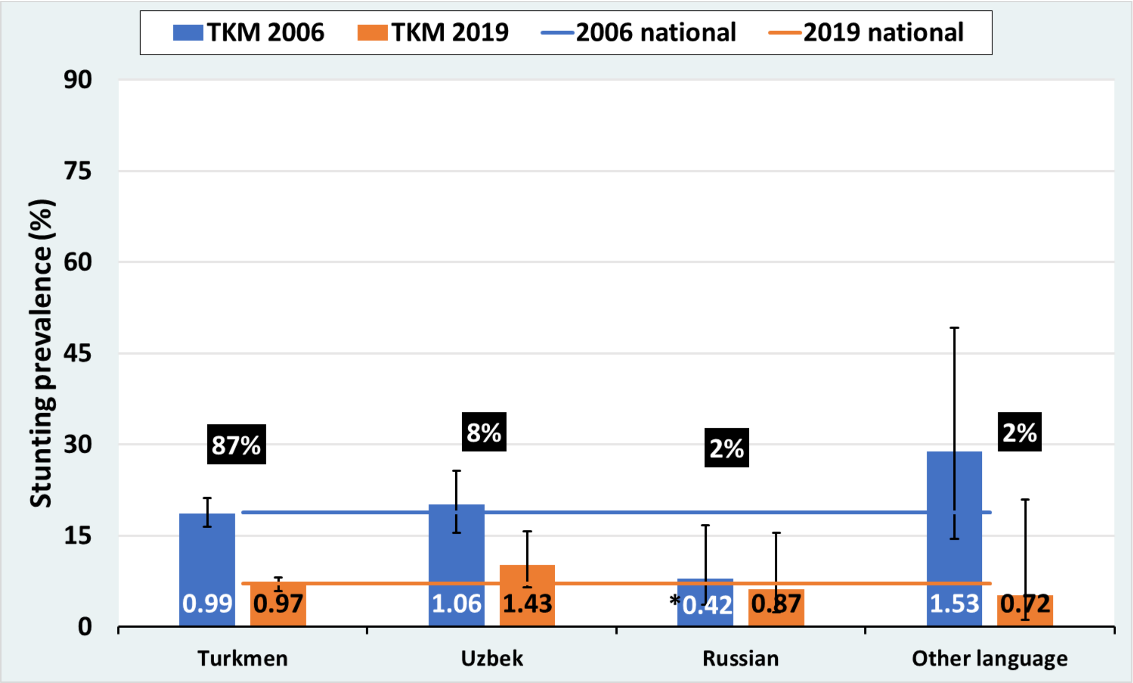

Uganda

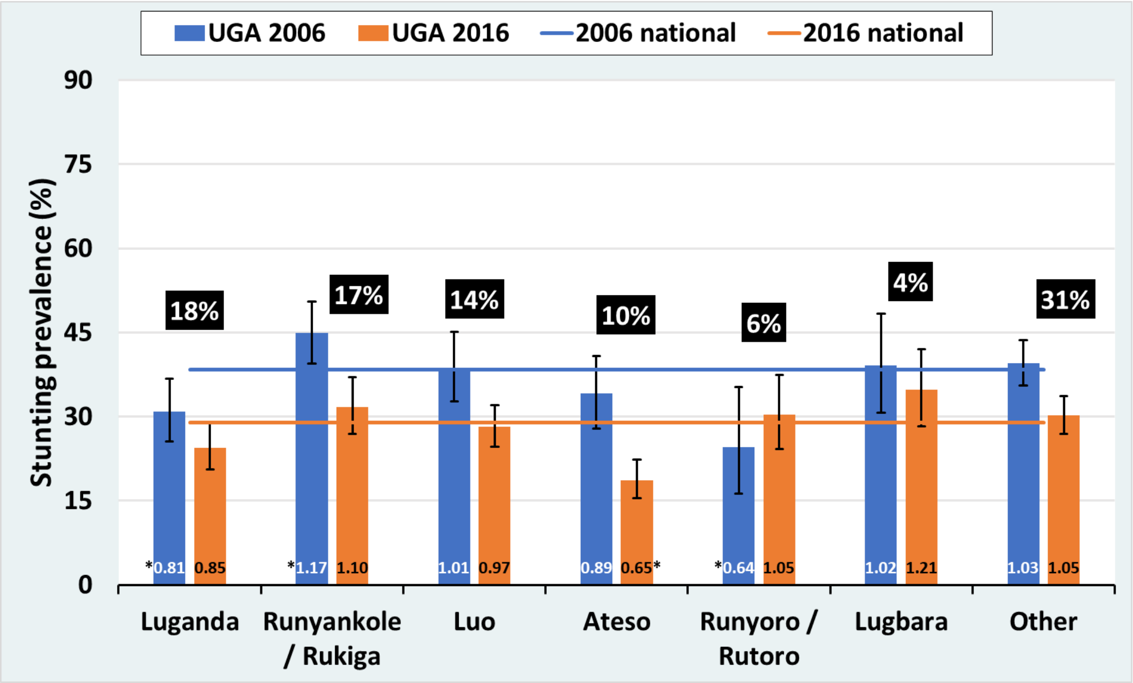

Zambia

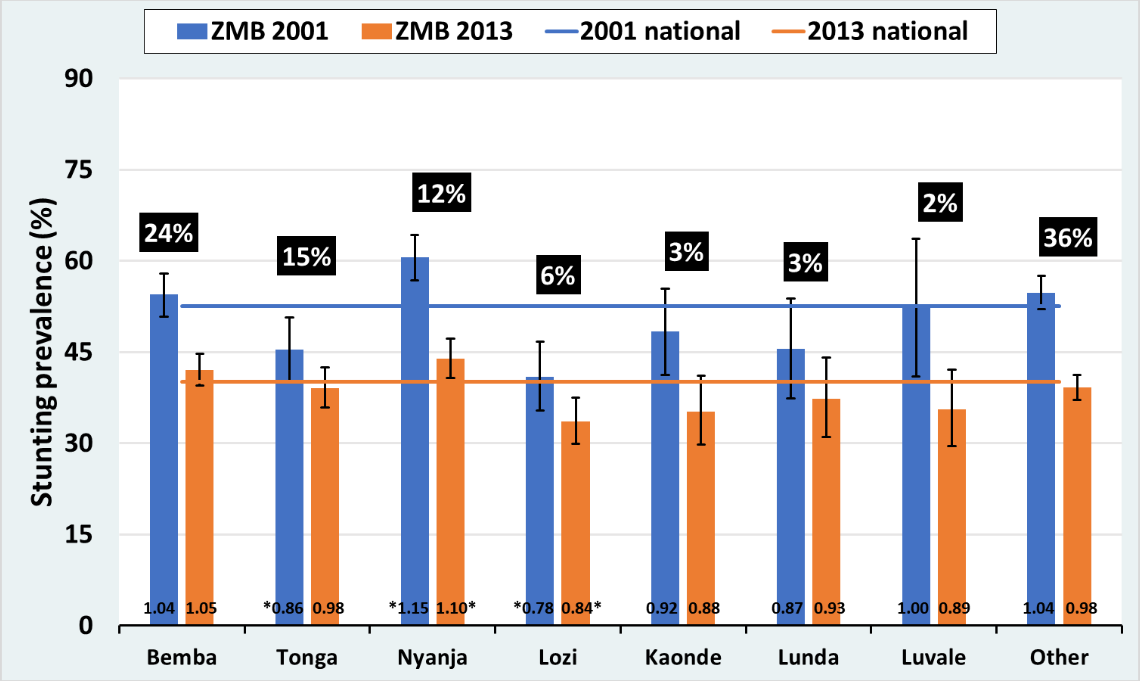

Zimbabwe

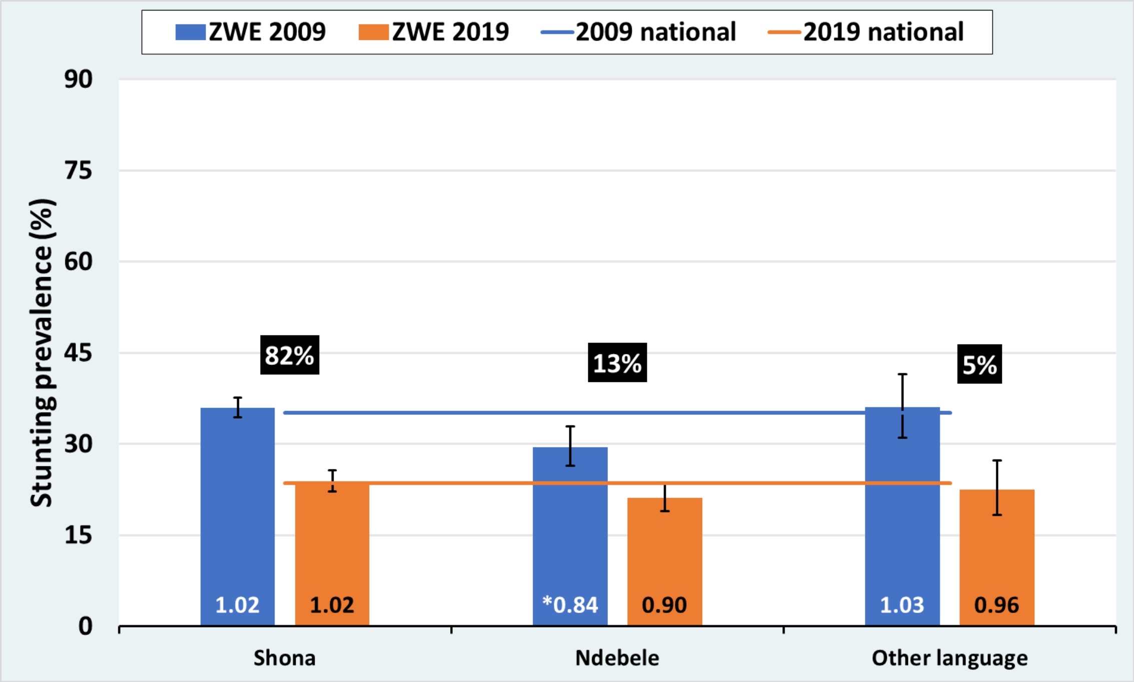

Supplement: Supplementary file 4 — Additional file 4. Title: Stunting prevalence by ethnic groups in the first and last surveys. Results for selected countries. The numbers in the black rectangles show the average proportion of the samples for each ethnic group in the two surveys. The numbers at the bottom of the bars show the ratio between the rate in a particular ethnic group and the national rate for that point in time. Description: One graph for each country showing Stunting prevalence by ethnic groups in the first and last surveys. [file 12939_2023_1888_MOESM4_ESM.pdf]
