# Supplementary material for: Time trends in ethnic inequalities in child health and nutrition: analysis of 59 low and middle-income countries
Source: Int J Equity Health. 2023 Apr 28;22:76. doi: 10.1186/s12939-023-01888-5 (PMC10148503; doi:10.1186/s12939-023-01888-5)

Supplementary figures 2. Composite coverage index by ethnic groups in the first and last surveys. Results for selected countries. The numbers in the black rectangles show the average proportion of the samples for each ethnic groups in the two surveys. The numbers at the bottom of the bars show the ratio between the rate in a particular ethnic group and the national rate for that point in time (* statistically significant in relation to the respective national average).

Albania

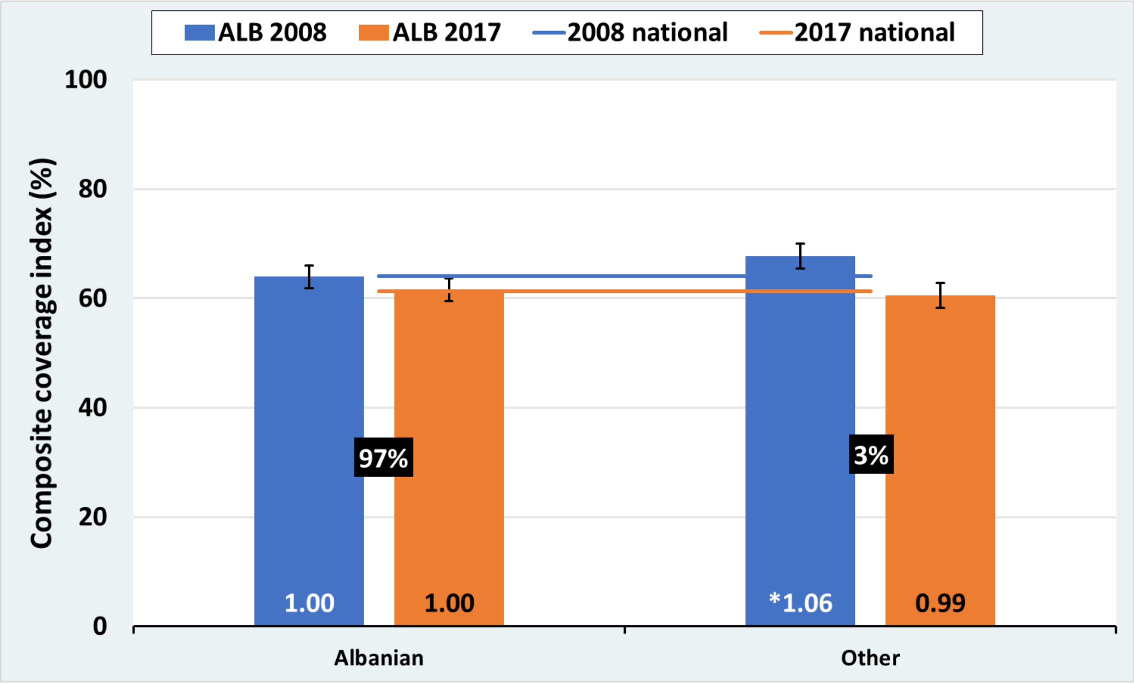

Belize

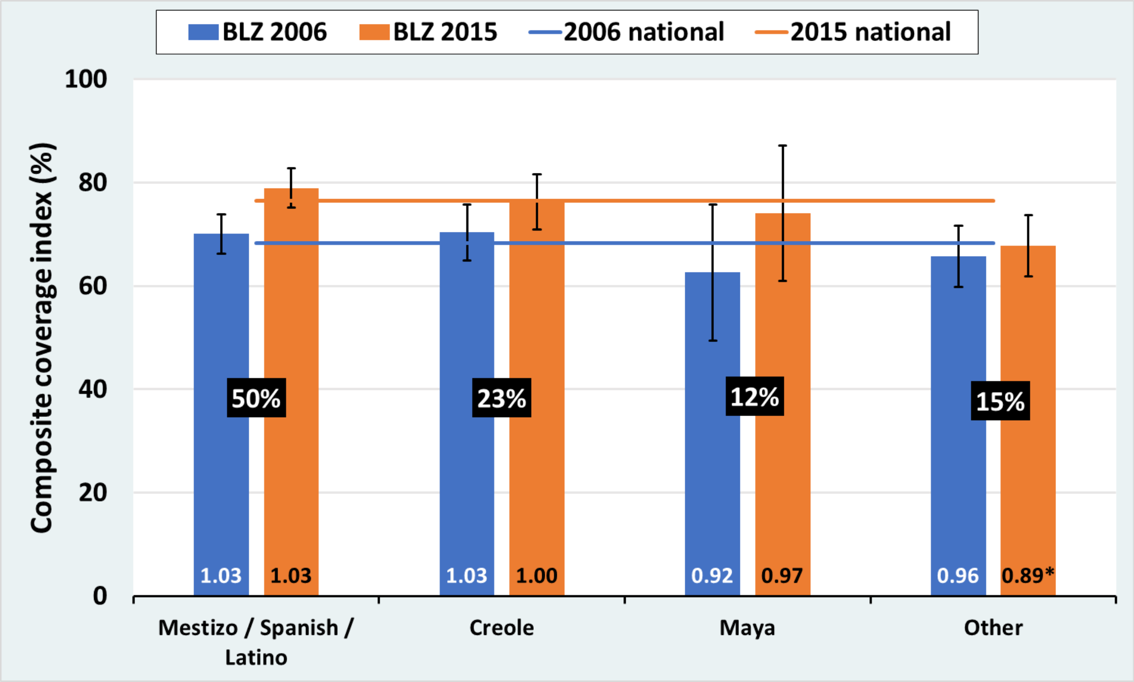

Benin

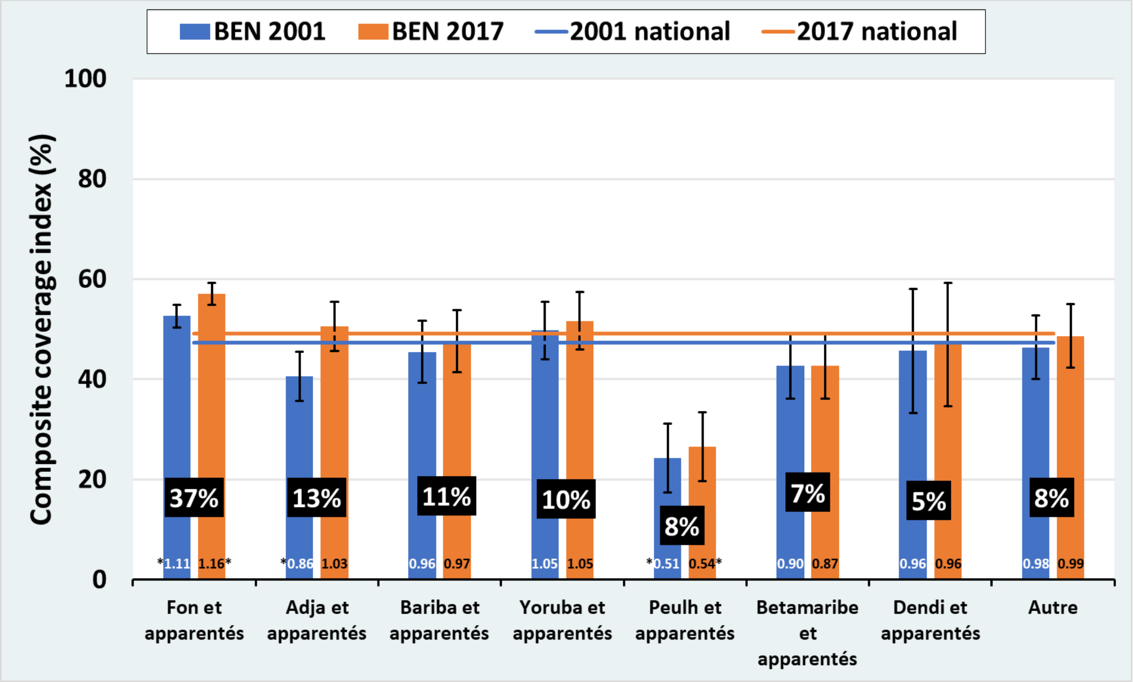

Burkina Faso

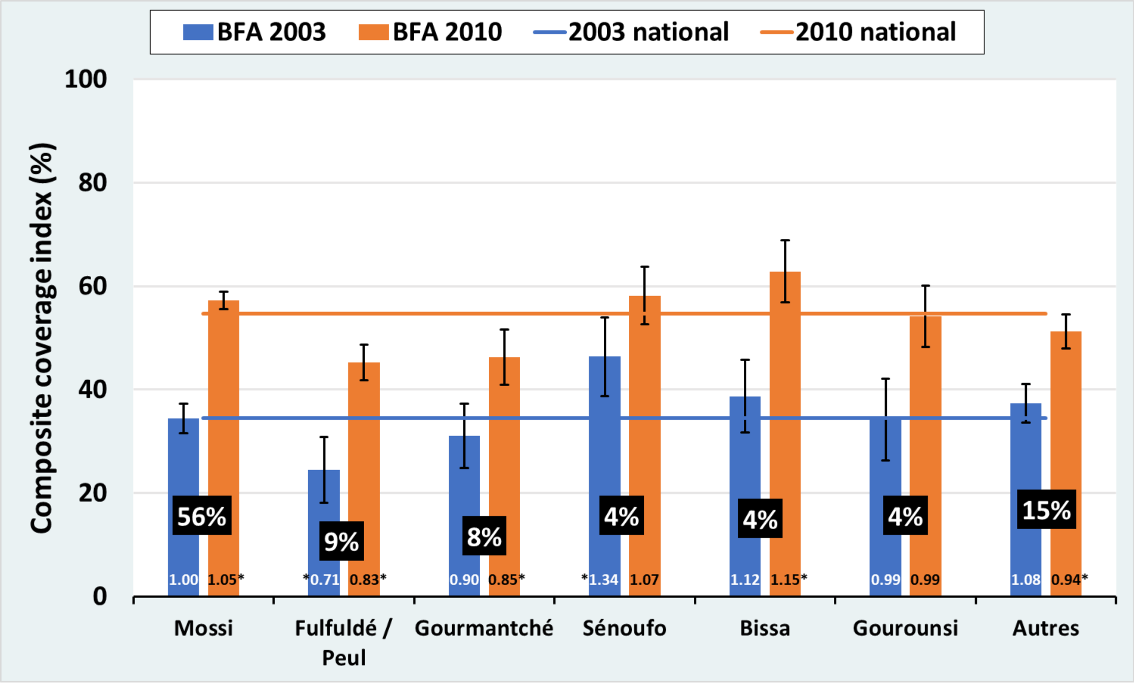

Cameroon

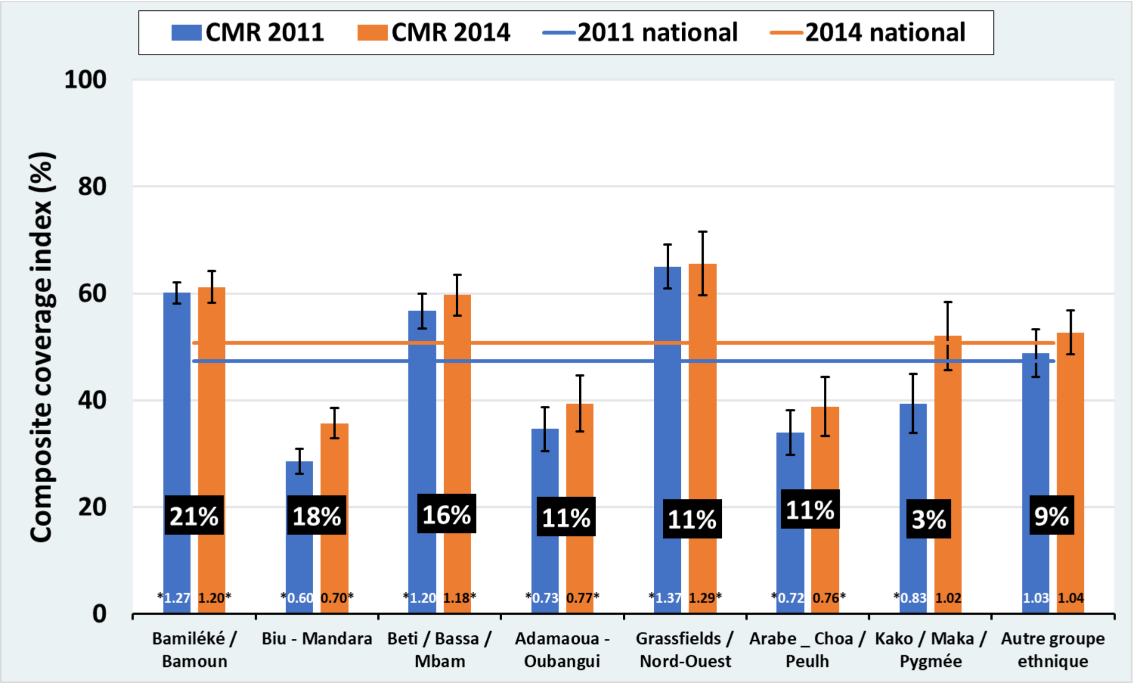

Central African Republic

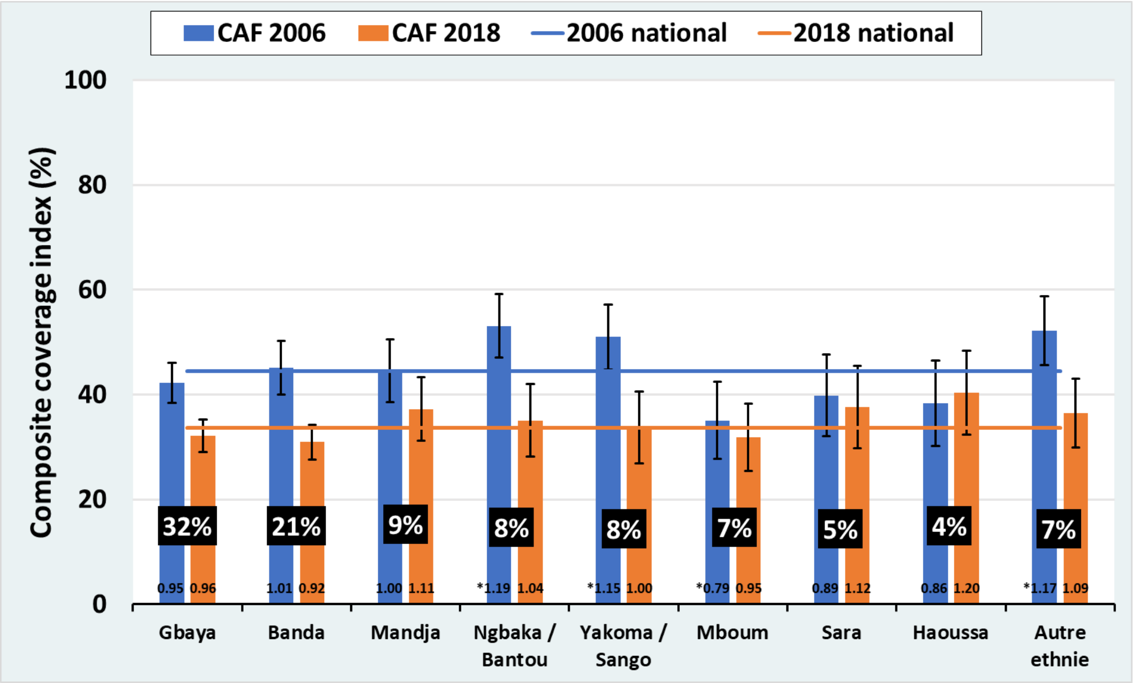

Chad

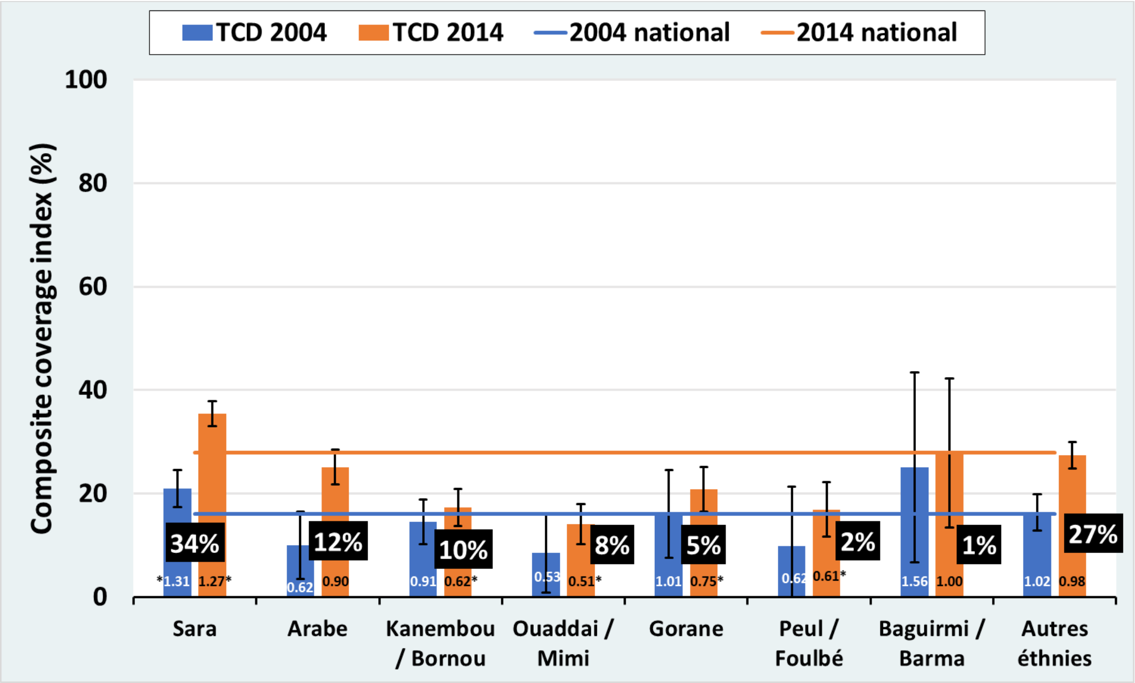

Congo DR

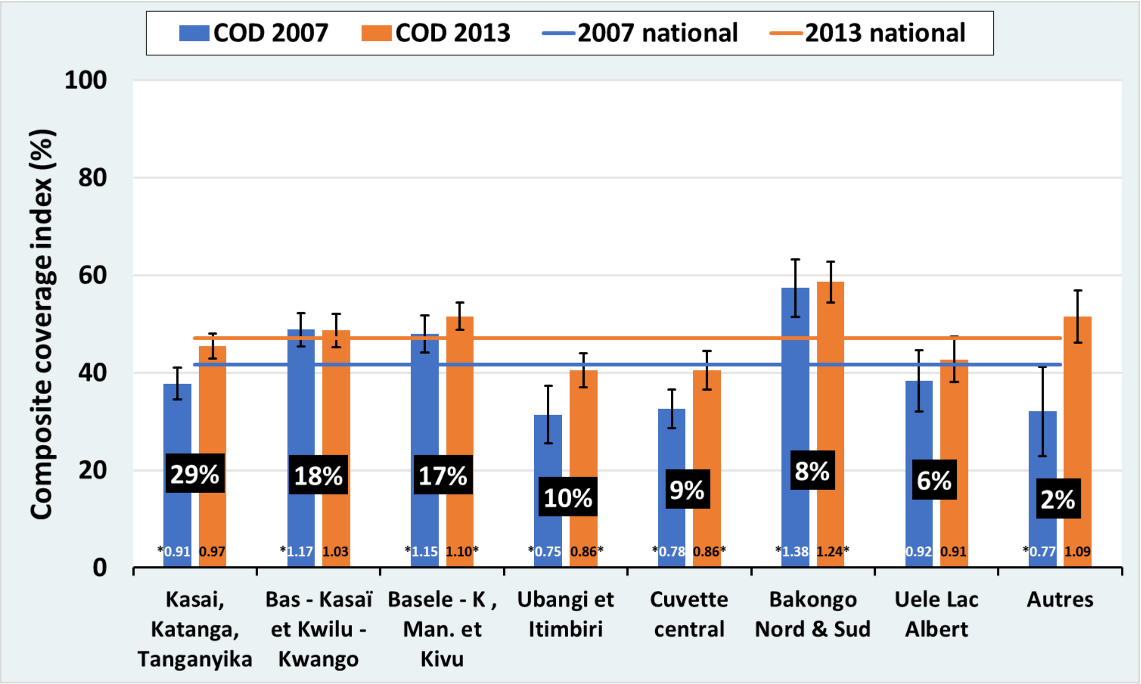

Congo Republic

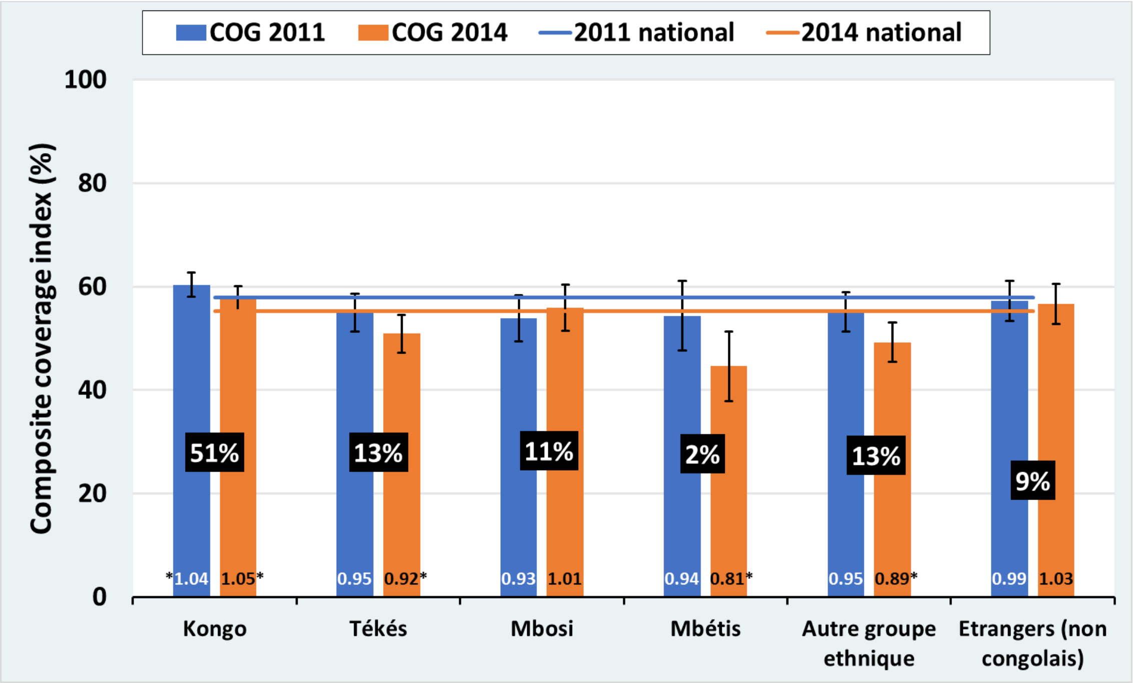

Costa Rica

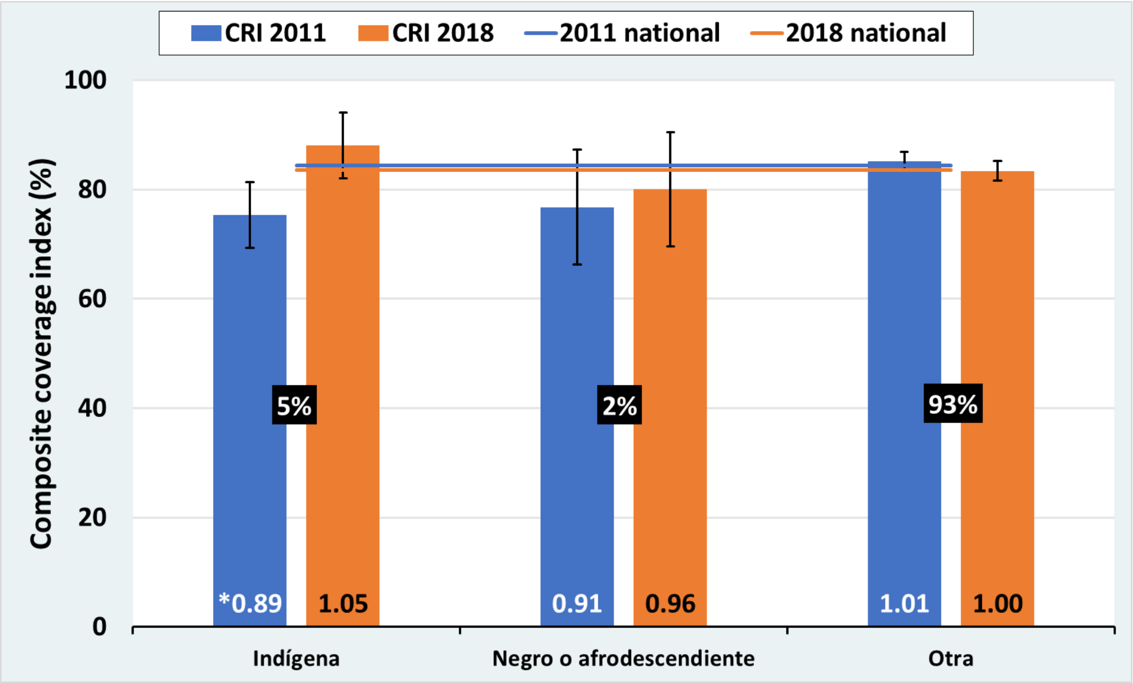

Côte d'Ivoire

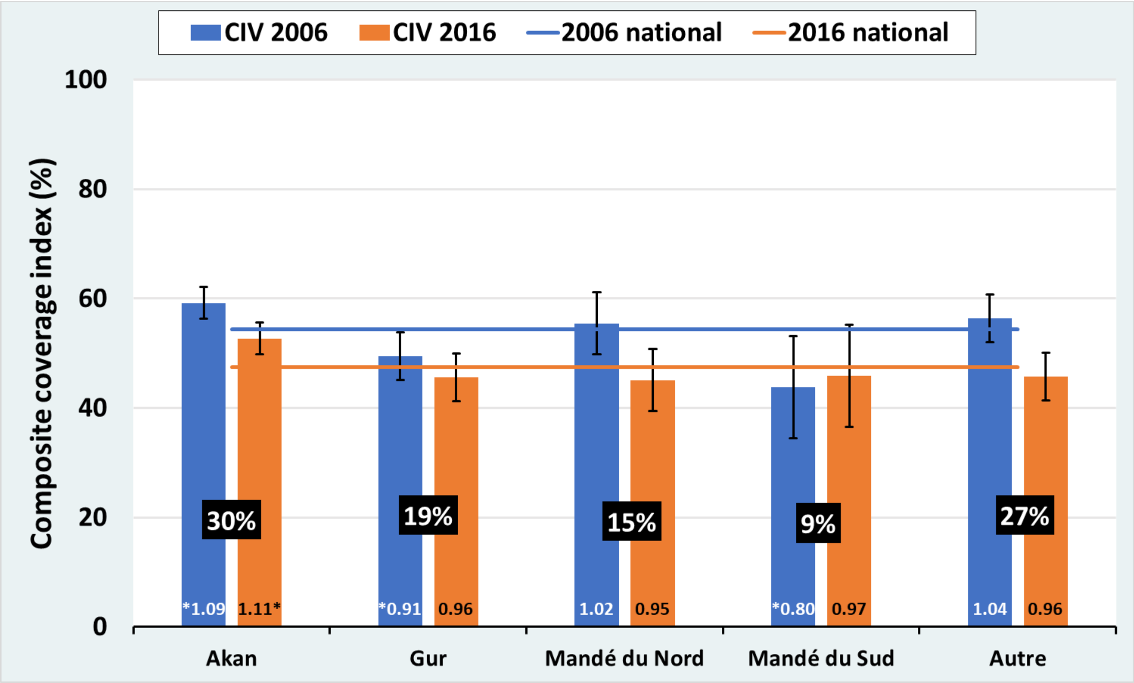

Dominican Republic

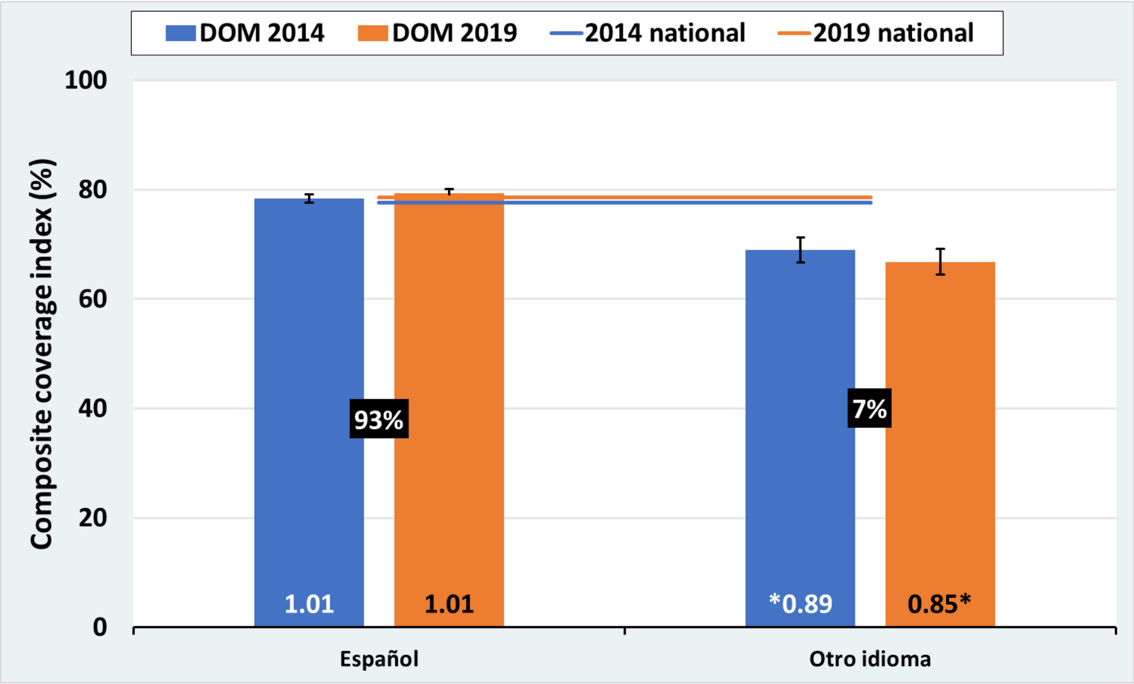

Ethiopia

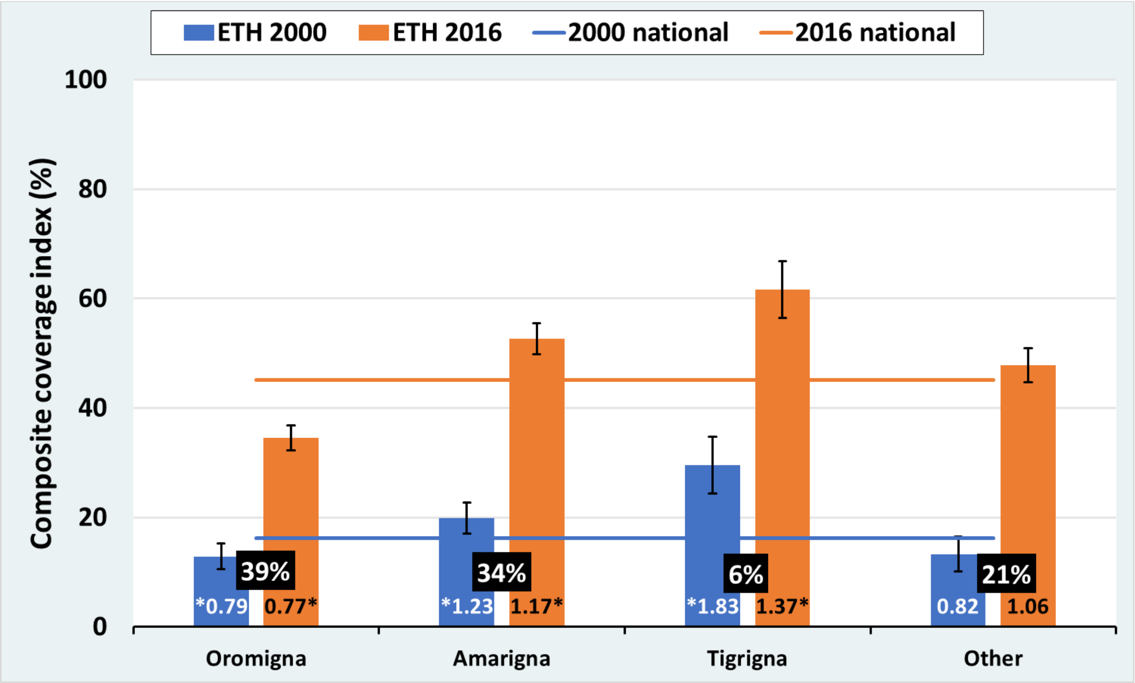

Gabon

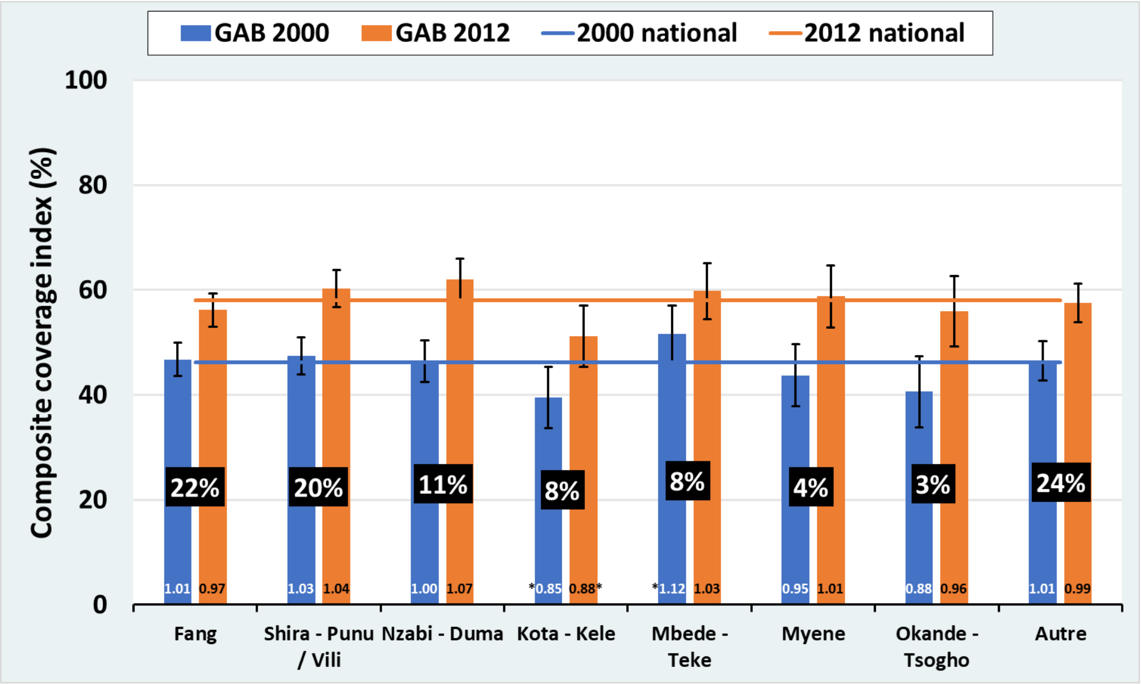

Gambia

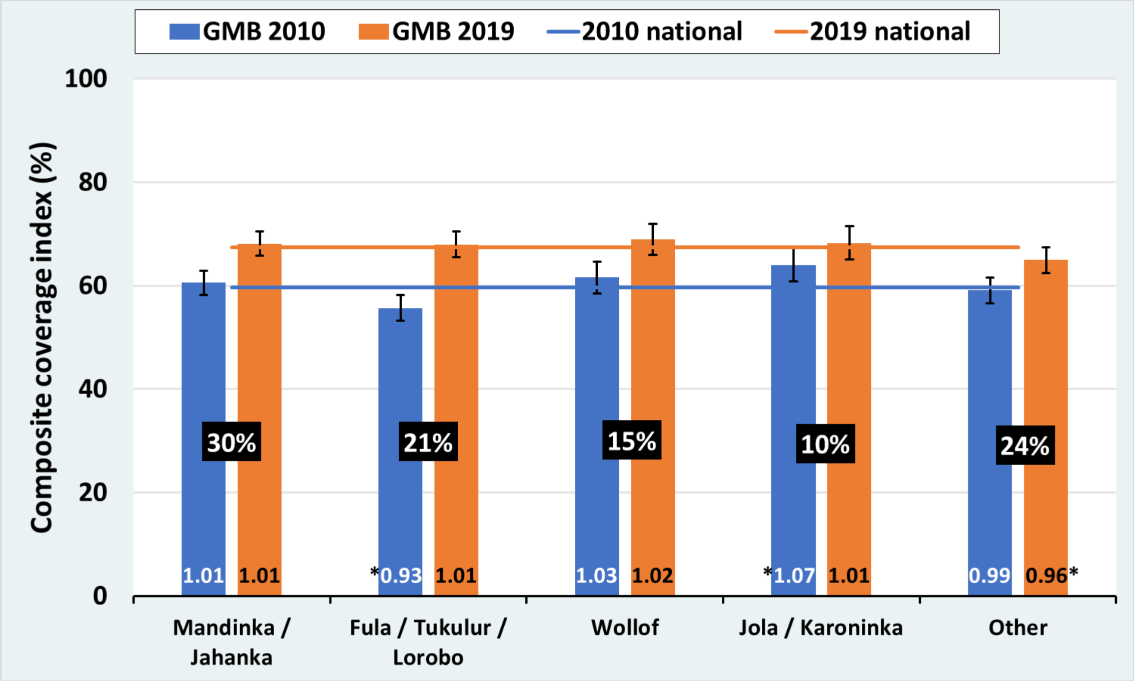

Ghana

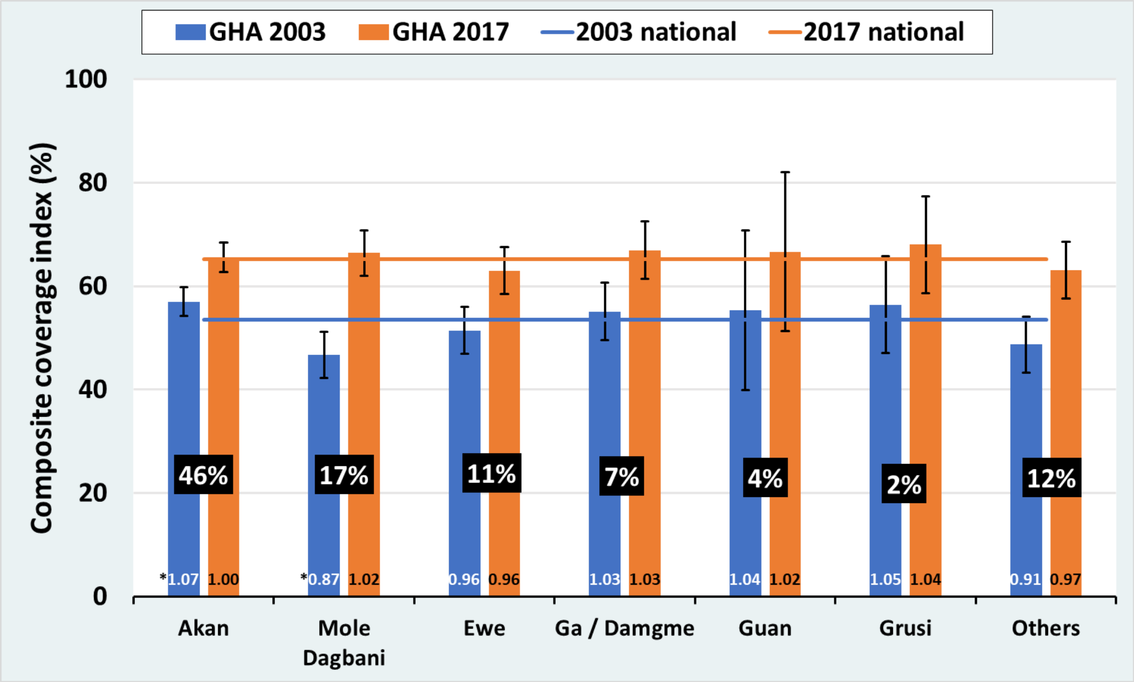

Guinea

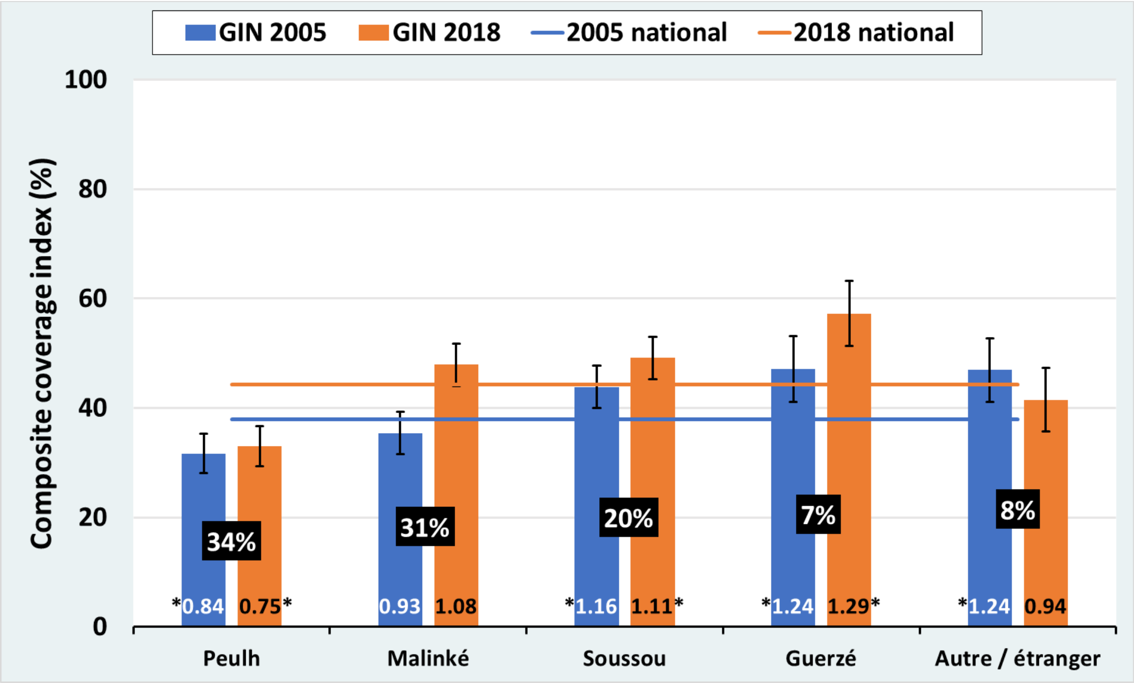

Guinea-Bissau

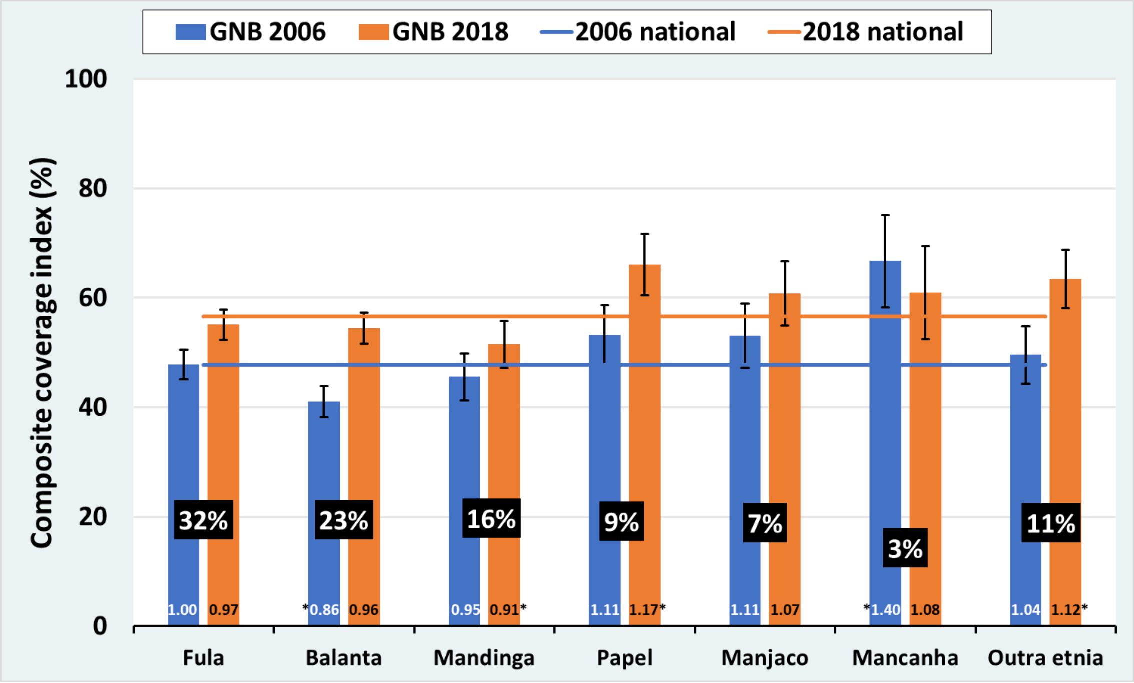

Guyana

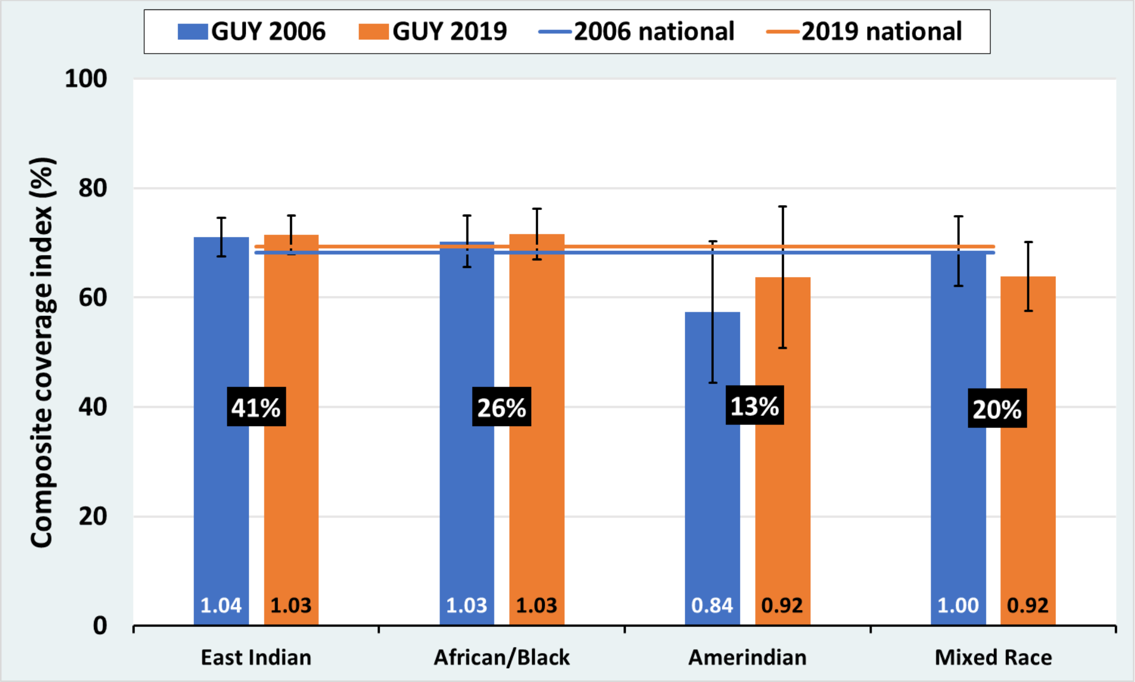

Honduras

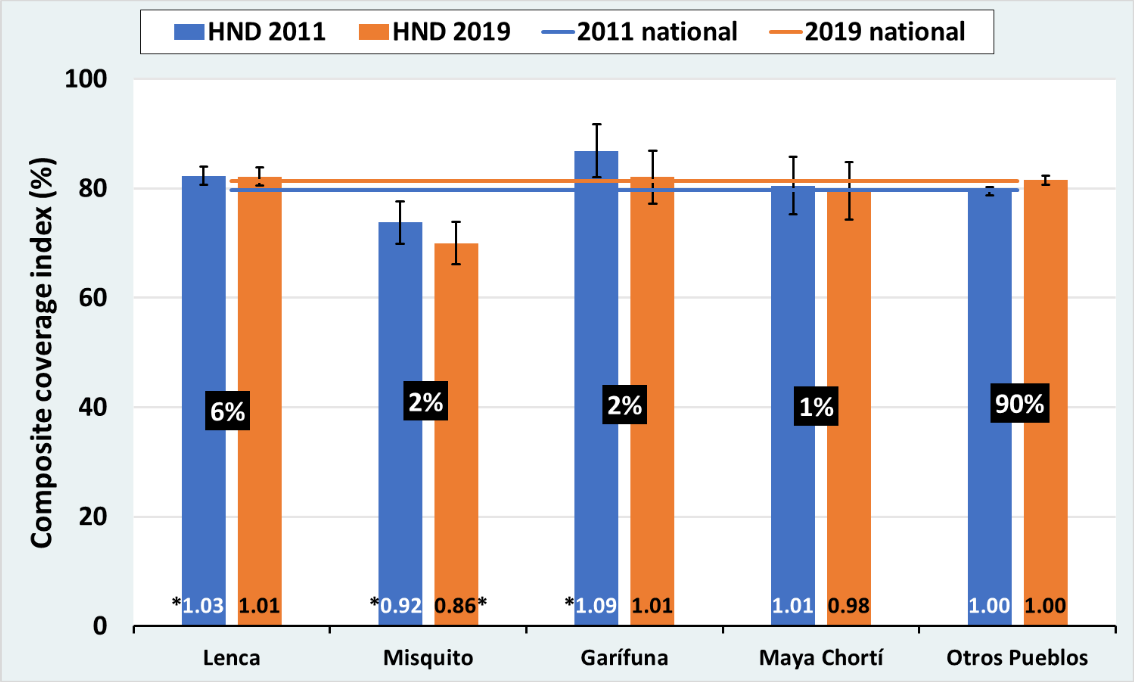

India

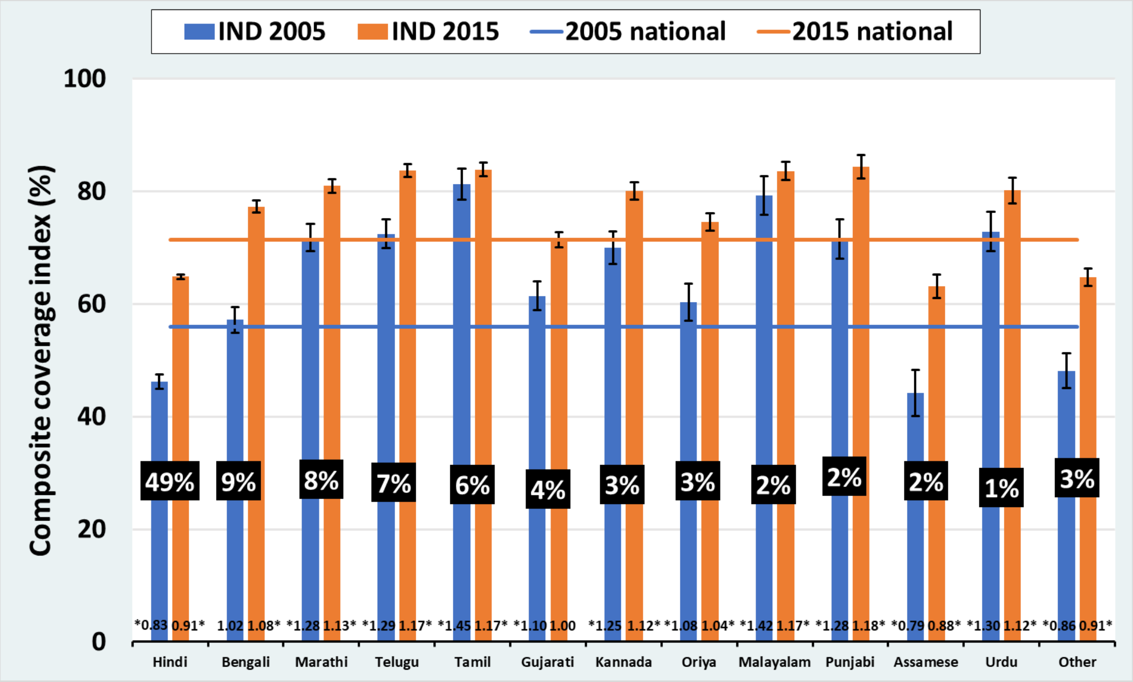
IND 2005
IND 2015
2005 national
2015 national
Composite coverage index (%)
100
80
60
40
20
0
49%
9%
8%
7%
6%
4%
3%
3%
2%
2%
2%
1%
3%
0.83 0.91
1.02 1.08*
1.28 1.13
1.29 1.17
1.45 1.17
*1.10 1.00
1.25 1.12
1.08 1.04
1.42 1.17
1.28 1.18
0.79 0.88
1.30 1.12
0.86 0.91
Hindi
Bengali
Marathi
Telugu
Tamil
Gujarati
Kannada
Oriya
Malayalam
Punjabi
Assamese
Urdu
Other

Kenya

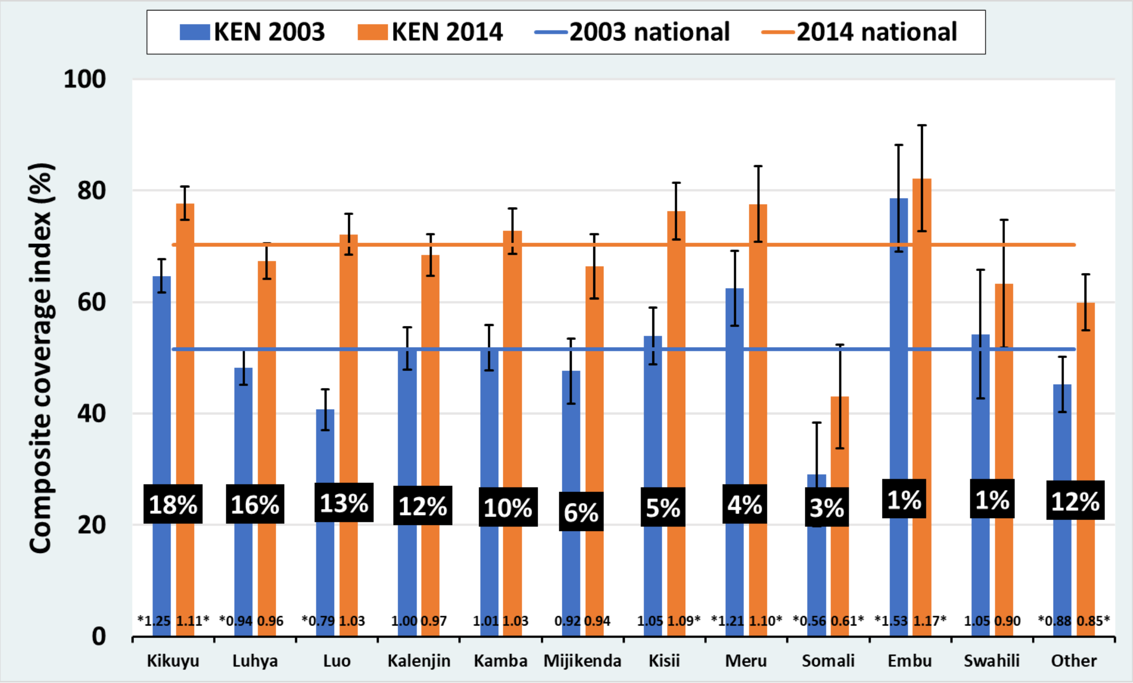
KEN 2003
KEN 2014
2003 national
2014 national
Composite coverage index (%)
100
80
60
40
20
0
18%
16%
13%
12%
10%
6%
5%
4%
3%
1%
1%
12%
1.25 1.11
*0.94 0.96
*0.79 1.03
1.00 0.97
1.01 1.03
0.92 0.94
1.05 1.09*
1.21 1.10
0.56 0.61
1.53 1.17
1.05 0.90
0.88 0.85
Kikuyu
Luhya
Luo
Kalenjin
Kamba
Mijikenda
Kisii
Meru
Somali
Embu
Swahili
Other

Kyrgyzstan

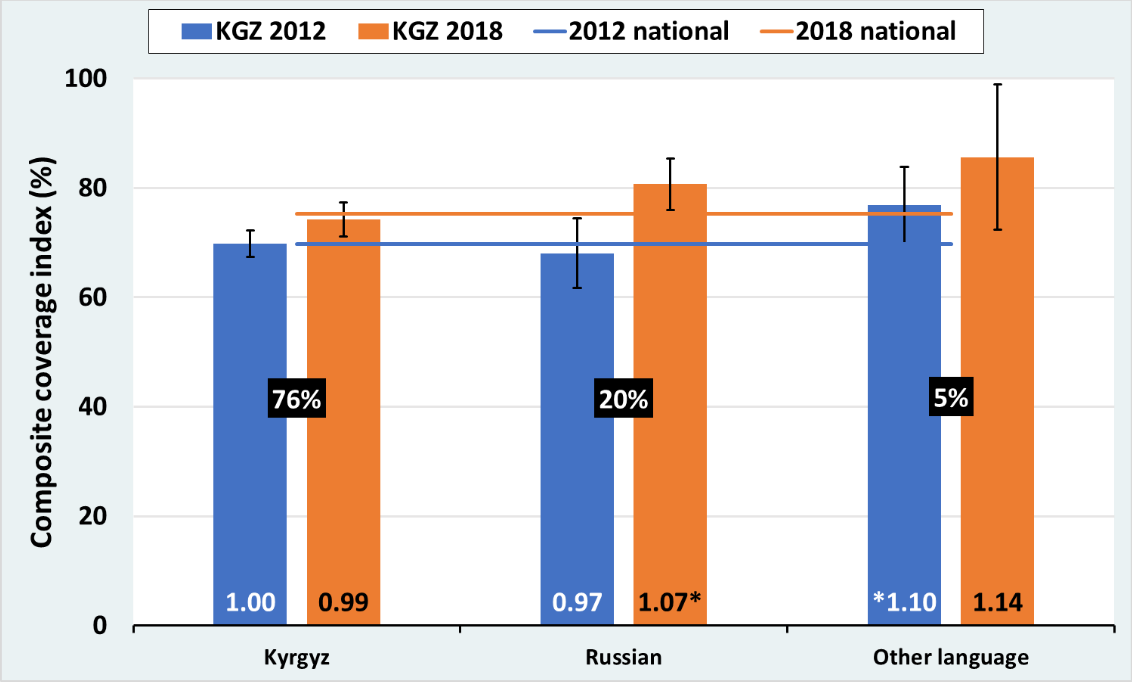

Lao PDR

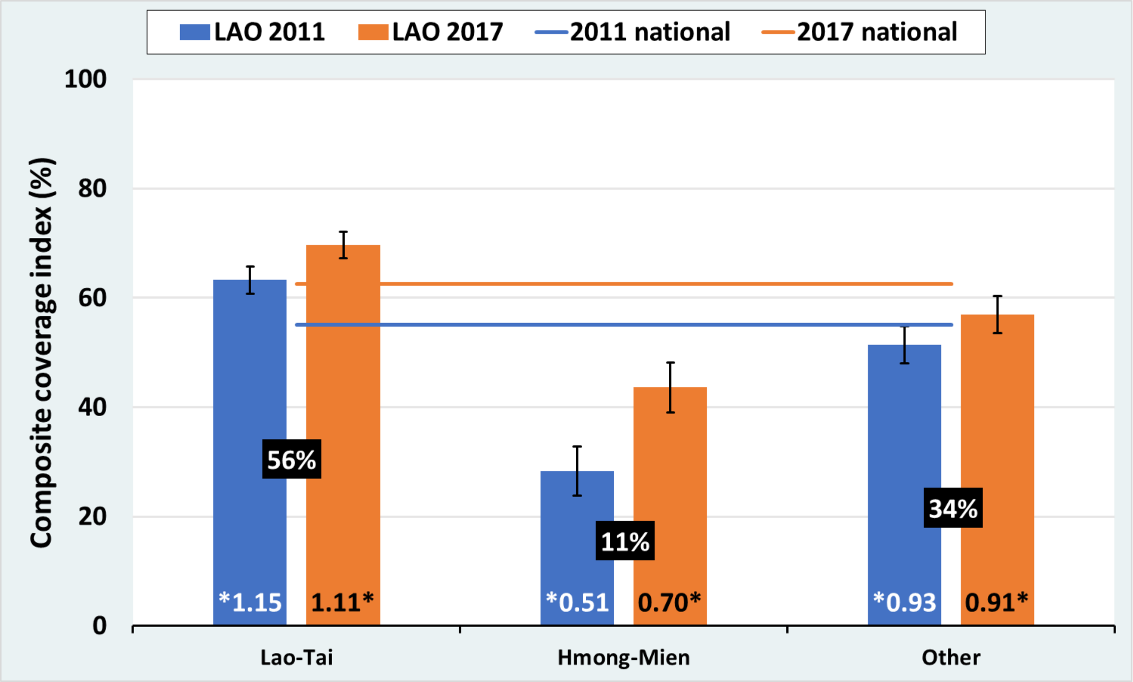

Malawi

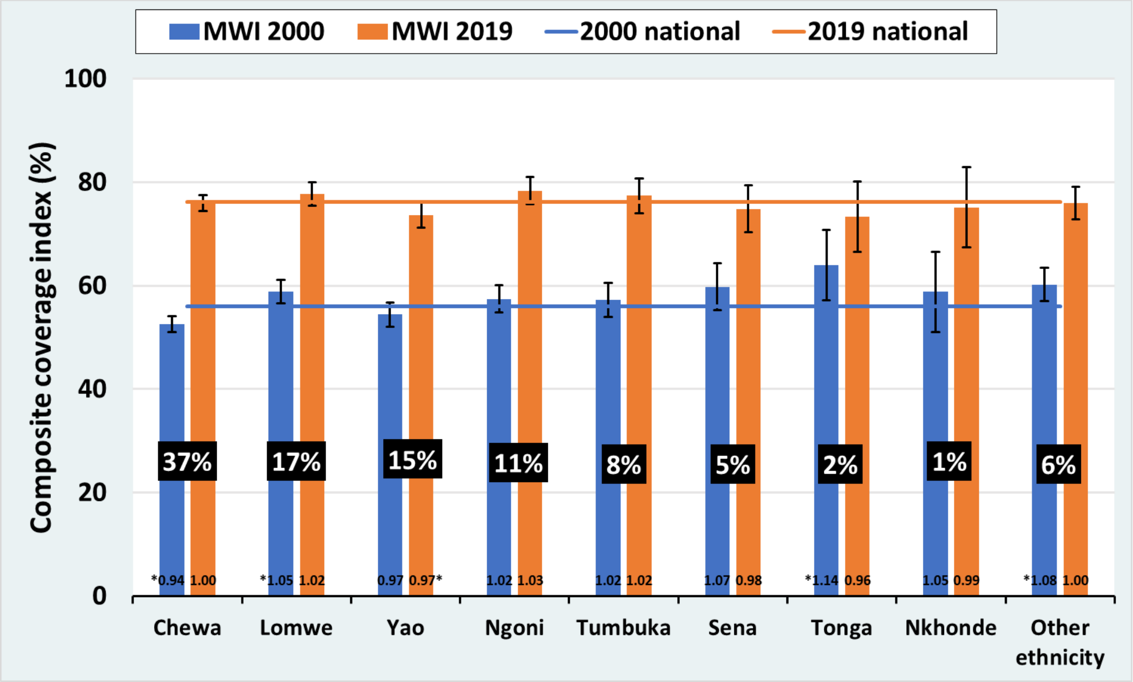
MWI 2000
MWI 2019
2000 national
2019 national
Composite coverage index (%)
100
80
60
40
20
0
37%
17%
15%
11%
8%
5%
2%
1%
6%
*0.94 1.00
*1.05 1.02
0.97 0.97*
1.02 1.03
1.02 1.02
1.07 0.98
*1.14 0.96
1.05 0.99
*1.08 1.00
Chewa
Lomwe
Yao
Ngoni
Tumbuka
Sena
Tonga
Nkhonde
Other ethnicity

Mali

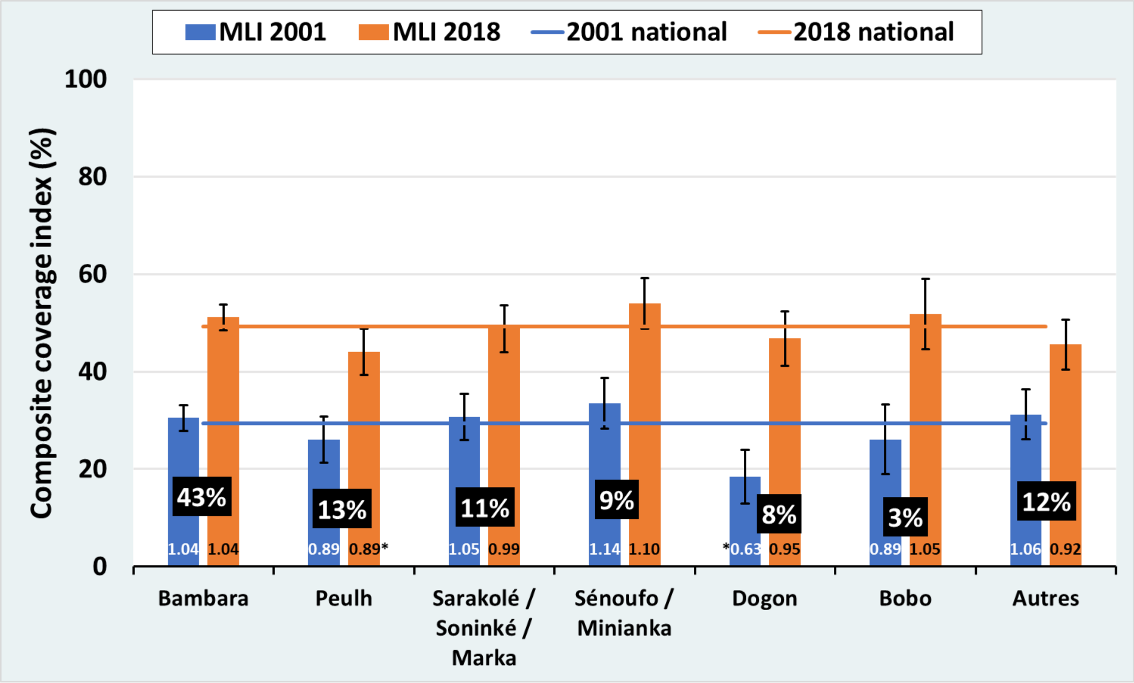
MLI 2001
MLI 2018
2001 national
2018 national
Composite coverage index (%)
100
80
60
40
20
0
43%
13%
11%
9%
8%
3%
12%
1.04 1.04
0.89 0.89*
1.05 0.99
1.14 1.10
*0.63 0.95
0.89 1.05
1.06 0.92
Bambara
Peulh
Sarakolé / Soninké / Marka
Sénoufo / Minianka
Dogon
Bobo
Autres

Mauritania

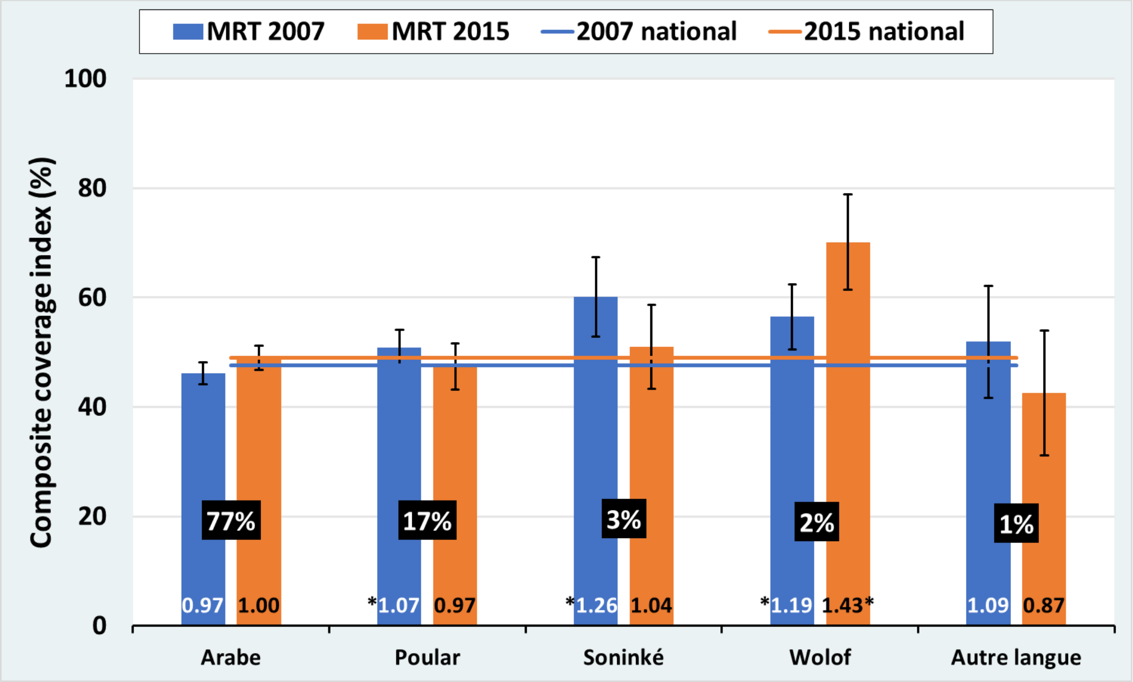

Moldova

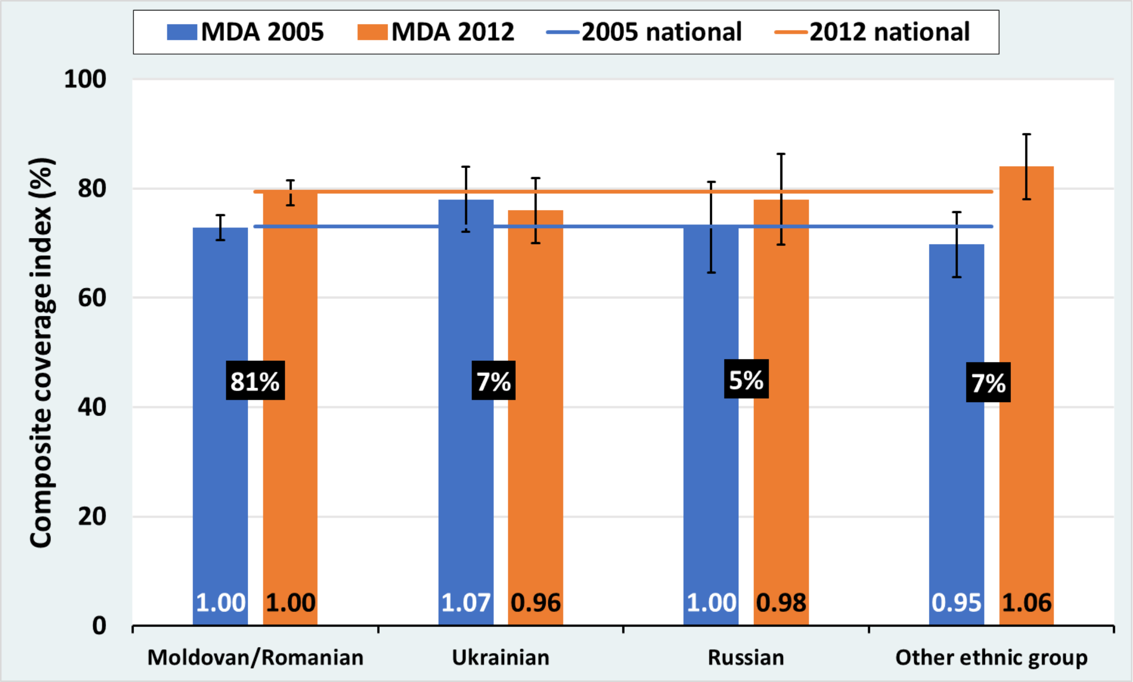

Mongolia

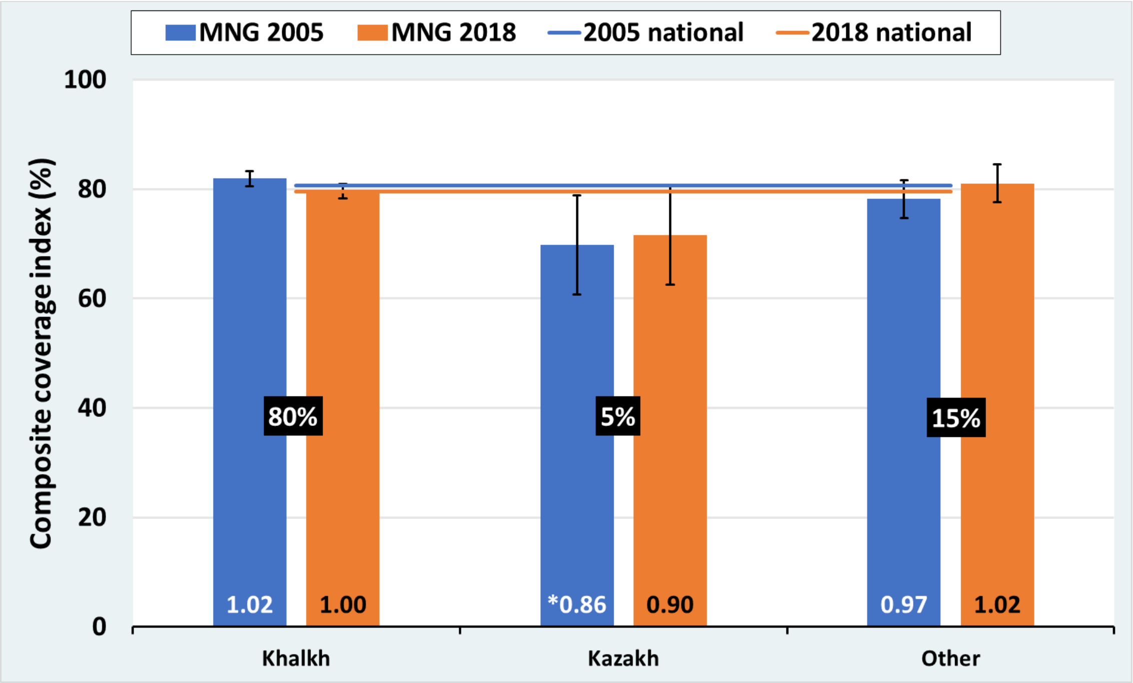

Mozambique

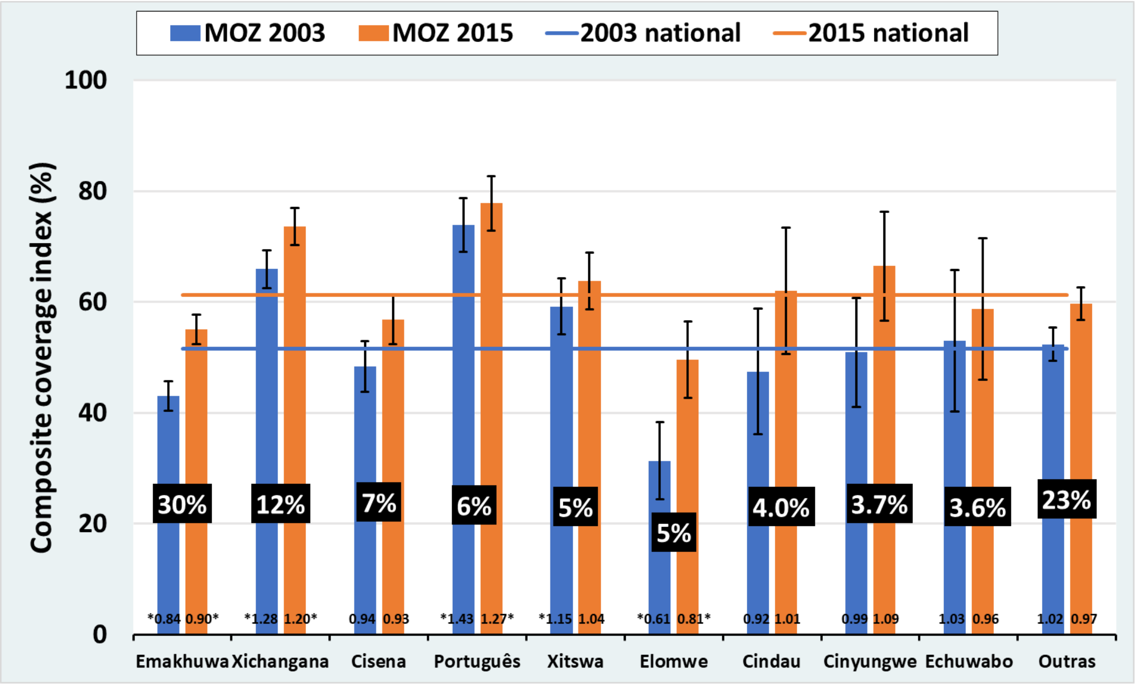

Namibia

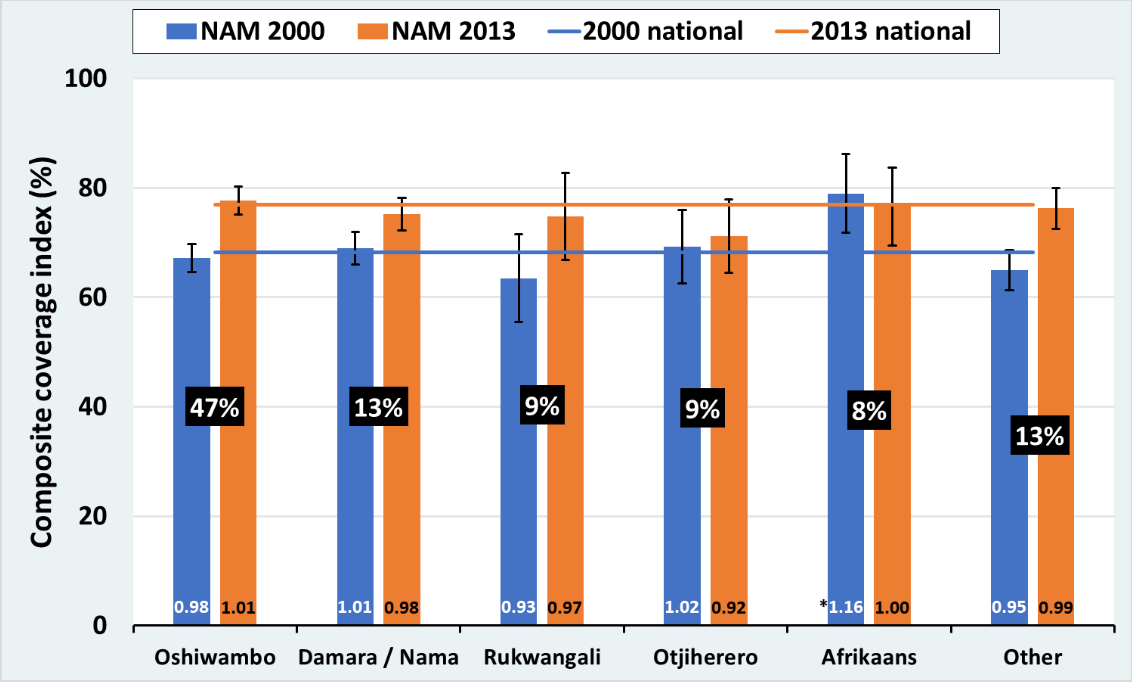

Nepal

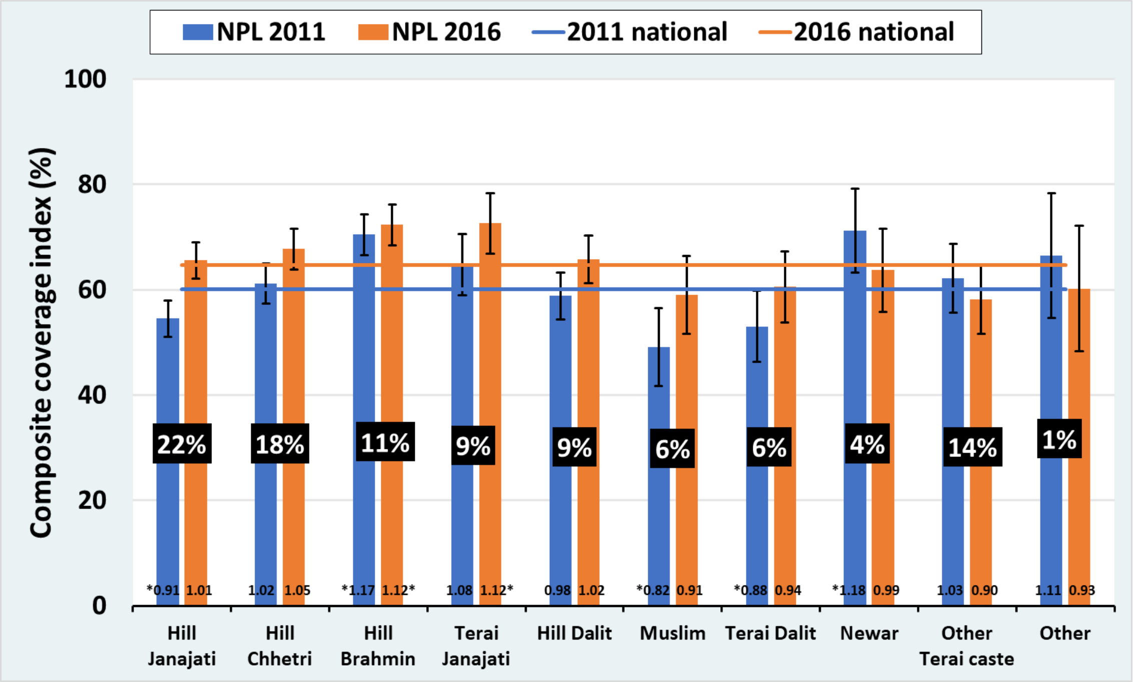

Niger

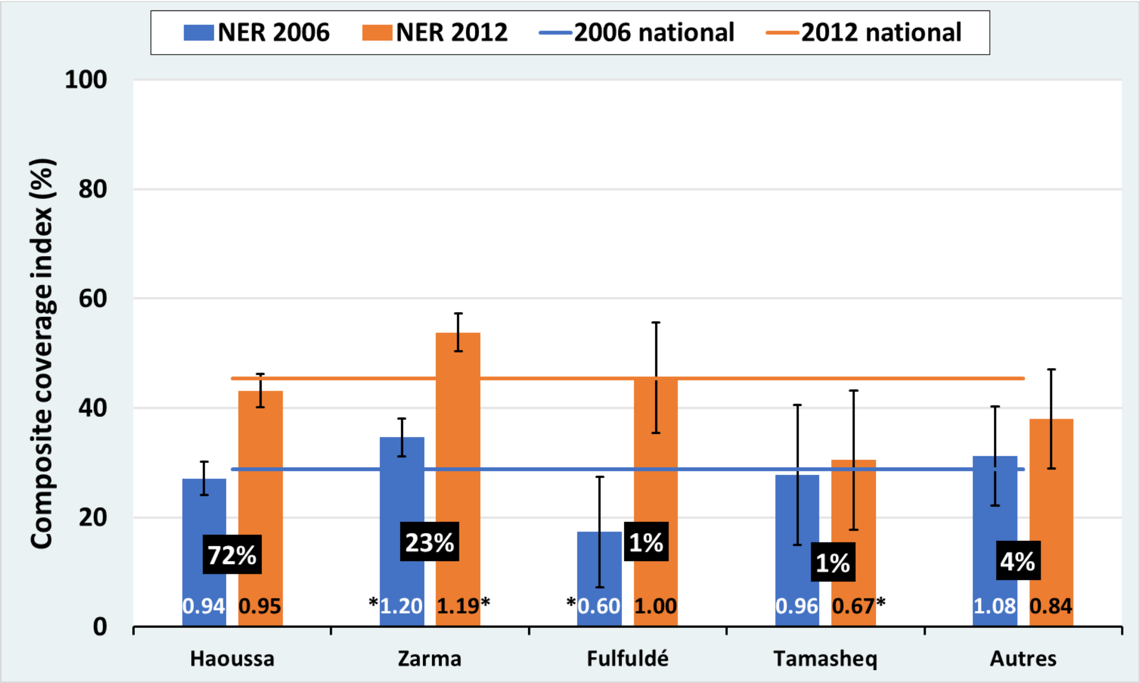

Nigeria

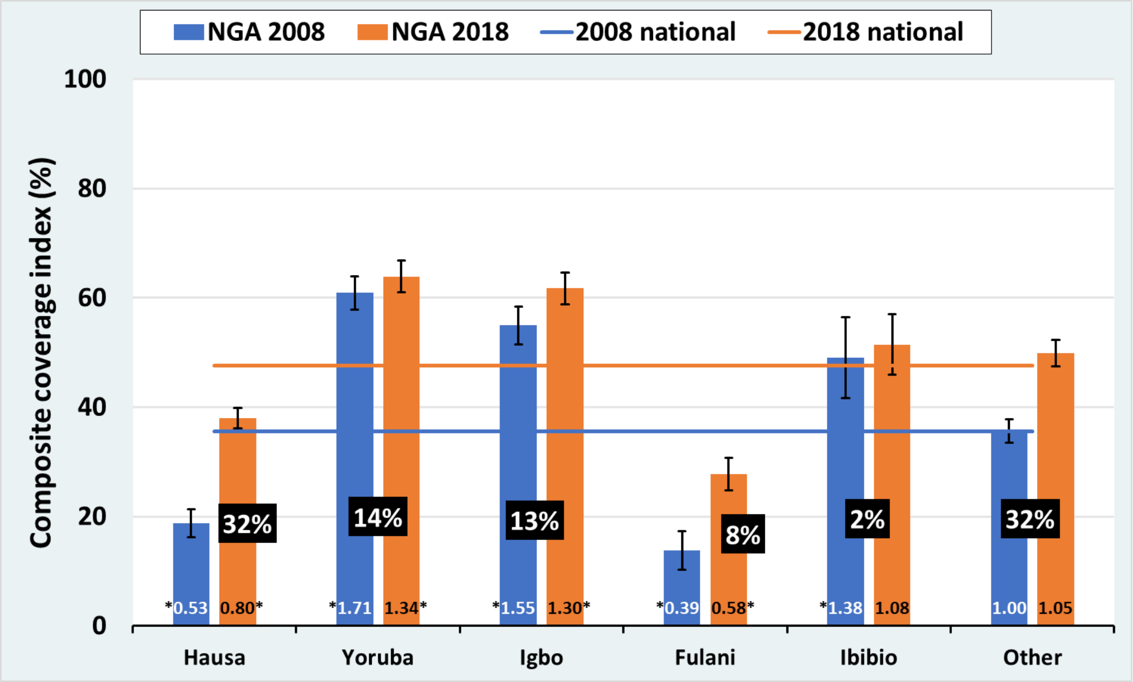

North Macedonia

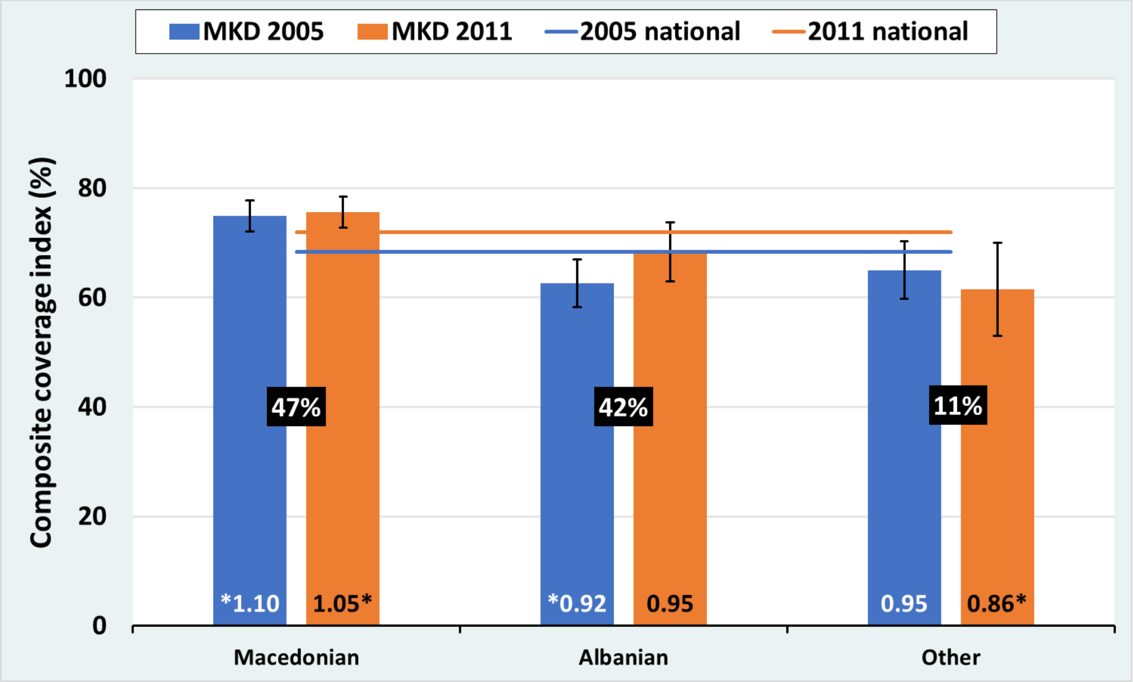

Pakistan

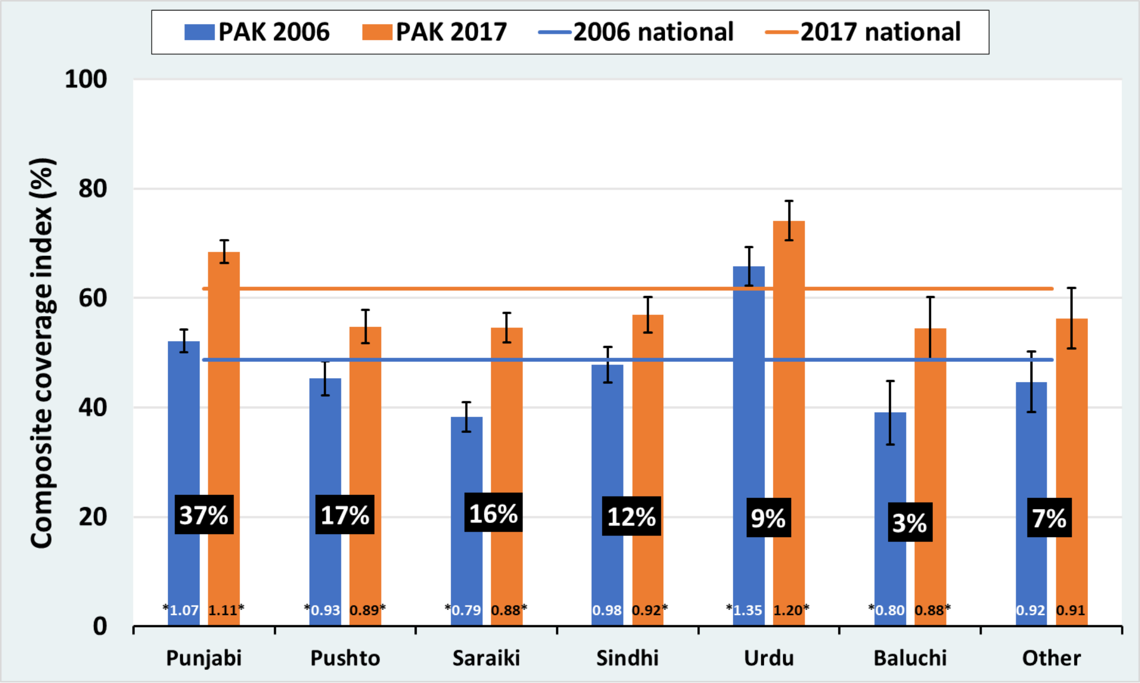

Peru

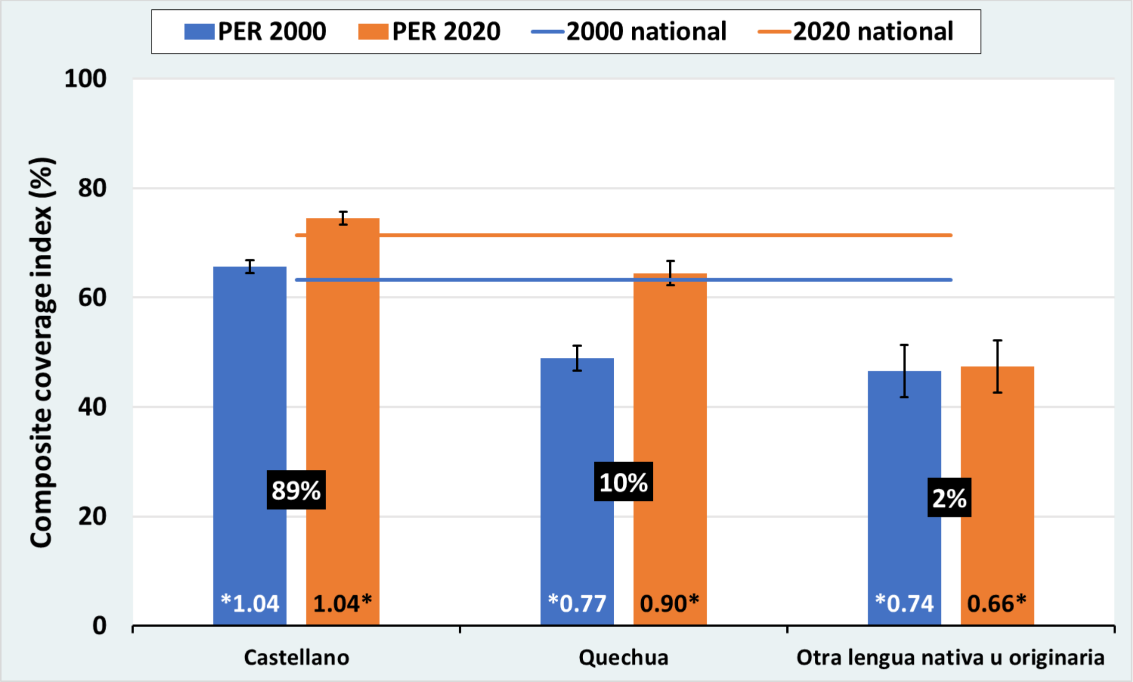

Philippines

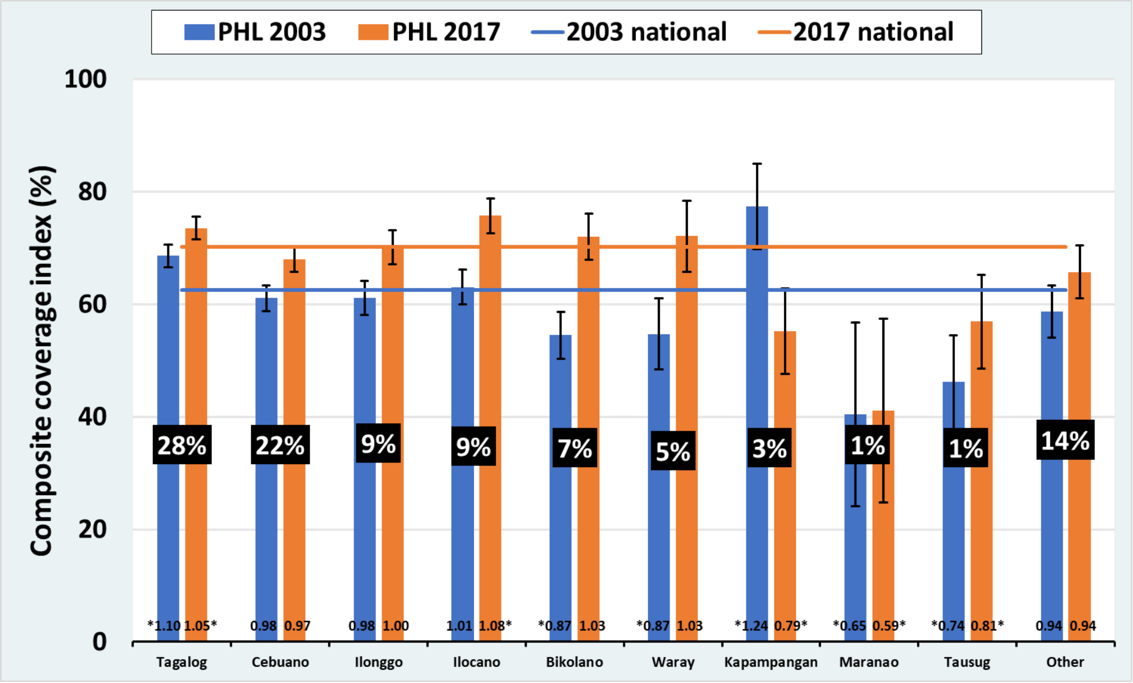

Sao Tome and Principe

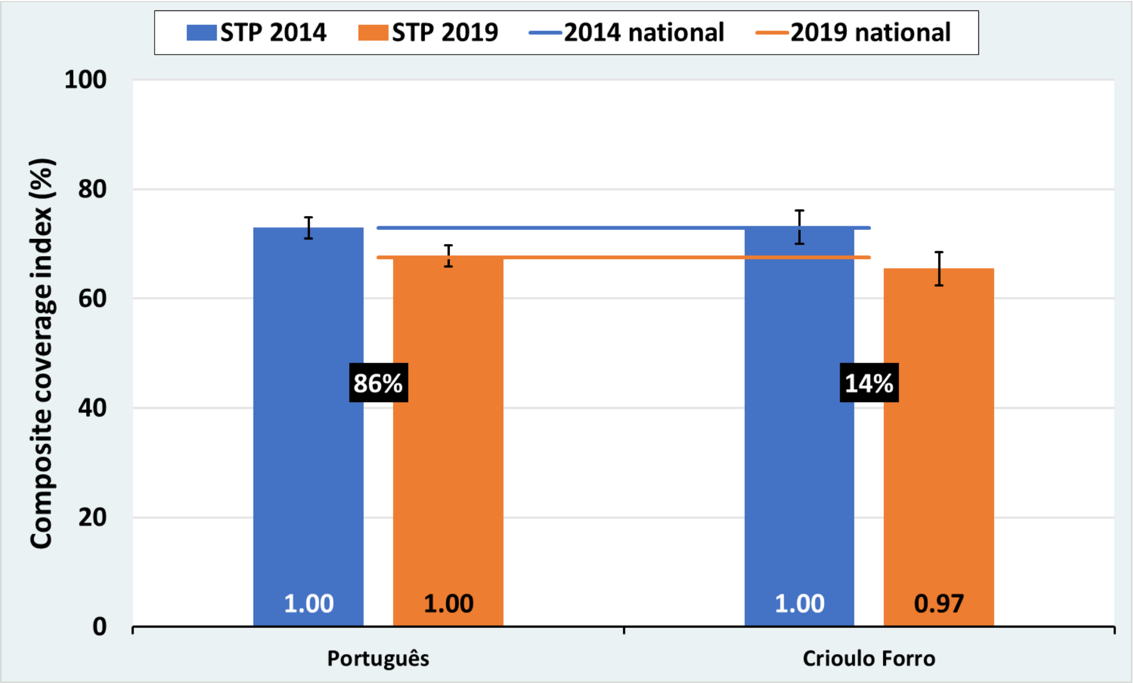

Senegal

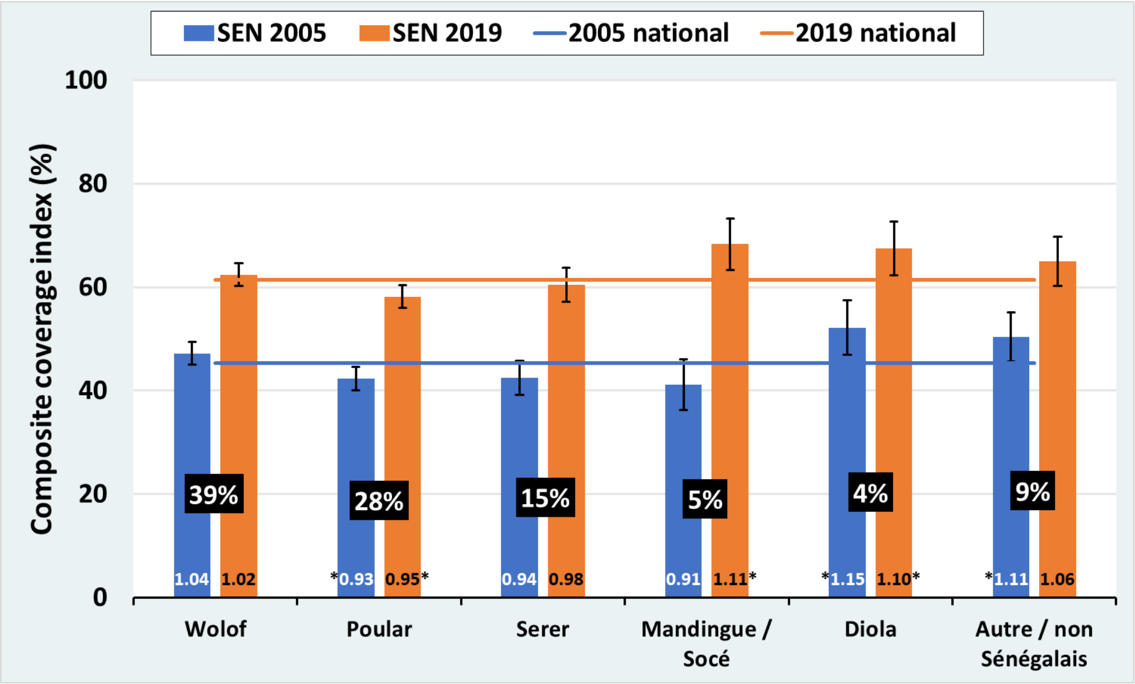

Sierra Leone

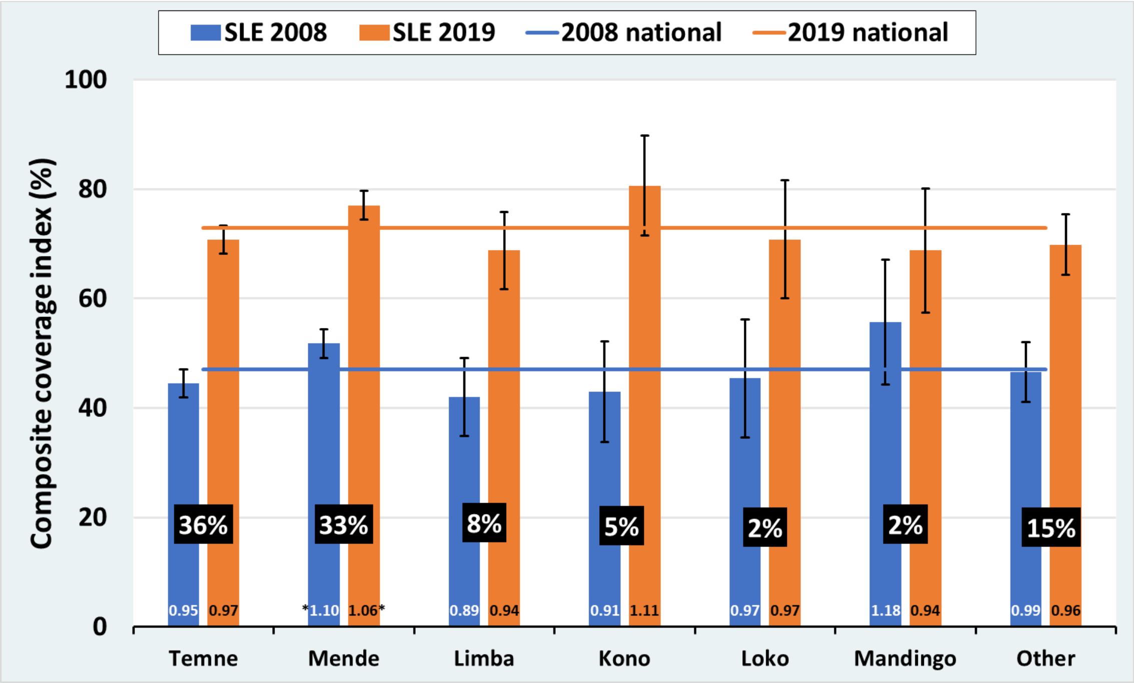

Tajikistan

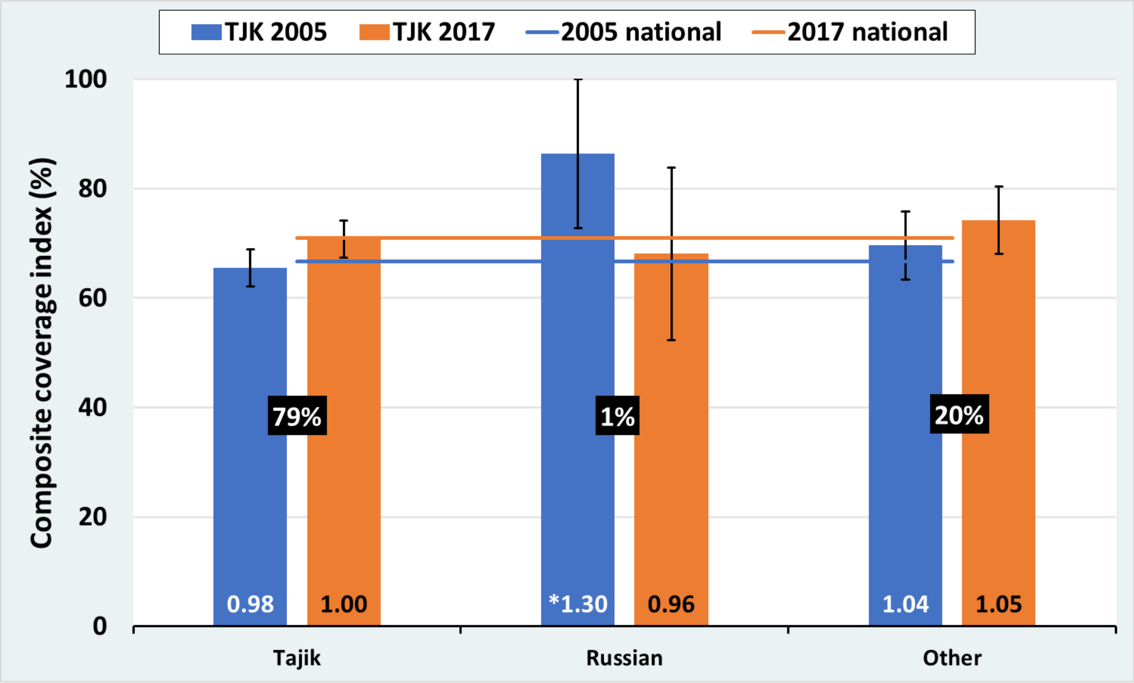

Timor-Leste

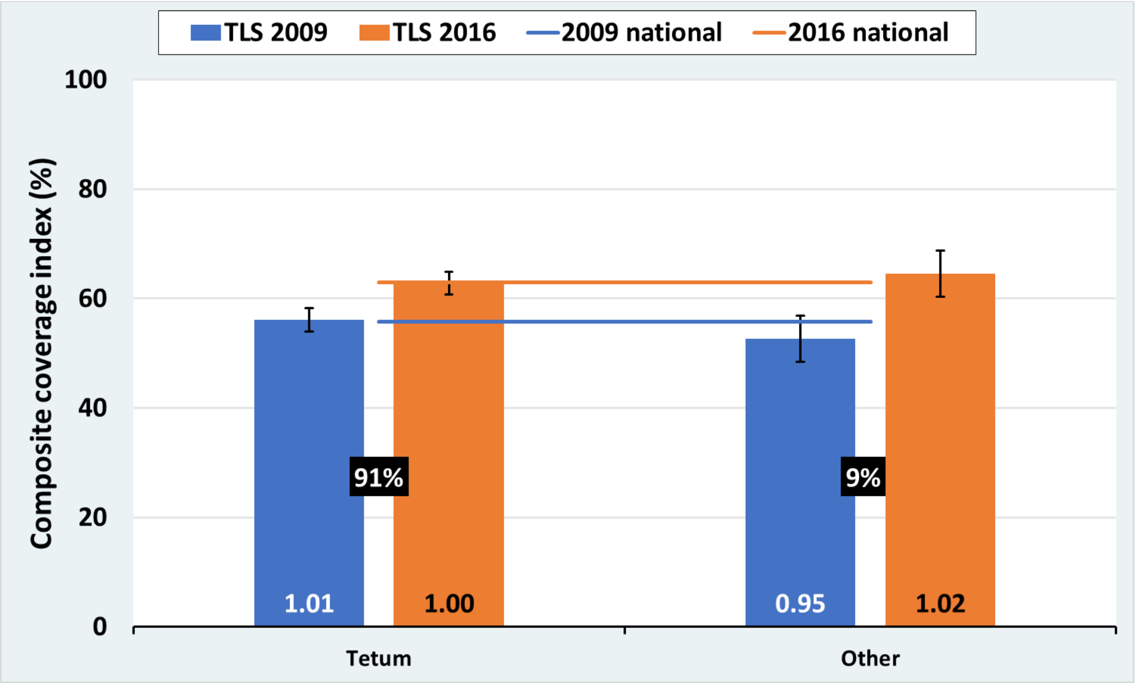

Togo

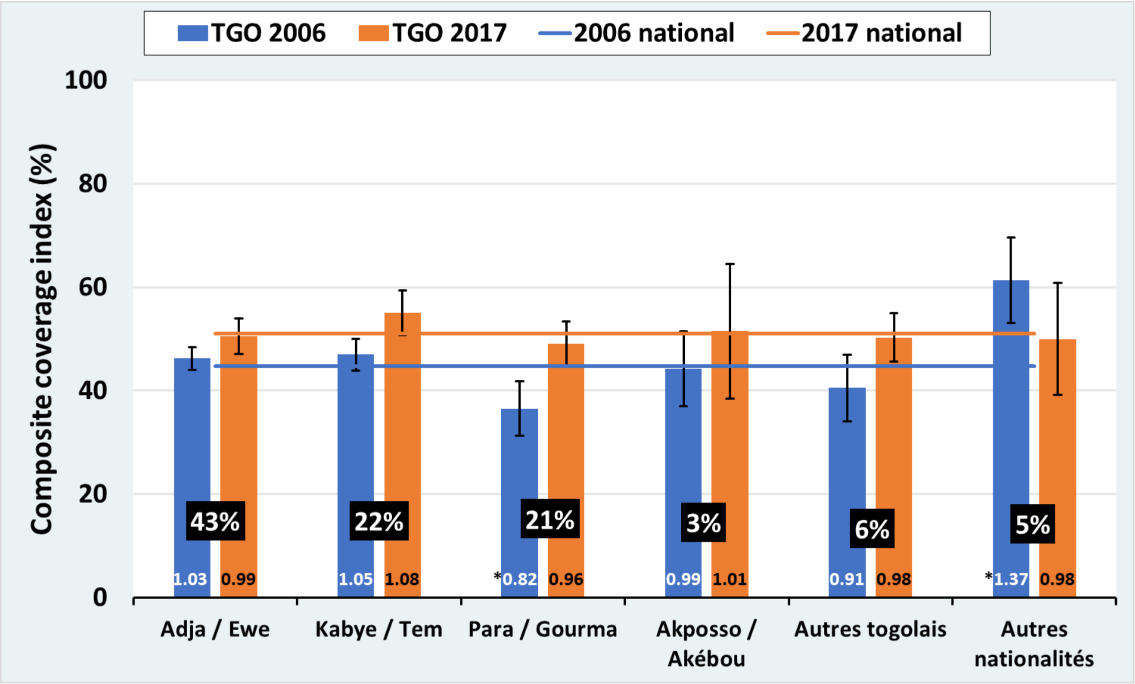

Turkmenistan

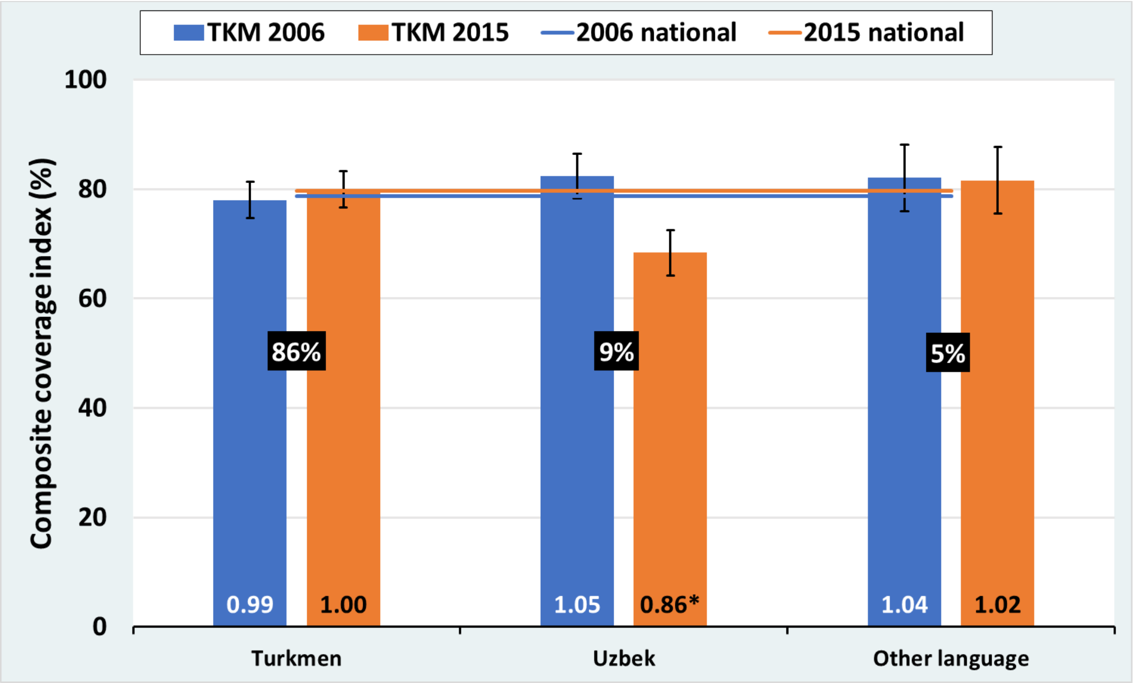

Uganda

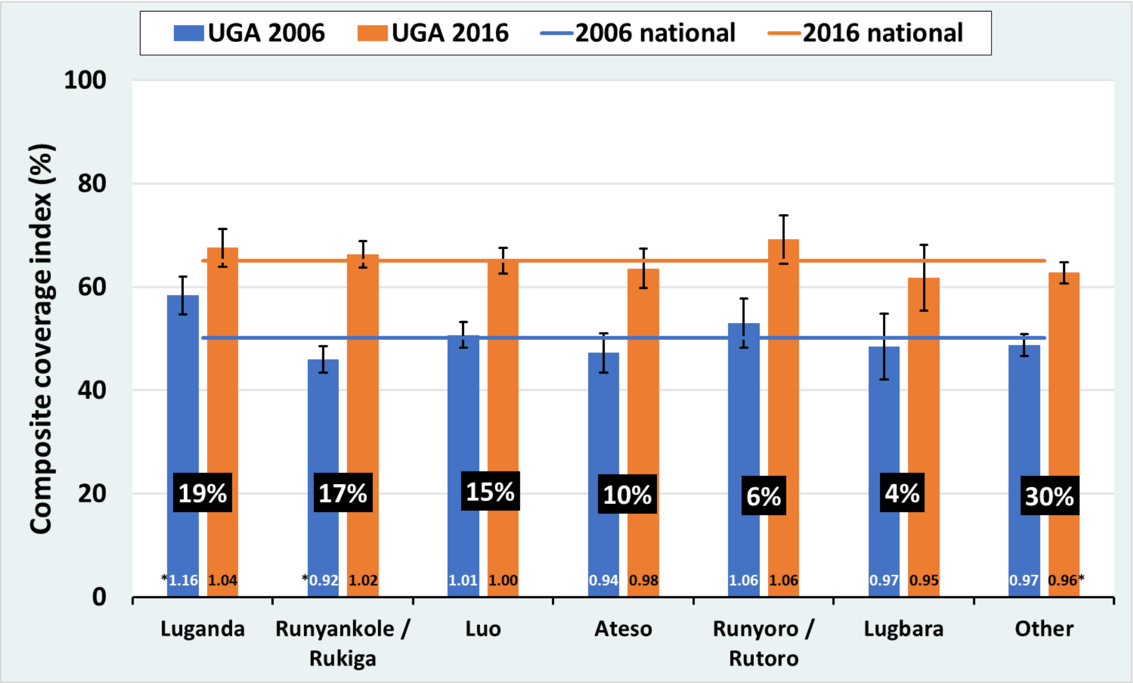

Vietnam

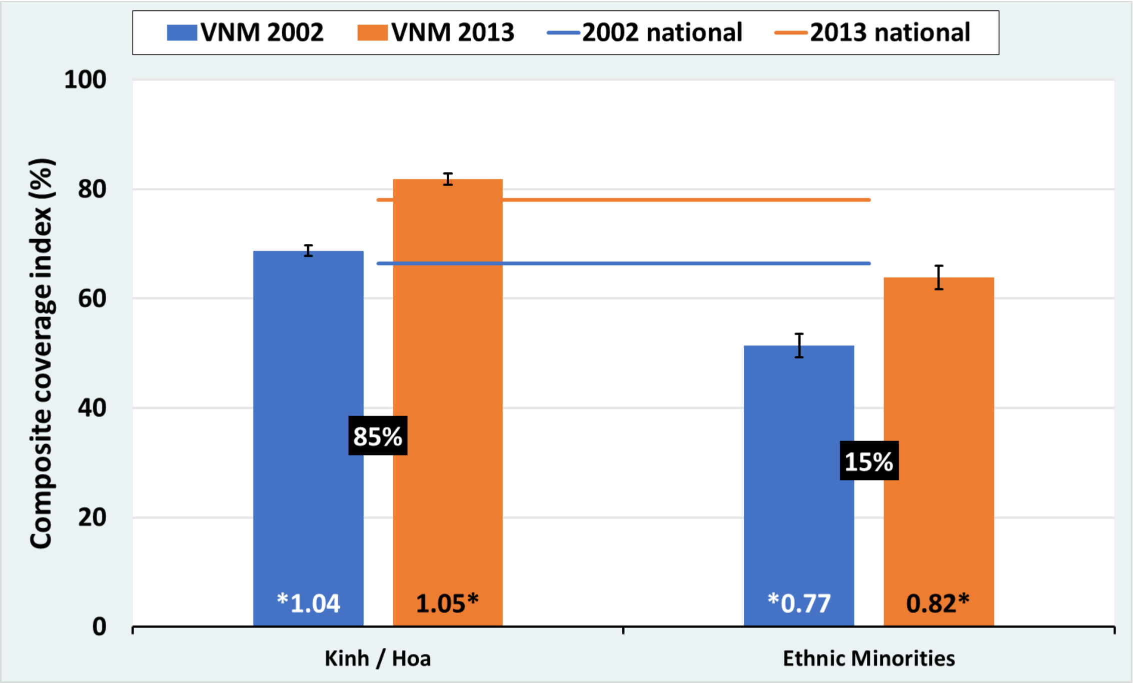

Zambia

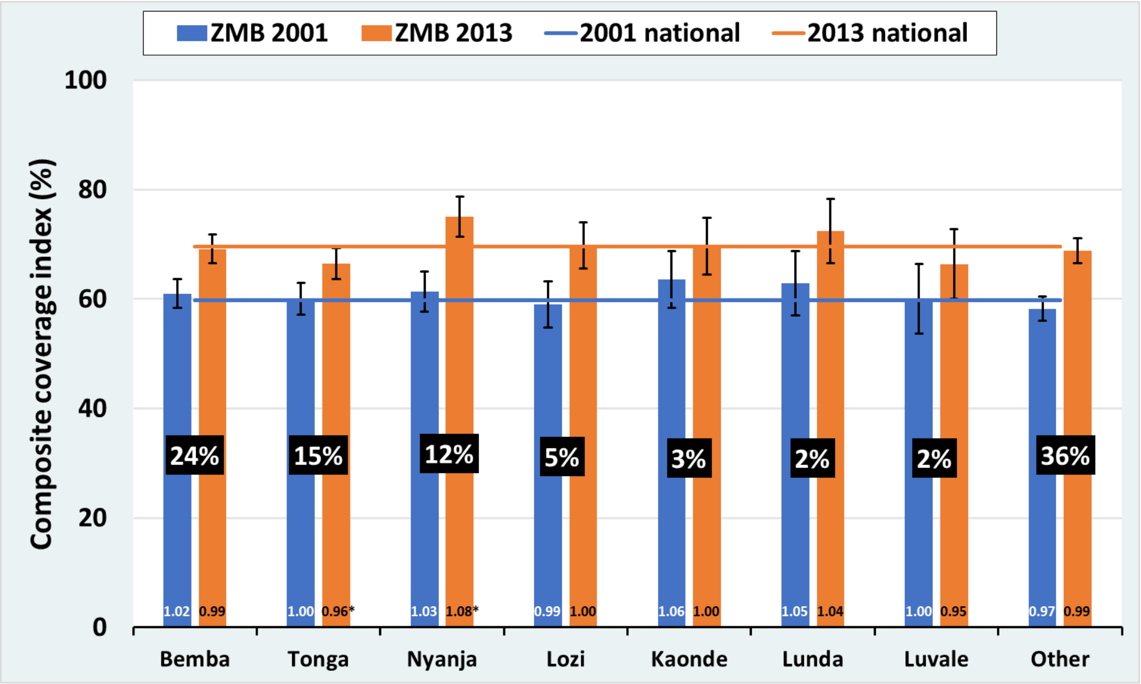

Supplement: Supplementary file 5 — Additional file 5. Title: Composite coverage index by ethnic groups in the first and last surveys. Results for selected countries. The numbers in the black rectangles show the average proportion of the samples for each ethnic group in the two surveys. The numbers at the bottom of the bars show the ratio between the rate in a particular ethnic group and the national rate for that point in time. Description: One graph for each country showing the composite coverage index (CCI) by ethnic groups in the first and last surveys. [file 12939_2023_1888_MOESM5_ESM.pdf]
