# Supplementary material for: Time trends in ethnic inequalities in child health and nutrition: analysis of 59 low and middle-income countries
Source: Int J Equity Health. 2023 Apr 28;22:76. doi: 10.1186/s12939-023-01888-5 (PMC10148503; doi:10.1186/s12939-023-01888-5)

Supplementary figures 3. Under-five mortality rate by ethnic groups in the first and last surveys. Results for selected countries. The numbers in the black rectangles show the average proportion of the samples for each ethnic groups in the two surveys. The numbers at the bottom of the bars show the ratio between the rate in a particular ethnic group and the national rate for that point in time (* statistically significant in relation to the respective national average).

Albania

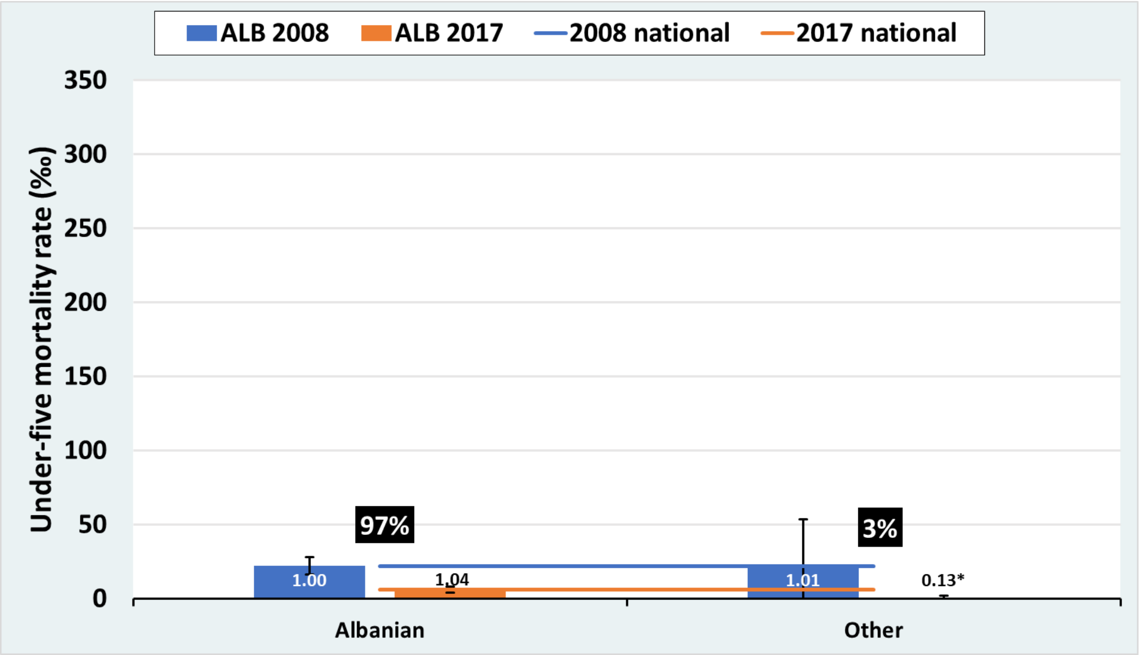

Benin

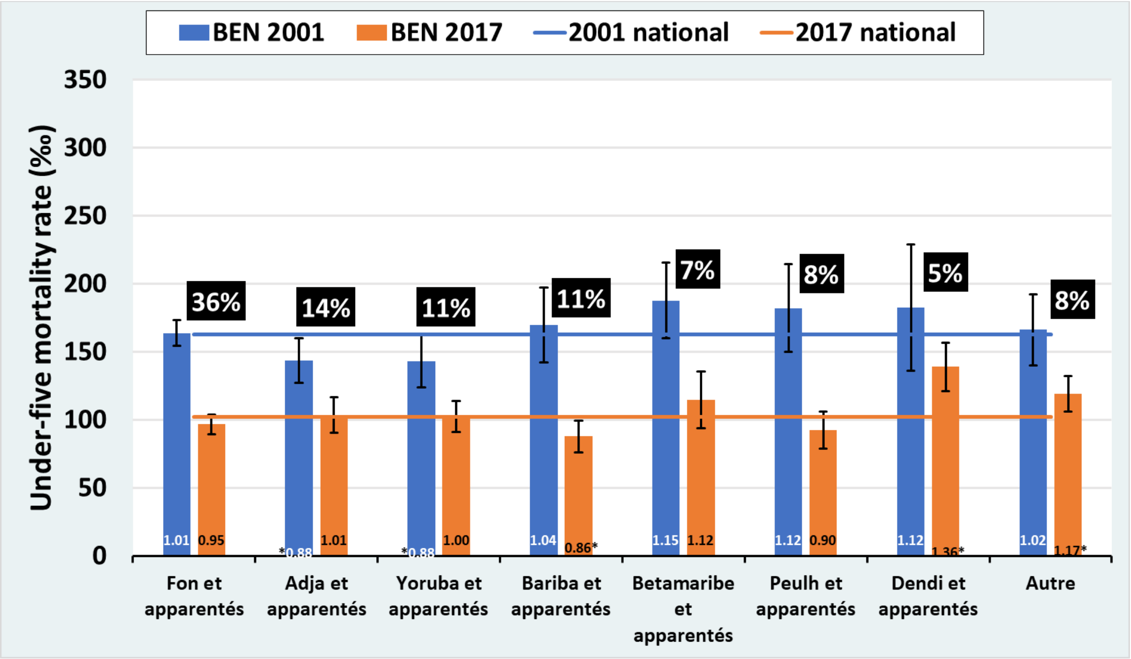

Burkina Faso

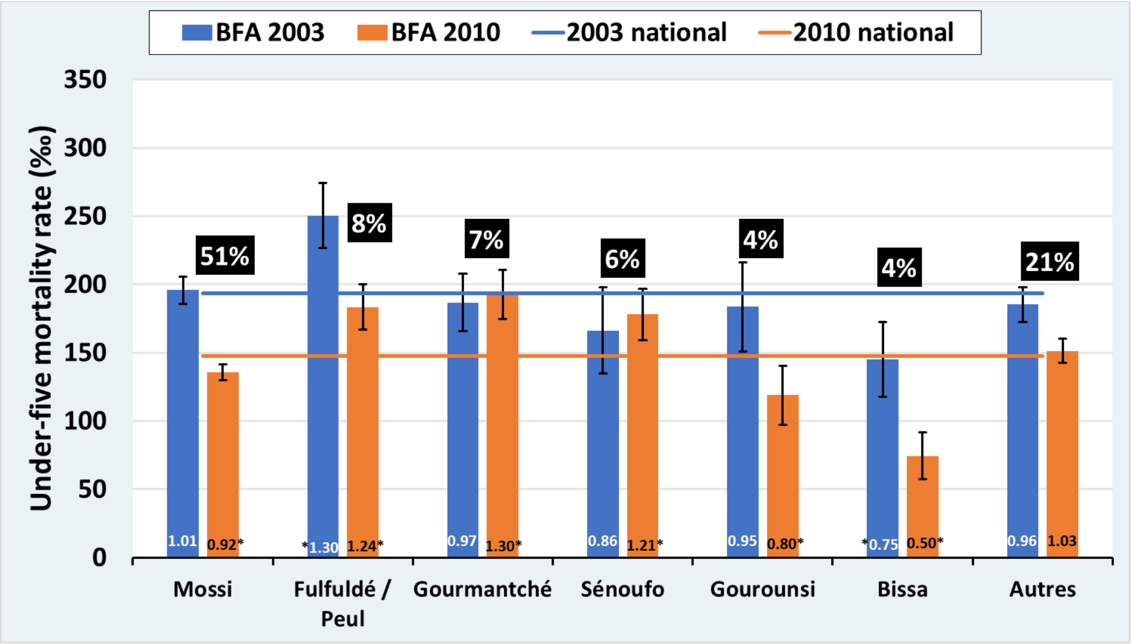

Cameroon

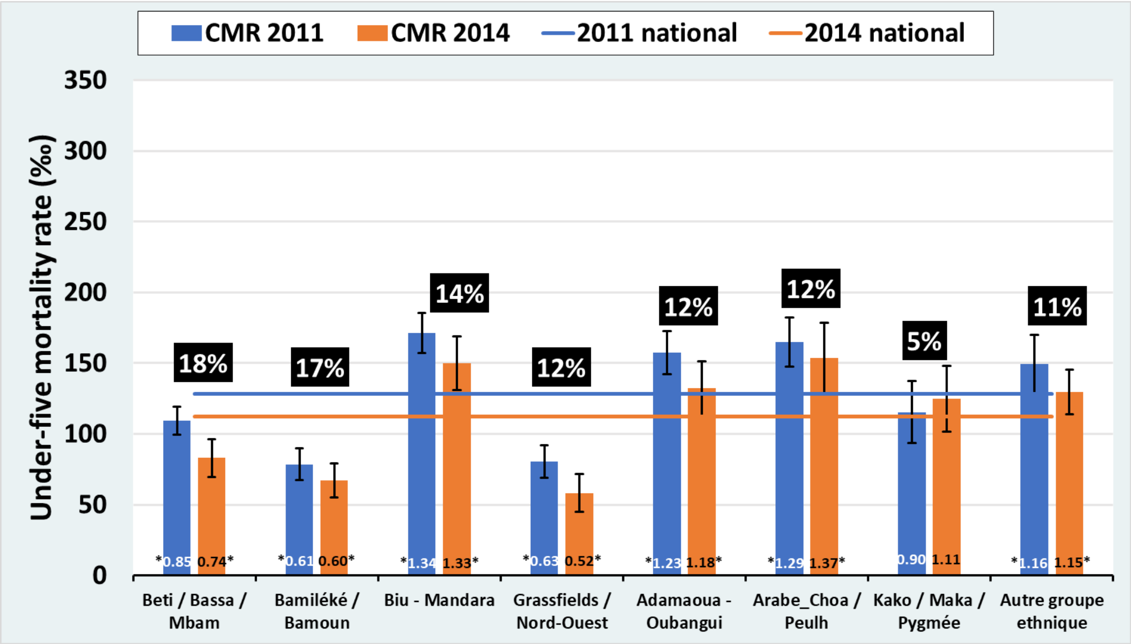

Chad

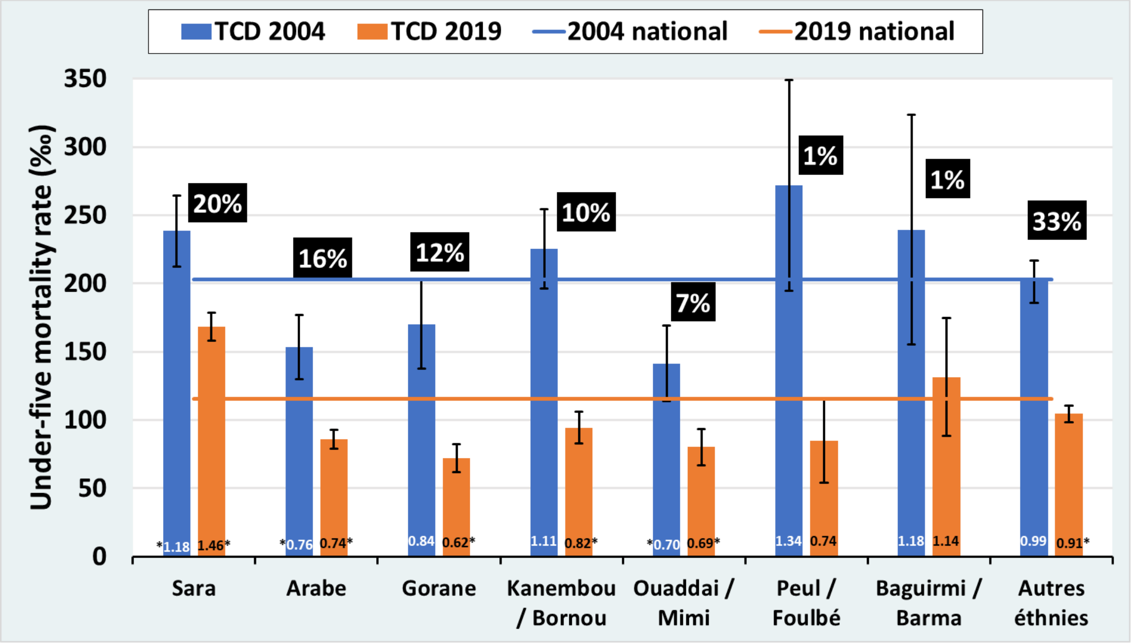

Colombia

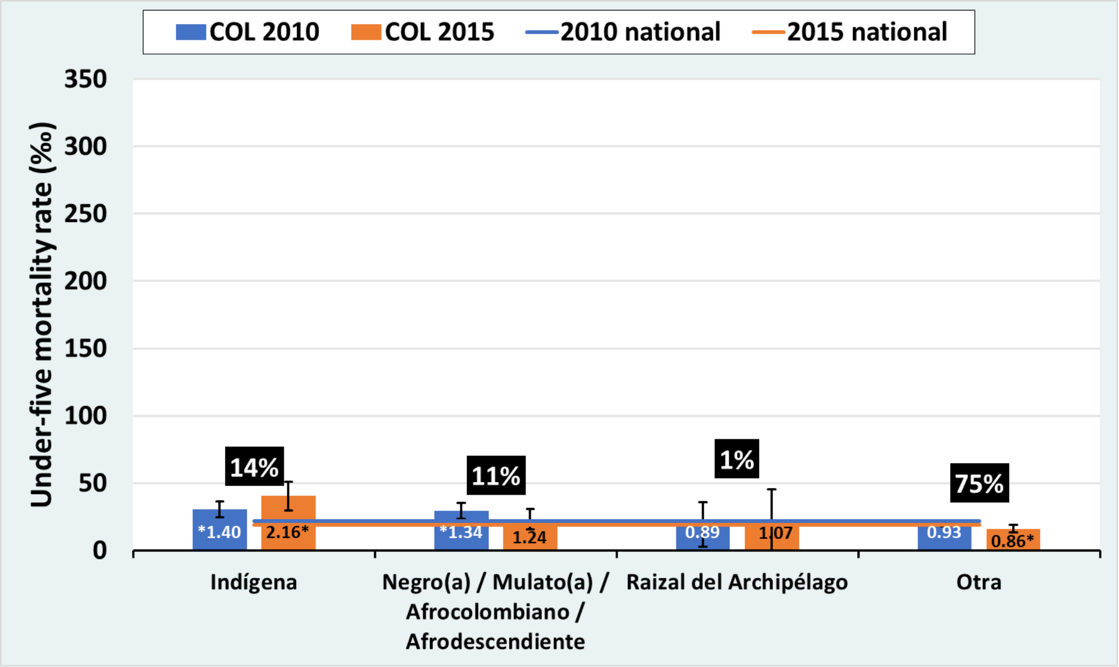

Congo DR

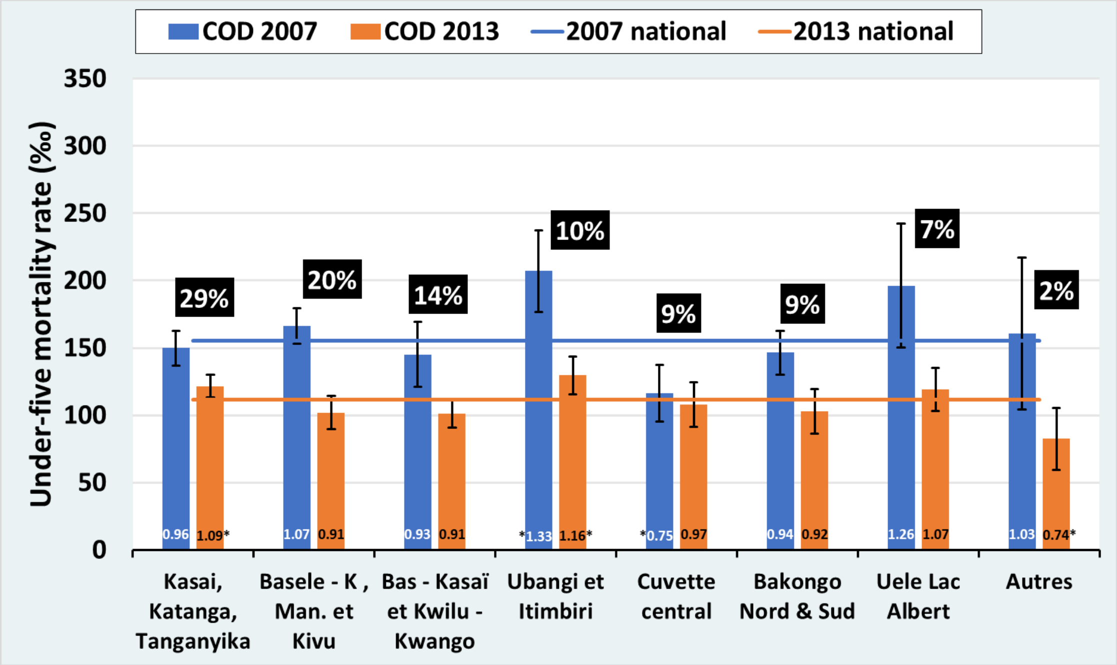

Congo Republic

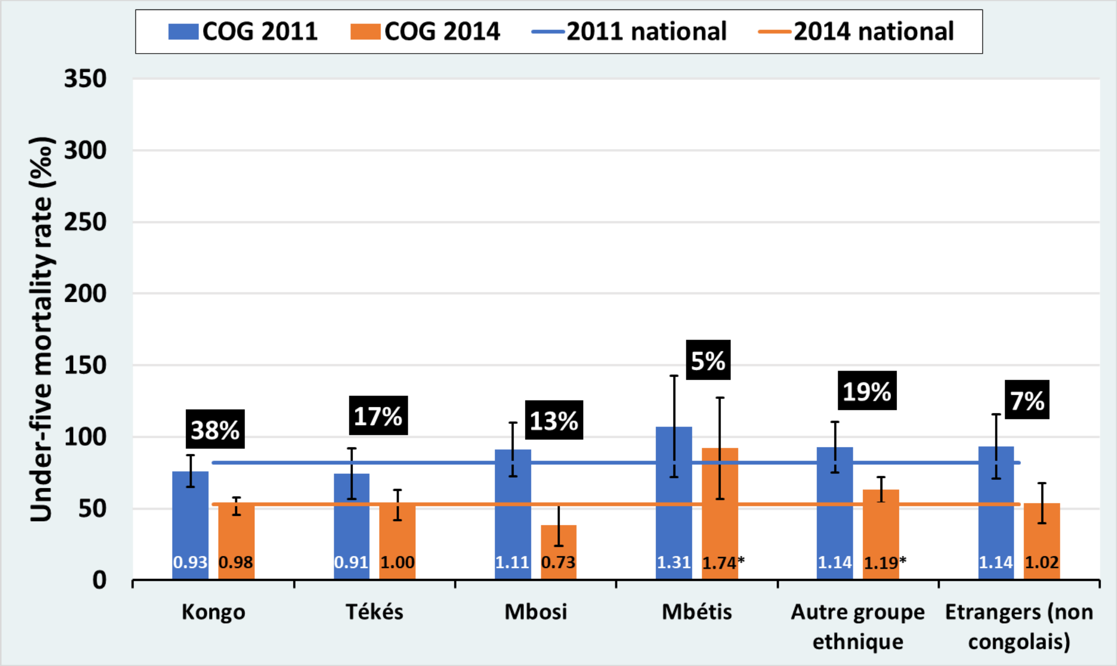

Dominican Republic

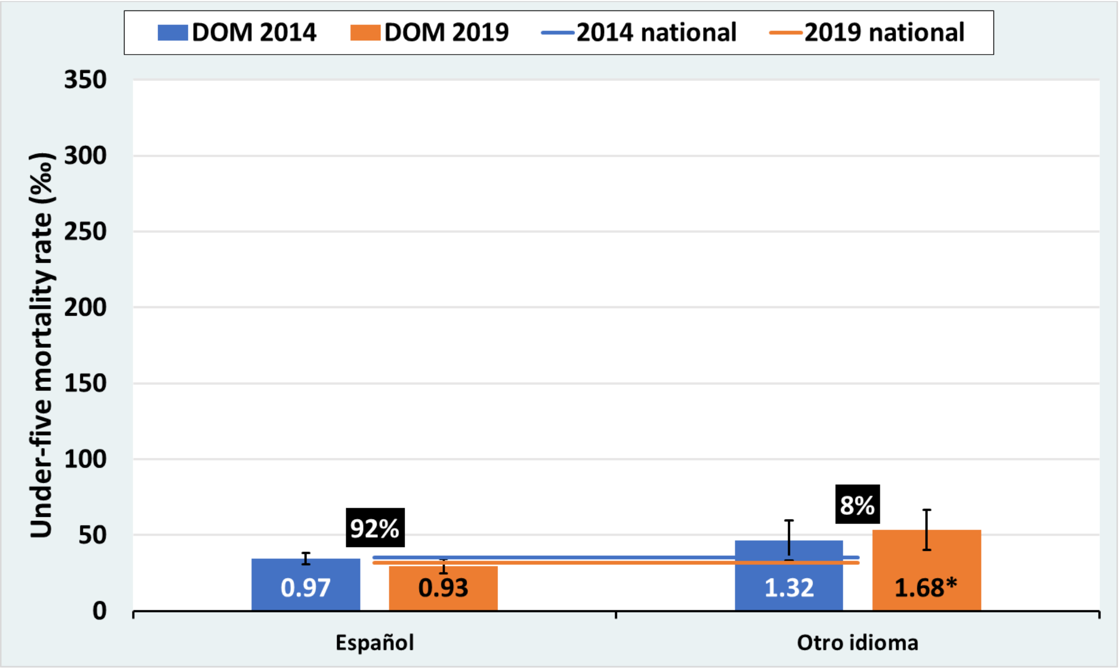

Ethiopia

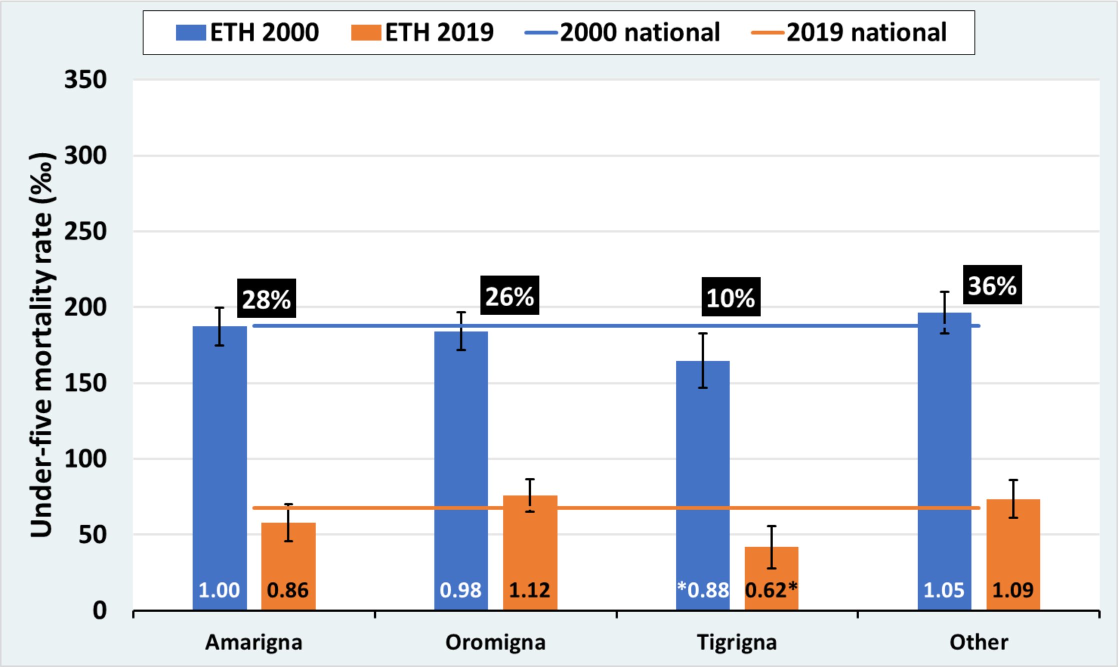

Gabon

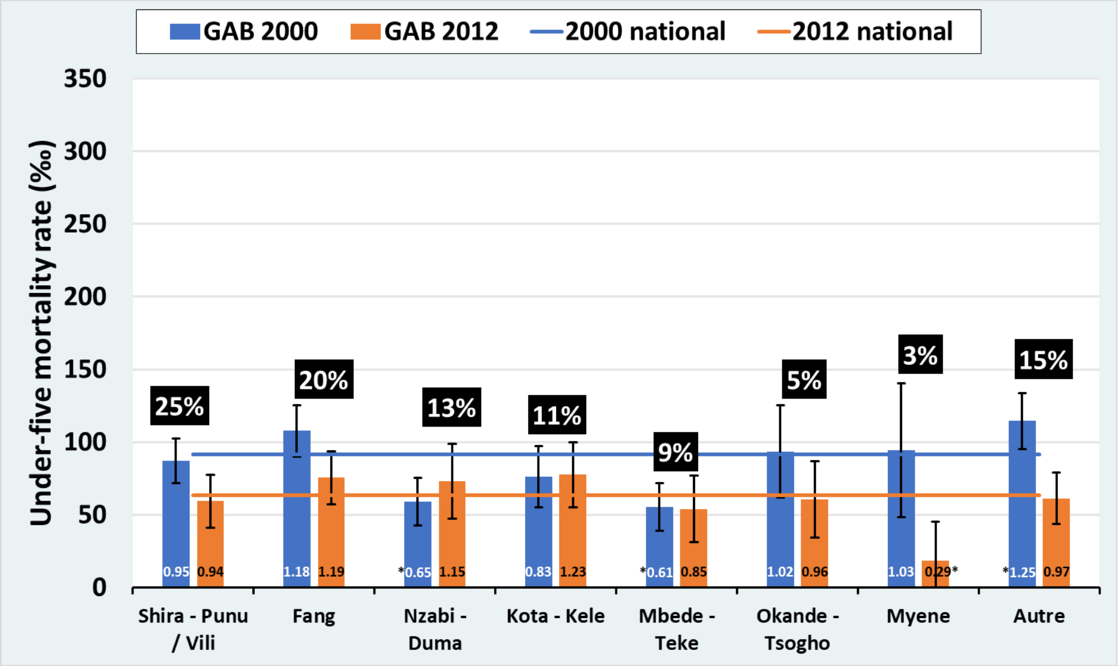

Gambia

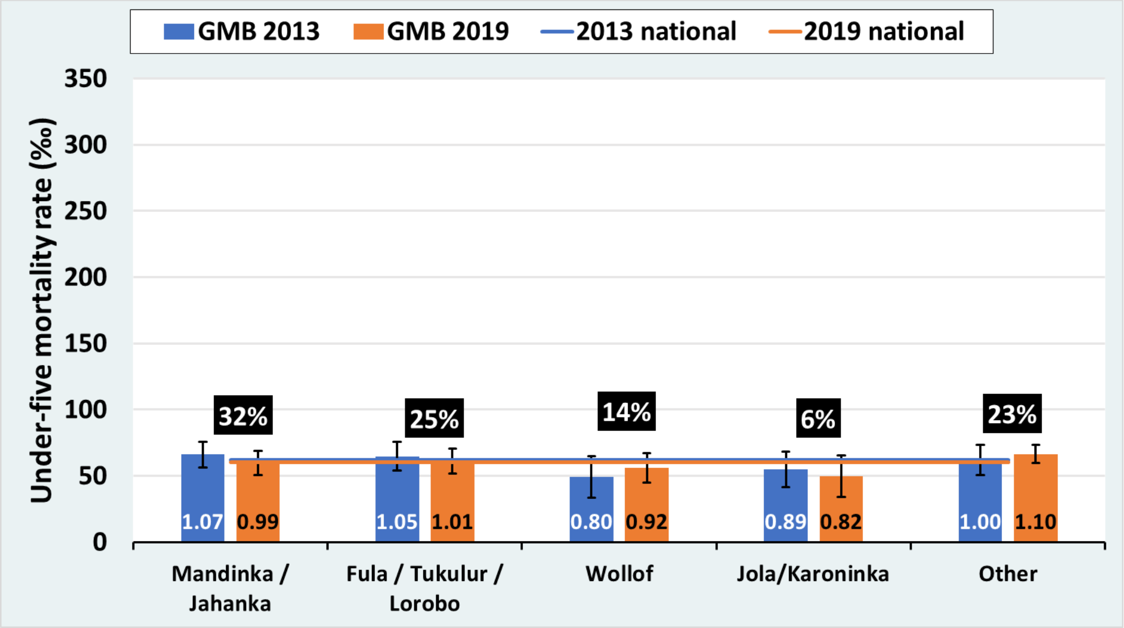

Ghana

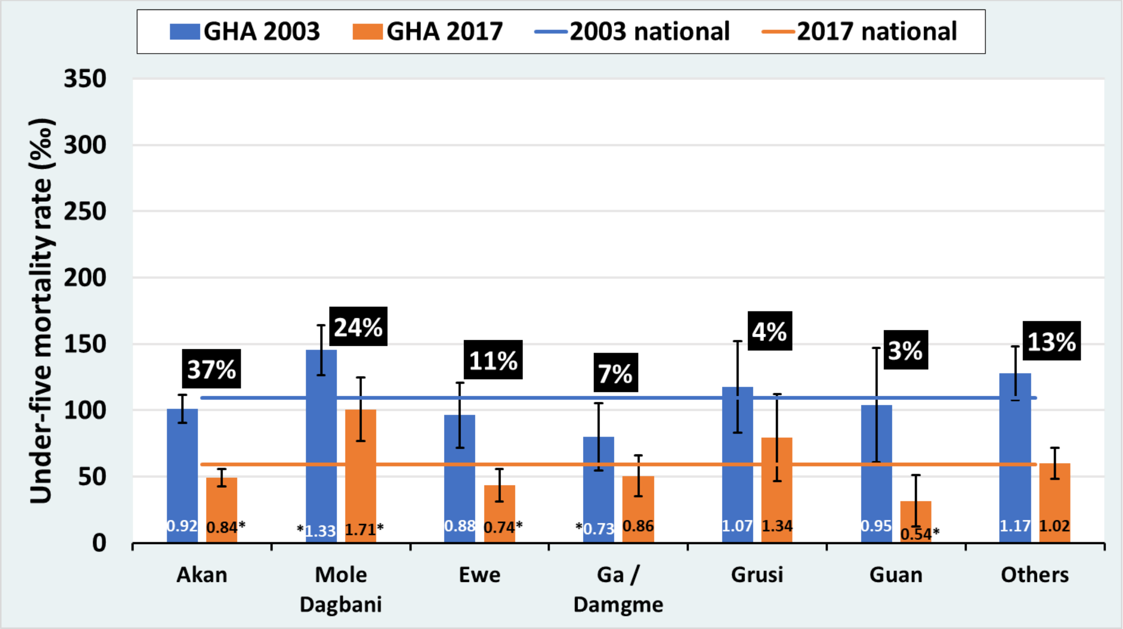

Guinea

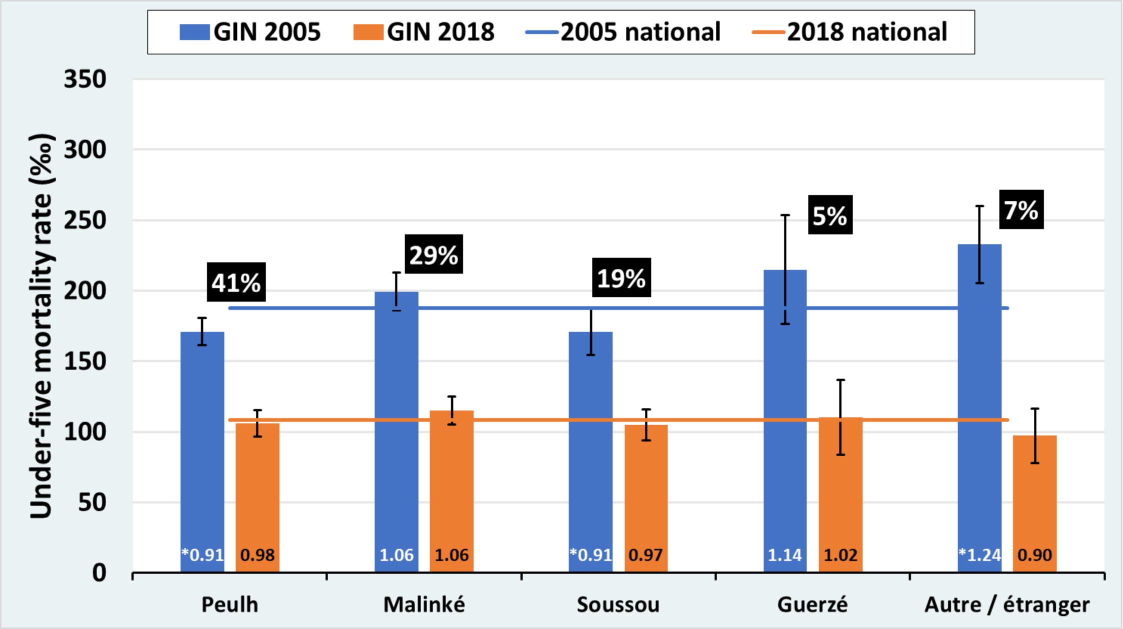

Guyana

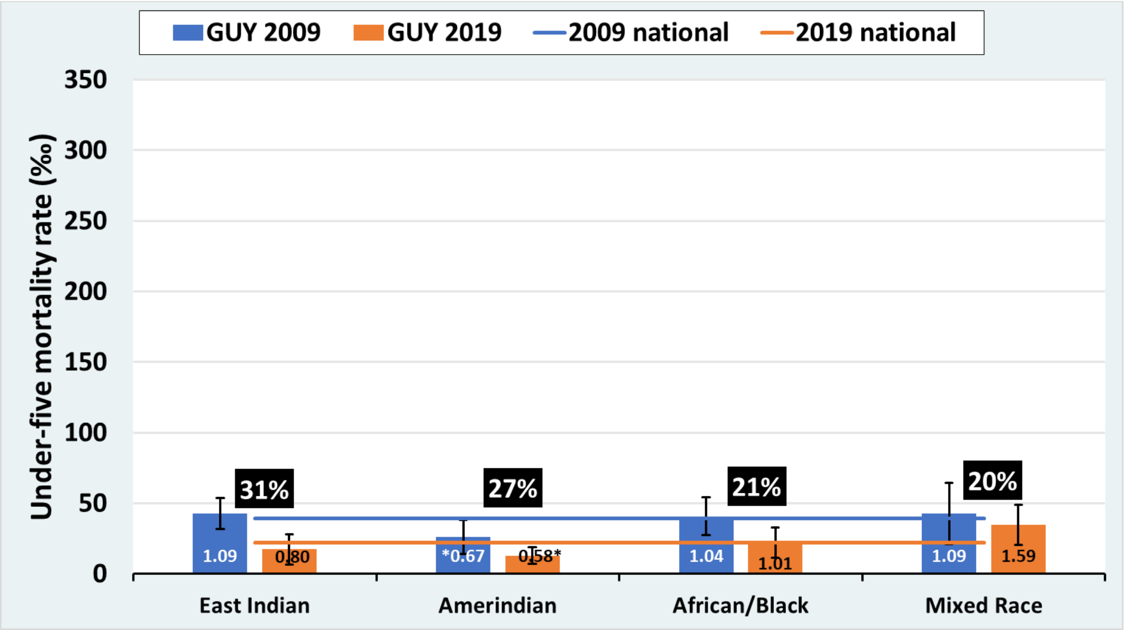

Honduras

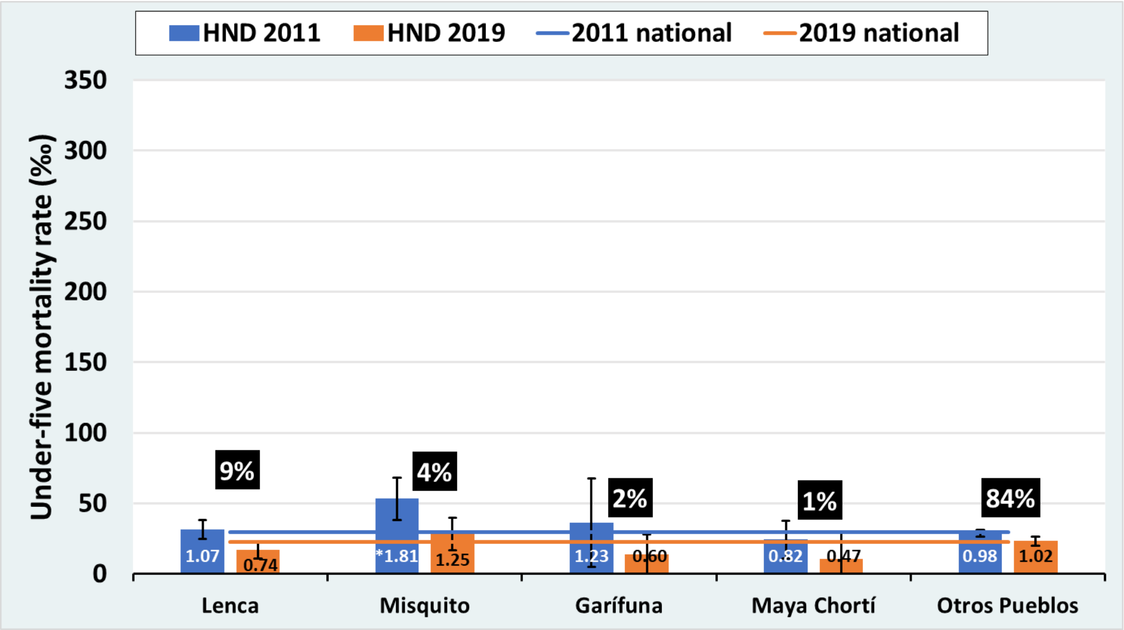

India

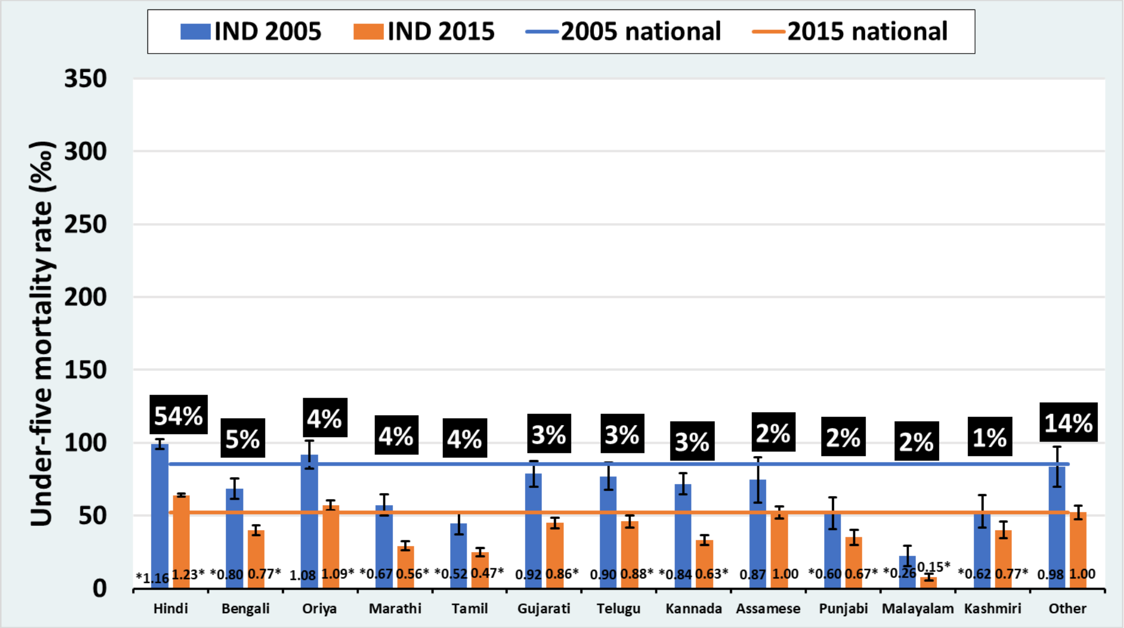

IND 2005
IND 2015
2005 national
2015 national
Under-five mortality rate (‰)
350
300
250
200
150
100
50
0
54%
5%
4%
4%
4%
3%
3%
3%
2%
2%
2%
1%
14%
1.16 1.23
0.80 0.77
1.08 1.09*
0.67 0.56
0.52 0.47
0.92 0.86*
0.90 0.88*
0.84 0.63
0.87 1.00
0.60 0.67
0.26 0.15
0.62 0.77
0.98 1.00
Hindi
Bengali
Oriya
Marathi
Tamil
Gujarati
Telugu
Kannada
Assamese
Punjabi
Malayalam
Kashmiri
Other

Kenya

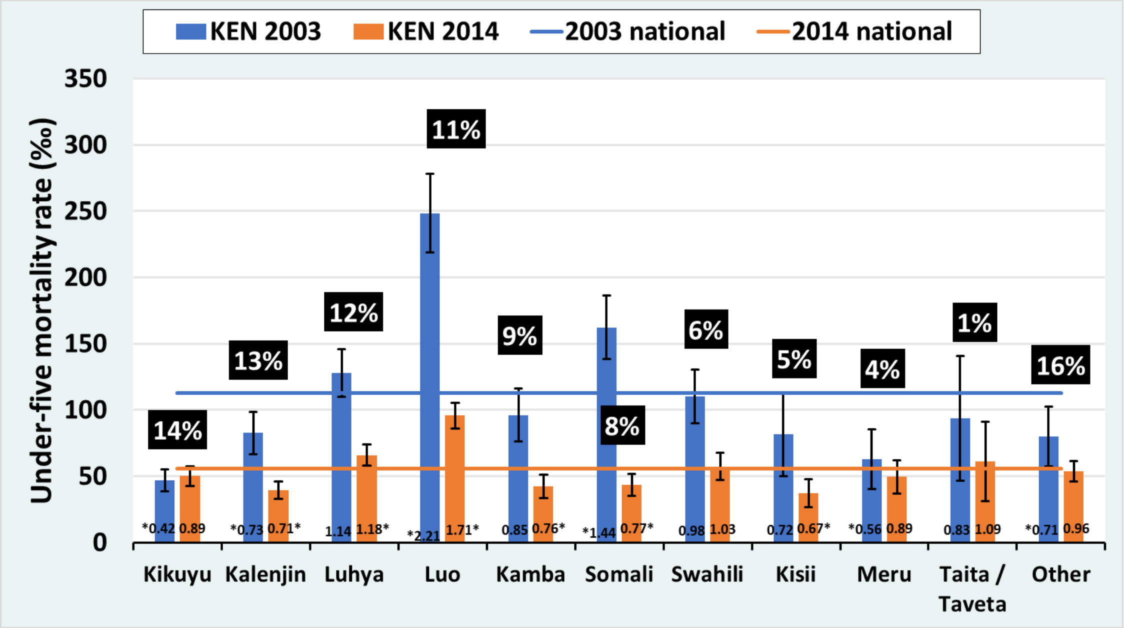

KEN 2003
KEN 2014
2003 national
2014 national
Under-five mortality rate (‰)
350
300
250
200
150
100
50
0
14%
13%
12%
11%
9%
8%
6%
5%
4%
1%
16%
*0.42 0.89
0.73 0.71
1.14 1.18*
2.21 1.71
0.85 0.76*
1.44 0.77
0.98 1.03
0.72 0.67*
*0.56 0.89
0.83 1.09
*0.71 0.96
Kikuyu
Kalenjin
Luhya
Luo
Kamba
Somali
Swahili
Kisii
Meru
Taita / Taveta
Other

Kosovo

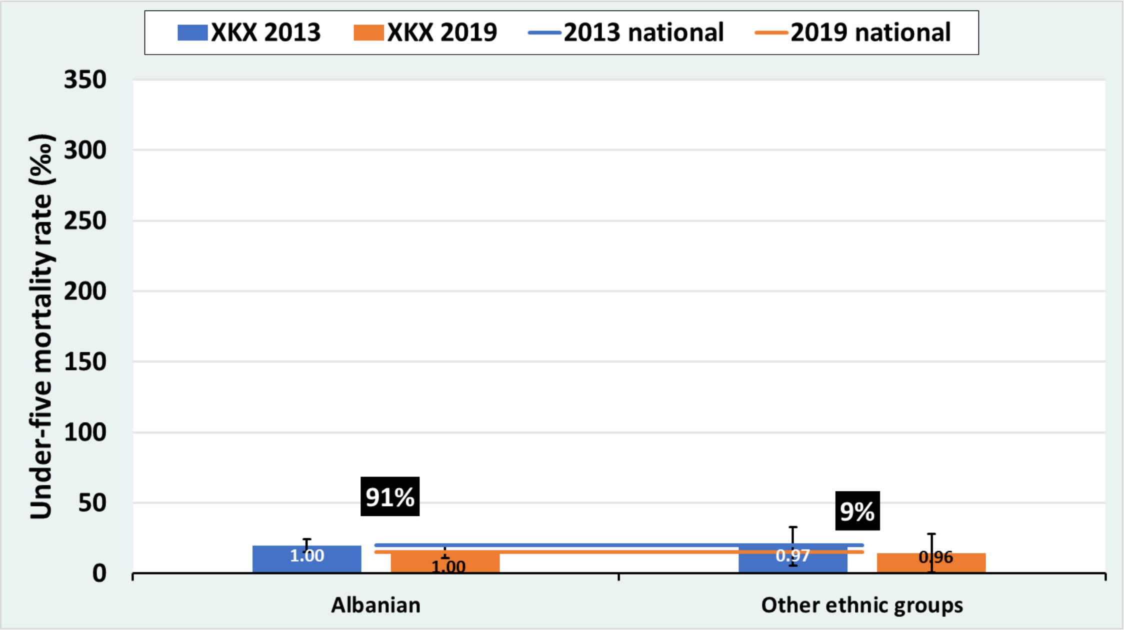

Kyrgyzstan

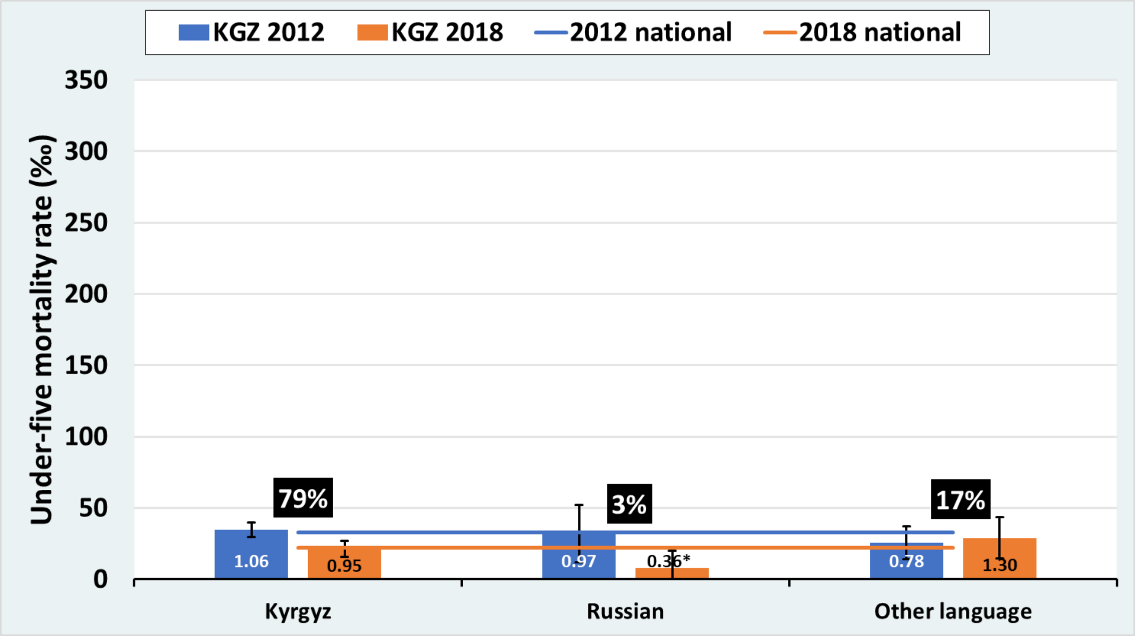

Lao PDR

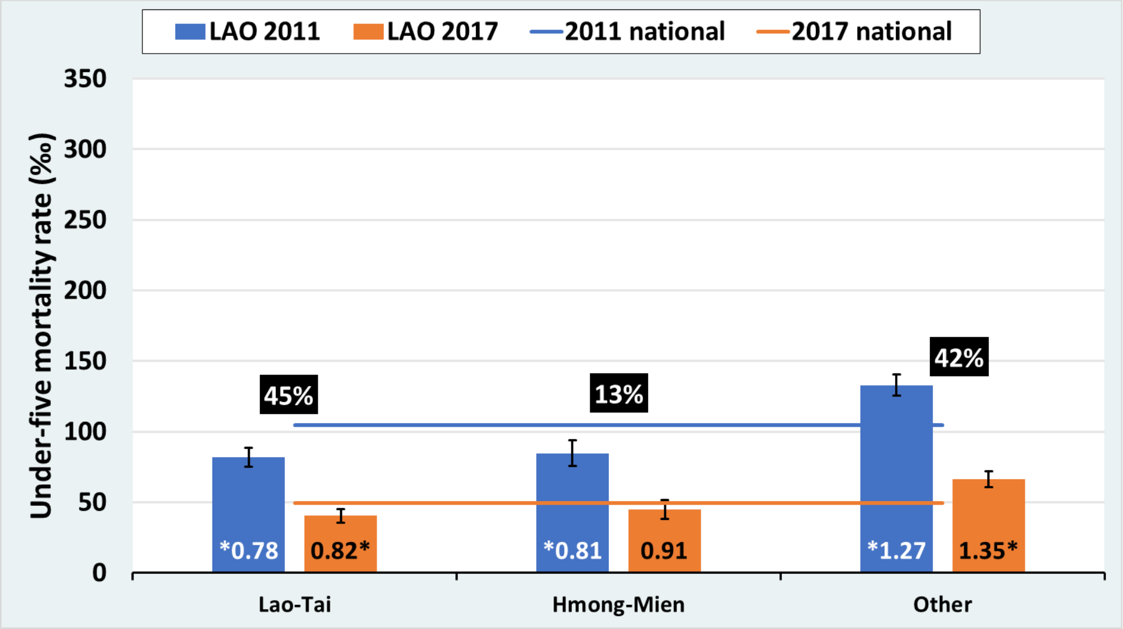

Malawi

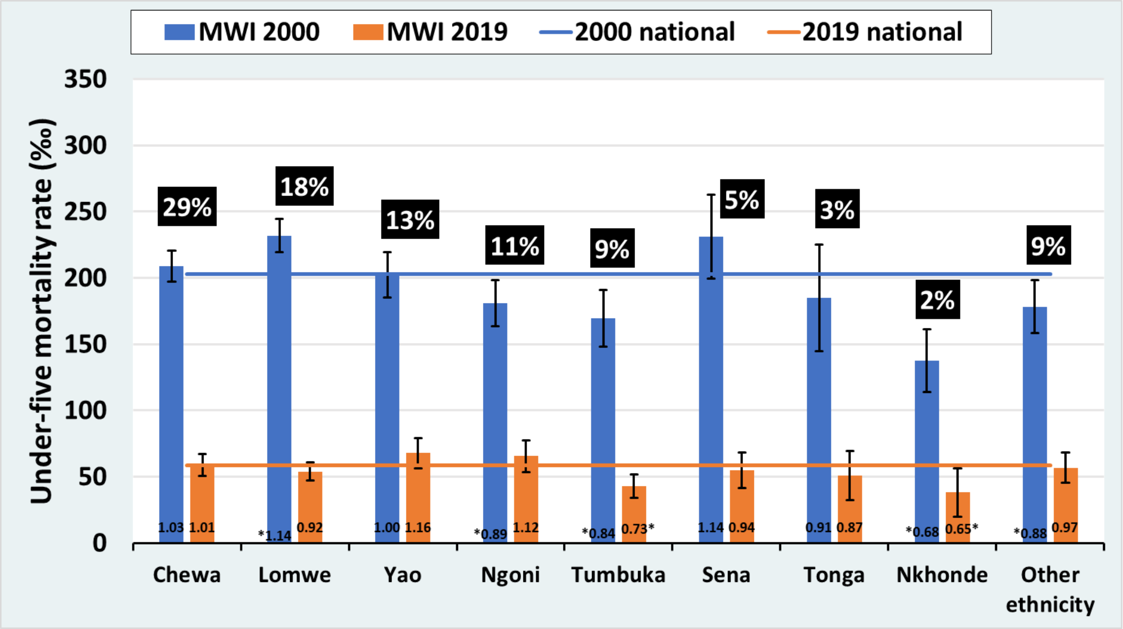

Mali

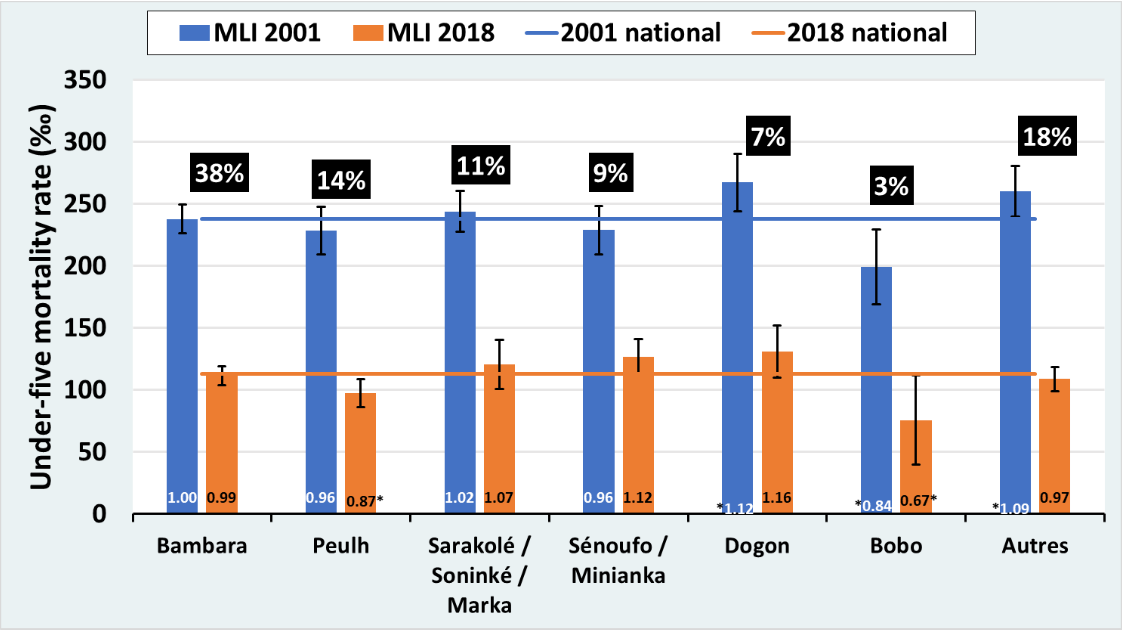

Mauritania

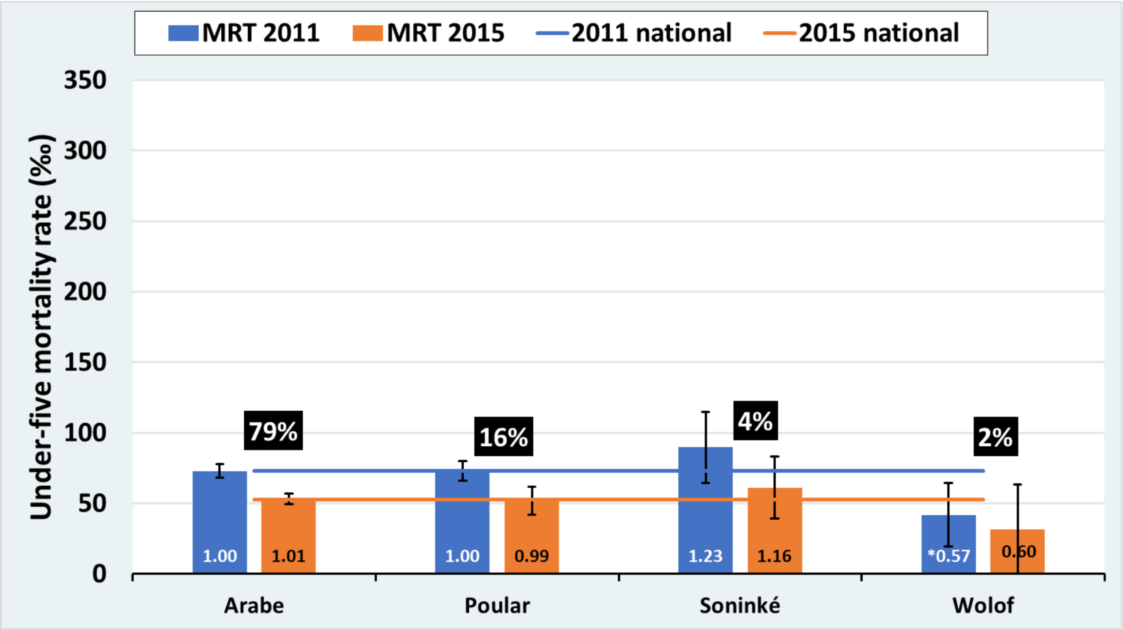

Moldova

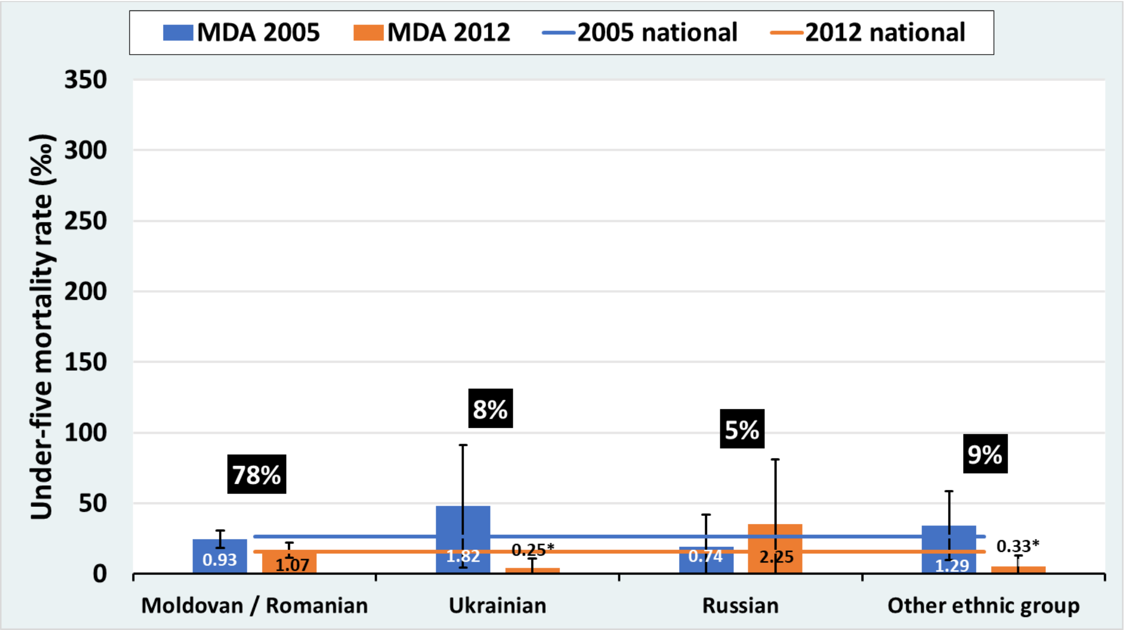

Mongolia

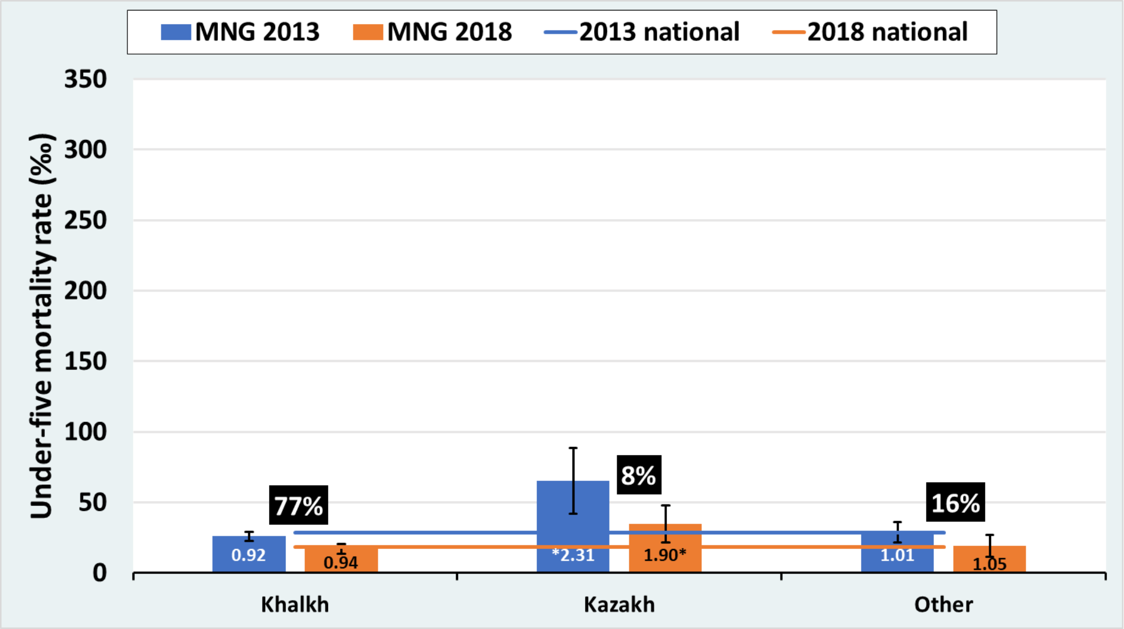

Mozambique

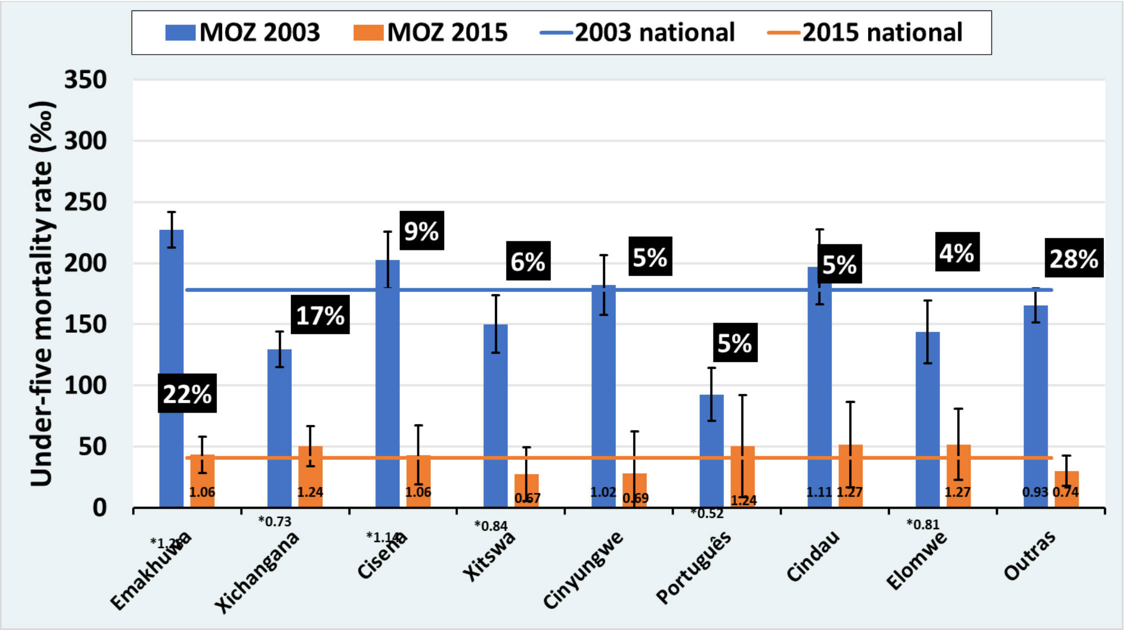

Namibia

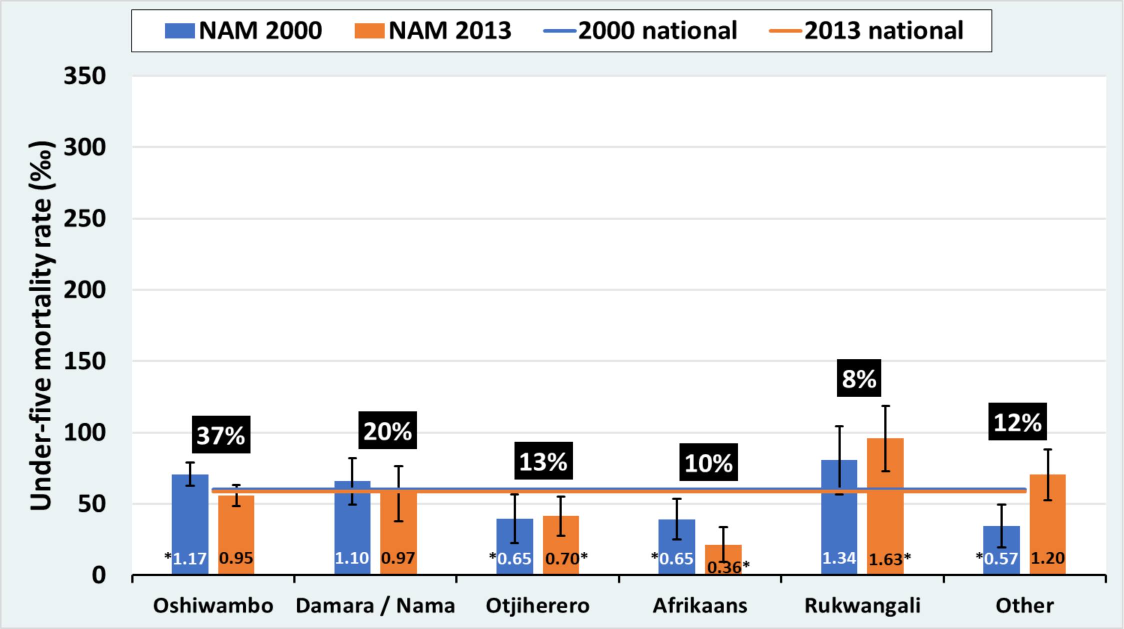

Nepal

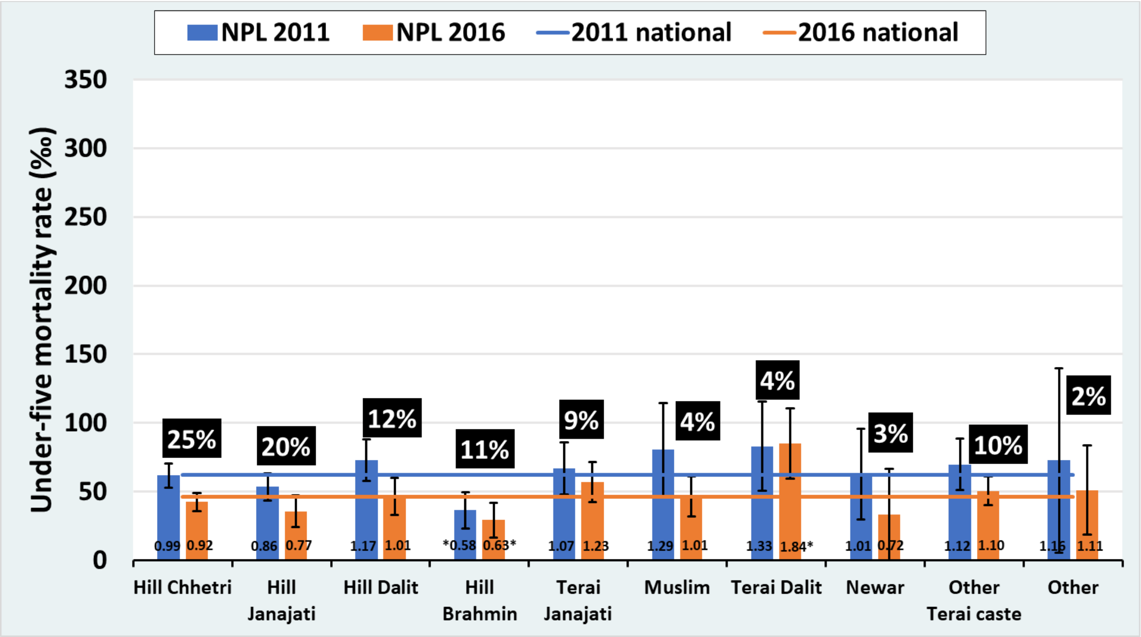
NPL 2011
NPL 2016
2011 national
2016 national
Under-five mortality rate (‰)
350
300
250
200
150
100
50
0
25%
20%
12%
11%
9%
4%
4%
3%
10%
2%
0.99
0.92
0.86
0.77
1.17
1.01
*0.58
0.63*
1.07
1.23
1.29
1.01
1.33
1.84*
1.01
0.72
1.12
1.10
1.16
1.11
Hill Chhetri
Hill Janajati
Hill Dalit
Hill Brahmin
Terai Janajati
Muslim
Terai Dalit
Newar
Other Terai caste
Other

Niger

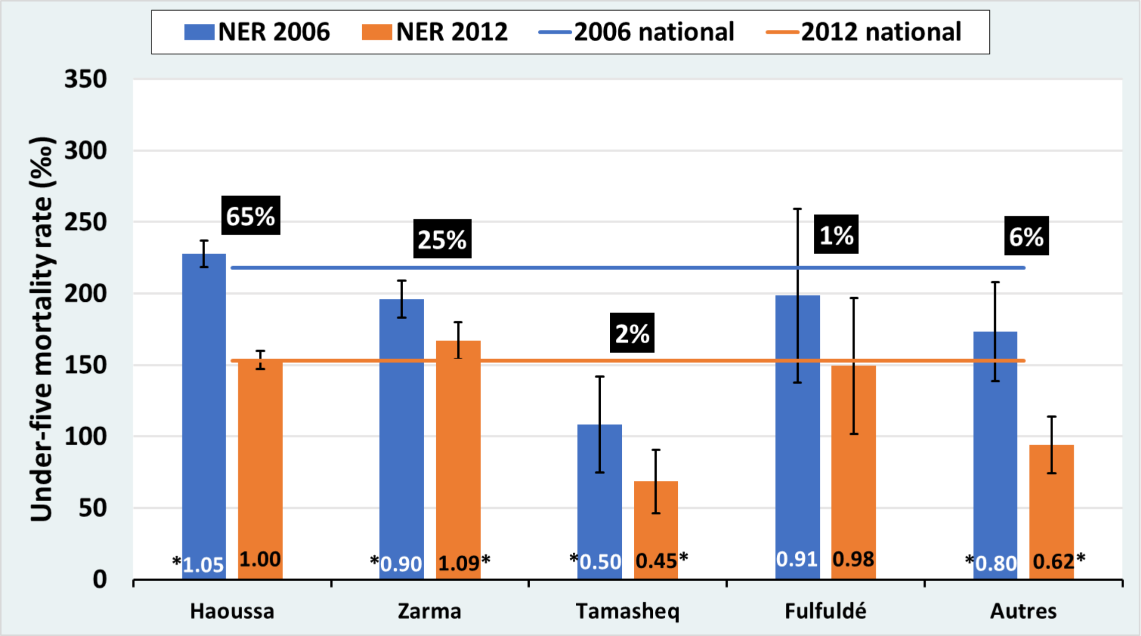
NER 2006
NER 2012
2006 national
2012 national
Under-five mortality rate (‰)
350
300
250
200
150
100
50
0
65%
25%
2%
1%
6%
*1.05
1.00
*0.90
1.09*
*0.50
0.45*
0.91
0.98
*0.80
0.62*
Haoussa
Zarma
Tamasheq
Fulfuldé
Autres

Nigeria

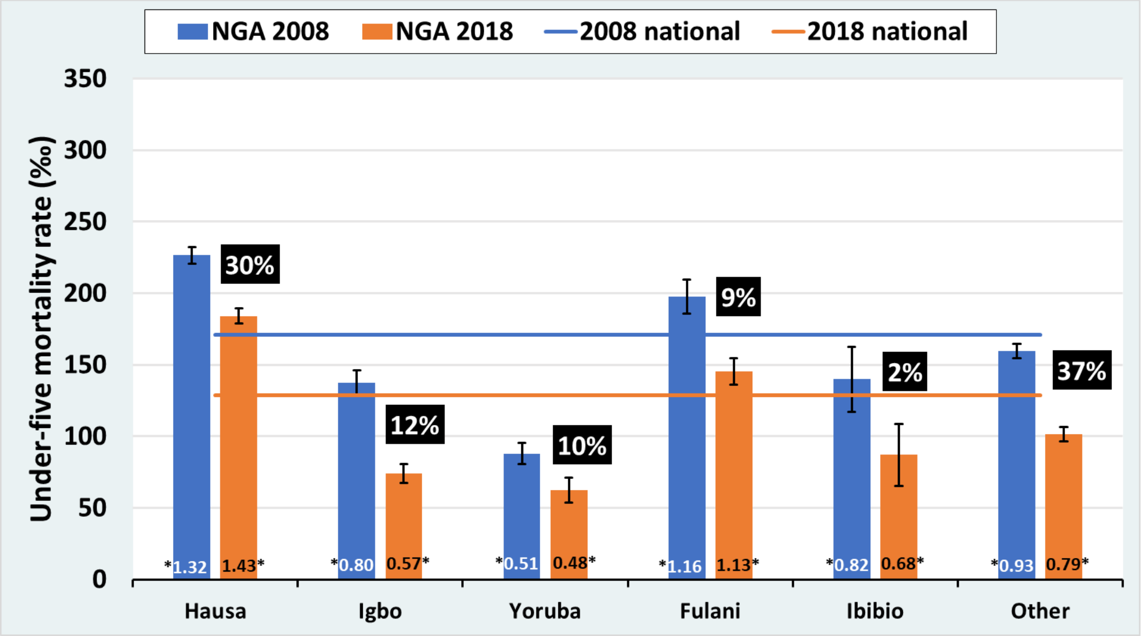

Pakistan

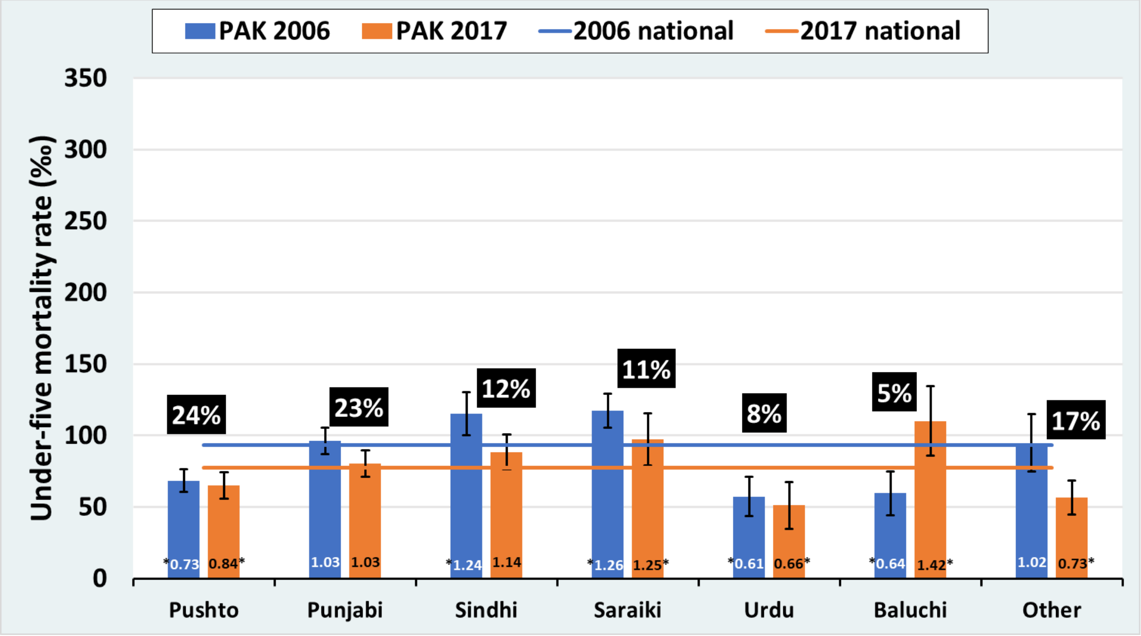

Peru

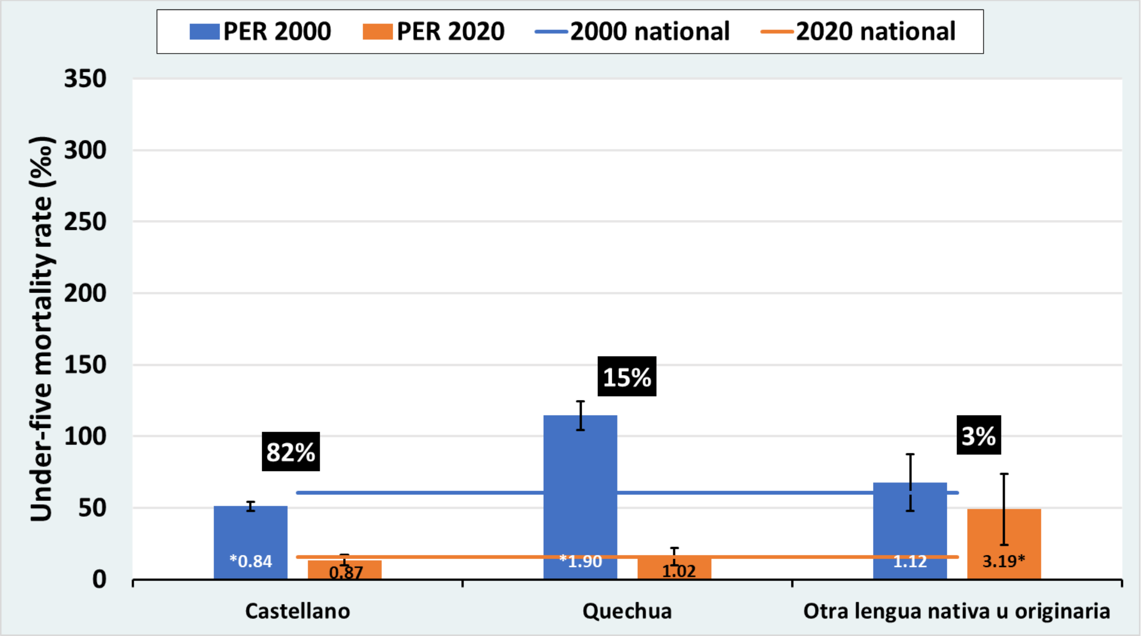

Philippines

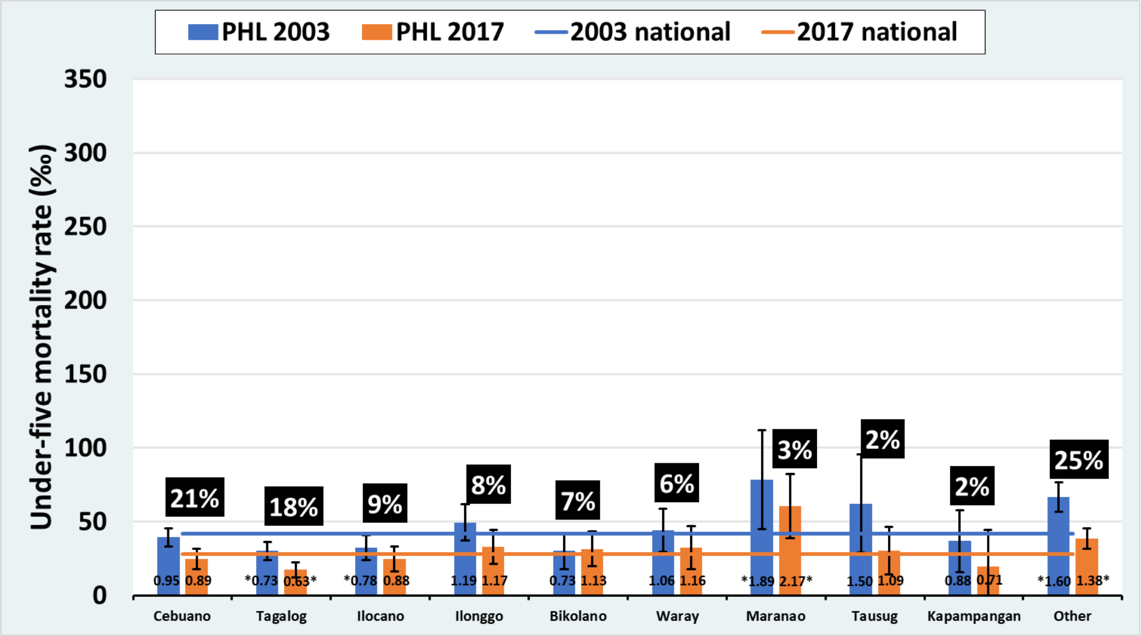

Sao Tome and Principe

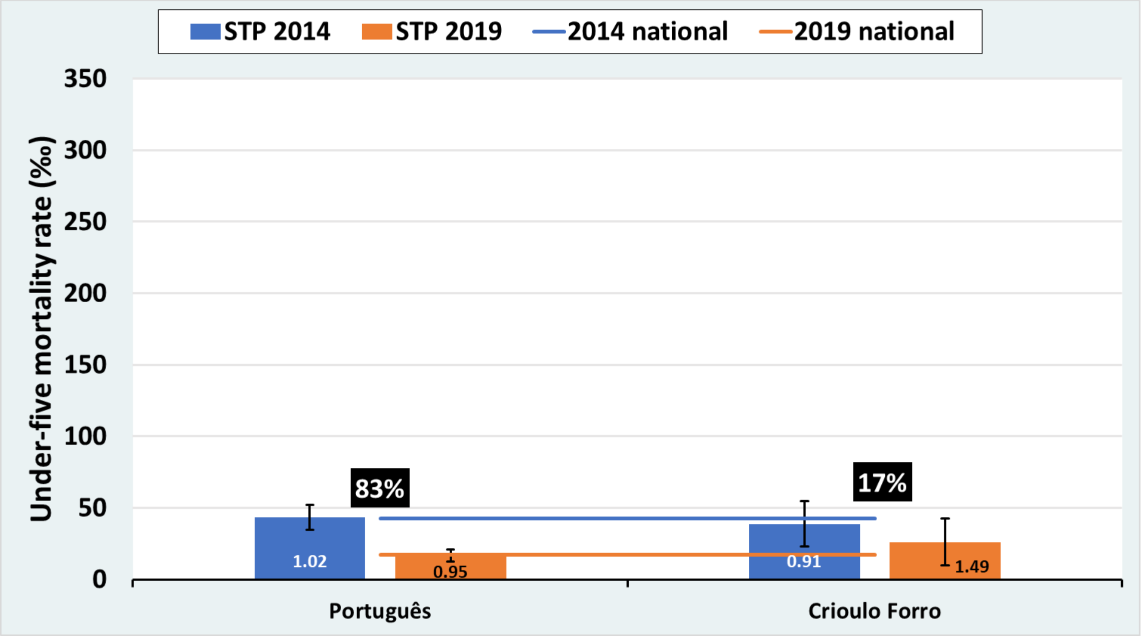

Senegal

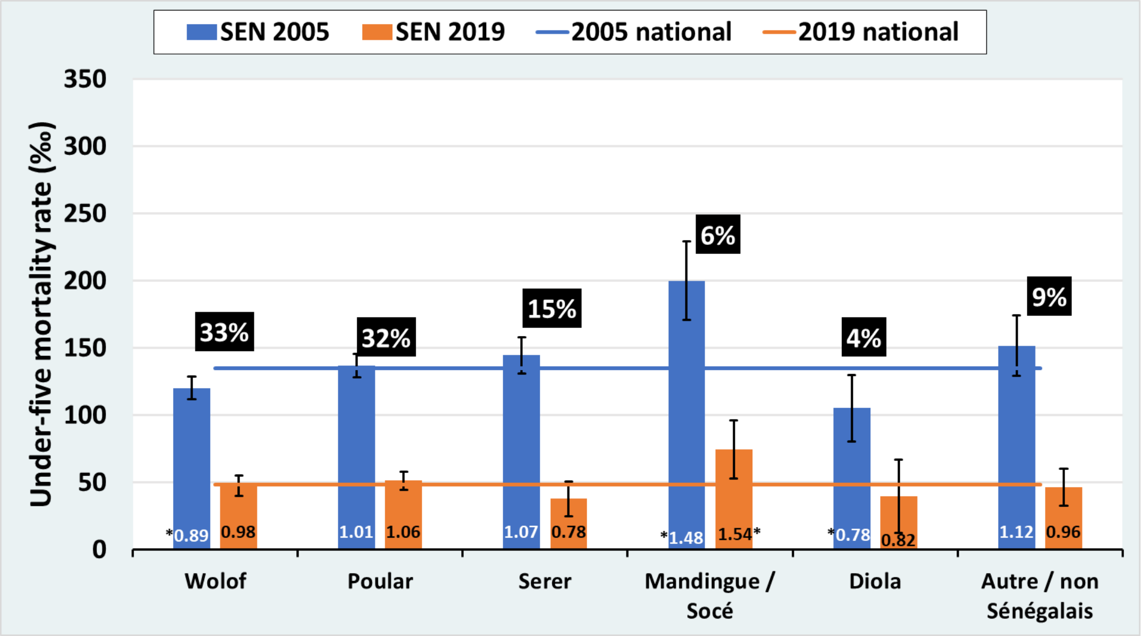

Sierra Leone

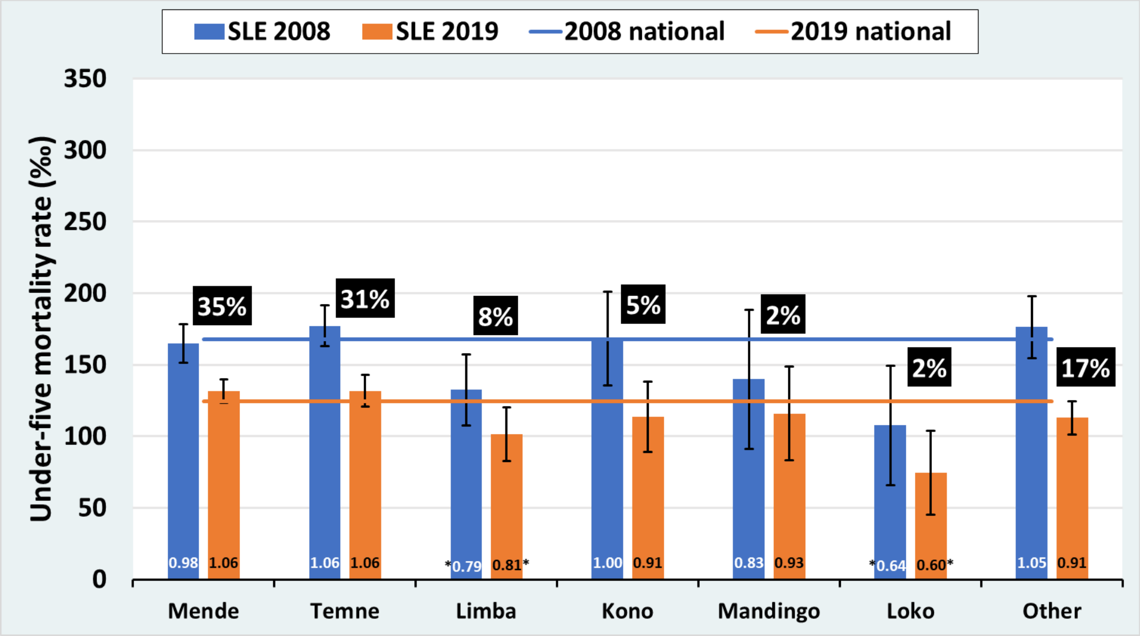

Tajikistan

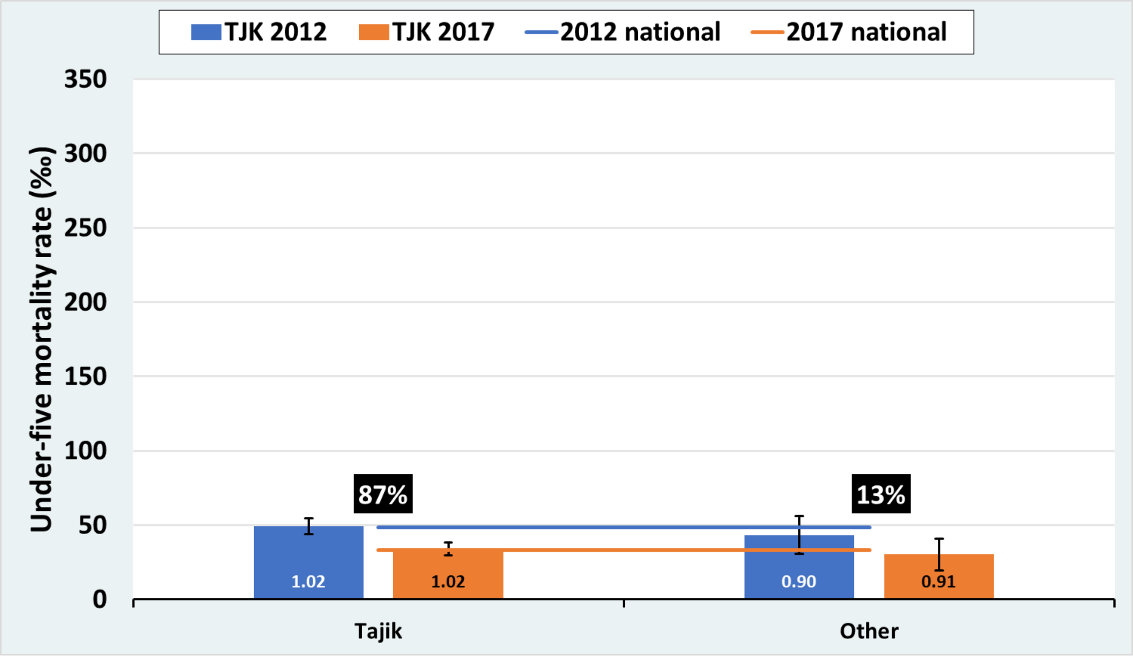

Timor-Leste

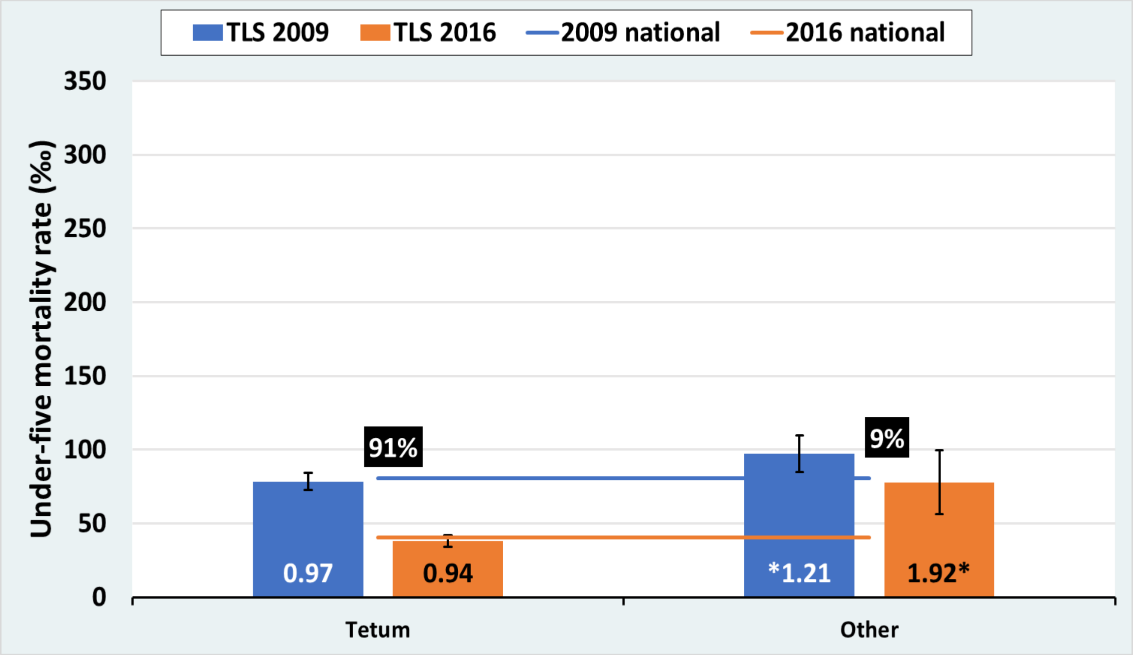

Togo

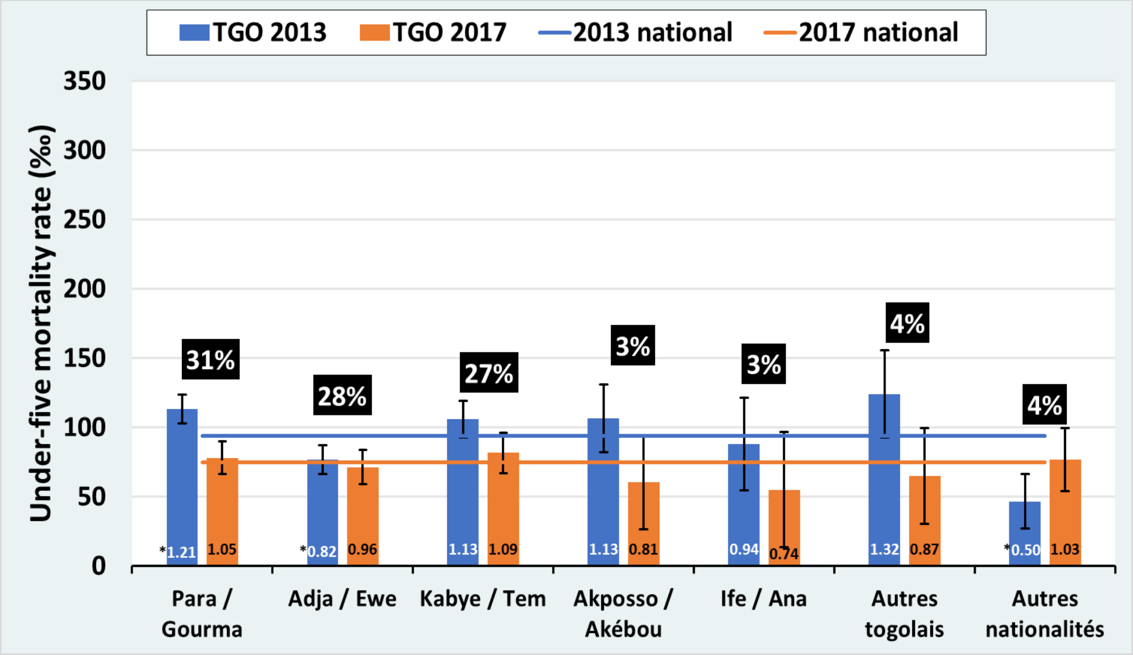

Türkiye

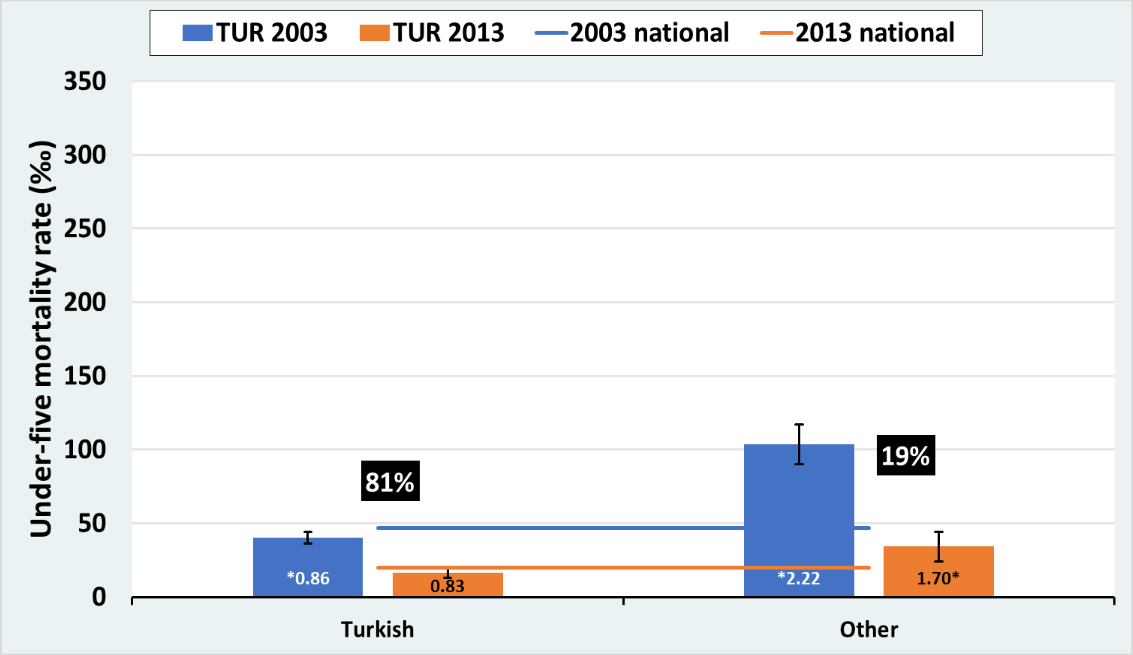

Turkmenistan

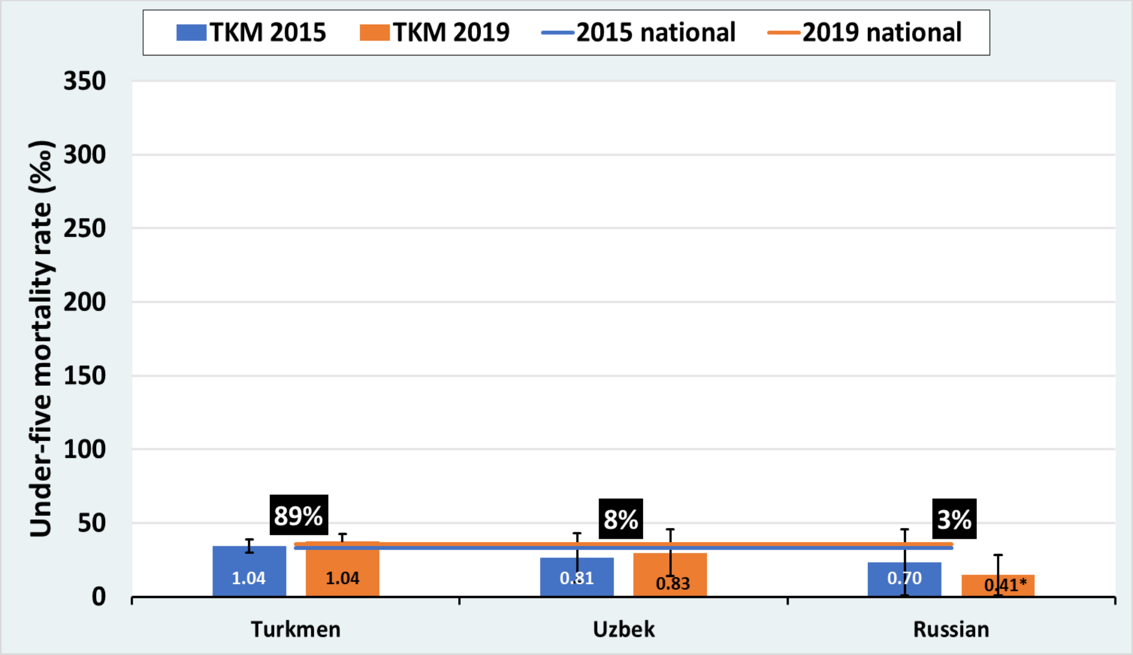

Uganda

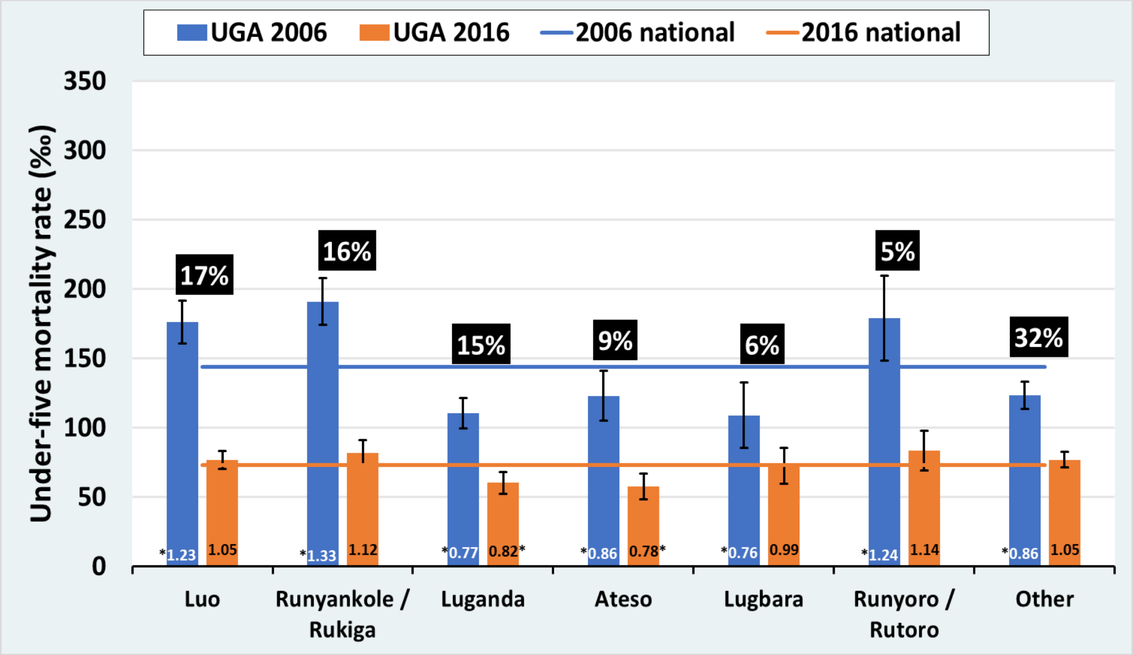

Vietnam

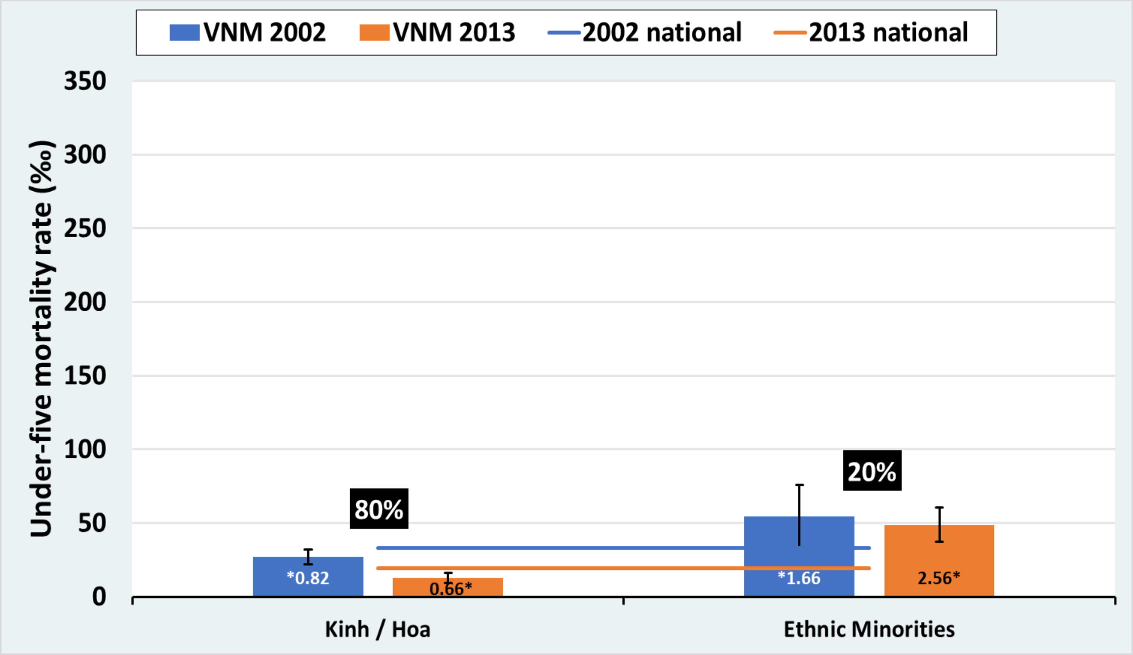

Zambia

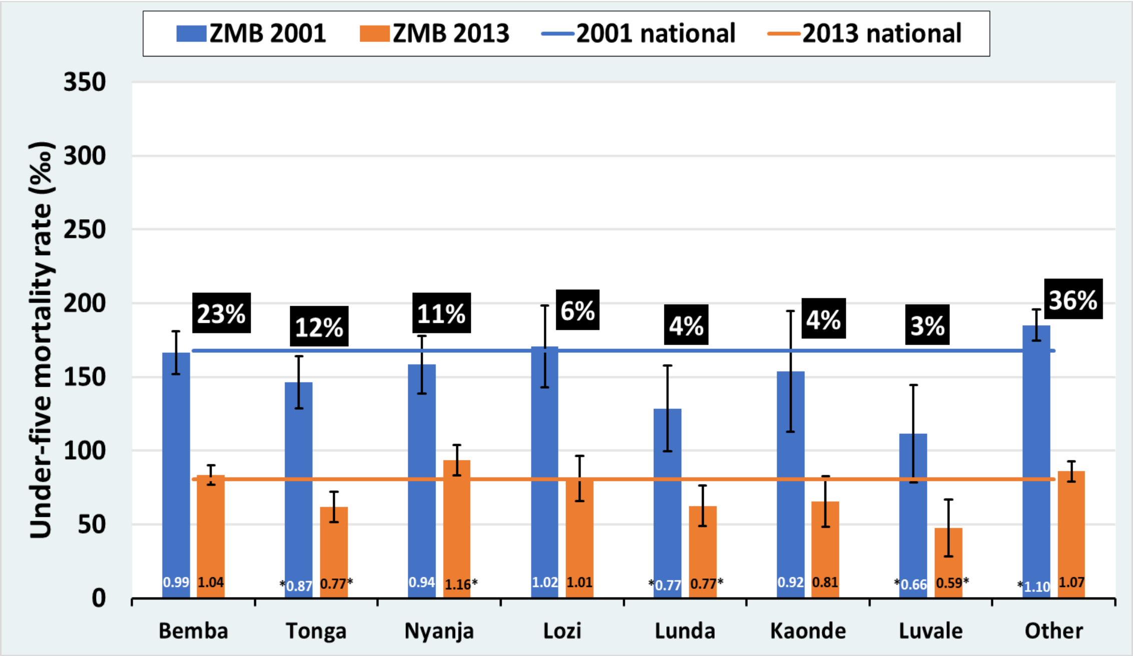

Zimbabwe

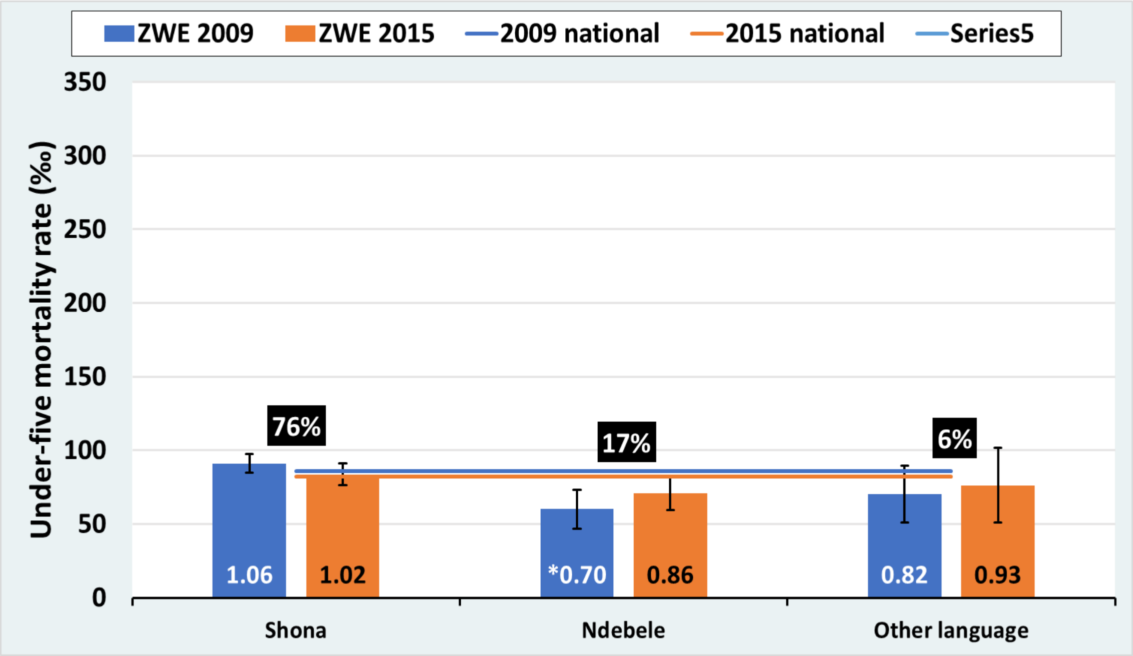

Supplement: Supplementary file 6 — Additional file 6. Title: Under-five mortality rate by ethnic groups in the first and last surveys. Results for selected countries. The numbers in the black rectangles show the average proportion of the samples for each ethnic group in the two surveys. The numbers at the bottom of the bars show the ratio between the rate in a particular ethnic group and the national rate for that point in time. Description: One graph for each country showing under-five mortality rate (U5MR) by ethnic groups in the first and last surveys. [file 12939_2023_1888_MOESM6_ESM.pdf]
